# 6-Amino-2,4,5-trimethylpyridin-3-ol and 2-amino-4,6-dimethylpyrimidin-5-ol derivatives as selective fibroblast growth factor receptor 4 inhibitors: design, synthesis, molecular docking, and anti-hepatocellular carcinoma efficacy evaluation

**DOI:** 10.1080/14756366.2022.2048378

**Published:** 2022-03-17

**Authors:** Chhabi Lal Chaudhary, Dongchul Lim, Prakash Chaudhary, Diwakar Guragain, Bhuwan Prasad Awasthi, Hee Dong Park, Jung-Ae Kim, Byeong-Seon Jeong

**Affiliations:** a College of Pharmacy, Yeungnam University, Gyeongsan, Republic of Korea; b Innovo Therapeutics Inc, Daejeon, Republic of Korea

**Keywords:** Aminotrimethylpyri(mi)dinol, FGFR4 kinase, molecular docking, hepatocellular carcinoma, anti-tumour

## Abstract

A novel series of aminotrimethylpyridinol and aminodimethylpyrimidinol derivatives were designed and synthesised for FGFR4 inhibitors. Structure-activity relationship on the FGFR4 inhibitory activity of the new compounds was clearly elucidated by an intensive molecular docking study. Anti-cancer activity of the compounds was evaluated using hepatocellular carcinoma (HCC) cell lines and a chick chorioallantoic membrane (CAM) tumour model. Compound **6O** showed FGFR4 inhibitory activity over FGFR1 − 3. Compared to the positive control BLU9931, compound **6O** exhibited at least 8 times higher FGFR4 selectivity. Strong anti-proliferative activity of compound **6O** was observed against Hep3B, an HCC cell line which was a much more sensitive cell line to BLU9931. *In vivo* anti-tumour activity of compound **6O** against Hep3B-xenografted CAM tumour model was almost similar to BLU9931. Overall, compound **6O**, a novel derivative of aminodimethylpyrimidinol, was a selective FGFR4 kinase inhibitor blocking HCC tumour growth.

## Graphical abstract

**Figure:**
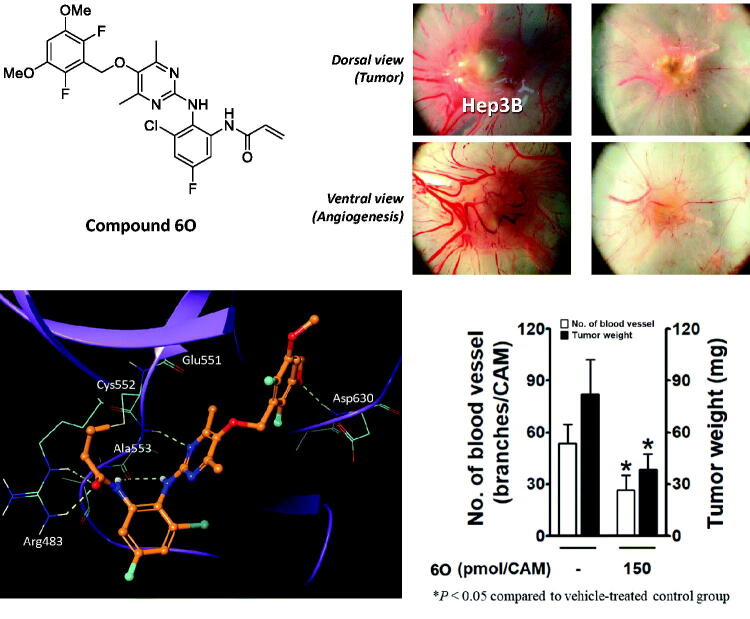

A series of methylated aminopyri(mi)dine compounds were designed and synthesised as FGFR4 inhibitors.

Compound **6O** showed a selective FGFR4 inhibitory activity over FGFR1 ∼ 3.

Dimethyl groups at the pyrimidine ring of compound **6O** prohibited a proper conformation for covalent bonding to FGFR1 ∼ 3.

The smaller size of fluorine at the dimethoxyphenyl ring in compound **6O** gives suitable conformation for strong binding interaction with FGFR4.

Compound **6O** inhibited Hep3B tumour growth xenografted on chick chorioallantoic membrane (CAM) tumour model

## 1. Introduction

Hepatocellular carcinoma (HCC) has been increasingly diagnosed over the past few decades and has become the third leading cause of cancer-related mortality worldwide^1^. After sorafenib (a multi-tyrosine kinase inhibitor) was authorised for the first-line treatment option of HCC treatment^2^, several multi-tyrosine kinase inhibitors including cabozantinib have been approved for patients with advanced-stage HCC^3^. In addition, PD-1 immune checkpoint inhibitors, such as nivolumab and pembrolizumab, have been added as therapeutic options in HCC treatment^3^. Despite these drugs being approved as the first-line or second-line therapy, tumour regression by immune checkpoint blockade only occurs in a limited proportion of patients^4^.

Fibroblast growth factor receptor 4 (FGFR4), one of the families of fibroblast growth factor receptors, is a tyrosine kinase receptor with a distinct 802 amino acid sequence. Normally, FGFR4 expression level is high during foetal development and drastically reduced thereafter^5^. Most of FGF family members, except FGF11 subfamily, are ligands of FGFR4^6^. FGF19, which is produced from the ileum as a postprandial hormone, has a more specific selective affinity to FGFR4 than the other FGF ligands. In contrast to the normal production and action of FGF19 as an endocrine hormone, FGF19 is overexpressed and co-expressed with FGFR4 in various cancers of liver, breast, lung, bladder, head, and neck^7–10^, indicating *FGF19* as a driver oncogene. Similarly, amplification of *FGFR4* gene is the predominant type and accounts for 78% of all *FGFR4* gene alterations in various cancers^11–14^. In addition, a significant correlation between overexpression of FGFR4 in tumour tissues and patients’ poor survival rate indicates *FGFR4* is also acting as an oncogene^15^^,^^16^.

**Figure 1.**
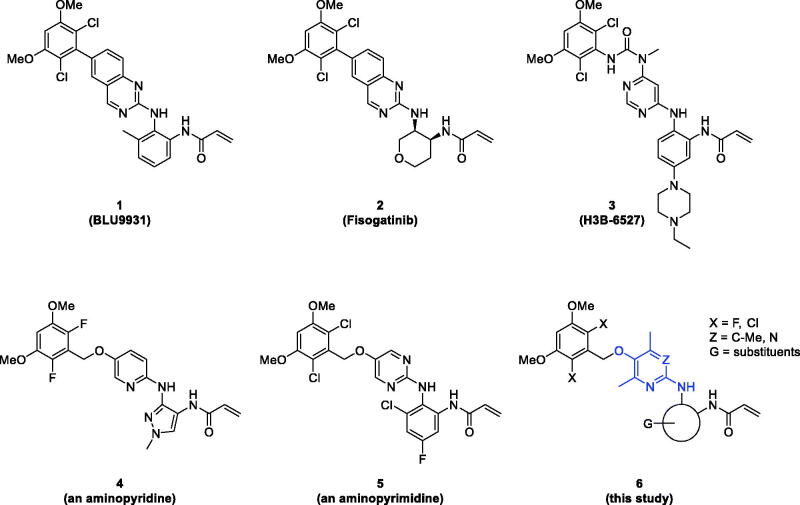
Representative selective and irreversible FGFR4 inhibitors (**1** ∼ **5**) and general structure (**6**) of novel compounds prepared in this study.

Numbers of research for the discovery of selective FGFR4 inhibitors have underlined the covalent interaction between Cys552 residue in the middle-hinge domain of the ATP-binding site of FGFR4 and acrylamide moiety of small molecule compounds, which was supported by X-ray co-crystal structures. BLU9931 (**1**) was discovered as a notable compound to possess potent FGFR4 inhibitory activity with good selectivity over FGFR1 ∼ 3. After that, a number of analogues of BLU9931 were designed and prepared, where fisogatinib (BLU554) (**2**) with better physicochemical properties has been evaluated in a clinical trial for the treatment of HCC. In addition, other Cys552 − acrylamide inhibitors such as H3B-6527 (**3**), aminopyrimidines (**4**), and aminopyridines (**5**) are undergoing either preclinical or clinical studies in recent years (Figure 1) ^17–26^. Multiple pan-FGFR inhibitors have shown weak efficacy against FGFR4 together with toxicity (hyperphosphatemia and soft tissue mineralization) due to activity against FGFR1 ∼ 3, which indicates a strong need for the discovery of FGFR4-selective inhibitors^27^.

We recently reported a string of anti-HCC compounds where bioisosterism was employed as the main strategy for the design of new compounds^28^^,^^29^. Bioisosterism is one of the efficient and practical strategies of new drug discovery. Replacement of a functional group of a lead compound may improve efficacy by changes in binding mode to molecular target, metabolic stability by modifying structure of labile sites, or physicochemical properties, etc^30^^,^^31^. In our previous studies, we designed and prepared a series of bioisosteres of sorafenib and cabozantinib for the discovery of anti-HCC agents, respectively^28^^,^^29^. As part of our on-going project of the discovery of new drug by bioisosteric replacement of a benzene, pyridine, or pyrimidine core ring of well-known drugs or promising compounds in the development stage with methyl-containing pyridine/pyrimidine rings, the present study was focussed on the synthesis and evaluation of a new series of aminotrimethylpyridinol- and aminodimethylpyrimidinol-derived FGFR4 inhibitors which are depicted as a general structure (**6**) in Figure 1. In addition, a molecular docking study was done in-depth for an explanation of the difference in FGFR4 inhibitory activity with structural change.

## 2. Results and discussion

### 2.1. Synthesis

**Figure 2.**
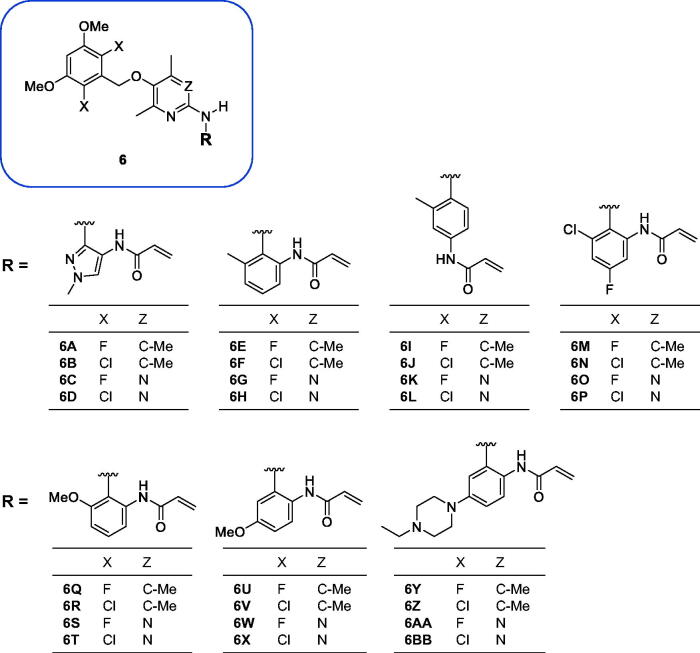
New compounds designed and synthesised for this study.

Figure 2 shows the structures of twenty-eight new compounds for this study. They were designed based on common aminopyridine/aminopyrimidine backbones of about hundred compounds such as compounds **4** and **5** in Figure 1 introduced in the literature^21–23^. The pyridine/pyrimidine centred rings were replaced by trimethylpyridine/dimethylpyrimidine, which was the major structural variation. Fluorine and chlorine were employed at the halogen position in the dimethoxy ring part and seven kinds of acryl amide-containing aromatic rings were combined.

**Scheme 1.**
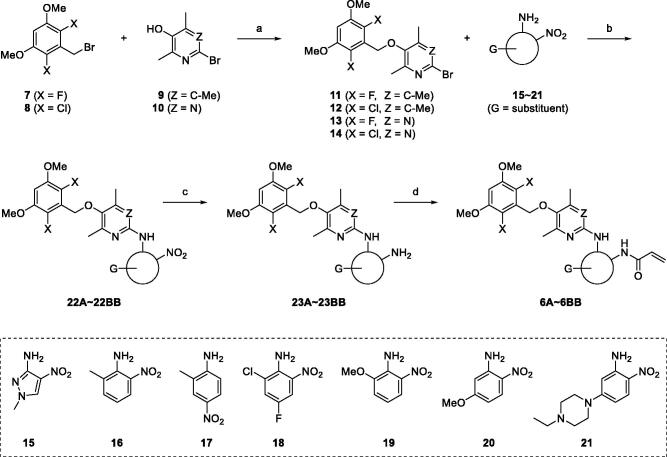
General synthetic scheme for **6A**∼**6BB**. *Reagents and Conditions*: (a) K_2_CO_3_, DMF, 80 °C; (b) Pd_2_(dba)_3_, BINAP, NaO*^t^*Bu, PhMe, reflux or Pd_2_(dba)_3_, xantphos, Cs_2_CO_3_, PhMe, reflux; (c) SnCl_2_·2H_2_O, EtOH, reflux or Zn, AcOH, DCM, r.t.; (d) acrylic acid, T3P, Et_3_N, DMF, 100 °C or acryloyl chloride, Et_3_N, DCM, 0 °C to r.t.

We thought that, among several synthetic methods shown in the literature on aminopyri(mi)dine FGFR4 inhibitors, the synthetic strategies described in the Eisai's patent specification of Eisai Co., Ltd^21^. were most suitable for preparing our new compounds (**6**). All twenty-eight final target compounds (**6A** ∼ **6BB**) were prepared by the synthetic method of Scheme 1 depicted as general structures for convenience. In brief, 3,5-dimethoxy-2,6-dihalobenzyl bromides (**7**, **8**) were combined with 6-bromo-2,4,5-trimethylpyridin-3-ol (**9**) or 2-bromo-4,6-dimethylpyrimidin-5-ol (**10**) to afford the four common intermediates **11** ∼ **14**. Combination of these four bromo-intermediates (**11** ∼ **14**) with seven aromatic amines (**15** ∼**21**) under the palladium-catalyzed amination reaction conditions afforded twenty-eight coupled compounds (**22A**∼**22BB**). The nitro groups in compounds **22** were reduced to the corresponding amines **23**, and finally, the acryloyl group was introduced to the resulting amine groups producing the final compounds (**6A** ∼ **6BB**).

**Scheme 2.**
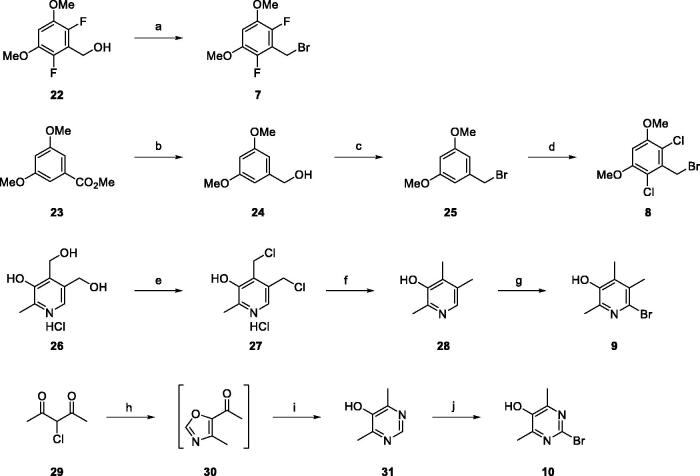
Preparation of the substrates (**7** ∼ **10**). *Reagents and Conditions*: (a) PBr_3_, CH_2_Cl_2_, r.t., 15 min, quant.; (b) LiAlH_4_, THF, −20 °C to r.t., 12 h, 97%; (c) PBr_3_, CH_2_Cl_2_, r.t., 15 min; (d) SO_2_Cl_2_, CH_3_CN-CH_2_Cl_2_, 0 °C to r.t., 42 h, 90% (for 2 steps); (e) SOCl_2_, DMF, ClCH_2_CH_2_Cl, reflux, 5 h, 93%; (f) Zn, AcOH, reflux, 3 h, 80%; (g) DBDMH, THF, r.t., 3 h, 80%; (h) HCO_2_NH_2_, HCO_2_H, 140 °C, 12 h; (i) NH_4_OH, 120 °C, 5 h, 34% (for 2 steps); (j) DBDMH, THF, r.t., 5 h, 63%.

Preparation of the required substrates **7** ∼ **10** is shown in Scheme 2. First, the fluoro-containing benzyl bromide **7** was readily prepared by a simple bromination from commercially available alcohol **22**^21^. For the synthesis of the chloro-version substrate **8**, we put the ring-chlorination step at the final stage. Reduction of methyl 3,5-dimethoxybenzoate (**23**) to primary alcohol **24** followed by bromination afforded the corresponding bromide **25**^32^. Lastly, chlorination of the ring with sulphuryl chloride gave the chloro-substrate **8**. The centred ring, 2,4,5-trimethylpyridin-3-ol (**9**), was prepared from pyridoxine (**26**) by the known procedure developed by us^33^. The two hydroxymethyl groups of **26** were chlorinated to give **27** which was then reductively cleaved to afford methyl compound **28**. Ring bromination of **28** finally gave the substrate **9**. Another centred ring, 2-bromo-4,6-dimethylpyrimidin-5-ol (**10**), was obtained by a three-step sequence. 3-Chloropentane-2,4-dione (**29**) was reacted with formamide in formic acid to give an oxazole intermediate **30** which was then treated with ammonia water to afford 4,6-dimethylpyrimidin-5-ol (**31**)^34^. The pyrimidine substrate **10** was finally obtained by bromination of **31**.

**Scheme 3.**
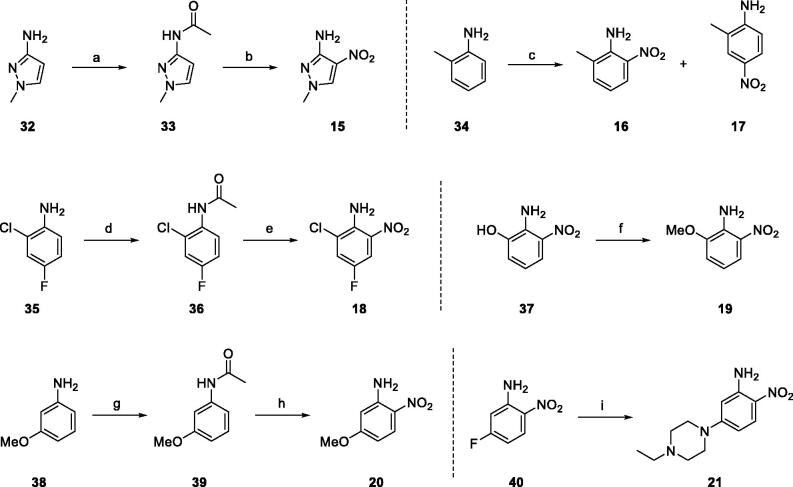
Preparation of the nitroamine compounds (**1 5** ∼**21**). *Reagents and Conditions*: (a) AcCl, THF, 0 °C to r.t., 2 h, quant.; (b) HNO_3_, H_2_SO_4_, 0 °C to r.t., 15 h, 51%; (c) i) Ac_2_O, 0 °C, 2 h, ii) HNO_3_, H_2_SO_4_, AcOH, 0 °C, 3 h then r.t., 12 h, iii) HCl, H_2_O, reflux, 5 h, **16** (35%), **17** (23%); (d) AcCl, THF, 0 °C to r.t., 2 h, quant.; (e) i) HNO_3_, H_2_SO_4_, AcOH, 0 °C, 3 h then r.t., 12 h, ii) HCl, H_2_O, reflux, 5 h, 59%; (f) MeI, K_2_CO_3_, acetone, r.t., 20 h, 95%; (g) AcCl, THF, 0 °C to r.t., 0.5 h, quant.; (h) i) HNO_3_, H_2_SO_4_, AcOH, 0 °C, 1.5 h then r.t., 2 d, ii) HCl, H_2_O, reflux, 3 h, 13%; (i) 1-ethylpiperazine, *^i^*Pr_2_NEt, NMP, 130 °C, 5 h, 98%.

Scheme 3 shows the preparation of substrates (**15** ∼ **21**) for the third part constituting the target compounds. They were synthesised from commercially available materials (**32**, **34**, **35**, **37**, **38**, and **40**) by a one- or three-step procedure. The compounds **15**, **16**, **17**, **18**, and **20** were prepared by a similar synthetic sequence, i.e., *N*-acetylation, nitration, and *N*-deacetylation^21^^,^^23^^,^^35^^,^^36^. For compound **19**, methylation of 2-amino-3-nitrophenol (**37**) was used, and replacement of fluoro of 5-fluoro-2-nitroaniline (**40**) with 1-ethylpiperazine afforded compound **21**, where the two materials, **37** and **40**, were purchasable.

### 2.2. Inhibitory activity of new compounds against FGFR kinases

**Figure 3.**
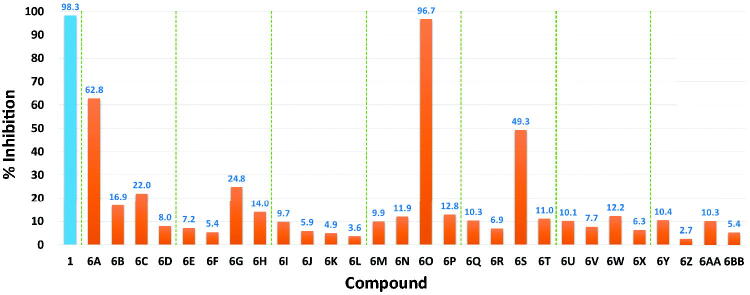
Inhibitory activities of the new compounds (**6A**∼**6BB**) along with BLU9931 (**1**) as the positive control at a fixed concentration (1 μM) against FGFR4 kinase. The numbers in the bar-graphs are mean values of % inhibition obtained from two independent experiments performed by the Reaction Biology Corp. (Malvern, PA, USA).

The new compounds (**6A**∼**6BB**) were subjected to cell-free kinase assay to examine their inhibitory activities against FGFR4 kinase at a fixed concentration (1 µM) along with BLU9931 (**1**), the positive control. Most compounds generally showed low inhibitory activity against FGFR4 (Figure 3). Roughly, the inhibitory activity of the difluoro-containing compounds was better than the corresponding dichloro analgoues. The inhibitory activity of compounds **6A**, **6O**, and **6S** was notable, and, in particular, compound **6O** showed an excellent inhibition comparable to that of BLU9931 at the 1 µM concentration tested.

A follow-up kinase assay of selected compounds, **6A** and **6O**, with 7 different concentrations, showed inhibition of the target kinase FGFR4 with IC_50_ values of 190 nM and 75.3 nM, respectively (Table 1). Compound **6A** inhibited FGFR1 ∼ 3 with IC_50_ 1565, 1149, and 277 nM, respectively, which corresponds to 1.5 ∼ 8 fold selectivity of FGFR4 over FGFR1 ∼ 3. Surprisingly, compound **6O** showed a marked FGFR4 selectivity of 398 ∼ 664 fold over FGFR1 ∼ 3 IC_50_ values of >50,000, 35,482, and >30,000 nM. This result indicates that compound **6O** has at least 8 times better FGFR4 selectivity than BLU9931 (**1**) (Table 1).

**Table 1.** IC_50_ values of the selected compounds against FGFR1 ∼ 4 kinases and selectivity.

Compound	IC_50_ (nM)^a^ / Ratio against FGFR4^b^			
	FGFR4	FGFR1	FGFR2	FGFR3
**1**	3.6	582.3/164.0	483.7/136.2	169.1/47.6
**6A**	190.0	1565.4/8.2	1149.4/6.0	277.3/1.5
**6O**	75.3	>50000/>663.8	35482.8/471.1	>30000/>398.3

^a^The IC_50_ values were obtained from three independent experiments.

^b^The ratio of FGFR1 ∼ 3 to FGFR4 inhibitory activity.

### 2.3. Structure-activity relationship (SAR) based on molecular docking

As shown in Figure 3, most of the trimethylpyridine- or dimethylpyrimidine-containing new compounds generally have low inhibitory activity against FGFR4 except for compounds **6A**, **6O**, and **6S**, which have fluorine in common at X position in Figure 2. We thought that a combination of fluorine substitution at the dimethoxyphenyl ring and the introduction of methyl groups to the pyridine or pyrimidine ring may contribute to the higher FGFR4 activity and selectivity of **6A** and **6O** as shown in Table 1. To clarify these substituent effects, we focussed on compounds **6E** and **6G**. These compounds are methylated analogs of known FGFR4 inhibitors **41** and **42**, which have bare pyridine and pyrimidine at the centre^21^. The FGFR4 percent inhibition data at 0.01 µM of these compounds are shown in Table 2. It is clear that the substitution of methyl groups at the pyridine and pyrimidine rings is detrimental to the activity. In order to further investigate the substitution effects of fluorines and methyl groups on the FGFR4 activity and selectivity, we performed covalent docking studies on relevant compounds.

**Table 2.** Percentages of inhibition of compounds at 0.01 μM concentration against FGFR4 kinase.

Structure	Compound	G	% Inhibition^a^
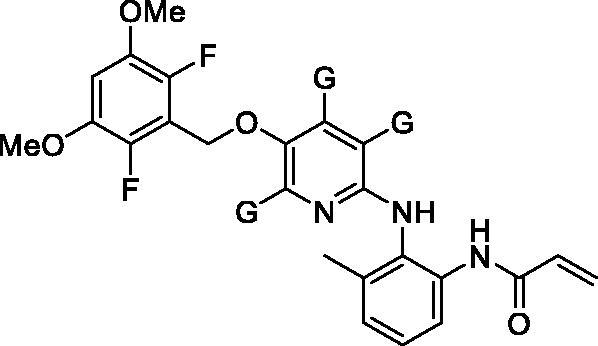	6E	Me	5.0
	41	H	91.0
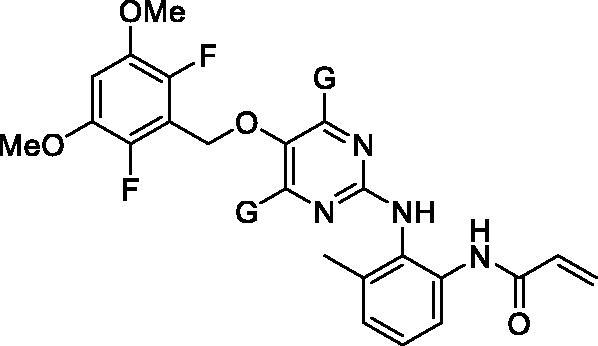	6G	Me	3.3
	42	H	87.4

^a^% Inhibition values were obtained from two independent experiments performed by the Reaction Biology Corp. (Malvern, PA, USA).

#### 2.3.1. Effect of methyl groups and halogen substituents on SAR

**Figure 4.**
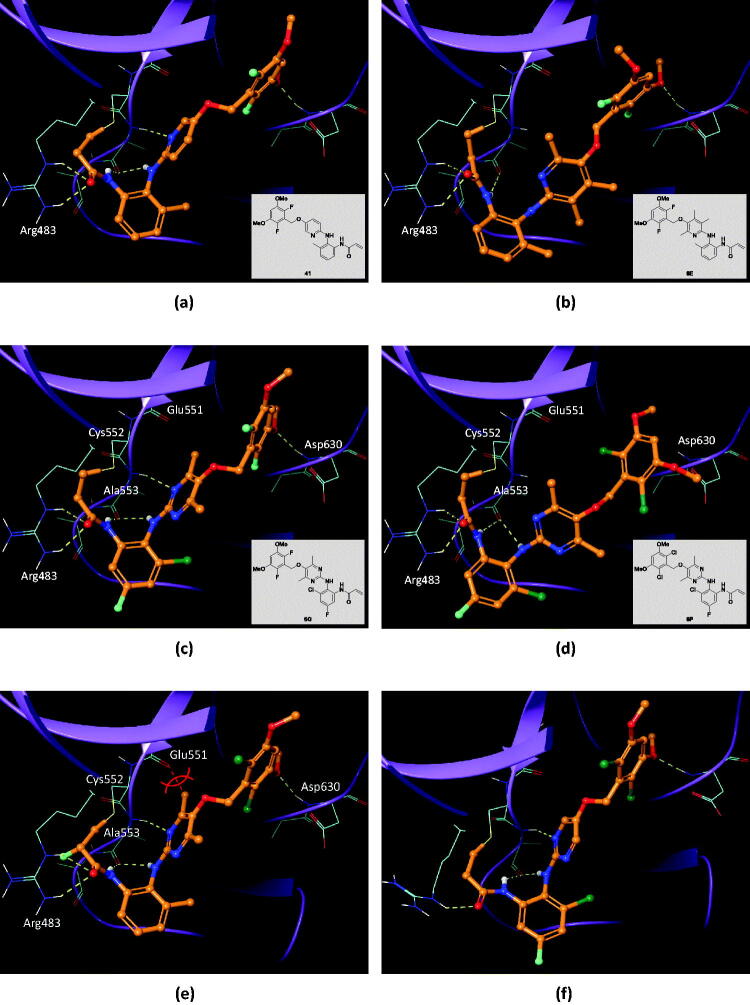
Effect of methyl groups and halogen substituents on binding to FGFR4. (a) Binding pose of FGFR4−**41** complex (LF VSscore= −11.88). (b) Binding pose of FGFR4−**6E** complex (LF VSscore= −10.97). Hydrogen bonds to Ala553 are hindered by the methyl groups of the pyridine ring. (c) Binding pose of FGFR4−**6O** complex (LF VSscore= −12.23). (d) Binding pose of FGFR4−**6P** complex (LF VSscore= −10.81). Hydrogen bonds to Ala553 are lost. (e) Introduction of methyl groups into the pyrimidine ring in FGFR4 − ligand complex crystal structure (PDB 7DTZ). Methyl groups in the pyrimidine ring would bump into the Glu551 peptide bond, forcing the ring plane to twist. (f) X-ray crystal structure of FGFR4−**6P-1** complex (PDB 6NVI).

Covalent docking studies were carried out using Flare from Cresset according to the methods described in detail in the experimental section. The LF Rank score and LF VSscore were used to identify correct binding pose and rank compounds in a virtual screening context, respectively. Introducing three methyl groups into the pyridine core (compound **41** → **6E**) cost LF VSscore of 0.908. As shown in Figure 4(a,b), this is a direct result of losing hydrogen bonds from Ala553 to the pyridine N and anilyl NH. Due to steric bulkiness of three methyl groups in compound **6E**, the pyridine ring plane is twisted by 90° and thus the hydrogen bonds are destroyed. On the other hand, introducing only two methyl groups into the pyrimidine ring is generally tolerated (compound **6O**). Unlike compound **6E**, it does not cause a twist of the pyrimidine ring plane (Figure 4(c)). However, replacing the fluorine atoms of the dimethoxyphenyl ring with chlorine (**6O** → **6P**) abolished the FGFR4 inhibitory activity (Figure 4(d)). To find out the origin of the activity change, we replaced hydrogens of the pyrimidine ring with methyl in the crystal structure of FGFR4 − ligand complex (PDB 7DTZ) and examined the binding interactions (Figure 4(e)). It turned out that the methyl group would clash with the peptide carbonyl oxygen of Glu551 and thus the ideal binding pose cannot be maintained as shown in Figure 4(d). LF VSscore also changed from −12.23 to −10.81 in agreement with the observed FGFR4 activities of **6O** and **6P**. Interestingly, removing methyl groups from the pyrimidine ring of **6P** restores the FGFR4 activity, i.e., it is known that compound **6P-1** exhibits an FGFR4 activity of IC_50_ 2.6 nM^37^. Figure 4(f) shows the X-ray co-crystal structure of FGFR4−**6P-1** complex (PDB 6NVI), in which, unlike **6P** (Figure 4(d)), one of the pyrimidine nitrogen atoms forms a hydrogen bond to Ala553. Therefore it can be concluded that both the small size of fluorine at the dimethoxyphenyl ring and the bulkiness of methyl groups at the pyrimidine ring amount to the observed FGFR4 activity and selectivity of compound **6O**.

#### 2.3.2. Selective inhibition of FGFR4 by compound 6O over FGFR1 − 3

**Figure 5.**
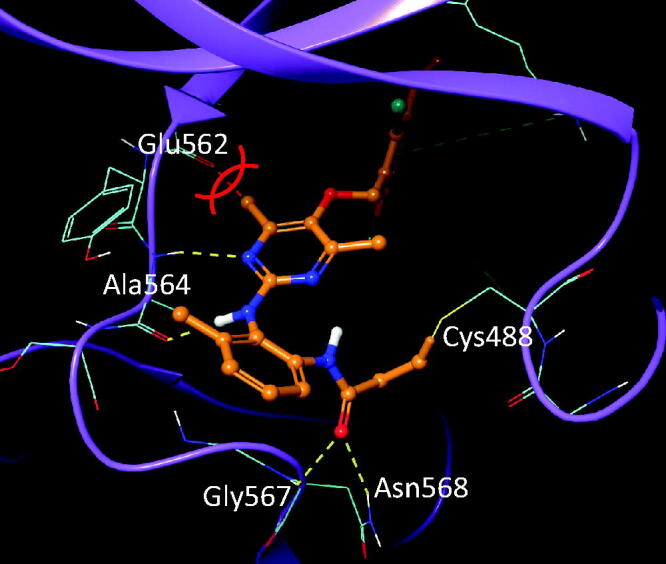
Introduction of methyl groups into the pyrimidyl ring in FGFR1 − ligand complex crystal structure (PDB 6NVL). Methyl groups in the pyrimidine ring would clash with the Glu562 on the hinge loop of FGFR1.

Compound **6O** showed an excellent selectivity towards FGFR4 over other FGFR subtypes (FGFR1 ∼ 3), whereas compound **6A** had a marginal selectivity (Table 1). Although detailed explanation for the selectivity profile of BLU554 for the hinge cysteine of FGFR4 has been reported recently^37^, more rationales are needed to address the exceptionally high selectivity profile of compound **6O**. We further conducted a molecular docking study to provide an answer to such a high degree of selectivity by **6O**. FGFR4 has a cysteine residue available for covalent binding of ligand in the hinge region, while other FGFR subtypes have one on the P-loop. X-ray crystal structures are found for FGFR − ligand complexes with a covalent bond at both the hinge and P-loop cysteine residues. It was proposed that the FGFR selectivity of BLU554 mainly comes from the rotational energy barriers of the ligand in solution and steric clash between the tetrahydropyran ring and the P-loop of FGFR1, specifically Leu484^37^. Although compound **6O** does not have a tetrahydropyran-like fragment that would clash with the P-loop of FGFR1, the methyl groups in the central pyrimidine ring would be too close to Glu562 at the hinge, hindering the binding (Figure 5). Therefore we propose that the outstanding FGFR4 selectivity of **6O** originates from the steric clash of the pyrimidine methyl groups to the hinge loop.

### 2.4. Anti-hepatocellular carcinoma efficacy evaluation

**Figure 6.**
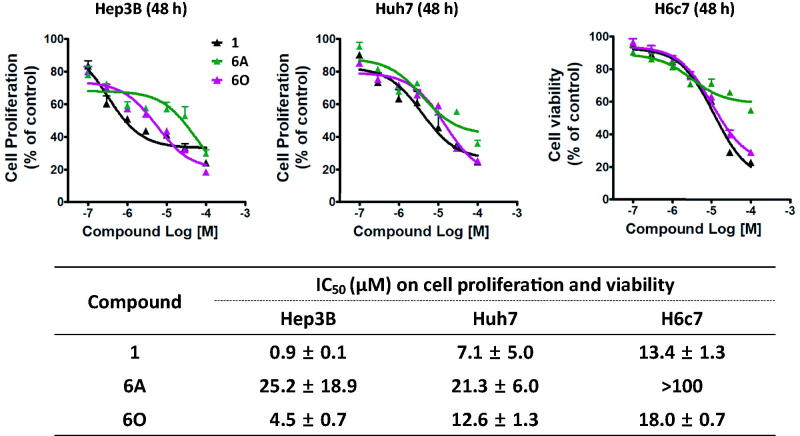
IC_50_ values of the selected compounds on proliferation of human liver cancer cell lines (Hep3B and Huh7) and viability of normal human pancreatic duct epithelial cell line (H6c7). Drugs were treated for 48 h. The values are mean ± SEM of three independent experiments performed in triplicate.

To examine anti-proliferative activities of compounds **6A** and **6O**, two HCC cell lines, Hep3B and Huh7 cells, were treated for 48 h with increasing concentrations, and their growth was measured by MTT assay. Hep3B and Huh7 cell lines overexpress FGF19 as a result of *FGF19* gene amplification and are suitable model cells to study FGF19–FGFR4 signalling^38–40^. Treatment of Hep3B cells with compounds **6A** and **6O** resulted in IC_50_ values of 25.2 and 4.5 µM, respectively, which was 28 and 5-fold less potent than compound **1** (Figure 6). The anti-proliferative activity of the positive control compound **1** in Huh7 cells was less effective than in Hep3B, showing a 7-fold difference. Compound **6O** showed a 2.8-fold difference of anti-proliferative activity in the two cell lines, whereas compounds **6A** did not show a significant difference between the two cell lines. IC_50_ values of cytotoxicity of the compounds **1**, **6A**, and **6O** measured in H6c7 normal human pancreatic duct epithelial cell line were 13.4, >100, and 18 µM, respectively, indicating compound **6A** was fairly safe, and cytotoxicity of **6O** and **1** was similar.

**Figure 7.**
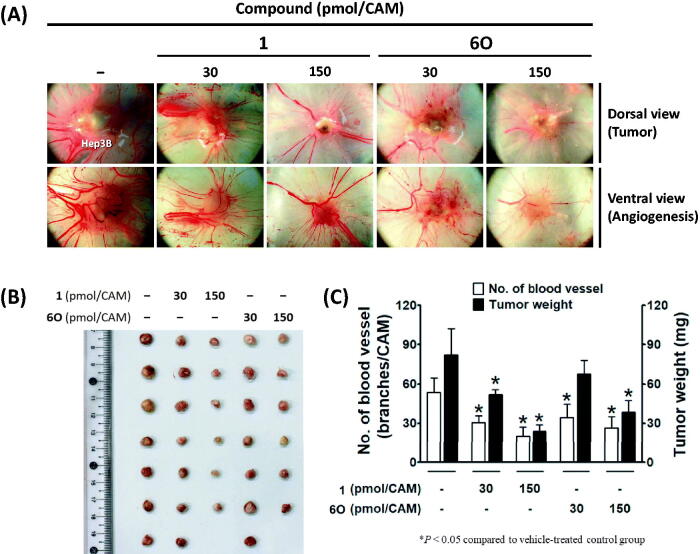
Inhibitory effects of compound **6O** and BLU9931 (**1**) on tumour growth and tumour-induced angiogenesis in Hep3B-xenografted CAM tumour model. Four days after Hep3B xenograft, both tumour growth and tumour-induced angiogenesis on the CAM tissues are shown in (A). The weight of tumour masses isolated from CAMs (B) and the number of new vessel branches formed on CAM were counted using Image J program (C). *P< 0.05 compared to the vehicle-treated group.

Next, we examined the *in vivo* antitumor efficacy of compound **6O** using a chick chorioallantoic membrane (CAM) tumour model implanted with Hep3B cells. The implanted Hep3B cells in the vehicle-treated control group developed a tumour mass and tumour-induced angiogenesis (Figure 7). Compound **6O** and positive control compound (BLU9931, **1**) dose-dependently inhibited the tumour growth. The inhibitory effect of **6O** on the tumour weight was slightly weaker than that of compound **1**. The tumour-induced angiogenesis was also significantly blocked by compounds, **6O** and **1**, and the antiangiogenic effect of compound **6O** was also slightly weaker than compound **1**.

## 3. Conclusion

A series of novel compounds containing aminotrimethylpyri(mi)dinol were designed and synthesised with the aim of the discovery of selective FGFR4 inhibitors. Among them, compound **6O** showed a potent and highly selective inhibitory activity against FGFR4 over FGFR1 ∼ 3. Structure-activity relationship study using intensive molecular docking calculations revealed that, by and large, introducing methyl groups at the central pyridine or pyrimidine ring can cause a steric clash with the hinge of FGFR4 kinase domain and thus weaken the binding affinity. However, exceptionally, compound **6O** with dimethylpyrimidine core ring and difluoro substituent was observed to maintain its conformation suitable for strong binding interaction. Molecular docking study also suggests that a steric clash of dimethyl groups of compound **6O** to a hinge of FGFR1 ∼ 3 interfere with adopting a proper pose for a strong binding between them. This explains the highly selective inhibition of compound **6O** against FGFR4 over other families. The anti-proliferative activity of compound **6O** on HCC cell lines was a little weaker than BLU9931 at low concentrations, however, the strength of the inhibitory effect was reversed at high concentrations. In addition, compound **6O** effectively inhibited both the growth and angiogenesis of HCC, one of the hypervascular tumours. Overall, the antitumor effect of compound **6O** was excellent and comparable to that of BLU9931.

## 4. Experimental section

### 4.1. Synthesis (Supplemental data)

#### 4.1.1. General

Unless noted otherwise, materials were purchased from commercial suppliers and used without further purification. Air or moisture-sensitive reactions were carried out under argon atmosphere. The reaction progress was monitored by thin layer-chromatography (TLC) using silica gel F_254_ plates. Products were purified by flash column chromatography using silica gel 60 (70–230 mesh) or by using the Biotage ‘Isolera One’ system with indicated solvents. Melting points were determined using a Fisher–Johns melting point apparatus and were not corrected. Low-resolution mass spectra (LRMS) were obtained using a JMS-700 (JEOL) and recorded either in molecular ion peak mode with an electron ionisation (EI) source or in positive ion mode with fast electron bombardment (FAB) source or using a Waters ZQ 2000 and recorded in a positive ion mode with an electrospray (ESI) source. High-resolution mass spectra (HRMS) were obtained using a JMS-700 (JEOL) and recorded either in molecular ion peak mode with an electron ionisation (EI) source or in positive ion mode with fast electron bombardment (FAB) source. NMR spectra were obtained using a Bruker-250 spectrometer (250 MHz for ^1^H NMR and 62.5 MHz for ^13^C NMR) and a Bruker Avance Neo 400 spectrometer (400 MHz for ^1^H NMR). Chemical shifts (δ) were expressed in ppm using a solvent as an internal standard and the coupling constant (J) in hertz.

#### 4.1.2. 3-(Bromomethyl)-2,4-difluoro-1,5-dimethoxybenzene (7) [CAS RN: 1956324–83-6]

To a solution of (2,6-difluoro-3,5-dimethoxyphenyl)methanol (**22**) (3.0 g, 14.69 mmol) in DCM (15 ml) was added a solution of PBr_3_ (1 M in DCM, 15.7 ml, 15.72 mmol) dropwise at 0 °C then stirred at room temperature for 30 min. The reaction mixture was quenched with ice water followed by DCM extraction. The combined organic solution was dried over MgSO_4_, filtered, concentrated to obtain **7** (3.75 g, 100%) as a grey solid.

#### 4.1.3. 4,6-Dimethylpyrimidin-5-ol (31) [CAS RN: 70345–38-9]

To a solution of 3-chloro-2,4-pentadione (**29**) (20 ml, 177.17 mmol) in formic acid (30 ml) was added formamide (16 ml, 402.84 mmol) and refluxed for 12 h. The reaction mixture was cooled to room temperature then aq. NH_4_OH was added dropwise until the basic pH was maintained. After refluxing the mixture for 5 h, it was cooled to room temperature and then concentrated. Acetone was added to the residue to acquire product in acetone. The acetone fraction was concentrated, and the residue was purified by silica gel column chromatography with EtOAc to obtain nd **31** (7.4 g, 34%) as a pale yellow solid.

#### 4.1.4. 2-Bromo-4,6-dimethylpyrimidin-5-ol (10)

To a solution of 4,6-dimethylpyrimidin-5-ol (**31**) (150 mg, 1.21 mmol) in THF (4 ml) was added DBDMH (242 mg, 0.85 mmol). The resulting mixture was stirred at room temperature for 5 h and then concentrated. The residue was diluted with EtOAc and water, and the aqueous layer was extracted with EtOAc. The combined organic solution was dried over MgSO_4_, filtered, and concentrated. The residue was purified by silica gel column chromatography with 10% to 40% EtOAc/Hexanes to obtain **10** (156 mg, 63%). Brown solid; TLC *R_f_* 0.55 (EtOAc/Hexanes = 1/1); m.p. 170 °C; ^1^H NMR (CDCl_3_) δ 2.47 (s, 6H); ^13^C NMR (CDCl_3_) δ 156.4 (2C), 147.2, 141.3, 18.7 (2C); HRMS (FAB) *m*/*z* calculated for C_6_H_8_BrN_2_O [M + H]^+^ 201.9742, found 201.9739.

#### 4.1.5. 2-Bromo-5-((2,6-difluoro-3,5-dimethoxybenzyl)oxy)-4,6-dimethylpyrimidine (13)

To a solution of 2-bromo-4,6-dimethylpyrimidin-5-ol (**10**) (90 mg, 0.44 mmol) in DMF (3 ml) were added 3-(bromomethyl)-2,4-difluoro-1,5-dimethoxybenzene (**7**) (94 mg, 0.35 mmol) and K_2_CO_3_ (304 mg, 2.20 mmol). The mixture was stirred at 80 °C for 12 h and then cooled to room temperature. It was quenched with ice water and stirred at 0 °C for 15 min. The precipitate formed in the mixture was collected by filtration and washed with ice water to obtain **13** (114 mg, 67%). White solid; TLC *R_f_* 0.63 (EtOAc/Hexanes = 1/2); m.p. 145 °C; ^1^H NMR (CDCl_3_) δ 6.70 (t, *J*= 8.2 Hz, 1H), 4.96 (t, *J*= 1.8 Hz, 2H), 3.89 (s, 6H), 2.47 (s, 6H); ^13 ^C NMR (CDCl_3_) δ 164.2 (2C), 149.5, 147.0 (d, *J*= 5.7 Hz), 145.7, 143.9 (d, *J*= 5.0 Hz), 143.7 (d, *J*= 5.0 Hz), 143.1 (d, *J*= 5.6 Hz), 113.1, 102.4, 62.5, 57.5 (2C), 18.9 (2C); HRMS (EI) *m*/*z* calculated for C_15_H_15_BrF_2_N_2_O_3_ [M]^+^ 388.0234, found 388.0239.

#### 4.1.6. N-(2-Chloro-4-fluorophenyl)acetamide (36) [CAS RN: 399–35-9]

To a solution of 2-chloro-4-fluoroaniline (**35**) (5.0 ml, 41.87 mmol) in THF (10 ml) was added acetyl chloride (5.9 ml, 83.75 mmol) at 0 °C. The mixture was stirred at room temperature for 5 h. The precipitate formed in the reaction mixture was collected to obtain **36** (8.2 g) as a white solid then used in the next step reaction without further purification.

#### 4.1.7. 2-Chloro-4-fluoro-6-nitroaniline (18) [CAS RN: 153505–32-9]

To a mixture of *N*-(2-chloro-4-fluorophenyl)acetamide (**36**) (1.0 g, 5.33 mmol) in acetic acid (2 ml) and concentrated H_2_SO_4_ (10 ml, 187.60 mmol) was added fuming HNO_3_ (334 µL, 8.0 mmol) dropwise at 0 °C. The mixture was stirred at 0 °C for 3 h and then it was poured into ice water and stirred at room temperature for 12 h. The mixture was neutralised using a solution of 6 M NaOH followed by EtOAc extraction. The combined organic solution was dried over MgSO_4_, filtered, and concentrated. After addition of an excessive amount of 1 M HCl, the mixture was refluxed for 5 h and then neutralised using a solution of 6 M NaOH followed by EtOAc extraction. The combined organic solution was dried over MgSO_4_, filtered, and concentrated. The residue was purified by silica gel column chromatography with 1% to 5% EtOAc/Hexanes to obtain **18** (596 mg, 59%) as a yellow solid.

#### 4.1.8. N-(2-Chloro-4-fluoro-6-nitrophenyl)-5-((2,6-difluoro-3,5-dimethoxybenzyl)oxy)-4,6-dimethylpyrimidin-2-amine (22 O)

To a mixture of 2-bromo-5-((2,6-difluoro-3,5-dimethoxybenzyl)oxy)-4,6-dimethylpyrimidine (**13**) (150 mg, 0.39 mmol) and 2-chloro-4-fluoro-6-nitroaniline (**18**) (110 mg, 0.58 mmol) in toluene (3 ml) were added Cs_2_CO_3_ (376 mg, 1.16 mmol), Xantphos (67 mg, 0.12 mmol), and Pd_2_(dba)_3_ (40 mg, 0.04 mmol). The mixture was refluxed for 20 h. and then diluted with DCM and washed with brine. The organic layer was dried over MgSO_4_, filtered, and concentrated. The residue was purified by silica gel column chromatography with 10% to 20% EtOAc/Hexanes to obtain **22O** (136 mg, 71%). Yellow solid; TLC *R_f_* 0.32 (EtOAc/Hexanes = 1/2); m.p. 170 °C; ^1^H NMR (CDCl_3_) δ 7.64 (dd, *J*= 7.9, 3.0 Hz, 1H), 7.45 (dd, *J*= 7.3, 3.0 Hz, 1H), 7.32 (s, 1H), 6.69 (t, *J*= 8.2 Hz, 1H), 4.86 (t, *J*= 1.8 Hz, 2H), 3.89 (s, 6H), 2.35 (s, 6H); ^13^C NMR (CDCl_3_) δ 161.8, 158.9, 154.9, 153.5, 147.2 (d, *J*= 5.6 Hz), 144.8, 144.5 (d, *J*= 8.8 Hz), 143.8 (d, *J*= 5.0 Hz), 143.7 (d, *J*= 4.9 Hz), 143.3 (d, *J*= 5.8 Hz), 130.7 (d, *J*= 10.0 Hz), 128.0 (d, *J*= 4.0 Hz), 121.6 (d, *J*= 25.3 Hz), 113.7 (t, *J*= 17.9 Hz), 111.8 (d, *J*= 26.8 Hz), 102.4 (t, *J*= 2.3 Hz), 62.5 (t, *J*= 3.6 Hz), 57.6 (2C), 18.6 (2C); HRMS (EI) *m*/*z* calculated for C_21_H_18_ClF_3_N_4_O_5_ [M]^+^ 498.0918, found 498.0914.

#### 4.1.9. 6-Chloro-N^1^-(5-((2,6-difluoro-3,5-dimethoxybenzyl)oxy)-4,6-dimethylpyrimidin-2-yl)-4-fluorobenzene-1,2-diamine (23 O)

To a mixture of *N*-(2-chloro-4-fluoro-6-nitrophenyl)-5-((2,6-difluoro-3,5-dimethoxybenzyl)oxy)-4,6-dimethylpyrimidin-2-amine (**22O**) (120 mg, 0.24 mmol) in EtOH (3 ml) was added SnCl_2_·2H_2_O (271 mg, 1.20 mmol). The reaction mixture was refluxed for 12 h and then concentrated. The residue was diluted with EtOAc and saturated K_2_CO_3_ followed by EtOAc extraction. The combined organic solution was dried over MgSO_4_, filtered, and concentrated. The residual solid was washed with EtOAc/Hexanes to obtain **23O** (101 mg, 89%). Grey solid; TLC *R_f_* 0.48 (DCM/MeOH = 20/1); m.p. 260 °C; ^1^H NMR (DMSO-*d*_6_) δ 8.04 (s, 1H), 7.07 (t, *J*= 7.9 Hz, 1H), 6.61–6.36 (m, 2H), 5.37 (s, 2H), 4.81 (s, 2H), 3.87 (s, 6H), 2.18 (s, 6H); ^13^C NMR (DMSO-*d*_6_) δ 162.4, 160.2, 158.5, 157.0, 148.8 (d, *J*= 13.5 Hz), 145.6 (d, *J*= 6.0 Hz), 143.4 (d, *J*= 4.6 Hz), 143.2 (d, *J*= 4.2 Hz), 142.0, 141.7, 134.0 (d, *J*= 14.6 Hz), 118.7, 113.3 (t, *J*= 18.3 Hz), 102.7 (d, *J*= 26.2 Hz), 101.7, 99.3 (d, *J*= 25.0 Hz), 61.9, 56.9 (2C), 18.3 (2C); HRMS (EI) m/z calculated for C_21_H_20_ClF_3_N_4_O_3_ [M]^+^ 468.1176, found 468.1177.

#### 4.1.10. N-(3-Chloro-2-((5-((2,6-difluoro-3,5-dimethoxybenzyl)oxy)-4,6-dimethylpyrimidin-2-yl)amino)-5-fluorophenyl)acrylamide (6 O)

To a solution of 6-chloro-*N^1^*-(5-((2,6-difluoro-3,5-dimethoxybenzyl)oxy)-4,6-dimethylpyrimidin-2-yl)-4-fluorobenzene-1,2-diamine (**23O**) (30 mg, 0.06 mmol) in DCM (2 ml) were added triethylamine (27 µL, 0.19 mmol) and acryloyl chloride (7.8 µL, 0.10 mmol) dropwise at 0 °C. The mixture was stirred at room temperature for 12 h and then diluted with DCM and saturated NaHCO_3_ followed by DCM extraction. The combined organic solution was dried over MgSO_4_, filtered, and concentrated. The residue was purified by silica gel column chromatography with 30% to 40% EtOAc/Hexanes to obtain **6O** (7 mg, 20%). White solid; TLC *R_f_* 0.35 (EtOAc/Hexanes = 1/1); m.p. 160 °C; ^1^H NMR (CDCl_3_) δ 9.12 (s, 1H), 8.01 (dd, *J*= 10.5, 2.7 Hz, 1H), 6.98 (dd, *J*= 7.7, 2.9 Hz, 1H), 6.70 (t, *J*= 8.2 Hz, 2H), 6.32 (dd, *J*= 16.9, 1.1 Hz, 1H), 6.11 (dd, *J*= 16.9, 10.1 Hz, 1H), 5.71 (dd, *J*= 10.1, 1.1 Hz, 1H), 4.90 (t, *J*= 1.7 Hz, 2H), 3.89 (s, 6H), 2.38 (s, 6H); ^13^C NMR (CDCl_3_) δ 163.7, 162.4, 162.2, 158.3, 156.4, 147.1 (d, *J*= 5.8 Hz), 144.2, 143.9 (d, *J*= 5.0 Hz), 143.7 (d, *J*= 5.0 Hz), 143.2 (d, *J*= 5.7 Hz), 136.8 (d, *J*= 12.9 Hz), 131.6, 131.1 (d, *J*= 12.7 Hz), 127.9, 124.3 (d, *J*= 3.4aHz), 113.6 (t, *J*= 17.8 Hz), 112.4 (d, *J*= 26.3 Hz), 109.4 (d, *J*= 27.1 Hz), 102.3 (t, *J*= 2.5 Hz), 62.6 (t, *J*= 3.6 Hz), 57.5 (2C), 18.9 (2C); HRMS (EI) *m*/*z* calculated for C_24_H_22_ClF_3_N_4_O_4_ [M]^+^ 522.1282, found 522.1285.

### 4.2. Biological evaluation

#### 4.2.1. FGFR kinase assay

A FGFR4 kinase assay was performed at the Reaction Biology Corporation (Malvern, PA, USA) using a Kinase HotSpotSM assay platform (www.reactionbiology.com, last accessed at 19/10/2020). Briefly, human FGFR4 kinase (5–10 mU), peptide substrate, and poly[Glu:Tyr] (4:1) 0.2 mg/ml were prepared in a reaction buffer with a final volume of 25 µL. The compounds were delivered into the reaction, followed ∼20 min. later by the addition of a mixture of ATP and [γ-^33^P-ATP] (specific activity approx. 500 cpm/pmol, concentration as required) to a final concentration of 10 µM. After incubation for 40 min. at 25 °C, the reaction was stopped by the addition of a 3% phosphoric acid solution. Then, the reaction was spotted onto a P30 filtermat, and unbound phosphate was removed by washing 3 times for 5 min in 75 mM phosphoric acid and once in methanol prior to drying and scintillation counting. The background counting derived from the control reactions containing the inactive enzyme was subtracted, and specific kinase activity data were expressed as the percentage of remaining kinase activity in the test compounds compared to the vehicle (dimethyl sulfoxide) reactions. IC_50_ values and curve fits were obtained using GraphPad Prism 5 software.

FGFR1 ∼ 3 kinase assays were performed using the adenosine diphosphate (ADP)-glow kinase assay kit of FGFR1, FGFR2, and FGFR3 kinase enzyme systems (Promega, WI, USA) in accordance with the manufacturer’s instructions. Kinase activity was detected by the addition 50 µM adenosine triphosphate (ATP) to a mixture of 0.2 µg/µL poly (Glu4, Tyr1), test drug (**1**, **6A**, **6O**), and corresponding enzyme, FGFR1 (1.5 ng/µL), FGFR2 (3 ng/µL), or FGFR3 (6 ng/µL). The reaction was carried out at 25 °C for 1 h in a total volume of 25 µL. Subsequently, 25 µL of ADP-Glow reagent was added to the mixture, and then incubated for 40 min at 25 °C. After the addition of 50 µL of ADP detection reagents for 30 min at 25 °C, luminescence was measured using a Fluostar Omega microplate reader (BMG LABTECH GmbH, Ortenberg, Germany).

#### 4.2.2. Cell lines and culture

Human HCC cell lines, Hep3B and Huh7 were obtained from American Type Culture Collection (ATCC, Manassas, VA, USA). An H6c7 human normal pancreatic duct epithelial cell line was purchased from Kerafast (Boston, MA, USA). Hep3B and Huh7 cells were cultured in Dulbecco′s Modified Eagle′s Medium (DMEM) (Hyclone, Logan, UT, USA). The media were supplemented with 10% foetal bovine serum (FBS) (Gibco/ThermoFisher Scientific) and 1% penicillin/streptomycin (Gibco/ThermoFisher Scientific). H6c7 cells were maintained in a keratinocyte serum-free medium supplemented with a recombinant endothelial growth factor (rEGF) and bovine pituitary extract (Gibco/ThermoFisher Scientific). All the cells were incubated at 37 °C under a 5% CO_2_ atmosphere.

#### 4.2.3. Cell proliferation assay

Cells were seeded at a density of 5000 cells/well in a 96-well plate. After overnight incubation, the cells were serum-starved using 1% FBS for 24 h. The next day, the cells were pre-treated with the different concentrations (0.1, 1, 3, 10, 30, 100 µM) of drugs for 1 h prior to the treatment with serum (10% FBS). After 48 h of incubation, 3–(4,5-dimethylthiazol-2-yl)-2,5-diphenyltetrazolium bromide (MTT) dye solution was added and incubated for 4 h. Next, DMSO was added, and after 30 min, the colour intensities were measured using a microplate reader (Versamax, Molecular Devices, Inc., USA) at 490 nm.

#### 4.2.4. Cytotoxicity assay

The cytotoxicity of the compounds was assessed by measuring the cell viability decrease using the MTT staining method. Briefly, H6c7 cells were seeded in a 96-well plate (Falcon, USA) at a density of 4 × 10^4^ cells per well in keratinocyte serum-free medium. After 24 h, Cells were incubated with different concentrations (0.1, 1, 3, 10, 30, and 100) µM of each compound for 48 h, and the cell viability was measured using MTT assay. Optical density was measured at 540 nm using a microplate reader (BMG LABTECH).

#### 4.2.5. Cam (chick chorioallantoic membrane) tumour model

Fertile chicken eggs were purchased from Byeolbichon Farm (Gyeongbuk, South Korea) and incubated at 37 °C and under 55% relative humidity. On the 9th day of egg incubation (post-fertilization), false air sac was generated on the relatively flat side of eggs by a negative pressure technique. A small window (1 cm^2^) was created on the false air sac surface of the eggs by separating the shell and membrane beneath (technique) using a grinding wheel (Dremel, Racine, WI, USA). Next, Hep3B cells were loaded at a density of 1.5 × 10^6^ cells/CAM with or without compound. After four days of drug treatment, the tumour weight, number of vessel branch points within the tumour region were analysed.

The chick embryo experiments were approved beforehand by the Institutional Animal Care and Use Committee of Yeungnam University and were performed accordingly the guidelines issued by the Institute of Laboratory Animal Resources (1996) and Yeungnam University (The care and use of animals 2009).

#### 4.2.6. Statistical analysis

The results are presented as the mean ± SEM and were analysed using one-way ANOVA followed by the Newman-Keuls comparison method using the GraphPad Prism software (version 5.0) (San Diego, CA, USA). P values less than 0.05 were considered statistically significant.

### 4.3. Computational study

Flare version 5.0 from Cresset (http://www.cresset-group.com/flare/) was used for covalent docking for this study. The crystal structure of the complex of FGFR4 and a 2-aminopyridine-based inhibitor (PDB ID 7DTZ) was chosen for docking owing to its high crystal resolution (2.01 Å) and the possession of strong hydrogen bonding interactions between the guanidyl group of Arg483 and the carbonyl oxygen of the acrylamide moiety of the ligand, which would help keep the ligands within the binding cavity in a proper orientation during the docking. Docking parameters were set up to use Cys552 as the covalent bonding residue and to use “Very Accurate but Slow” as the calculation method. For post-docking analysis, LF Rank Score was used to identify correct binding pose, while LF VSscore was used to rank compounds in a virtual screening context^41^.

Procedures for the synthesis of compound 6O are provided here. Synthetic procedures for all compounds are described in Supplementary Material.

## Supplementary Material

Supplemental Material
