# Supplementary material for: 6-Amino-2,4,5-trimethylpyridin-3-ol and 2-amino-4,6-dimethylpyrimidin-5-ol derivatives as selective fibroblast growth factor receptor 4 inhibitors: design, synthesis, molecular docking, and anti-hepatocellular carcinoma efficacy evaluation
Source: J Enzyme Inhib Med Chem. 2022 Mar 17;37(1):844–56. doi: 10.1080/14756366.2022.2048378 (PMC8933034; doi:10.1080/14756366.2022.2048378)
Supplement: Supplemental Material [file IENZ_A_2048378_SM0510.pdf]

Supplemental material

6-Amino-2,4,5-trimethylpyridin-3-ol and 2-amino-4,6-dimethylpyrimidin-5-ol derivatives as selective fibroblast growth factor receptor 4 inhibitors: Design, synthesis, molecular docking, and anti-hepatocellular carcinoma efficacy evaluation

Chhabi Lal Chaudhary[a], Dongchul Lim[b,*], Prakash Chaudhary[a], Diwakar Guragain[a], Bhuwan Prasad Awasthi[a], Hee Dong Park[b], Jung-Ae Kim[a,*], Byeong-Seon Jeong[a,*]

[a]*College of Pharmacy, Yeungnam University, 280 Daehak-ro, Gyeongsan 38541, Republic of Korea*

[b]*Innovo Therapeutics Inc., Daeduck biz center C-313, 17 Techno 4-ro, Yuseong-gu, Daejeon 34013, Republic of Korea*

Correspondence

- Dongchul Lim: dclim@innovothera.com
- Jung-Ae Kim: jakim@yu.ac.kr
- Byeong-Seon Jeong: jeongb@ynu.ac.kr

Contents

- Synthetic procedures.
- ^{1}H and ^{13}C NMR spectra for all new compounds.

Synthetic procedures

General

Unless noted otherwise, materials were purchased from commercial suppliers and used without further purification. Air or moisture-sensitive reactions were carried out under argon atmosphere. The reaction progress was monitored by thin layer-chromatography (TLC) using silica gel F_{254} plates. Products were purified by flash column chromatography using silica gel 60 (70–230 mesh) or by using the Biotage 'Isolera One' system with indicated solvents. Melting points were determined using a Fisher–Johns melting point apparatus and were not corrected. Low-resolution mass spectra (LRMS) were obtained using a JMS-700 (JEOL) and recorded either in molecular ion peak mode with an electron ionization (EI) source or in positive ion mode with fast electron bombardment (FAB) source or using a Waters ZQ 2000 and recorded in a positive ion mode with an electrospray (ESI) source. High-resolution mass spectra (HRMS) were obtained using a JMS-700 (JEOL) and recorded either in molecular ion peak mode with an electron ionization (EI) source or in positive ion mode with fast electron bombardment (FAB) source. NMR spectra were obtained using a Bruker-250 spectrometer (250 MHz for ^{1}H NMR and 62.5 MHz for ^{13}C NMR) and a Bruker Avance Neo 400 spectrometer (400 MHz for ^{1}H NMR). Chemical shifts (δ) were expressed in ppm using a solvent as an internal standard and the coupling constant (J) in hertz.

_N_-(3-((5-((2,6-Difluoro-3,5-dimethoxybenzyl)oxy)-3,4,6-trimethylpyridin-2-yl)amino)-1-methyl-1_H_-pyrazol-4-yl)acrylamide (6A)

To a solution of acrylic acid (5 µL, 0.08 mmol) in DMF (1 ml) were added propanephosphonic acid anhydride (T3P, 50% in EtOAc) (46 µL, 0.08 mmol) and Et_3N (20 µL, 0.14 mmol) then stirred at 100 °C for 3 h. To the resulting mixture was added **23A** (25.0 mg, 0.06 mmol) and stirred for 1 h. The reaction mixture was quenched with ice water then diluted with EtOAc. The organic layer was dried over $MgSO_4$, filtered, and concentrated. The residue was purified by flash silica gel column chromatography with 2% to 5% MeOH/DCM to obtain **6A** (13 mg, 48%). Brown solid; TLC R_f 0.37 (DCM/MeOH = 20/1); m.p. 135 °C; MS (EI) *m*/*z* 487 $[M]^+$; ^{1}H NMR ($CDCl_3$) δ 10.75 (s, 1H), 8.10 (s, 1H), 6.69 (t, *J* = 8.2 Hz, 1H), 6.35 (dd, *J* = 16.9, 2.2 Hz, 1H), 6.30–6.17 (m, 1H), 5.67 (dd, *J* = 9.5, 2.2 Hz, 1H), 4.83 (t, *J* = 1.7 Hz, 2H), 3.90 (s, 6H), 3.78 (s, 3H), 2.46 (s, 3H), 2.30 (s, 3H), 2.18 (s, 3H); ^{13}C NMR ($CDCl_3$) δ 162.3, 149.4, 147.3 (d, *J* = 5.8 Hz), 145.4 (2C), 143.8 (d, *J* = 5.0 Hz), 143.6 (d, *J* = 4.8 Hz), 143.4 (d, *J* = 5.9

Hz), 140.9, 131.5 (2C), 126.3, 125.3, 115.1, 114.1 (t, J = 18.0 Hz), 111.9, 102.1, 62.5, 57.5 (2C), 39.2, 18.7, 13.3, 13.0; HRMS (EI) *m/z* calculated for $C_{24}H_{27}F_2N_5O_4$ $[M]^+$ 487.2031, found 487.2033.

N-(3-((5-((2,6-Dichloro-3,5-dimethoxybenzyl)oxy)-3,4,6-trimethylpyridin-2-yl)amino)-1-methyl-1*H*-pyrazol-4-yl)acrylamide (6B)

To a solution of acrylic acid (9 µL, 0.13 mmol) in DMF (1 ml) were added propanephosphonic acid anhydride (T3P, 50% in EtOAc) (77 µL, 0.13 mmol) and Et_3N (27 µL, 0.19 mmol) then stirred at 100 °C for 3 h. To the resulting mixture was added **23B** (30 mg, 0.06 mmol) and stirred for 1 h. The reaction mixture was quenched with ice water then diluted with EtOAc. The organic layer was dried over $MgSO_4$, filtered, and concentrated. The residue was purified by flash silica gel column chromatography with 50% to 100% EtOAc/Hexanes to obtain **6B** (15 mg, 43%). White solid; TLC R_f 0.37 (EtOAc); m.p. 170 °C; MS (EI) *m/z* 519 $[M]^+$; ^{1}H NMR (DMSO-d_6) δ 9.73 (s, 1H), 8.00 (s, 1H), 7.83 (s, 1H), 6.98 (s, 1H), 6.32 (dd, J = 16.9, 10.1 Hz, 1H), 6.14 (dd, J = 17.0, 1.9 Hz, 1H), 5.66 (dd, J = 10.1, 1.9 Hz, 1H), 5.05 (s, 2H), 3.93 (s, 6H), 3.74 (s, 3H), 2.17 (s, 3H), 2.16 (s, 3H), 2.09 (s, 3H); ^{13}C NMR (DMSO-d_6) δ 161.4, 154.3 (2C), 144.8, 140.3, 140.2, 133.2, 131.4, 126.0, 123.9, 114.9 (2C), 112. 8, 98.9, 69.4, 56.8 (2C), 38.7, 13.2 (2C), 13.1; HRMS (EI) *m/z* calculated for $C_{24}H_{27}Cl_2N_5O_4$ $[M]^+$ 519.1440, found 519.1443.

N-(3-((5-((2,6-Difluoro-3,5-dimethoxybenzyl)oxy)-4,6-dimethylpyrimidin-2-yl)amino)-1-methyl-1*H*-pyrazol-4-yl)acrylamide (6C)

To a solution of acrylic acid (18 µL, 0.26 mmol) in DMF (1 ml) were added propanephosphonic acid anhydride (T3P, 50% in EtOAc) (155 µL, 0.26 mmol) and Et_3N (54 µL, 0.39 mmol) then stirred at 100 °C for 3 h. To the resulting mixture was added **23C** (55 mg, 0.13 mmol) and stirred for 15 minutes. The reaction mixture was quenched with ice water then diluted with EtOAc. The organic solution was dried over $MgSO_4$, filtered, and concentrated. The residue was purified by flash silica gel column chromatography with 50% to 100% EtOAc/Hexanes to obtain **6C** (38 mg, 61%). Pale yellow solid; TLC R_f 0.50 (DCM/MeOH = 20/1, developed twice); m.p. 232 °C; MS (EI) *m/z* 474 $[M]^+$; ^{1}H NMR (DMSO-d_6) δ 9.49 (s, 1H), 8.86 (s, 1H), 8.05 (s, 1H), 7.08 (t, J = 8.4 Hz, 1H), 6.44 (dd, J = 17.0, 10.1 Hz, 1H), 6.15 (dd, J = 17.0, 2.1 Hz, 1H), 5.64 (dd, J = 10.1, 2.1 Hz, 1H), 4.84 (s, 2H), 3.87 (s, 6H), 3.74 (s, 3H), 2.20 (s, 6H);

^{13}C NMR (DMSO-d_6) δ 161.4, 160.5 (2C), 156.2, 145.5, 143.4 (d, J = 4.8 Hz), 143.2 (d, J = 4.8 Hz), 142.2, 141.7 (d, J = 6.0 Hz), 137.8, 131.5, 125.9, 123.7, 114.2, 113.2 (t, J = 18.0 Hz), 101.6, 62.1, 56.9 (3C), 18.3 (2C); HRMS (EI) m/z calculated for $C_{22}H_{24}F_2N_6O_4$ $[M]^+$ 474.1827, found 474.1831.

N-(3-((5-((2,6-Dichloro-3,5-dimethoxybenzyl)oxy)-4,6-dimethylpyrimidin-2-yl)amino)-1-methyl-1H-pyrazol-4-yl)acrylamide (6D)

To a solution of **23D** (28 mg, 0.06 mmol) in DCM (1 mL) was added acryloyl chloride (6 µL, 0.07 mmol) dropwise at 0 °C, and the resulting mixture was stirred at room temperature for 30 minutes. The reaction mixture was diluted with DCM and saturated $NaHCO_3$ and the aqueous layer was extracted with DCM. The combined organic solution was dried over $MgSO_4$, filtered, and concentrated. The residue was purified by flash silica gel column chromatography with 3% to 5% MeOH/DCM to obtain **6D** (25.0 mg, 80%). Pale yellow solid; TLC R_f 0.79 (MeOH/DCM = 9/1); m.p. 235 °C; MS (EI) m/z 506 $[M]^+$; ^1H NMR ($CDCl_3$) δ 10.46 (s, 1H), 8.10 (s, 1H), 7.57 (s, 1H), 6.62 (s, 1H), 6.34 (dd, J = 16.9, 1.7 Hz, 1H), 6.17 (dd, J = 16.9, 9.9 Hz, 1H), 5.66 (dd, J = 9.9, 1.7 Hz, 1H), 5.19 (s, 2H), 3.93 (s, 6H), 3.79 (s, 3H), 2.39 (s, 6H); ^{13}C NMR ($CDCl_3$) δ 162.1, 161.9, 155.3 (2C), 154.8 (2C), 143.8, 138.9, 133.4, 131.4, 126.3, 125.0, 116.7, 111.7 (2C), 98.2, 70.0, 56.8 (2C), 39.3, 19.4 (2C); HRMS (EI) m/z calculated for $C_{22}H_{24}Cl_2N_6O_4$ $[M]^+$ 506.1236, found 506.1240.

N-(2-((5-((2,6-Difluoro-3,5-dimethoxybenzyl)oxy)-3,4,6-trimethylpyridin-2-yl)amino)-3-methylphenyl)acrylamide (6E)

To a solution of **23E** (75 mg, 0.17 mmol) in DCM (3 mL) were added Et_3N (100 µL, 0.72 mmol) and acryloyl chloride (25 µL, 0.31 mmol) dropwise at 0 °C. The resulting mixture was stirred at room temperature for 30 minutes, and diluted with DCM and saturated $NaHCO_3$. The aqueous layer was extracted with DCM, and the combined organic solution was dried over $MgSO_4$, filtered, and concentrated. The residue was purified by flash silica gel column chromatography with 30% to 40% EtOAc/Hexanes to obtain **6E** (19 mg, 23%). White solid; TLC R_f 0.60 (EtOAc/Hexanes = 1/1); m.p. 235 °C; MS (EI) m/z 497 $[M]^+$; ^1H NMR (DMSO-d_6) δ 9.76 (s, 1H), 7.42 (dd, J = 6.0, 3.5 Hz, 1H), 7.08 (dt, J = 10.9, 7.0 Hz, 4H), 6.44 (dd, J = 17.0, 10.0 Hz, 1H), 6.22 (dd, J = 17.0, 2.0 Hz, 1H), 5.72 (dd, J = 10.0, 2.0 Hz, 1H), 4.71 (s, 2H), 3.87 (s, 6H), 2.17 (s, 3H), 2.10 (s, 3H), 2.07 (d, J = 1.9 Hz, 6H); ^{13}C NMR (DMSO-d_6) δ

163.8 (2C), 150.5, 145.6 (d, J = 6.2 Hz), 144.7, 143.6, 143.3 (d, J = 4.6 Hz), 143.2 (d, J = 4.8 Hz), 141.8 (d, J = 6.1 Hz), 139.1, 136.5 (2C), 133.6 (d, J = 6.8 Hz), 131.7, 126.9 (d, J = 11.3 Hz), 124.5, 121.3, 114.1, 113.6 (t, J = 18.3 Hz), 101.5, 61.8, 56.9 (2C), 18.9, 18.5, 12.8, 12.2; HRMS (EI) m/z calculated for $C_{27}H_{29}F_2N_3O_4$ $[M]^+$ 497.2126, found 497.2130.

N-(2-((5-((2,6-Dichloro-3,5-dimethoxybenzyl)oxy)-3,4,6-trimethylpyridin-2-yl)amino)-3-methylphenyl)acrylamide (6F)

To a solution of **23F** (25 mg, 0.05 mmol) in DCM (1 mL) were added Et_3N (30 µL, 0.22 mmol) and acryloyl chloride (10 µL, 0.12 mmol) dropwise at 0 °C. The resulting mixture was stirred at room temperature for 30 minutes and then diluted with DCM and saturated $NaHCO_3$. The aqueous was extracted with DCM, and the combined organic solution was dried over $MgSO_4$, filtered, and concentrated. The residue was dissolved in a small amount of EtOAc and then hexanes was added to get a precipitate. The precipitate was filtered, washed with hexanes to obtain **6F** (11 mg, 10%). White solid; TLC R_f 0.54 (EtOAc/Hexanes = 1/1); m.p. 232 °C; MS (EI) m/z 529 $[M]^+$; ^{1}H NMR (DMSO-d_6) δ 9.74 (s, 1H), 7.42 (d, J = 3.4 Hz, 1H), 7.16 – 7.02 (m, 3H), 6.97 (s, 1H), 6.43 (dd, J = 17.0, 10.0 Hz, 1H), 6.21 (dd, J = 17.0, 2.0 Hz, 1H), 5.71 (dd, J = 10.0, 2.1 Hz, 1H), 4.99 (s, 2H), 3.93 (s, 6H), 2.14 (s, 3H), 2.08 (s, 3H), 2.06 (s, 3H), 2.04 (s, 3H); ^{13}C NMR (DMSO-d_6**)** δ 163.7, 154.3 (2C), 150.2, 144.7, 144.0, 139.1, 136.4, 133.7, 133.5, 133.3, 131.7, 126.9, 126.7, 124.4, 121.2, 114.9 (2C), 114.0, 98.9, 69.3, 56.7, 19.0, 18.8, 12.8 (2C); HRMS (EI) m/z calculated for $C_{27}H_{29}Cl_2N_3O_4$ $[M]^+$ 529.1535, found 529.1528.

N-(2-((5-((2,6-Difluoro-3,5-dimethoxybenzyl)oxy)-4,6-dimethylpyrimidin-2-yl)amino)-3-methylphenyl)acrylamide (6G)

To a solution of **23G** (20 mg, 0.05 mmol) in DCM (2 mL) were added Et_3N (19 µL, 0.14 mmol) and acryloyl chloride (5 µL, 0.06 mmol) dropwise at 0 °C. The resulting mixture was stirred at room temperature for 30 minutes and then diluted with DCM and saturated $NaHCO_3$. The aqueous layer was extracted with DCM, and the combined organic solution was dried over $MgSO_4$, filtered, and concentrated. The residue was dissolved in a small amount of EtOAc and then hexanes was added for precipitation. The precipitate was filtered and washed with hexanes to give **6G** (17 mg, 83%). Pale yellow solid; TLC R_f 0.25 (Hexanes/EtOAc = 1/1); m.p. 170 °C; MS (EI) m/z 484 $[M]^+$; ^{1}H NMR ($CDCl_3$) δ 8.79 (s, 1H), 7.91 (dd, J = 28.3, 7.4 Hz, 1H), 7.19 (dd, J = 9.4, 6.3 Hz, 1H), 7.04 (d, J = 7.4 Hz, 1H), 6.69 (t, J = 8.2 Hz, 1H), 6.59 (s, 1H), 6.29

(dd, *J* = 16.9, 1.5 Hz, 1H), 6.12 (dd, *J* = 16.9, 10.0 Hz, 1H), 5.65 (dd, *J* = 10.0, 1.7 Hz, 1H), 4.88 (s, 2H), 3.89 (s, 6H), 2.35 (d, *J* = 1.8 Hz, 6H), 2.28 (s, 3H); ^{13}C NMR ($CDCl_3$) δ 167.8, 163.7, 162.2 (2C), 156.6, 147.1 (d, *J* = 5.6 Hz), 143.8 (d, *J* = 4.8 Hz), 143.6 (d, *J* = 5.0 Hz), 143.4, 143.2 (d, *J* = 5.7 Hz), 134.4, 134.3, 132.1, 129.3, 126.8, 126.5, 122.0 (d, *J* = 16.8 Hz), 113.7 (t, *J* = 18.0 Hz), 102.2, 62.6, 57.5 (2C), 18.9 (3C); HRMS (EI) *m*/*z* calculated for $C_{25}H_{26}F_2N_4O_4$ $[M]^+$ 484.1922, found 484.1926.

***N*-(2-((5-((2,6-Dichloro-3,5-dimethoxybenzyl)oxy)-4,6-dimethylpyrimidin-2-yl)amino)-3-methylphenyl)acrylamide (6H)**

To a solution of **23H** (20 mg, 0.04 mmol) in DCM (2 mL) were added Et_3N (18 μL, 0.13 mmol) and acryloyl chloride (4 μL, 0.05 mmol) dropwise at 0 °C. The resulting mixture was stirred at room temperature for 30 minutes and then diluted with DCM and saturated $NaHCO_3$. The aqueous layer was extracted with DCM, and the combined organic solution was dried over $MgSO_4$, filtered, and concentrated. The residue was dissolved in a small amount of EtOAc and then hexanes was added for precipitation. The precipitate was filtered and washed with hexanes to give **6H** (20 mg, 88%). Pale yellow solid; TLC R_f 0.23 (Hexanes/EtOAc = 1/1); m.p. 220 °C; MS (EI) *m*/*z* 516 $[M]^+$; ^{1}H NMR ($CDCl_3$) δ 8.78 (s, 1H), 7.97 (d, *J* = 7.3 Hz, 1H), 7.19 (t, *J* = 7.8 Hz, 1H), 7.03 (d, *J* = 7.3 Hz, 1H), 6.60 (d, *J* = 4.3 Hz, 2H), 6.28 (dd, *J* = 16.9, 1.5 Hz, 1H), 6.11 (dd, *J* = 16.9, 10.0 Hz, 1H), 5.65 (dd, *J* = 10.0, 1.7 Hz, 1H), 5.19 (s, 2H), 3.93 (s, 6H), 2.30 (s, 6H), 2.27 (s, 3H); ^{13}C NMR ($CDCl_3$) δ 167.8, 163.7, 162.1, 156.4, 154.8 (2C), 145.9, 143.8, 134.5, 134.3, 133.5, 132.2, 129.3, 126.7, 126.5, 121.7, 116.7 (2C), 98.1, 69.9, 56.8 (2C), 19.4 (2C), 18.9; HRMS (EI) *m*/*z* calculated for $C_{25}H_{26}Cl_2N_4O_4$ $[M]^+$ 516.1331, found 516.1333.

***N*-(4-((5-((2,6-Difluoro-3,5-dimethoxybenzyl)oxy)-3,4,6-trimethylpyridin-2-yl)amino)-3-methylphenyl)acrylamide (6I)**

To a solution of **23I** (30 mg, 0.07 mmol) in DCM (2 mL) were added Et_3N (47 μL, 0.34 mmol) and acryloyl chloride (6 μL, 0.07 mmol) dropwise at 0 °C. The resulting mixture was stirred at room temperature for 30 minutes and then diluted with DCM and saturated $NaHCO_3$. The aqueous layer was extracted with DCM and the combined organic solution was dried over $MgSO_4$, filtered, and concentrated. The residue was purified by flash silica gel column chromatography with 20% to 40% EtOAc/Hexanes to obtain **6I** (10 mg, 29%). White solid; TLC R_f 0.61 (EtOAc); m.p. 224 °C; MS (EI) *m*/*z* 497 $[M]^+$; ^{1}H NMR (DMSO-d_6) δ 9.95 (s,

1H), 7.55–7.34 (m, 2H), 7.19 (d, J = 8.5 Hz, 1H), 7.15–6.98 (m, 2H), 6.43 (dd, J = 16.9, 10.0 Hz, 1H), 6.22 (dd, J = 16.9, 2.0 Hz, 1H), 5.82–5.61 (m, 1H), 4.75 (s, 2H), 3.88 (s, 6H), 2.16 (s, 3H), 2.15 (s, 3H), 2.12 (s, 3H), 2.08 (s, 3H); ^{13}C NMR (DMSO-d_6) δ 162.7, 150.5, 144.9 (2C), 144.3, 143.3 (d, J = 4.2 Hz), 143.2 (d, J = 4.3 Hz), 139.2, 136.7, 133.8, 132.1, 131.4, 131.3, 126.1, 123.3, 121.3, 117.2, 115.7, 113.6 (t, J = 18.9 Hz), 101.5, 61.8, 56.9 (2C), 18.5 (s, J = 2.6 Hz), 18.5, 13.1, 12.2; HRMS (EI) m/z calculated for $C_{27}H_{29}F_2N_3O_4$ $[M]^+$ 497.2126, found 497.2125.

***N*-(4-((5-((2,6-Dichloro-3,5-dimethoxybenzyl)oxy)-3,4,6-trimethylpyridin-2-yl)amino)-3-methylphenyl)acrylamide (6J)**

To a solution of **23J** (25 mg, 0.05 mmol) in DCM (2 mL) were added Et_3N (36 µL, 0.26 mmol) and acryloyl chloride (5 µL, 0.06 mmol) dropwise at 0 °C. The resulting mixture was stirred at room temperature for 30 minutes and diluted with DCM and saturated $NaHCO_3$. The aqueous layer was extracted with DCM and the combined organic layer was dried over $MgSO_4$, filtered, and concentrated. The residual solid was washed with MeOH to give **6J** (14 mg, 50%). White solid; TLC R_f 0.40 (EtOAc/Hexanes = 1/1, developed twice); m.p. 252 °C; MS (EI) m/z 529 $[M]^+$; ^{1}H NMR (DMSO-d_6) δ 9.92 (s, 1H), 7.53–7.33 (m, 2H), 7.18 (d, J = 8.6 Hz, 1H), 6.98 (s, 2H), 6.43 (dd, J = 17.0, 10.0 Hz, 1H), 6.22 (dd, J = 16.9, 2.2 Hz, 1H), 5.71 (dd, J = 9.9, 2.1 Hz, 1H), 5.04 (s, 2H), 3.94 (s, 6H), 2.13 (d, J = 5.0 Hz, 9H), 2.06 (s, 3H); ^{13}C NMR (DMSO-d_6) δ 162.6, 154.3 (2C), 150.2, 144.9, 144.8, 139.2, 136.9, 133.6, 133.3, 132.2, 131.0, 126.0, 122.8, 121.3, 117.2, 115.7, 114.9 (2C), 98.9, 69.2, 56.7 (2C), 19.1, 18.4, 13.1, 12.8; HRMS (EI) m/z calculated for $C_{27}H_{29}Cl_2N_3O_4$ $[M]^+$ 529.1535, found 529.1531.

***N*-(4-((5-((2,6-Difluoro-3,5-dimethoxybenzyl)oxy)-4,6-dimethylpyrimidin-2-yl)amino)-3-methylphenyl)acrylamide (6K)**

To a solution of **23K** (40 mg, 0.09 mmol) in DCM (2 mL) were added Et_3N (39 µL, 0.28 mmol) and acryloyl chloride (11 µL, 0.14 mmol) dropwise at 0 °C. The resulting mixture was stirred at room temperature for 6 h, and diluted with DCM and saturated $NaHCO_3$. The aqueous layer was extracted with DCM and the combined organic solution was dried over $MgSO_4$, filtered, and concentrated. The residue was purified by flash silica gel column chromatography with 40% to 50% EtOAc/Hexanes to obtain **6K** (14 mg, 31%). Pale yellow solid; TLC R_f 0.25 (Hexanes/EtOAc = 1/1); m.p. 213 °C; MS (EI) m/z 484 $[M]^+$; ^{1}H NMR ($CDCl_3$) δ 8.06 (d, J =

8.7 Hz, 1H), 7.51 (s, 1H), 7.42–7.27 (m, 2H), 6.75 (s, 1H), 6.69 (t, J = 8.2 Hz, 1H), 6.48–6.34 (m, 1H), 6.22 (dd, J = 16.7, 10.1 Hz, 1H), 5.73 (dd, J = 10.0, 1.3 Hz, 1H), 4.88 (s, 2H), 3.89 (s, 6H), 2.40 (s, 6H), 2.29 (s, 3H); ^{13}C NMR ($CDCl_3$) δ 163.4, 161.5 (2C), 155.8 (2C), 147.2 (d, J = 5.9 Hz), 143.8 (d, J = 4.9 Hz), 143.6 (d, J = 4.9 Hz), 143.3 (d, J = 4.3 Hz), 134.9, 132.7, 131.4, 128.7, 127.5, 122.3, 121.3, 118.4, 113.9 (t, J = 18.0 Hz), 102.2, 62.5, 57.5 (2C), 18.9 (2C), 18.4; HRMS (EI) m/z calculated for $C_{25}H_{26}F_2N_4O_4$ $[M]^+$ 484.1922, found 484.1922.

_N_-(4-((5-((2,6-Dichloro-3,5-dimethoxybenzyl)oxy)-4,6-dimethylpyrimidin-2-yl)amino)-3-methylphenyl)acrylamide (6L)

To a solution of **23L** (40 mg, 0.09 mmol) in DCM (2 mL) were added Et_3N (36 μL, 0.26 mmol) and acryloyl chloride (11 μL, 0.13 mmol) dropwise at 0 °C. The resulting mixture was stirred at room temperature for 30 min, and diluted with DCM and saturated $NaHCO_3$. The aqueous layer was extracted with DCM and the combined organic solution was dried over $MgSO_4$, filtered, and concentrated. The residue was purified by flash silica gel column chromatography with 20% to 30% EtOAc/Hexanes to **6L** (38 mg, 84%). White solid; TLC R_f 0.44 (DCM/MeOH = 20/1); m.p. 200 °C; MS (EI) m/z 516 $[M]^+$; ^{1}H NMR ($CDCl_3$) δ 8.01 (d, J = 8.7 Hz, 1H), 7.60 (s, 1H), 7.48 (s, 1H), 7.31 (d, J = 8.3 Hz, 1H), 6.72 (s, 1H), 6.60 (s, 1H), 6.40 (dd, J = 16.8, 1.2 Hz, 1H), 6.21 (dd, J = 16.8, 10.1 Hz, 1H), 5.70 (dd, J = 10.0, 1.5 Hz, 1H), 5.17 (s, 2H), 3.92 (s, 6H), 2.36 (s, 6H), 2.25 (s, 3H); ^{13}C NMR ($CDCl_3$) δ 163.5, 161.4 (2C), 155.6, 154.7 (2C), 143.7, 134.8, 133.6, 132.9, 131.4, 128.8, 127.4, 122.3, 121.3, 118.4, 116.7 (2C), 98.1, 69.8, 56.7 (2C), 19.4 (2C), 18.4; HRMS (EI) m/z calculated for $C_{25}H_{26}Cl_2N_4O_4$ $[M]^+$ 516.1331, found 516.1338.

_N_-(3-Chloro-2-((5-((2,6-difluoro-3,5-dimethoxybenzyl)oxy)-3,4,6-trimethylpyridin-2-yl)amino)-5-fluorophenyl)acrylamide (6M)

To a solution of **23M** (25 mg, 0.05 mmol) in DCM (1 mL) were added Et_3N (22 μL, 0.16 mmol) and acryloyl chloride (8 μL, 0.10 mmol) dropwise at 0 °C. The resulting mixture was stirred at room temperature for 30 min, and diluted with DCM and saturated $NaHCO_3$. The aqueous layer was extracted with DCM and the combined organic solution was dried over $MgSO_4$, filtered, and concentrated. The residue was purified by flash silica gel column chromatography with 20% to 35% EtOAc/Hexanes to obtain **6M** (16 mg, 57%). White solid; TLC R_f 0.67 (EtOAc/Hexanes = 1/1); m.p. 215 °C; MS (EI) m/z 535 $[M]^+$; ^{1}H NMR (DMSO-d_6) δ 9.64 (s,

1H), 7.89 (dd, *J* = 10.9, 2.9 Hz, 1H), 7.24 (dd, *J* = 8.3, 3.0 Hz, 1H), 7.18 (s, 1H), 7.07 (t, *J* = 8.4 Hz, 1H), 6.56 (dd, *J* = 17.0, 10.1 Hz, 1H), 6.23 (dd, *J* = 17.0, 1.9 Hz, 1H), 5.74 (dd, *J* = 10.1, 1.9 Hz, 1H), 4.71 (s, 2H), 3.87 (s, 6H), 2.18 (s, 3H), 2.16 (s, 3H), 2.06 (s, 3H); ^{13}C NMR (DMSO-d_6) δ 163.8, 160.5, 156.7, 150.5, 145.6 (d, *J* = 6.0 Hz), 144.8, 144.3, 143.3 (d, *J* = 4.8 Hz), 143.2 (d, *J* = 4.8 Hz), 141.8 (d, *J* = 6.1 Hz), 139.2, 137.9 (d, *J* = 12.9 Hz), 133.4 (d, *J* = 13.1 Hz), 131.7, 127.9–127.3 (m), 114.9, 113.6 (t, *J* = 18.2 Hz), 111.9 (d, *J* = 25.7 Hz), 107.8 (d, *J* = 26.9 Hz), 101.5, 61.8, 56.9 (2C), 18.5, 13.0, 12.2; HRMS (EI) *m/z* calculated for $C_{26}H_{25}ClF_3N_3O_4$ $[M]^+$ 535.1486, found 535.1496.

***N*-(3-Chloro-2-((5-((2,6-dichloro-3,5-dimethoxybenzyl)oxy)-3,4,6-trimethylpyridin-2-yl)amino)-5-fluorophenyl)acrylamide (6N)**

To a solution of **23N** (50 mg, 0.10 mmol) in DCM (2 mL) were added Et_3N (95 µL, 0.68 mmol) and acryloyl chloride (39 µL, 0.49 mmol) dropwise at 0 °C. The resulting mixture was stirred at room temperature for 30 min, and diluted with DCM and saturated $NaHCO_3$. The aqueous layer was extracted with DCM and the combined organic solution was dried over $MgSO_4$, filtered, and concentrated. The residue was purified by flash silica gel column chromatography with 20% to 30% EtOAc/Hexanes to obtain **6N** (54 mg, 97%). Pale yellow solid; TLC R_f 0.52 (EtOAc/Hexanes = 1/1); m.p. 227 °C; MS (EI) *m/z* 569 $[M]^+$; ^{1}H NMR (DMSO-d_6) δ 9.65 (s, 1H), 7.90 (dd, *J* = 10.9, 3.0 Hz, 1H), 7.25 (dd, *J* = 8.3, 3.0 Hz, 1H), 7.17 (s, 1H), 6.97 (s, 1H), 6.56 (dd, *J* = 16.9, 10.2 Hz, 1H), 6.23 (dd, *J* = 17.0, 1.8 Hz, 1H), 5.74 (dd, *J* = 10.1, 1.8 Hz, 1H), 4.97 (s, 2H), 3.93 (s, 6H), 2.16 (s, 3H), 2.14 (s, 3H), 2.04 (s, 3H); ^{13}C NMR (DMSO-d_6) δ 163.7, 154.3 (2C), 150.2, 144.8, 144.7, 139.3, 137.8 (d, *J* = 12.8 Hz), 136.5, 133.3, 133.1, 131.6, 127.6, 127.5, 114.9 (2C), 114.7, 111.8 (d, *J* = 25.4 Hz), 107.7 (d, *J* = 26.8 Hz), 98.9, 69.2, 56.7 (2C), 19.1, 12.9, 12.8; HRMS (EI) *m/z* calculated for $C_{26}H_{25}Cl_3FN_3O_4$ $[M]^+$ 567.0895, found 567.0896.

***N*-(3-Chloro-2-((5-((2,6-difluoro-3,5-dimethoxybenzyl)oxy)-4,6-dimethylpyrimidin-2-yl)amino)-5-fluorophenyl)acrylamide (6O)**

To a solution of **23O** (30 mg, 0.06 mmol) in DCM (2 mL) were added Et_3N (27 µL, 0.19 mmol) and acryloyl chloride (8 µL, 0.10 mmol) dropwise at 0 °C. The resulting mixture was stirred at room temperature for 12 h, and diluted with DCM and saturated $NaHCO_3$. The aqueous layer was extracted with DCM and the combined organic solution was dried over $MgSO_4$, filtered,

and concentrated. The residue was purified by flash silica gel column chromatography with 30% to 40% EtOAc/Hexanes to obtain **6O** (7 mg, 20%). White solid; TLC R_f 0.35 (EtOAc/Hexanes = 1/1); m.p. 160 °C; MS (EI) *m/z* 522 $[M]^+$; ^{1}H NMR ($CDCl_3$) δ 9.12 (s, 1H), 8.01 (dd, *J* = 10.5, 2.7 Hz, 1H), 6.98 (dd, *J* = 7.7, 2.9 Hz, 1H), 6.70 (t, *J* = 8.2 Hz, 2H), 6.32 (dd, *J* = 16.9, 1.1 Hz, 1H), 6.11 (dd, *J* = 16.9, 10.1 Hz, 1H), 5.71 (dd, *J* = 10.1, 1.1 Hz, 1H), 4.90 (t, *J* = 1.7 Hz, 2H), 3.89 (s, 6H), 2.38 (s, 6H); ^{13}C NMR ($CDCl_3$) δ 163.7, 162.4, 162.2, 158.3, 156.4, 147.1 (d, *J* = 5.8 Hz), 144.2, 143.9 (d, *J* = 5.0 Hz), 143.7 (d, *J* = 5.0 Hz), 143.2 (d, *J* = 5.7 Hz), 136.8 (d, *J* = 12.9 Hz), 131.6, 131.1 (d, *J* = 12.7 Hz), 127.9, 124.3 (d, *J* = 3.4 Hz), 113.6 (t, *J* = 17.8 Hz), 112.4 (d, *J* = 26.3 Hz), 109.4 (d, *J* = 27.1 Hz), 102.3 (t, *J* = 2.5 Hz), 62.6 (t, *J* = 3.6 Hz), 57.5 (2C), 18.9 (2C); HRMS (EI) *m/z* calculated for $C_{24}H_{22}ClF_3N_4O_4$ $[M]^+$ 522.1282, found 522.1285.

N-(3-Chloro-2-((5-((2,6-dichloro-3,5-dimethoxybenzyl)oxy)-4,6-dimethylpyrimidin-2-yl)amino)-5-fluorophenyl)acrylamide (6P)

To a solution of **23P** (20 mg, 0.04 mmol) in DCM (1 mL) were added Et_3N (25 µL, 0.18 mmol) and acryloyl chloride (4 µL, 0.05 mmol) dropwise at 0 °C. The resulting mixture was stirred at room temperature for 2 h, and diluted with DCM and saturated $NaHCO_3$. The aqueous layer was extracted with DCM and the combined organic solution was dried over $MgSO_4$, filtered, and concentrated. The residue was purified by flash silica gel column chromatography with 3% to 5% MeOH/DCM to obtain **6P** (10 mg, 44%). White solid; TLC R_f 0.53 (DCM/MeOH = 20/1); m.p. 230 °C; MS (EI) *m/z* 556 $[M]^+$; ^{1}H NMR ($CDCl_3$) δ 9.13 (s, 1H), 8.00 (dd, *J* = 10.3, 2.7 Hz, 1H), 6.97 (dd, *J* = 7.7, 2.9 Hz, 1H), 6.62 (s, 1H), 6.57 (s, 1H), 6.31 (dd, *J* = 16.9, 1.3 Hz, 1H), 6.09 (dd, *J* = 16.9, 10.2 Hz, 1H), 5.71 (dd, *J* = 10.2, 1.3 Hz, 1H), 5.20 (s, 2H), 3.94 (s, 6H), 2.34 (s, 6H); ^{13}C NMR ($CDCl_3$) δ 163.7, 162.3 (2C), 158.2, 156.4, 154.8 (2C), 144.7, 136.7 (d, *J* = 12.8 Hz), 133.5, 131.7, 131.0 (d, *J* = 12.8 Hz), 127.8, 124.6, 116.7 (2C), 112.4 (d, *J* = 26.2 Hz), 109.4 (d, *J* = 27.2 Hz), 98.2, 69.9, 56.8 (2C), 19.5 (2C); HRMS (EI) *m/z* calculated for $C_{24}H_{22}Cl_3FN_4O_4$ $[M]^+$ 554.0691, found 554.0693.

N-(2-((5-((2,6-Difluoro-3,5-dimethoxybenzyl)oxy)-3,4,6-trimethylpyridin-2-yl)amino)-3-methoxyphenyl)acrylamide (6Q)

To a solution of **23Q** (30 mg, 0.065 mmol) in DCM (2 mL) were added Et_3N (45 µL, 0.33 mmol) and acryloyl chloride (11 µL, 0.13 mmol) dropwise at 0 °C. The resulting mixture was

stirred at room temperature for 30 min, and diluted with DCM and saturated $NaHCO_3$. The aqueous layer was extracted with DCM and the combined organic solution was dried over $MgSO_4$, filtered, and concentrated. The residue was purified by flash silica gel column chromatography with 20% to 40% EtOAc/Hexanes to obtain **6Q** (12 mg, 35%). White solid; TLC R_f 0.39 (EtOAc/Hexanes = 1/1); m.p. 171 °C; MS (EI) *m/z* 513 $[M]^+$; ^{1}H NMR (DMSO-d_6) δ 9.82 (s, 1H), 7.37 (d, *J* = 8.0 Hz, 1H), 7.20–6.99 (m, 2H), 6.87 (d, *J* = 8.2 Hz, 1H), 6.78 (s, 1H), 6.43–6.24 (m, 1H), 6.15 (d, *J* = 16.9 Hz, 1H), 5.69 (d, *J* = 9.9 Hz, 1H), 4.75 (s, 2H), 3.88 (s, 6H), 3.72 (s, 3H), 2.18 (s, 3H), 2.13 (d, *J* = 2.3 Hz, 6H); ^{13}C NMR **(**DMSO-d_6**)** δ 163.1, 154.4, 150.7, 145.7, 144.6, 144.0, 143.3 (d, *J* = 4.9 Hz), 143.1 (d, *J* = 4.8 Hz), 141.8 (d, *J* = 6.1 Hz), 139.4, 134.0, 132.0, 126.3, 124.4, 123.6, 115.8, 114.7, 113.5, 108.1, 101.7, 61.8, 56.9 (2C), 55.8, 18.3, 12.7, 12.2; HRMS (EI) *m/z* calculated for $C_{27}H_{29}F_2N_3O_5$ $[M]^+$ 513.2075, found 513.2073.

***N*-(2-((5-((2,6-Dichloro-3,5-dimethoxybenzyl)oxy)-3,4,6-trimethylpyridin-2-yl)amino)-3-methoxyphenyl)acrylamide (6R)**

To a solution of **23R** (30 mg, 0.06 mmol) in DCM (2 mL) were added Et_3N (43 µL, 0.31 mmol) and acryloyl chloride (10 µL, 0.12 mmol) dropwise at 0 °C. The resulting mixture was stirred at room temperature for 30 min, and diluted with DCM and saturated $NaHCO_3$. The aqueous layer was extracted with DCM and the combined organic solution was dried over $MgSO_4$, filtered, and concentrated. The residue was purified by flash silica gel column chromatography with 20% to 40% EtOAc/Hexanes to obtain **6R** (22 mg, 66%). Yellow solid; TLC R_f 0.31 (EtOAc/Hexanes = 1/1); m.p. 160 °C; MS (EI) *m/z* 545 $[M]^+$; ^{1}H NMR (DMSO-d_6) δ 9.86 (s, 1H), 7.36 (d, *J* = 8.1 Hz, 1H), 7.12 (t, *J* = 8.2 Hz, 1H), 6.86 (d, *J* = 7.4 Hz, 1H), 6.79 (s, 1H), 6.31 (dd, *J* = 17.0, 9.9 Hz, 1H), 6.14 (dd, *J* = 17.0, 2.1 Hz, 1H), 5.75 (s, 1H), 5.69 (dd, *J* = 9.9, 2.1 Hz, 1H), 5.01 (s, 2H), 3.93 (s, 6H), 3.71 (s, 3H), 2.15 (s, 3H), 2.11 (s, 3H), 2.09 (s, 3H); ^{13}C NMR (DMSO-d_6) δ 163.1, 154.4 (2C), 154.3, 150.5, 144.6, 144.5, 139.6, 133.9, 133.3, 132.1, 126.5, 124.4, 123.6, 115.9, 114.8 (2C), 114.6, 108.2, 98.8, 69.3, 56.7 (2C), 55.8, 19.0, 12.9, 12.8; HRMS (EI) *m/z* calculated for $C_{27}H_{29}Cl_2N_3O_5$ $[M]^+$ 545.1484, found 545.1485.

***N*-(2-((5-((2,6-Difluoro-3,5-dimethoxybenzyl)oxy)-4,6-dimethylpyrimidin-2-yl)amino)-3-methoxyphenyl)acrylamide (6S)**

To a solution of **23S** (25 mg, 0.06 mmol) in DCM (2 mL) were added Et_3N (23 µL, 0.17 mmol) and acryloyl chloride (9 µL, 0.11 mmol) dropwise at 0 °C. The resulting mixture was stirred at room temperature for 1 h, and diluted with DCM and saturated $NaHCO_3$. The aqueous layer was extracted with DCM and the combined organic solution was dried over $MgSO_4$, filtered, and concentrated. The residue was purified by flash silica gel column chromatography with 30% to 45% EtOAc/Hexanes to obtain **6S** (21 mg, 75%). White solid; TLC R_f 0.26 (Hexanes/EtOAc = 1/1); m.p. 137 °C; MS (EI) *m/z* 500 $[M]^+$; ^1H NMR ($CDCl_3$) δ 9.90 (s, 1H), 7.65 (d, *J* = 8.0 Hz, 1H), 7.23–7.12 (m, 1H), 7.01 (s, 1H), 6.80–6.61 (m, 2H), 6.30 (d, *J* = 16.9 Hz, 1H), 6.10 (dd, *J* = 16.9, 10.1 Hz, 1H), 5.66 (dd, *J* = 10.1, 1.5 Hz, 1H), 4.88 (s, 2H), 3.89 (s, 6H), 3.86 (s, 3H), 2.41 (s, 6H); ^{13}C NMR ($CDCl_3$) δ 167.7, 163.7, 162.1 (2C), 156.9, 152.5 (2C), 147.0 (d, *J* = 5.8 Hz), 143.7 (dd, *J* = 10.4, 4.9 Hz), 143.1 (d, *J* = 5.7 Hz), 132.9, 132.4, 126.6, 125.3, 121.3, 117.4, 113.6 (t, *J* = 18.0 Hz), 106.9, 102.0 , 62.6, 57.4 (2C), 56.0, 18.9 (2C); HRMS (EI) *m/z* calculated for $C_{25}H_{26}F_2N_4O_5$ $[M]^+$ 500.1871, found 500.1870.

_N_-(2-((5-((2,6-dichloro-3,5-dimethoxybenzyl)oxy)-4,6-dimethylpyrimidin-2-yl)amino)-3-methoxyphenyl)acrylamide (6T)

To a solution of **23T** (25 mg, 0.05 mmol) in DCM (2 mL) were added Et_3N (22 µL, 0.16 mmol) and acryloyl chloride (8 µL, 0.10 mmol) dropwise at 0 °C. The resulting mixture was stirred at room temperature for 1 h, and diluted with DCM and saturated $NaHCO_3$. The aqueous layer was extracted with DCM and the combined organic solution was dried over $MgSO_4$, filtered, and concentrated. The residue was purified by flash silica gel column chromatography with 30% to 45% EtOAc/Hexanes to obtain **6T** (23 mg, 84%). White solid; TLC R_f 0.27 (Hexanes/EtOAc = 1/1); m.p. 196 °C; MS (EI) *m/z* 532 $[M]^+$; ^1H NMR ($CDCl_3$) δ 9.89 (s, 1H), 7.60 (dd, *J* = 24.8, 8.1 Hz, 1H), 7.17 (td, *J* = 8.3, 3.9 Hz, 1H), 7.00 (s, 1H), 6.73 (d, *J* = 8.1 Hz, 1H), 6.61 (s, 1H), 6.29 (d, *J* = 16.1 Hz, 1H), 6.09 (dd, *J* = 16.9, 10.1 Hz, 1H), 5.65 (dd, *J* = 10.1, 1.4 Hz, 1H), 5.18 (s, 2H), 3.94 (s, 6H), 3.85 (s, 3H), 2.39 (s, 6H); ^{13}C NMR ($CDCl_3$) δ 167.6, 163.7, 162.0 (2C), 156.7, 154.7 (2C), 152.5, 144.0, 133.4, 132.9, 132.4, 126.5, 125.3, 121.3, 117.4, 116.5, 106.9, 98.0, 69.9, 56.7 (2C), 56.0, 19.5 (2C); HRMS (EI) *m/z* calculated for $C_{25}H_{26}Cl_2N_4O_5$ $[M]^+$ 532.1280, found 532.1282.

_N_-(2-((5-((2,6-Difluoro-3,5-dimethoxybenzyl)oxy)-3,4,6-trimethylpyridin-2-yl)amino)-4-methoxyphenyl)acrylamide (6U)

To a solution of **23U** (25 mg, 0.05 mmol) in DCM (1 mL) were added Et_3N (23 µL, 0.16 mmol) and acryloyl chloride (7 µL, 0.08 mmol) dropwise at 0 °C. The resulting mixture was stirred at room temperature for 30 min, and diluted with DCM and saturated $NaHCO_3$. The aqueous layer was extracted with DCM and the combined organic solution was dried over $MgSO_4$, filtered, and concentrated. The residue was dissolved in a small amount of $CHCl_3$ then hexanes was added to get a precipitate. The precipitate was filtered, washed with hexanes to obtain **6U** (22 mg, 80%). Light blue solid; TLC R_f 0.34 (DCM/MeOH = 20/1); m.p. 215 °C; MS (EI) *m/z* 513 $[M]^+$; ^1H NMR (DMSO-d_6) δ 9.89 (s, 1H), 7.46 (d, *J* = 2.8 Hz, 1H), 7.38 (s, 1H), 7.17 (d, *J* = 8.7 Hz, 1H), 7.08 (t, *J* = 8.4 Hz, 1H), 6.60–6.49 (m, 1H), 6.50–6.36 (m, 1H), 6.26 (dd, *J* = 17.0, 1.9 Hz, 1H), 5.76 (dd, *J* = 10.0, 2.0 Hz, 1H), 4.78 (s, 2H), 3.88 (s, 6H), 3.72 (s, 3H), 2.24 (s, 3H), 2.17 (s, 3H), 2.02 (s, 3H); ^{13}C NMR (DMSO-d_6) δ 164.0, 157.2 (2C), 148.9, 145.6 (d, *J* = 6.2 Hz), 144.9 (d, *J* = 16.8 Hz), 143.3 (d, *J* = 4.9 Hz), 143.2 (d, *J* = 4.6 Hz), 141.8 (d, *J* = 6.0 Hz), 139.9, 137.3, 131.3 (2C), 126.7, 126.0, 120.8, 116.2, 113.5 (t, *J* = 18.2 Hz), 106.7 (d, *J* = 22.9 Hz), 101.5, 61.9, 56.9 (2C), 55.0, 18.5, 12.8, 12.3; HRMS (EI) *m/z* calculated for $C_{27}H_{29}F_2N_3O_5$ $[M]^+$ 513.2075, found 513.2075.

***N*-(2-((5-((2,6-Dichloro-3,5-dimethoxybenzyl)oxy)-3,4,6-trimethylpyridin-2-yl)amino)-4-methoxyphenyl)acrylamide (6V)**

To a solution of **23V** (30 mg, 0.06 mmol) in DCM (2 mL) were added Et_3N (43 µL, 0.31 mmol) and acryloyl chloride (6 µL, 0.07 mmol) dropwise at 0 °C. The resulting mixture was stirred at room temperature for 30 min, and diluted with DCM and saturated $NaHCO_3$. The aqueous layer was extracted with DCM and the combined organic solution was dried over $MgSO_4$, filtered, and concentrated. The residue was dissolved in a small amount of $CHCl_3$ then hexanes was added to get a precipitate. The precipitate was filtered, washed with hexanes to obtain **6V** (19 mg, 56%). Light green solid; TLC R_f 0.53 (DCM/MeOH = 20/1); m.p. 212 °C; MS (EI) *m/z* 545 $[M]^+$; ^1H NMR (DMSO-d_6) δ 9.83 (s, 1H), 7.40 (d, *J* = 2.7 Hz, 1H), 7.34 (s, 1H), 7.17 (d, *J* = 8.7 Hz, 1H), 6.98 (s, 1H), 6.54 (dd, *J* = 8.7, 2.8 Hz, 1H), 6.48–6.38 (m, 1H), 6.25 (dd, *J* = 17.0, 2.0 Hz, 1H), 5.79–5.71 (m, 1H), 5.07 (s, 2H), 3.94 (s, 6H), 3.71 (s, 3H), 2.22 (s, 3H), 2.13 (s, 3H), 2.00 (s, 3H); ^{13}C NMR (DMSO-d_6) δ 163.9, 157.2, 154.3 (2C), 148.6, 145.3, 145.1, 139.9, 137.5, 133.2, 131.3, 126.6, 125.9, 120.6, 116.3, 114.9 (2C), 106.7, 106.1, 98.9, 69.2, 56.7 (2C), 55.0, 19.1, 12.9, 12.8; HRMS (EI) *m/z* calculated for $C_{27}H_{29}Cl_2N_3O_5$ $[M]^+$ 545.1484, found 545.1476.

***N*-(2-((5-((2,6-Difluoro-3,5-dimethoxybenzyl)oxy)-4,6-dimethylpyrimidin-2-yl)amino)-4-methoxyphenyl)acrylamide (6W)**

To a solution of **23W** (60 mg, 0.13 mmol) in DCM (1 mL) were added Et_3N (56 µL, 0.40 mmol) and acryloyl chloride (13 µL, 0.16 mmol) dropwise at 0 °C. The resulting mixture was stirred at room temperature for 30 min, and diluted with DCM and saturated $NaHCO_3$. The aqueous layer was extracted with DCM and the combined organic solution was dried over $MgSO_4$, filtered, and concentrated. The residue was purified by flash silica gel column chromatography with 3% to 5% MeOH/DCM to obtain **6W** (7 mg, 10%). Pale yellow solid; TLC R_f 0.42 (DCM/MeOH = 20/1); m.p. 198 °C; MS (EI) *m/z* 500 $[M]^+$; ^1H NMR ($CDCl_3$) δ 8.49 (s, 1H), 7.62 (d, *J* = 8.9 Hz, 1H), 7.15 (d, *J* = 2.7 Hz, 1H), 6.97 (s, 1H), 6.72 (dt, *J* = 12.1, 5.4 Hz, 2H), 6.34 (d, *J* = 16.6 Hz, 1H), 6.16 (dd, *J* = 16.9, 10.1 Hz, 1H), 5.69 (d, *J* = 9.9 Hz, 1H), 4.89 (t, *J* = 1.7 Hz, 2H), 3.90 (s, 6H), 3.80 (s, 3H), 2.40 (s, 6H); ^{13}C NMR ($CDCl_3$) δ 165.6, 164.2, 161.9 (2C), 157.8, 156.2, 147.1, 143.8 (d, *J* = 4.9 Hz), 143.7–143.6 (m), 143.3 (d, *J* = 6.1 Hz), 134.0, 131.7, 127.0, 126.8, 123.3, 118.2, 113.7 (t, *J* = 18.0 Hz), 110.4, 108.85, 102.2, 62.5, 57.5 (2C), 55.6, 19.0 (2C); HRMS (EI) *m/z* calculated for $C_{25}H_{26}F_2N_4O_5$ $[M]^+$ 500.1871, found 500.1870.

***N*-(2-((5-((2,6-Dichloro-3,5-dimethoxybenzyl)oxy)-4,6-dimethylpyrimidin-2-yl)amino)-4-methoxyphenyl)acrylamide (6X)**

To a solution of **23X** (20 mg, 0.04 mmol) in DCM (2 mL) were added Et_3N (18 µL, 0.13 mmol) and acryloyl chloride (5 µL, 0.06 mmol) dropwise at 0 °C. The resulting mixture was stirred at room temperature for 12 h, and diluted with DCM and saturated $NaHCO_3$. The aqueous layer was extracted with DCM and the combined organic solution was dried over $MgSO_4$, filtered, and concentrated. The residue was purified by flash silica gel column chromatography with 50% to 60% EtOAc/Hexanes to obtain **6X** (9 mg, 42%). Pale yellow solid; TLC R_f 0.34 (DCM/MeOH = 20/1); m.p. 217 °C; MS (EI) *m/z* 532 $[M]^+$; ^1H NMR ($CDCl_3$) δ 8.53 (s, 1H), 7.63 (d, *J* = 8.9 Hz, 1H), 7.26–7.11 (m, 2H), 6.72 (dd, *J* = 8.8, 2.7 Hz, 1H), 6.62 (s, 1H), 6.34 (d, *J* = 15.8 Hz, 1H), 6.18 (dd, *J* = 17.0, 9.9 Hz, 1H), 5.68 (d, *J* = 9.9 Hz, 1H), 5.20 (s, 2H), 3.93 (s, 6H), 3.79 (s, 3H), 2.37 (s, 6H); ^{13}C NMR ($CDCl_3$) δ 164.2, 161.9 (2C), 157.7, 155.5, 154.8 (2C), 143.9, 133.7, 133.4, 131.7, 127.0, 126.8, 123.3, 116.7 (2C), 110.4, 108.9, 98.2, 69.9, 56.8 (2C), 55.6, 19.4 (2C); HRMS (EI) *m/z* calculated for $C_{25}H_{26}Cl_2N_4O_5$ $[M]^+$ 532.1280, found 532.1276.

***N*-(2-((5-((2,6-Difluoro-3,5-dimethoxybenzyl)oxy)-3,4,6-trimethylpyridin-2-yl)amino)-4-(4-ethylpiperazin-1-yl)phenyl)acrylamide (6Y)**

To a solution of **23Y** (40 mg, 0.08 mmol) in DCM (2 mL) were added Et_3N (58 µL, 0.42 mmol) and acryloyl chloride (8 µL, 0.1 mmol) dropwise at 0 °C. The resulting mixture was stirred at room temperature for 30 min, and diluted with DCM and saturated $NaHCO_3$. The aqueous layer was extracted with DCM and the combined organic solution was dried over $MgSO_4$, filtered, and concentrated. The residue was purified by flash silica gel column chromatography with 10% MeOH/DCM to obtain **6Y** (7 mg, 12%, for two steps). White solid; TLC R_f 0.43 (DCM/MeOH = 9/1); m.p. 200 °C; MS (EI) *m*/*z* 595 $[M]^+$; ^1H NMR ($CDCl_3$) δ 8.93 (s, 1H), 7.33 (d, *J* = 8.8 Hz, 1H), 6.91 (d, *J* = 2.4 Hz, 1H), 6.66 (dt, *J* = 8.0, 5.3 Hz, 2H), 6.43 (s, 1H), 6.27 (dd, *J* = 16.9, 1.6 Hz, 1H), 6.13 (dd, *J* = 16.9, 9.9 Hz, 1H), 5.63 (dd, *J* = 9.9, 1.8 Hz, 1H), 4.84 (s, 2H), 3.89 (s, 6H), 3.34–3.10 (m, 4H), 2.69–2.53 (m, 4H), 2.47 (dd, *J* = 14.6, 7.4 Hz, 1H), 2.40 (s, 1H), 2.29 (s, 3H), 2.13 (s, 3H), 1.12 (t, *J* = 7.2 Hz, 3H); ^{13}C NMR ($CDCl_3$) δ 164.1, 150.4, 149.7, 147.3 (d, *J* = 6.0 Hz), 146.2, 145.4, 143.8 (d, *J* = 4.8 Hz), 143.6 (d, *J* = 5.0 Hz), 143.5 (d, *J* = 5.9 Hz), 141.1, 135.6 131.9 (2C), 126.4, 125.9, 122.6, 115.9, 114.3 (t, *J* = 18.2 Hz), 112.1, 110.7, 102.1, 62.4, 57.6 (2C), 53.0 (2C), 52.5, 49.4 (2C), 19.0, 13.4, 12.9, 12.1; HRMS (EI) *m*/*z* calculated for $C_{32}H_{39}F_2N_5O_4$ $[M]^+$ 595.2970, found 595.2975.

***N*-(2-((5-((2,6-Dichloro-3,5-dimethoxybenzyl)oxy)-3,4,6-trimethylpyridin-2-yl)amino)-4-(4-ethylpiperazin-1-yl)phenyl)acrylamide (6Z)**

To a solution of **23Z** (40 mg, 0.07 mmol) in DCM (1 mL) was added Et_3N (58 µL, 0.42 mmol) and acryloyl chloride (6.7 µL, 0.08 mmol) dropwise at 0 °C. The resulting mixture was stirred at room temperature for 30 min, and diluted with DCM and saturated $NaHCO_3$. The aqueous layer was extracted with DCM and the combined organic solution was dried over $MgSO_4$, filtered, and concentrated. The residue was purified by flash silica gel column chromatography with 40% to 50% EtOAc/Hexanes to obtain **6Z** (12 mg, 22%, for two steps). Pale yellow solid; TLC R_f 0.30 (DCM/MeOH = 9/1); m.p. 180 °C; MS (EI) *m*/*z* 627 $[M]^+$; ^1H NMR ($CDCl_3$) δ 9.04 (s, 1H), 7.29 (s, 1H), 6.88 (d, *J* = 2.4 Hz, 1H), 6.66–6.56 (m, 2H), 6.45 (s, 1H), 6.32–6.20 (m, 1H), 6.13 (t, *J* = 13.3 Hz, 1H), 5.62 (dd, *J* = 9.8, 1.8 Hz, 1H), 5.17 (s, 2H), 3.92 (s, 6H), 3.20 (s, 4H), 2.62 (s, 4H), 2.50 (q, *J* = 7.2 Hz, 2H), 2.35 (s, 3H), 2.24 (s, 3H), 2.10 (s, 3H), 1.14 (t, *J* = 7.2 Hz, 3H); ^{13}C NMR ($CDCl_3$) δ 164.1, 154.7 (2C), 150.1, 149.4, 146.2, 146.1, 141.3,

135.8, 134.3, 131.9, 126.4, 125.9, 122.7, 116.8, 116.0 (2C), 112.1, 110.6, 98.0, 69.9, 56.8 (2C), 52.8 (2C), 52.5 (2C), 49.1, 19.6, 13.6, 13.5, 11.8; HRMS (EI) *m*/*z* calculated for $C_{32}H_{39}Cl_2N_5O_4$ $[M]^+$ 627.2379, found 627.2370.

_N_-(2-((5-((2,6-Difluoro-3,5-dimethoxybenzyl)oxy)-4,6-dimethylpyrimidin-2-yl)amino)-4-(4-ethylpiperazin-1-yl)phenyl)acrylamide (6AA)

To a solution of **23AA** (40 mg, 0.08 mmol) in DCM (1 mL) was added Et_3N (58 µL, 0.42 mmol) and acryloyl chloride (20 µL, 0.25 mmol) dropwise at 0 °C. The resulting mixture was stirred at room temperature for 30 min, and diluted with DCM and saturated $NaHCO_3$. The aqueous layer was extracted with DCM and the combined organic solution was dried over $MgSO_4$, filtered, and concentrated. The residue was purified by flash silica gel column chromatography with 10% MeOH/DCM to obtain **6AA** (12 mg, 28%). White solid; TLC R_f 0.26 (DCM/MeOH = 9/1); m.p. 238 °C; MS (EI) *m*/*z* 582 $[M]^+$; ^1H NMR ($CDCl_3$) δ 8.70 (s, 1H), 7.65 (d, *J* = 8.8 Hz, 1H), 7.02 (d, *J* = 2.3 Hz, 1H), 6.91 (s, 1H), 6.77 (dd, *J* = 8.8, 2.5 Hz, 1H), 6.69 (t, *J* = 8.2 Hz, 1H), 6.32 (d, *J* = 16.9 Hz, 1H), 6.13 (dd, *J* = 16.9, 10.0 Hz, 1H), 5.66 (d, *J* = 10.0 Hz, 1H), 4.89 (s, 2H), 3.89 (s, 6H), 3.23 (s, 4H), 2.63 (s, 4H), 2.51 (dd, *J* = 14.4, 7.2 Hz, 1H), 2.39 (s, 6H), 1.15 (t, *J* = 7.2 Hz, 3H); ^{13}C NMR ($CDCl_3$) δ 164.0, 161.9 (2C), 156.4, 149.5, 147.2 (d, *J* = 5.6 Hz), 143.8 (d, *J* = 5.0 Hz), 143.7 (d, *J* = 4.9 Hz), 143.5, 143.3, 142.0, 133.2, 131.9, 126.5 (d, *J* = 16.7 Hz), 123.0, 113.8 (t, *J* = 17.8 Hz), 113.3, 111.0, 102.2, 62.5, 57.5 (2C), 52.8 (2C), 52.5 (2C), 49.1, 19.0 (2C), 12.0; HRMS (EI) *m*/*z* calculated for $C_{30}H_{36}F_2N_6O_4$ $[M]^+$ 582.2766, found 582.2772.

_N_-(2-((5-((2,6-Dichloro-3,5-dimethoxybenzyl)oxy)-4,6-dimethylpyrimidin-2-yl)amino)-4-(4-ethylpiperazin-1-yl)phenyl)acrylamide (6BB)

To a solution of **23BB** (20 mg, 0.04 mmol) in DCM (1 mL) was added Et_3N (18 µL, 0.13 mmol) and acryloyl chloride (10.0 µL, 0.12 mmol) dropwise at 0 °C. The resulting mixture was stirred at room temperature for 30 min, and diluted with DCM and saturated $NaHCO_3$. The aqueous layer was extracted with DCM and the combined organic solution was dried over $MgSO_4$, filtered, and concentrated. The residue was dissolved in a small amount of EtOAc then hexanes was added to get a precipitate. The precipitate was filtered, washed with hexane to obtain **6BB** (6 mg, 27%). Grey solid; TLC R_f 0.39 (DCM/MeOH = 9/1); m.p. 250 °C; MS (EI) *m*/*z* 614 $[M]^+$; ^1H NMR ($CDCl_3$) δ 8.74 (s, 1H), 7.66 (d, *J* = 8.8 Hz, 1H), 7.00 (d, *J* = 2.3 Hz, 1H), 6.86

(s, 1H), 6.77 (dd, J = 8.8, 2.5 Hz, 1H), 6.62 (s, 1H), 6.31 (d, J = 15.6 Hz, 1H), 6.13 (dd, J = 16.9, 10.1 Hz, 1H), 5.72–5.58 (m, 1H), 5.19 (s, 2H), 3.94 (s, 6H), 3.26 (s, 4H), 2.67 (s, 4H), 2.55 (d, J = 6.8 Hz, 2H), 2.36 (s, 6H), 1.17 (t, J = 7.1 Hz, 3H); ^{13}C NMR ($CDCl_3$) δ 164.0, 161.8 (2C), 156.2, 154.8 (2C), 149.3, 144.0, 133.6, 133.2, 132.0, 126.6, 126.4 123.2, 116.7 (2C), 113.3, 111.1, 98.1, 69.9, 56.8 (2C), 52.7 (2C), 52.5 (2C), 49.0, 19.5 (2C), 11.8; HRMS (EI) m/z calculated for $C_{30}H_{36}Cl_2N_6O_4$ $[M]^+$ 614.2175, found 614.2174.

3-(Bromomethyl)-2,4-difluoro-1,5-dimethoxybenzene (7) [CAS RN: 1956324-83-6]

To a solution of (2,6-difluoro-3,5-dimethoxyphenyl)methanol (**22**) (3.0 g, 14.69 mmol) in DCM (15 mL) was added PBr_3 (1 M in DCM, 15.7 mL, 15.72 mmol) dropwise at 0 °C. The reaction mixture was stirred at room temperature for 30 minutes and quenched with ice water. After extraction of the mixture with DCM, the combined organic solution was dried over $MgSO_4$, filtered, concentrated to afford **7** (3.75 g, 100%) as a grey solid.

3-(Bromomethyl)-2,4-dichloro-1,5-dimethoxybenzene (8) [CAS RN: 1589080-79-4]

1-(Bromomethyl)-3,5-dimethoxybenzene (**25**) was dissolved in acetonitrile (10 mL) then added a solution of sulfuryl chloride (1 M in DCM, 31 mL) dropwise at 0 °C and stirred at room temperature for 24 h. Additional amount of sulfuryl chloride (10 mL×3) was added in 6 h interval three times. The reaction mixture was quenched with ice water after 42 h then extracted with DCM. The combined organic solution was dried over $MgSO_4$, filtered, and concentrated. The residue was purified by flash silica gel column chromatography with 10% EtOAc/Hexanes to obtain **8** (4.2 g, 90%) as a white solid.

6-Bromo-2,4,5-trimethylpyridin-3-ol (9) [CAS RN: 1444336-68-8]

To a suspension of 2,4,5-trimethylpyridin-3-ol (**28**) (3.6 g, 26.24 mmol) in THF (30 mL) was added DBDMH (3.7 g, 12.94 mmol) and the resulting mixture was stirred for 3 h at room temperature. The mixture was concentrated, and the residue was diluted with EtOAc and water. The aqueous layer was extracted with EtOAc and the combined organic layer was dried over $MgSO_4$, filtered, and concentrated. The residue was purified by silica gel column chromatography with 15% to 25% EtOAc/Hexanes to obtain **9** (4.5 g, 78% yield) as a yellow solid.

2-Bromo-4,6-dimethylpyrimidin-5-ol (10)

To a solution of 4,6-dimethylpyrimidin-5-ol (**31**) (150 mg, 1.21 mmol) in THF (4 mL) was added DBDMH (242 mg, 0.85 mmol). The resulting mixture was stirred at room temperature for 5 h then concentrated. The residue was diluted with EtOAc and water followed by EtOAc extraction. The combined organic solution was dried over $MgSO_4$, filtered, and concentrated. The residue was purified by flash silica gel column chromatography with 10% to 40% EtOAc/Hexanes to obtain **10** (156 mg, 63%). Brown solid; TLC R_f 0.55 (EtOAc/Hexanes = 1/1); m.p. 170 °C; MS (FAB) *m*/*z* 202 $[M+H]^+$; ^{1}H NMR ($CDCl_3$) δ 2.47 (s, 6H); ^{13}C NMR ($CDCl_3$) δ 156.4 (2C), 147.2, 141.3, 18.7 (2C); HRMS (FAB) *m*/*z* calculated for $C_6H_8BrN_2O$ $[M+H]^+$ 201.9742, found 201.9739.

2-Bromo-5-((2,6-difluoro-3,5-dimethoxybenzyl)oxy)-3,4,6-trimethylpyridine (11)

To a solution of 6-bromo-2,4,5-trimethylpyridin-3-ol (**9**) (1.0 g, 4.63 mmol) in DMF (10 mL) were added 3-(bromomethyl)-2,4-difluoro-1,5-dimethoxybenzene (**7**) (1.4 g, 5.24 mmol) and K_2CO_3 (3.2 g, 23.15 mmol). The resulting mixture was stirred at 80 °C for 24 h and then cooled to room temperature. After addition of ice water, the mixture was stirred at 0 °C for 15 min. The precipitate formed in the reaction mixture was filtered, the filter cake was washed with ice water to **11** (1.2 g, 71%). White solid; TLC R_f 0.55 (EtOAc/Hexanes = 1/2); m.p. 181 °C; MS (EI) *m*/*z* 401 $[M]^+$; ^{1}H NMR ($CDCl_3$) δ 6.69 (t, *J* = 8.2 Hz, 1H), 4.86 (t, *J* = 1.9 Hz, 2H), 3.89 (s, 6H), 2.48 (s, 3H), 2.32 (s, 3H), 2.30 (s, 3H); ^{13}C NMR ($CDCl_3$) δ 151.1, 150.7, 147.2 (d, *J* = 5.8 Hz), 143.8 (d, *J* = 4.9 Hz), 143.6 (d, *J* = 5.0 Hz), 143.3 (d, *J* = 5.8 Hz), 142.0 (2C), 138.5, 132.2, 113.8 (t, *J* = 18.0 Hz), 102.2, 62.2, 57.5 (2C), 18.9 (2C), 13.7; HRMS (EI) *m*/*z* calculated for $C_{17}H_{18}BrF_2NO_3$ $[M]^+$ 401.0438, found 401.0432.

2-Bromo-5-((2,6-dichloro-3,5-dimethoxybenzyl)oxy)-3,4,6-trimethylpyridine (12)

To a solution of 6-bromo-2,4,5-trimethylpyridin-3-ol (**9**) (1.0 g, 4.63 mmol) in DMF (10.0 ml) were added 3-(bromomethyl)-2,4-dichloro-1,5-dimethoxybenzene (**8**) (1.5 g, 5.0 mmol) and K_2CO_3 (3.2 g, 23.15 mmol). The resulting mixture was stirred at 80 °C for 24 h, and then cooled to room temperature. After addition of ice water, the mixture was stirred at 0 °C for 15 min. The precipitate formed in the reaction mixture was filtered, and the filter cake was washed with ice water to obtain **12** (1.7 g, 84%). White solid; TLC R_f 0.29 (EtOAc/Hexanes = 1/4); m.p. 185 °C; MS (EI) *m*/*z* 435 $[M]^+$; ^{1}H NMR ($CDCl_3$) δ 6.60 (s, 1H), 5.18 (s, 2H), 3.93 (s, 6H), 2.45 (s, 3H), 2.29 (s, 3H), 2.24 (s, 3H); ^{13}C NMR ($CDCl_3$) δ 154.7 (2C), 151.6, 150.6, 142.0,

138.2, 133.6, 131.9, 116.5, 97.9, 69.7, 56.7, 19.7, 19.0, 14.3; HRMS (EI) *m*/*z* calculated for $C_{17}H_{18}BrCl_2NO_3$ $[M]^+$ 432.9847, found 432.9846.

2-Bromo-5-((2,6-difluoro-3,5-dimethoxybenzyl)oxy)-4,6-dimethylpyrimidine (13)

To a solution of 2-bromo-4,6-dimethylpyrimidin-5-ol (**10**) (90 mg, 0.44 mmol) in DMF (3 mL) were added 3-(bromomethyl)-2,4-difluoro-1,5-dimethoxybenzene (**7**) (94 mg, 0.35 mmol) and K_2CO_3 (304.1 mg, 2.20 mmol). The resulting mixture was stirred at 80 °C for 12 h, and then cooled to room temperature. After addition of ice water, the mixture was stirred at 0 °C for 15 min. The precipitate formed in the reaction mixture was filtered, and the filter cake was washed with ice water to obtain **13** (114 mg, 67%). White solid; TLC R_f 0.63 (EtOAc/Hexanes = 1/2); m.p. 145 °C; MS (EI) *m*/*z* 388 $[M]^+$; ^1H NMR ($CDCl_3$) δ 6.70 (t, *J* = 8.2 Hz, 1H), 4.96 (t, *J* = 1.8 Hz, 2H), 3.89 (s, 6H), 2.47 (s, 6H); ^{13}C NMR ($CDCl_3$) δ 164.2 (2C), 149.5, 147.0 (d, *J* = 5.7 Hz), 145.7, 143.9 (d, *J* = 5.0 Hz), 143.7 (d, *J* = 5.0 Hz), 143.1 (d, *J* = 5.6 Hz), 113.1, 102.4, 62.5, 57.5 (2C), 18.9 (2C); HRMS (EI) *m*/*z* calculated for $C_{15}H_{15}BrF_2N_2O_3$ $[M]^+$ 388.0234, found 388.0239.

2-Bromo-5-((2,6-dichloro-3,5-dimethoxybenzyl)oxy)-4,6-dimethylpyrimidine (14)

To a solution of 2-bromo-4,6-dimethylpyrimidin-5-ol (**10**) (50 mg, 0.25 mmol) in DMF (2 mL) were added 3-(bromomethyl)-2,4-dichloro-1,5-dimethoxybenzene (**8**) (59 mg, 0.20 mmol) and K_2CO_3 (170 mg, 1.23 mmol). The resulting mixture was stirred at 80 °C for 12 h, and then cooled to room temperature. After addition of ice water, the mixture was stirred at 0 °C for 15 min. The precipitate formed in the reaction mixture was filtered, and the filter cake was washed with ice water to obtain **14** (62 mg, 75%). White solid; TLC R_f 0.55 (EtOAc/Hexanes = 1/1); m.p. 220 °C; MS (EI) *m*/*z* 422 $[M]^+$; ^1H NMR ($CDCl_3$) δ 6.62 (s, 1H), 5.26 (s, 2H), 3.94 (s, 6H), 2.44 (s, 6H); ^{13}C NMR ($CDCl_3$) δ 164.1 (2C), 154.8 (2C), 149.9, 145.3, 132.9, 116.6 (2C), 98.3, 69.8, 56.8 (2C), 19.4 (2C); HRMS (EI) *m*/*z* calculated for $C_{15}H_{15}BrCl_2N_2O_3$ $[M]^+$ 419.9643, found 419.9637.

1-Methyl-4-nitro-1*H*-pyrazol-3-amine (15) [CAS RN: 20055-00-9]

To a mixture of *N*-(1-methyl-1*H*-pyrazol-3-yl)acetamide hydrochloride (**33**) (2.0 g, 11.40 mmol) in concentrated H_2SO_4 (7.7 mL, 144.45 mmol) was added fuming HNO_3 dropwise at 0 °C and then stirred for 2.5 h. The reaction mixture was poured into ice water and stirred at

room temperature for 12 h. The reaction mixture was then neutralized using a solution of 6 M NaOH and saturated $NaHCO_3$ followed by EtOAc extraction. The combined organic layer was $MgSO_4$ dried, filtered, and concentrated. The residue was purified by flash silica gel column chromatography with 10% to 50% EtOAc/Hexanes to obtain **15** (823 mg, 51%) as a yellow solid.

2-Methyl-6-nitroaniline (16) [CAS RN: 570-24-1] and 2-methyl-4-nitroaniline (17) [CAS RN: 99-52-5]

A solution of *o*-toluidine (**34**) (2 mL, 18.81 mmol) in acetic anhydride (13 mL, 137.53 mmol) was stirred at 0 °C for 2 h, and then 65% HNO_3 (2.6 mL, 38.34 mmol) was added dropwise for above 1 h. The resulting mixture was continuously stirred at 0 °C for additional 4 h. Subsequently, the reaction mixture was poured into ice water and stirred overnight at room temperature. The precipitate formed was filtered, washed with ice water, and collected. To the crude solid was added 6 M HCl in excess amount then refluxed for 4 h. The resulting mixture was neutralized using 6 M NaOH solution then basified using saturated $NaHCO_3$ followed by EtOAc extraction. The combined organic solution was $MgSO_4$ dried, filtered, and concentrated. The residue was purified by flash silica gel column chromatography with 5% to 15% EtOAc/Hexanes to obtain **16** (1.0 g, 35%, for two steps) and **17** (665 mg, 23%, for two steps) respectively as a yellow solid.

2-Chloro-4-fluoro-6-nitroaniline (18) [CAS RN: 153505-32-9]

To a mixture of *N*-(2-chloro-4-fluorophenyl)acetamide (**36**) (1.0 g, 5.33 mmol) in acetic acid (2 mL) and concentrated H_2SO_4 (10 mL, 187.60 mmol) was added fuming HNO_3 (334.0 µL, 8.0 mmol) dropwise at 0 °C, and then stirred for 3 h. The reaction mixture was poured into ice water and stirred at room temperature for 12 h. The reaction mixture was then neutralized using a solution of 6 M NaOH followed by EtOAc extraction. The combined organic solution was $MgSO_4$ dried, filtered, and concentrated. To the residue was added an excessive amount of 1 M HCl then refluxed for 5 h. The reaction mixture was then neutralized using 6 M NaOH followed by EtOAc extraction. The combined organic solution was $MgSO_4$ dried, filtered, and concentrated. The residue was purified by silica gel column chromatography with 1% to 5% EtOAc/Hexanes to obtain **18** (596 mg, 59%) as a yellow solid.

2-Methoxy-6-nitroaniline (19) [CAS RN: 16554-45-3]

To a solution of 2-amino-3-nitrophenol (**37**) (1.0 g, 6.49 mmol) in acetone (10 mL) were added K_2CO_3 (1.1 g, 7.96 mmol) and MeI (496 µL, 7.96 mmol). The resulting mixture was stirred at room temperature for 20 h then concentrated. To the residue was added EtOAc then washed with water. The organic layer was dried over $MgSO_4$, filtered, and concentrated to obtain **19** (1.04 g, 95%) as an orange solid.

5-Methoxy-2-nitroaniline (20) [CAS RN: 16133-49-6]

To a solution of *N*-(3-methoxyphenyl)acetamide (**39**) (650 mg, 3.94 mmol) in acetic acid (5 mL) was added 65% HNO_3 (2.6 mL, 38.34 mmol) dropwise at 0 °C. The resulting mixture was stirred for 1.5 h at the same temperature, and then poured into ice water then stirred at room temperature overnight. The precipitate formed in the reaction mixture was filtered, washed with ice water, and collected. To the residue was added 6 M HCl in excess amount then refluxed for 3 h. The reaction mixture was neutralized using 6 M NaOH solution then basified using a saturated solution of $NaHCO_3$ followed by EtOAc extraction. The combined organic solution was $MgSO_4$ dried, filtered, and concentrated. The residue was purified by flash silica gel column chromatography with 20% EtOAc/Hexanes to obtain **20** (89 mg, 13%, for two steps) as an orange solid.

5-(4-Ethylpiperazin-1-yl)-2-nitroaniline (21) [CAS RN: 23470-40-8]

To a solution of 5-fluoro-2-nitroaniline (**40**) (100 mg, 0.64 mmol) in *N*-methylpyrrolidine (**23**) (3 mL) were added 1-ethylpiperazine (163 µL, 1.28 mmol) and DIPEA (279 µL, 1.60 mmol). The resulting mixture was stirred at 130 °C for 5 h and then poured into ice water. The precipitate formed was filtered, and the filter cake washed with ice water to obtain **21** (154 mg, 96%) as a yellow solid.

5-((2,6-Difluoro-3,5-dimethoxybenzyl)oxy)-3,4,6-trimethyl-*N*-(1-methyl-4-nitro-1*H*-pyrazol-3-yl)pyridin-2-amine (22A)

To a mixture of **11** (50 mg, 0.12 mmol) and **15** (21 mg, 0.15 mmol) in toluene (3 mL) were added NaO^{t}Bu (24 mg, 0.25 mmol), BINAP (1.5 mg, 0.003 mmol), and $Pd_2(dba)_3$ (1.3 mg, 0.001 mmol). The resulting mixture was refluxed for 12 h, and then diluted with EtOAc. The organic layer was washed with brine, dried over $MgSO_4$, filtered, and concentrated. The residue

was purified by silica gel column chromatography with 5% to 15% EtOAc/DCM to obtain **22A** (27 mg, 47%). Yellow solid; TLC R_f 0.20 (EtOAc/Hexanes = 1/1, developed twice); m.p. 215 °C; MS (EI) *m/z* 463 $[M]^+$; ^{1}H NMR (DMSO-d_6) δ 8.66 (s, 1H), 8.32 (s, 1H), 7.08 (s, 1H), 4.80 (s, 2H), 3.88 (s, 6H), 3.76 (s, 3H), 2.21 (s, 3H), 2.20 (s, 3H), 2.11 (s, 3H); ^{13}C NMR (DMSO-d_6) δ 147.1 (2C), 146.4, 146.0, 145.6 (d, J = 6.0 Hz), 143.4 (d, J = 4.9 Hz), 143.2 (d, J = 4.9 Hz), 141.7 (d, J = 6.0 Hz), 140.1 (2C), 131.3, 123.4, 120.9, 113.4 (t, J = 18.0 Hz), 101.6, 61.8, 56.9 (2C), 18.5, 13.8, 12.3; HRMS (EI) *m/z* calculated for $C_{21}H_{23}F_2N_5O_5$ $[M]^+$ 463.1667, found 463.1668.

5-((2,6-Dichloro-3,5-dimethoxybenzyl)oxy)-3,4,6-trimethyl-*N*-(1-methyl-4-nitro-1*H*-pyrazol-3-yl)pyridin-2-amine (22B)

To a mixture of **12** (200 mg, 0.46 mmol) and **15** (65 mg, 0.46 mmol) in toluene (5 mL) were added NaO^{t}Bu (88 mg, 0.92 mmol), BINAP (11 mg, 0.02 mmol), and $Pd_2(dba)_3$ (9.5 mg, 0.01 mmol). The resulting mixture was refluxed for 24 h, and then diluted with EtOAc. The organic layer was washed with brine, dried over $MgSO_4$, filtered, and concentrated. The residue was purified by silica gel column chromatography with 0% to 10% EtOAc/DCM with 3% MeOH additive later to obtain **22B** (143 mg, 63%). Yellow solid; TLC R_f 0.17 (EtOAc/Hexanes = 1/1); m.p. 230 °C; MS (EI) *m/z* 495 $[M]^+$; ^{1}H NMR ($CDCl_3$) δ 8.44 (s, 1H), 8.02 (s, 1H), 6.61 (s, 1H), 5.18 (s, 2H), 3.93 (s, 6H), 3.87 (s, 3H), 2.53 (s, 3H), 2.28 (s, 3H), 2.21 (s, 3H); ^{13}C NMR ($CDCl_3$) δ 154.7 (2C), 148.4, 148.0, 147.0, 145.9, 141.3, 134.1, 129.4, 122.6, 119.7, 116.8 (2C), 98.0, 69.7, 56.8 (2C), 40.5, 20.1, 13.9, 13.7; HRMS (EI) *m/z* calculated for $C_{21}H_{23}Cl_2N_5O_5$ $[M]^+$ 495.1076, found 495.1077.

5-((2,6-Difluoro-3,5-dimethoxybenzyl)oxy)-4,6-dimethyl-*N*-(1-methyl-4-nitro-1*H*-pyrazol-3-yl)pyrimidin-2-amine (22C)

To a mixture of **13** (200 mg, 0.51 mmol) and **15** (73 mg, 0.51 mmol) in toluene (5 mL) were added NaO^{t}Bu (147 mg, 0.53 mmol), BINAP (13 mg, 0.02 mmol), and $Pd_2(dba)_3$ (11 mg, 0.01 mmol). The resulting mixture was refluxed for 5 h, and then diluted with EtOAc. The organic layer was washed with brine, dried over $MgSO_4$, filtered, and concentrated. The residue was purified by silica gel column chromatography with 2% to 4% EtOAc/DCM with 1% MeOH additive later to obtain **22C** (104 mg, 45%). Yellow solid; TLC R_f 0.32 (EtOAc/Hexanes = 1/2); m.p. 229 °C; MS (EI) *m/z* 450 $[M]^+$; H NMR ($CDCl_3$) δ 8.90 (s, 1H), 8.06 (s, 1H), 6.69 (t, J =

8.2 Hz, 1H), 4.91 (t, J = 1.8 Hz, 2H), 3.93 (s, 3H), 3.89 (s, 6H), 2.49 (s, 6H); ^{13}C NMR ($CDCl_3$) δ 162.2 (2C), 153.2, 147.1 (d, J = 5.8 Hz), 145.0, 144.7, 143.8 (d, J = 5.0 Hz), 143.6 (d, J = 5.0 Hz), 143.2 (d, J = 6.0 Hz), 129.7, 123.0, 113.7 (t, J = 18.0 Hz), 102.2, 62.4, 57.5 (2C), 40.6, 19.2 (2C); HRMS (EI) *m/z* calculated for $C_{19}H_{20}F_2N_6O_5$ $[M]^+$ 450.1463, found 450.1457.

5-((2,6-Dichloro-3,5-dimethoxybenzyl)oxy)-4,6-dimethyl-*N*-(1-methyl-4-nitro-1*H*-pyrazol-3-yl)pyrimidin-2-amine (22D)

To a mixture of **14** (100 mg, 0.24 mmol) and **15** (40 mg, 0.28 mmol) in toluene (3 mL) were added Cs_2CO_3 (232 mg, 0.71 mmol), Xantphos (41 mg, 0.07 mmol), and $Pd_2(dba)_3$ (25 mg, 0.02 mmol). The resulting mixture was refluxed for 12 h, and then diluted with EtOAc. The organic layer was washed with brine, dried over $MgSO_4$, filtered, and concentrated. The residue was dissolved in a small amount of $CHCl_3$ then hexanes was added to get a precipitate. The precipitate was filtered, washed with hexane to obtain **22D** (75 mg, 65%). Yellow solid; TLC R_f 0.39 (EtOAc); m.p. 260 °C; MS (EI) *m/z* 482 $[M]^+$; ^{1}H NMR ($CDCl_3$) δ 8.91 (s, 1H), 8.05 (s, 1H), 6.60 (s, 1H), 5.22 (s, 2H), 3.92 (s, 9H), 2.45 (s, 6H); ^{13}C NMR ($CDCl_3$) δ 162.1 (2C), 154.8 (2C), 153.0, 145.4, 144.7, 133.5, 129.7, 122.9, 121.0, 116.7 (2C), 98.1, 69.7, 56.8 (2C), 40.6, 19.7 (2C); HRMS (EI) *m/z* calculated for $C_{19}H_{20}Cl_2N_6O_5$ $[M]^+$ 482.0872, found 482.0879.

5-((2,6-Difluoro-3,5-dimethoxybenzyl)oxy)-3,4,6-trimethyl-*N*-(2-methyl-6-nitrophenyl)pyridin-2-amine (22E)

To a mixture of **11** (50 mg, 0.12 mmol) and **16** (21 mg, 0.14 mmol) in toluene (2 mL) were added Cs_2CO_3 (121 mg, 0.37 mmol), BINAP (3 mg, 0.005 mmol), and $Pd_2(dba)_3$ (2.5 mg, 0.003 mmol). The resulting mixture was refluxed for 24 h, and then diluted with EtOAc. The organic layer was washed with brine, dried over $MgSO_4$, filtered, and concentrated. The residue was purified by silica gel column chromatography with 20% to 40% DCM/Hexanes along with 5% EtOAc additive to obtain **22E** (42 mg, 72%). Orange solid; TLC R_f 0.50 (EtOAc/Hexanes = 1/2); m.p. 185 °C; MS (EI) *m/z* 473 $[M]^+$; ^{1}H NMR ($CDCl_3$) δ 8.37 (s, 1H), 7.92 (d, J = 8.3 Hz, 1H), 7.41 (d, J = 7.3 Hz, 1H), 7.05 (t, J = 7.9 Hz, 1H), 6.68 (t, J = 8.1 Hz, 1H), 4.82 (s, 2H), 3.89 (s, 6H), 2.30 (s, 6H), 2.26 (s, 3H), 2.08 (s, 3H); ^{13}C NMR ($CDCl_3$) δ 148.5, 147.4 (d, J = 5.6 Hz), 146.7, 143.8 (d, J = 5.0 Hz), 143.6 (d, J = 4.8 Hz), 142.3, 140.6 (d, J = 1.8 Hz), 137.2, 136.5 (2C), 135.8, 123.4 (2C), 122.0, 118.0, 114.3 (t, J = 17.9 Hz), 102.1, 62.2, 57.6 (2C), 20.4, 18.9, 13.5, 12.8; HRMS (EI) *m/z* calculated for $C_{24}H_{25}F_2N_3O_5$ $[M]^+$ 473.1762, found 473.1765.

5-((2,6-Dichloro-3,5-dimethoxybenzyl)oxy)-3,4,6-trimethyl-*N*-(2-methyl-6-nitrophenyl)pyridin-2-amine (22F)

To a mixture of **12** (200 mg, 0.46 mmol) and **16** (70 mg, 0.46 mmol) in toluene (5 mL) were added Cs_2CO_3 (450 mg, 1.38 mmol), Xantphos (80 mg, 0.14 mmol), and $Pd_2(dba)_3$ (24 mg, 0.02 mmol). The resulting mixture was refluxed for 72 h, and then diluted with EtOAc. The organic layer was washed with brine, dried over $MgSO_4$, filtered, and concentrated. The residue was purified by silica gel column chromatography with 30% to 50% EtOAc/Hexanes to obtain **22F** (101 mg, 43%). Orange solid; TLC R_f 0.39 (EtOAc/Hexanes = 1/2); m.p. 233 °C; MS (EI) *m/z* 505 $[M]^+$; ^1H NMR ($CDCl_3$) δ 8.46 (s, 1H), 7.93 (dd, *J* = 8.3, 1.4 Hz, 1H), 7.40 (d, *J* = 7.4 Hz, 1H), 7.03 (t, 1H), 6.60 (s, 1H), 5.15 (s, 2H), 3.93 (s, 6H), 2.27 (s, 3H), 2.25 (s, 6H), 2.04 (s, 3H); ^{13}C NMR ($CDCl_3$) δ 154.7 (2C), 148.2, 147.4, 146.7, 142.1, 140.6, 137.5, 136.5, 135.6, 134.3, 123.4, 121.8, 118.1, 116.8 (2C), 98.0, 69.8, 56.8 (2C), 20.4, 19.6, 13.5, 13.4; HRMS (EI) *m/z* calculated for $C_{24}H_{25}Cl_2N_3O_5^+$ $[M]^+$ 505.1171, found 505.1175.

5-((2,6-Difluoro-3,5-dimethoxybenzyl)oxy)-4,6-dimethyl-*N*-(2-methyl-6-nitrophenyl)pyrimidin-2-amine (22G)

To a mixture of **13** (150 mg, 0.39 mmol) and **16** (64 mg, 0.42 mmol) in toluene (3 mL) were added Cs_2CO_3 (376 mg, 1.16 mmol), Xantphos (67 mg, 0.12 mmol), and $Pd_2(dba)_3$ (40 mg, 0.04 mmol). The resulting mixture was refluxed for 33 h, and then diluted with DCM. The organic layer was washed with brine, dried over $MgSO_4$, filtered, and concentrated. The residue was purified by silica gel column chromatography with 15% to 25% EtOAc/Hexanes to obtain **22G** (113 mg, 64%). Yellow solid; TLC R_f 0.26 (Hexanes/EtOAc = 2/1); m.p. 220 °C; MS (EI) *m/z* 460 $[M]^+$; ^1H NMR ($CDCl_3$) δ 7.82 (d, *J* = 8.2 Hz, 1H), 7.75 (s, 1H), 7.47 (d, *J* = 7.5 Hz, 1H), 7.17 (t, *J* = 7.9 Hz, 1H), 6.68 (t, *J* = 8.1 Hz, 1H), 4.85 (s, 2H), 3.88 (s, 6H), 2.34 (s, 6H), 2.30 (s, 3H); ^{13}C NMR ($CDCl_3$) δ 161.7 (2C), 154.9, 147.2 (d, *J* = 5.9 Hz), 144.6, 144.1, 143.8 (d, *J* = 4.9 Hz), 143.6 (d, *J* = 4.9 Hz), 143.3 (d, *J* = 5.8 Hz), 136.6 135.6, 132.5, 124.2, 123.1, 113.8 (t, *J* = 18.0 Hz), 102.3, 62.4, 57.5 (2C), 19.4, 18.7 (2C).

5-((2,6-Dichloro-3,5-dimethoxybenzyl)oxy)-4,6-dimethyl-*N*-(2-methyl-6-nitrophenyl)pyrimidin-2-amine (22H)

To a mixture of **14** (150 mg, 0.36 mmol) and **16** (59 mg, 0.39 mmol) in toluene (3 mL) were added Cs_2CO_3 (347 mg, 1.07 mmol), Xantphos (62 mg, 0.11 mmol), and $Pd_2(dba)_3$ (37 mg, 0.04 mmol). The resulting mixture was refluxed for 12 h, and then diluted with DCM. The organic layer was washed with brine, dried over $MgSO_4$, filtered, and concentrated. The residue was purified by silica gel column chromatography with 30% to 50% EtOAc/Hexanes to obtain **22H** (103 mg, 59%). Yellow solid; TLC R_f 0.23 (Hexanes/EtOAc = 2/1); m.p. 209 °C; MS (EI) *m*/*z* 492 $[M]^+$; ^{1}H NMR ($CDCl_3$) δ 7.82 (dd, *J* = 8.2, 1.2 Hz, 1H), 7.77 (s, 1H), 7.46 (d, *J* = 7.3 Hz, 1H), 7.16 (t, *J* = 7.9 Hz, 1H), 6.61 (s, 1H), 5.17 (s, 2H), 3.93 (s, 6H), 2.31 (s, 6H), 2.29 (s, 3H); ^{13}C NMR ($CDCl_3$) δ 161.6, 154.8 (2C), 154.7 (2C), 144.5, 136.5, 135.7, 133.6, 132.7, 124.1, 123.1 (2C), 116.8 (2C), 98.2, 69.8, 56.8 (2C), 19.5, 19.3 (2C); HRMS (EI) *m*/*z* calculated for $C_{22}H_{22}Cl_2N_4O_5$ $[M]^+$ 492.0967, found 492.0962.

5-((2,6-Difluoro-3,5-dimethoxybenzyl)oxy)-3,4,6-trimethyl-*N*-(2-methyl-4-nitrophenyl)pyridin-2-amine (22I)

To a mixture of **11** (150 mg, 0.37 mmol) and **17** (62 mg, 0.41 mmol) in toluene (5 mL) were added Cs_2CO_3 (362 mg, 1.11 mmol), Xantphos (64 mg, 0.11 mmol), and $Pd_2(dba)_3$ (38 mg, 0.04 mmol). The resulting mixture was refluxed for 12 h, and then diluted with DCM. The organic layer was washed with brine, dried over $MgSO_4$, filtered, and concentrated. The residue was purified by silica gel column chromatography with 5% MeOH/DCM to obtain **22I** (96 mg, 55%). Yellow solid; TLC R_f 0.31 (EtOAc/Hexanes = 1/2); m.p. 233 °C; MS (EI) *m*/*z* 473 $[M]^+$; ^{1}H NMR ($CDCl_3$) δ 8.06 (s, 1H), 8.03 (d, *J* = 2.7 Hz, 1H), 7.70 (d, *J* = 8.8 Hz, 1H), 6.70 (t, *J* = 8.2 Hz, 1H), 6.27 (s, 1H), 4.89 (t, *J* = 1.8 Hz, 2H), 3.91 (s, 6H), 2.49 (s, 3H), 2.38 (s, 3H), 2.32 (s, 3H), 2.14 (s, 3H); ^{13}C NMR ($CDCl_3$) δ 148.2, 148.1, 147.4, 147.3, 147.0, 143.9 (d, *J* = 5.0 Hz), 143.7 (d, *J* = 4.9 Hz), 143.5 (d, *J* = 5.8 Hz), 141.6, 140.2, 126.2, 123.8, 123.8, 120.5, 114.5, 114.1 (t, *J* = 18.1 Hz), 102.3, 62.4, 57.6 (2C), 19.1, 17.9, 13.9, 13.0; HRMS (EI) *m*/*z* calculated for $C_{24}H_{25}F_2N_3O_5$ $[M]^+$ 473.1762, found 473.1756.

5-((2,6-Dichloro-3,5-dimethoxybenzyl)oxy)-3,4,6-trimethyl-*N*-(2-methyl-4-nitrophenyl)pyridin-2-amine (22J)

To a mixture of **12** (150 mg, 0.35 mmol) and **17** (58 mg, 0.38 mmol) in toluene (5 mL) were added Cs_2CO_3 (337 mg, 1.04 mmol), Xantphos (60 mg, 0.10 mmol), and $Pd_2(dba)_3$ (36 mg, 0.04 mmol). The resulting mixture was refluxed for 12 h, and then diluted with DCM. The

organic layer was washed with brine, dried over $MgSO_4$, filtered, and concentrated. The residual solid was washed with EtOAc to obtain **22J** (103 mg, 59%). Yellow solid; TLC R_f 0.26 (EtOAc/Hexanes = 1/2); m.p. 265 °C; MS (EI) *m*/*z* 505 $[M]^+$; ^{1}H NMR ($CDCl_3$) δ 8.15–7.97 (m, 2H), 7.61 (d, *J* = 8.8 Hz, 1H), 6.63 (s, 1H), 6.26 (s, 1H), 5.23 (s, 2H), 3.94 (s, 6H), 2.46 (s, 3H), 2.37 (s, 3H), 2.27 (s, 3H), 2.11 (s, 3H); ^{13}C NMR ($CDCl_3$) δ 154.8 (2C), 148.8, 148.2, 147.5, 146.7, 141.8, 140.2, 134.0, 126.2, 123.8 (2C), 120.7, 116.9, 116.5, 114.4, 98.2, 69.9, 56.8 (2C), 19.7, 17.9, 14.0, 13.7; HRMS (EI) *m*/*z* calculated for $C_{24}H_{25}Cl_2N_3O_5$ $[M]^+$ 505.1171, found 505.1171.

5-((2,6-Difluoro-3,5-dimethoxybenzyl)oxy)-4,6-dimethyl-*N*-(2-methyl-4-nitrophenyl)pyrimidin-2-amine (22K)

To a mixture of **13** (150 mg, 0.39 mmol) and **17** (65 mg, 0.42 mmol) in toluene (3 mL) were added Cs_2CO_3 (376 mg, 1.16 mmol), Xantphos (67 mg, 0.12 mmol), and $Pd_2(dba)_3$ (40 mg, 0.04 mmol). The resulting mixture was refluxed for 12 h, and then diluted with DCM. The organic layer was washed with brine, dried over $MgSO_4$, filtered, and concentrated. The residue was purified by silica gel column chromatography with DCM to obtain **22K** (102 mg, 58%). Yellow solid; TLC R_f 0.17 (Hexanes/EtOAc = 1/1); m.p. 232 °C; MS (EI) *m*/*z* 460 $[M]^+$; ^{1}H NMR ($CDCl_3$) δ 8.71 (d, *J* = 9.1 Hz, 1H), 8.13 (dd, *J* = 9.2, 2.6 Hz, 1H), 8.07 (d, *J* = 2.6 Hz,1H), 7.08 (t, *J* = 8.0 Hz, 1H), 6.71 (t, *J* = 8.2 Hz, 1H), 4.93 (t, *J* = 1.8 Hz, 2H), 3.90 (s, 6H), 2.46 (s, 6H), 2.41 (s, 3H); ^{13}C NMR ($CDCl_3$) δ 161.8, 154.4 (2C), 144.7, 144.6, 143.9, 141.2, 135.0, 129.1, 128.5, 125.9, 125.0, 123.5, 116.9, 113.7, 102.2, 62.6, 57.5 (2C), 19.0 (2C), 18.2; HRMS (EI) *m*/*z* calculated for $C_{22}H_{22}F_2N_4O_5$ $[M]^+$ 460.1528, found 460.1553.

5-((2,6-Dichloro-3,5-dimethoxybenzyl)oxy)-4,6-dimethyl-*N*-(2-methyl-4-nitrophenyl)pyrimidin-2-amine (22L)

To a mixture of **14** (150 mg, 0.36 mmol) and **17** (60 mg, 0.39 mmol) in toluene (3 mL) were added Cs_2CO_3 (347 mg, 1.07 mmol), Xantphos (62 mg, 0.11 mmol), and $Pd_2(dba)_3$ (37 mg, 0.04 mmol). The resulting mixture was refluxed for 7 h, and then diluted with DCM. The organic layer was washed with brine, dried over $MgSO_4$, filtered, and concentrated. The residue was purified by silica gel column chromatography with DCM to obtain **22L** (91 mg, 52%). Yellow solid; TLC R_f 0.28 (Hexanes/EtOAc = 2/1); m.p. 262 °C; MS (EI) *m*/*z* 492 $[M]^+$; ^{1}H NMR (DMSO-d_6) δ 8.77 (s, 1H), 8.23 (d, *J* = 8.7 Hz, 1H), 8.05 (d, *J* = 11.5 Hz, 2H), 7.01 (s,

1H), 5.16 (s, 2H), 3.94 (s, 6H), 2.38 (s, 3H), 2.29 (s, 6H); ^{13}C NMR (DMSO-d_6) δ 171.1, 160.6, 154.4 (2C), 145.4, 143.7, 140.9, 132.6, 128.8, 125.3, 122.0, 119.6, 115.0 (2C), 111.5, 99.2, 69.3, 56.8 (2C), 18.7 (2C), 17.9.; HRMS (EI) *m/z* calculated for $C_{22}H_{22}Cl_2N_4O_5$ $[M]^+$ 492.0967, found 492.0968.

***N*-(2-Chloro-4-fluoro-6-nitrophenyl)-5-((2,6-difluoro-3,5-dimethoxybenzyl)oxy)-3,4,6-trimethylpyridin-2-amine (22M)**

To a mixture of **11** (100 mg, 0.25 mmol) and **18** (47 mg, 0.25 mmol) in toluene (5 mL) were added Cs_2CO_3 (244 mg, 0.75 mmol), BINAP (3.1 mg, 0.01 mmol), and $Pd_2(dba)_3$ (2.5 mg, 0.002 mmol). The resulting mixture was refluxed for 30 h, and then diluted with EtOAc. The organic layer was washed with brine, dried over $MgSO_4$, filtered, and concentrated. The residue was purified by silica gel column chromatography with 20% to 50% DCM/Hexanes to obtain **22M** (120 mg, 95%). Orange solid; TLC R_f 0.50 (EtOAc/Hexanes = 1/2); m.p. 265 °C; MS (ESI) *m/z* 512 $[M+H]^+$; ^{1}H NMR ($CDCl_3$) δ 8.17 (d, *J* = 9.0 Hz, 2H), 6.73 (t, *J* = 8.1 Hz, 1H), 4.95 (d, *J* = 1.6 Hz, 2H), 3.92 (s, 6H), 2.99 (s, 3H), 2.74 (s, 3H), 2.49 (s, 3H); ^{13}C NMR ($CDCl_3$) δ 156.7, 152.9, 151.8, 147.2, 144.0, 143.8, 143.6 (2C), 137.3 (2C), 136.5, 128.9, 124.2, 113.5, 110.7, 108.3, 102.6, 63.8, 57.6 (2C), 14.4, 14.0, 13.6.

***N*-(2-Chloro-4-fluoro-6-nitrophenyl)-5-((2,6-dichloro-3,5-dimethoxybenzyl)oxy)-3,4,6-trimethylpyridin-2-amine (22N)**

To a mixture of **12** (150 mg, 0.35 mmol) and **18** (96 mg, 0.50 mmol) in toluene (5 mL) were added Cs_2CO_3 (450 mg, 1.38 mmol), Xantphos (72 mg, 0.12 mmol), and $Pd_2(dba)_3$ (50 mg, 0.05 mmol). The resulting mixture was refluxed for 15 h, and then diluted with EtOAc. The organic layer was washed with brine, dried over $MgSO_4$, filtered, and concentrated. The residue was purified by silica gel column chromatography with 30% to 50% EtOAc/Hexanes to obtain **22N** (153 mg, 81%). Yellow solid; TLC R_f 0.39 (EtOAc/Hexanes = 1/2); m.p. 200 °C; MS (EI) *m/z* 545 $[M]^+$; ^{1}H NMR ($CDCl_3$) δ 7.66 (dd, *J* = 8.1, 3.0 Hz, 1H), 7.41 (dd, *J* = 7.4, 3.0 Hz, 1H), 7.24 (s, 1H), 6.60 (s, 1H), 5.13 (s, 2H), 3.92 (s, 6H), 2.26 (s, 3H), 2.24 (s, 3H), 2.23 (s, 3H); ^{13}C NMR ($CDCl_3$) δ 157.4, 154.7 (2C), 153.5, 148.0, 146.8, 146.2, 142.4 (d, *J* = 9.4 Hz), 141.1, 134.1, 131.2 (d, *J* = 3.4 Hz), 129.3 (d, *J* = 10.0 Hz), 122.1 (d, *J* = 25.5 Hz), 117.6, 116.7, 111.5 (d, *J* = 26.7 Hz), 98.0, 69.8, 56.7 (2C), 19.2, 13.4, 13.2; HRMS (EI) *m/z* calculated for $C_{23}H_{21}Cl_3FN_3O_5$ $[M]^+$ 543.0531, found 543.0525.

_N_-(2-Chloro-4-fluoro-6-nitrophenyl)-5-((2,6-difluoro-3,5-dimethoxybenzyl)oxy)-4,6-dimethylpyrimidin-2-amine (22O)

To a mixture of **13** (150 mg, 0.39 mmol) and **18** (110 mg, 0.58 mmol) in toluene (3 mL) were added Cs_2CO_3 (376 mg, 1.16 mmol), Xantphos (69 mg, 0.12 mmol), and $Pd_2(dba)_3$ (40 mg, 0.04 mmol). The resulting mixture was refluxed for 30 h, and then diluted with DCM. The organic layer was washed with brine, dried over $MgSO_4$, filtered, and concentrated. The residue was purified by silica gel column chromatography with 10% to 20% EtOAc/Hexanes to obtain **22O** (136 mg, 71%). Yellow solid; TLC R_f 0.32 (Hexanes/EtOAc = 2/1); m.p. 170 °C; MS (EI) *m/z* 498 $[M]^+$; ^{1}H NMR ($CDCl_3$) δ 7.64 (dd, *J* = 7.9, 3.0 Hz, 1H), 7.45 (dd, *J* = 7.3, 3.0 Hz, 1H), 7.32 (s, 1H), 6.69 (t, *J* = 8.2 Hz, 1H), 4.86 (t, *J* = 1.8 Hz, 2H), 3.89 (s, 6H), 2.35 (s, 6H); ^{13}C NMR ($CDCl_3$) δ 161.8, 158.9, 154.9, 153.5, 147.2 (d, *J* = 5.6 Hz), 144.8, 144.5 (d, *J* = 8.8 Hz), 143.8 (d, *J* = 5.0 Hz), 143.7 (d, *J* = 4.9 Hz), 143.3 (d, *J* = 5.8 Hz), 130.7 (d, *J* = 10.0 Hz), 128.0 (d, *J* = 4.0 Hz), 121.6 (d, *J* = 25.3 Hz), 113.7 (t, *J* = 17.9 Hz), 111.8 (d, *J* = 26.8 Hz), 102.4 (t, *J* = 2.3 Hz), 62.5 (t, *J* = 3.6 Hz), 57.6 (2C), 18.6 (2C); HRMS (EI) *m/z* calculated for $C_{21}H_{18}ClF_3N_4O_5$ $[M]^+$ 498.0918, found 498.0914.

_N_-(2-Chloro-4-fluoro-6-nitrophenyl)-5-((2,6-dichloro-3,5-dimethoxybenzyl)oxy)-4,6-dimethylpyrimidin-2-amine (22P)

To a mixture of **14** (100 mg, 0.24 mmol) and **18** (68 mg, 0.36 mmol) in toluene (3 mL) were added Cs_2CO_3 (232 mg, 0.71 mmol), Xantphos (41 mg, 0.07 mmol), and $Pd_2(dba)_3$ (25 mg, 0.02 mmol). The resulting mixture was refluxed for 12 h, and then diluted with DCM. The organic layer was washed with brine, dried over $MgSO_4$, filtered, and concentrated. The residue was purified by silica gel column chromatography with 20% to 30% EtOAc/Hexanes to obtain **22P** (57 mg, 45%). Yellow solid; TLC R_f 0.26 (Hexanes/EtOAc = 2/1); m.p. 211 °C; MS (EI) *m/z* 532 $[M]^+$; ^{1}H NMR ($CDCl_3$) δ 7.62 (dd, *J* = 7.9, 3.0 Hz, 1H), 7.44 (dd, *J* = 7.3, 3.0 Hz, 1H), 7.35 (s, 1H), 6.60 (s, 1H), 5.15 (s, 2H), 3.92 (s, 6H), 2.31 (s, 6H); ^{13}C NMR ($CDCl_3$) δ 161.7 (2C), 158.8, 154.7 (2C), 153.2, 145.2, 144.4 (d, *J* = 8.8 Hz), 133.4, 130.6 (d, *J* = 10.1 Hz), 128.0 (d, *J* = 3.9 Hz), 121.5 (d, *J* = 25.3 Hz), 116.8 (2C), 111.7 (d, *J* = 26.8 Hz), 98.2, 69.8, 56.8 (2C), 19.2 (2C); HRMS (EI) *m/z* calculated for $C_{21}H_{18}Cl_3FN_4O_5$ $[M]^+$ 530.0327, found 530.0328.

5-((2,6-Difluoro-3,5-dimethoxybenzyl)oxy)-*N*-(2-methoxy-6-nitrophenyl)-3,4,6-trimethylpyridin-2-amine (22Q)

To a mixture of **11** (150 mg, 0.37 mmol) and **19** (68 mg, 0.41 mmol) in toluene (5 mL) were added Cs_2CO_3 (362 mg, 1.11 mmol), Xantphos (64 mg, 0.11 mmol), and $Pd_2(dba)_3$ (38 mg, 0.04 mmol). The resulting mixture was refluxed for 16 h, and then diluted with DCM. The organic layer was washed with brine, dried over $MgSO_4$, filtered, and concentrated. The residue was purified by silica gel column chromatography with 35% EtOAc/Hexanes to obtain **22Q** (118 mg, 65%). Yellow solid; TLC R_f 0.29 (EtOAc/Hexanes = 1/2); m.p. 195 °C; MS (EI) *m*/*z* 489 $[M]^+$; ^{1}H NMR ($CDCl_3$) δ 7.56 (dd, *J* = 8.2, 1.6 Hz, 1H), 7.06 (dd, *J* = 8.1, 1.6 Hz, 1H), 6.98 (t, *J* = 8.1 Hz, 1H), 6.68 (t, *J* = 8.1 Hz, 1H), 4.81 (t, *J* = 1.8 Hz, 2H), 3.89 (s, 6H), 3.87 (s, 3H), 2.30 (s, 3H), 2.28 (s, 3H), 2.24 (s, 3H); ^{13}C NMR ($CDCl_3$) δ 151.5, 147.4 (d, *J* = 5.9 Hz), 147.1, 147.0, 146.6, 143.8 (d, *J* = 4.9 Hz), 143.6 (d, *J* = 1.7 Hz), 143.5, 141.6, 140.5, 127.0, 120.3, 117.7, 117.4, 114.7, 114.4, 102.3, 62.28–62.09 (m), 57.6 (2C), 56.6, 18.5, 13.3, 12.7; HRMS (EI) *m*/*z* calculated for $C_{24}H_{25}F_2N_3O_6$ $[M]^+$ 489.1711, found 489.1707.

5-((2,6-Dichloro-3,5-dimethoxybenzyl)oxy)-*N*-(2-methoxy-6-nitrophenyl)-3,4,6-trimethylpyridin-2-amine (22R)

To a mixture of **12** (150 mg, 0.35 mmol) and **19** (62 mg, 0.38 mmol) in toluene (5 mL) were added Cs_2CO_3 (337 mg, 1.04 mmol), Xantphos (59 mg, 0.10 mmol), and $Pd_2(dba)_3$ (35 mg, 0.03 mmol). The resulting mixture was refluxed for 15 h, and then diluted with DCM. The organic layer was washed with brine, dried over $MgSO_4$, filtered, and concentrated. The residue was purified by silica gel column chromatography with 40% EtOAc/Hexanes to obtain **22R** (131 mg, 74%). Yellow solid; TLC R_f 0.23 (EtOAc/Hexanes = 1/2); m.p. 223 °C; MS (EI) *m*/*z* 521 $[M]^+$; ^{1}H NMR ($CDCl_3$) δ 7.56 (dd, *J* = 8.2, 1.5 Hz, 1H), 7.30 (s, 1H), 7.10–6.91 (m, 2H), 6.60 (s, 1H), 5.13 (s, 2H), 3.91 (s, 6H), 3.85 (s, 3H), 2.29 (s, 3H), 2.24 (s, 3H), 2.22 (s, 3H); ^{13}C NMR ($CDCl_3$) δ 154.7 (2C), 151.5, 147.7, 146.8, 146.6, 141.4, 140.5, 134.3, 127.2, 120.2 (2), 117.7, 117.4, 116.9, 114.4 (2C), 98.1, 69.8, 56.8 (2C), 56.6, 19.3, 13.4, 13.3; HRMS (EI) *m*/*z* calculated for $C_{24}H_{25}Cl_2N_3O_6$ $[M]^+$ 521.1120, found 521.1118.

5-((2,6-Difluoro-3,5-dimethoxybenzyl)oxy)-*N*-(2-methoxy-6-nitrophenyl)-4,6-dimethylpyrimidin-2-amine (22S)

To a mixture of **13** (150 mg, 0.39 mmol) and **19** (71 mg, 0.42 mmol) in toluene (3 mL) were added Cs_2CO_3 (376 mg, 1.16 mmol), Xantphos (67 mg, 0.12 mmol), and $Pd_2(dba)_3$ (40 mg, 0.04 mmol). The resulting mixture was refluxed for 20 h, and then diluted with DCM. The organic layer was washed with brine, dried over $MgSO_4$, filtered, and concentrated. The residue was purified by silica gel column chromatography with 15% to 25% EtOAc/Hexanes to obtain **22S** (141 mg, 77%). Yellow solid; TLC R_f 0.10 (Hexanes/EtOAc = 2/1); m.p. 162 °C; MS (EI) *m*/*z* 476 $[M]^+$; ^{1}H NMR ($CDCl_3$) δ 7.51 (d, *J* = 3.3 Hz, 2H), 7.15–7.05 (m, 2H), 6.68 (t, *J* = 8.2 Hz, 1H), 4.85 (t, *J* = 1.5 Hz, 2H), 3.91 (s, 3H), 3.89 (s, 6H), 2.36 (s, 6H); ^{13}C NMR ($CDCl_3$) δ 161.5, 153.9, 151.9, 147.2 (d, *J* = 5.9 Hz), 144.5, 143.8, 143.7 (d, *J* = 5.6 Hz), 143.6, 143.3 (d, *J* = 5.8 Hz), 143.2, 123.7, 122.4, 117.1, 114.1, 113.6 (d, *J* = 18.0 Hz), 102.3, 62.4 (t, *J* = 3.6 Hz), 57.5 (2C), 56.6, 18.6 (2C); HRMS (EI) *m*/*z* calculated for $C_{22}H_{22}F_2N_4O_6$ $[M]^+$ 476.1507, found 476.1502.

5-((2,6-Dichloro-3,5-dimethoxybenzyl)oxy)-*N*-(2-methoxy-6-nitrophenyl)-4,6-dimethylpyrimidin-2-amine (22T)

To a mixture of **14** (150 mg, 0.36 mmol) and **19** (66 mg, 0.39 mmol) in toluene (3 mL) were added Cs_2CO_3 (347 mg, 1.07 mmol), Xantphos (62 mg, 0.11 mmol), and $Pd_2(dba)_3$ (36 mg, 0.04 mmol). The resulting mixture was refluxed for 6 h, and then diluted with DCM. The organic layer was washed with brine, dried over $MgSO_4$, filtered, and concentrated. The residue was purified by silica gel column chromatography with 5% EtOAc/DCM to obtain **22T** (117 mg, 65%). Yellow solid; TLC R_f 0.14 (Hexanes/EtOAc = 2/1); m.p. 225 °C; MS (EI) *m*/*z* 508 $[M]^+$; ^{1}H NMR ($CDCl_3$) δ 7.51 (dd, *J* = 5.6, 3.9 Hz, 2H), 7.15–7.01 (m, 2H), 6.60 (s, 1H), 5.15 (s, 2H), 3.92 (s, 6H), 3.90 (s, 3H), 2.34 (s, 6H); ^{13}C NMR ($CDCl_3$) δ 161.4, 154.7 (2C), 153.7 (2C), 151.8, 144.9, 143.1, 133.6, 123.8, 122.3, 117.1, 116.8, 114.1 (2C), 98.2, 69.8, 56.8 (2C), 56.5, 19.2 (2C); HRMS (EI) *m*/*z* calculated for $C_{22}H_{22}Cl_2N_4O_6$ $[M]^+$ 508.1916, found 508.1912.

5-((2,6-Difluoro-3,5-dimethoxybenzyl)oxy)-*N*-(5-methoxy-2-nitrophenyl)-3,4,6-trimethylpyridin-2-amine (22U)

To a mixture of **11** (200 mg, 0.50 mmol) and **20** (115 mg, 0.55 mmol) in toluene (10 mL) were added Cs_2CO_3 (486 mg, 1.49 mmol), BINAP (15 mg, 0.02 mmol), and $Pd_2(dba)_3$ (13 mg, 0.01 mmol). The resulting mixture was refluxed for 30 h, and then diluted with EtOAc. The organic layer was washed with brine, dried over $MgSO_4$, filtered, and concentrated. The residue was

purified by silica gel column chromatography with DCM to obtain **22U** (106 mg, 44%). Yellow solid; TLC R_f 0.53 (EtOAc/Hexanes = 1/2); m.p. 228 °C; MS (EI) *m*/*z* 489 $[M]^+$; ^{1}H NMR ($CDCl_3$) δ 10.68 (s, 1H), 8.57 (d, *J* = 2.7 Hz, 1H), 8.21 (d, *J* = 9.5 Hz, 1H), 6.70 (t, *J* = 8.2 Hz, 1H), 6.44 (dd, *J* = 9.5, 2.7 Hz, 1H), 4.88 (t, *J* = 1.7 Hz, 2H), 3.91 (s, 6H), 3.90 (s, 3H), 2.52 (s, 3H), 2.33 (s, 3H), 2.28 (s, 3H); ^{13}C NMR ($CDCl_3$) δ 165.9, 147.5 (2C), 147.3 (d, *J* = 5.9 Hz), 146.9, 143.8 (d, *J* = 4.9 Hz), 143.7 (d, *J* = 4.8 Hz), 143.4 (d, *J* = 6.0 Hz), 143.0, 141.3, 128.5, 127.9, 120.3, 114.1 (t, *J* = 18.1 Hz), 108.4, 102.1, 100.3, 62.3, 57.5 (2C), 55.9, 19.3, 13.5, 13.0; HRMS (EI) *m*/*z* calculated for $C_{24}H_{25}F_2N_3O_6$ $[M]^+$ 489.1711, found 489.1711.

5-((2,6-Dichloro-3,5-dimethoxybenzyl)oxy)-*N*-(5-methoxy-2-nitrophenyl)-3,4,6-trimethylpyridin-2-amine (22V)

To a mixture of **12** (200 mg, 0.46 mmol) and **20** (85 mg, 0.51 mmol) in toluene (5 mL) were added Cs_2CO_3 (450 mg, 1.38 mmol), Xantphos (80 mg, 0.14 mmol), and $Pd_2(dba)_3$ (48 mg, 0.05 mmol). The resulting mixture was refluxed for 18 h, and then diluted with DCM. The organic layer was washed with brine, dried over $MgSO_4$, filtered, and concentrated. The residue was purified by silica gel column chromatography with 30% to 40% EtOAc/Hexanes to obtain **22V** (171 mg, 71%). Yellow solid; TLC R_f 0.58 (EtOAc/Hexanes = 1/2); m.p. 243 °C; MS (EI) *m*/*z* 521 $[M]^+$; ^{1}H NMR ($CDCl_3$) δ 10.63 (s, 1H), 8.49 (d, *J* = 2.7 Hz, 1H), 8.20 (d, *J* = 9.5 Hz, 1H), 6.62 (s, 1H), 6.42 (dd, *J* = 9.5, 2.7 Hz, 1H), 5.21 (s, 2H), 3.94 (s, 6H), 3.88 (s, 3H), 2.49 (s, 3H), 2.27 (s, 3H), 2.26 (s, 3H); ^{13}C NMR ($CDCl_3$) δ 165.9, 154.8 (2C), 148.1, 147.2, 146.9, 143.1, 141.4, 134.1, 128.5, 127.9, 120.3, 116.9, 108.4 (2C), 100.2, 98.2, 69.9, 56.8 (2C), 55.8, 19.9, 13.7, 13.5; HRMS (EI) *m*/*z* calculated for $C_{24}H_{25}Cl_2N_3O_6$ $[M]^+$ 521.1120, found 521.1120.

5-((2,6-Dicfluoro-3,5-dimethoxybenzyl)oxy)-*N*-(5-methoxy-2-nitrophenyl)-4,6-dimethylpyrimidin-2-amine (22W)

To a mixture of **13** (150 mg, 0.39 mmol) and **20** (71 mg, 0.42 mmol) in toluene (3 mL) were added Cs_2CO_3 (346 mg, 1.16 mmol), Xantphos (67 mg, 0.12 mmol), and $Pd_2(dba)_3$ (40 mg, 0.04 mmol). The resulting mixture was refluxed for 6 h, and then diluted with DCM. The organic layer was washed with brine, dried over $MgSO_4$, filtered, and concentrated. The residue was purified by silica gel column chromatography with 30% to 40% EtOAc/Hexanes to obtain **22W** (110 mg, 60%). Yellow solid; TLC R_f 0.35 (Hexanes/EtOAc = 2/1); m.p. 218 °C; MS (EI) *m*/*z* 476 $[M]^+$; ^{1}H NMR ($CDCl_3$) δ 10.83 (s, 1H), 8.83 (d, *J* = 2.6 Hz, 1H), 8.22 (d, *J* = 9.5 Hz,

1H), 6.70 (t, *J* = 8.2 Hz, 1H), 6.57–6.45 (m, 1H), 4.93 (s, 2H), 3.94 (s, 3H), 3.90 (s, 6H), 2.47 (s, 6H); ^{13}C NMR ($CDCl_3$) δ 165.6, 161.6, 154.5 (2C), 147.2 (d, *J* = 5.8 Hz), 144.9 (2C), 143.8 (dd, *J* = 10.5, 4.9 Hz), 143.3 (d, *J* = 5.8 Hz), 140.7 (2C), 128.7, 128.5, 114.1–113.3 (m), 108.6, 102.2, 62.5 (t, *J* = 4.5 Hz), 57.5 (2C), 55.9, 19.1 (2C); HRMS (EI) *m*/*z* calculated for $C_{22}H_{22}F_2N_4O_6$ $[M]^+$ 476.1507, found 476.1510.

5-((2,6-Dichloro-3,5-dimethoxybenzyl)oxy)-*N*-(5-methoxy-2-nitrophenyl)-4,6-dimethylpyrimidin-2-amine (22X)

To a mixture of **14** (91 mg, 0.22 mmol) and **20** (40 mg, 0.24 mmol) in toluene (3 mL) were added Cs_2CO_3 (211 mg, 0.65 mmol), Xantphos (38 mg, 0.07 mmol), and $Pd_2(dba)_3$ (23 mg, 0. 02 mmol). The resulting mixture was refluxed for 12 h, and then diluted with DCM. The organic layer was washed with brine, dried over $MgSO_4$, filtered, and concentrated. The residue was purified by silica gel column chromatography with 20% to 50% EtOAc/Hexanes to obtain **22X** (109 mg, 99%). Yellow solid; TLC R_f 0.27 (Hexanes/EtOAc = 2/1); m.p. 265 °C; MS (EI) *m*/*z* 508 $[M]^+$; ^{1}H NMR ($CDCl_3$) δ 10.82 (s, 1H), 8.83 (d, *J* = 2.7 Hz, 1H), 8.23 (d, *J* = 9.5 Hz, 1H), 6.63 (s, 1H), 6.50 (dd, *J* = 9.5, 2.7 Hz, 1H), 5.24 (s, 2H), 3.94 (s, 6H), 3.93 (s, 3H), 2.45 (s, 6H); ^{13}C NMR ($CDCl_3$) δ 165.6, 161.5 (2C), 154.8 (2C), 154.3, 145.3, 140.7, 133.5, 128.7, 128.6, 116.8 (2C), 108.5, 102.1, 98.2, 69.9, 56.8 (2C), 55.9, 19.6 (2C); HRMS (EI) *m*/*z* calculated for $C_{22}H_{22}Cl_2N_4O_6$ $[M]^+$ 508.0916, found 508.0918.

5-((2,6-Difluoro-3,5-dimethoxybenzyl)oxy)-*N*-(5-(4-ethylpiperazin-1-yl)-2-nitrophenyl)-3,4,6-trimethylpyridin-2-amine (22Y)

To a mixture of **11** (200 mg, 0.50 mmol) and **21** (137 mg, 0.55 mmol) in toluene (10 mL) were added Cs_2CO_3 (489 mg, 1.49 mmol), Xantphos (87 mg, 0.15 mmol), and $Pd_2(dba)_3$ (52 mg, 0.05 mmol). The resulting mixture was refluxed for 2 h, and then diluted with DCM. The organic layer was washed with brine, dried over $MgSO_4$, filtered, and concentrated. The residue was purified by silica gel column chromatography with 3% to 5% MeOH/DCM to obtain **22Y** (269 mg, 94%). Orange solid; TLC R_f 0.45 (DCM/MeOH = 20/1); m.p. 220 °C; MS (EI) *m*/*z* 571 $[M]^+$; ^{1}H NMR ($CDCl_3$) δ 10.83 (s, 1H), 8.58 (d, *J* = 2.4 Hz, 1H), 8.16 (d, *J* = 9.7 Hz, 1H), 6.70 (t, *J* = 8.1 Hz, 1H), 6.40 (dd, *J* = 9.7, 2.6 Hz, 1H), 4.86 (s, 2H), 3.90 (s, 6H), 3.61–3.41 (m, 4H), 2.68–2.55 (m, 4H), 2.50 (s, 3H), 2.45 (d, *J* = 7.2 Hz, 1H), 2.32 (s, 3H), 2.27 (s, 3H), 1.13 (t, *J* = 7.2 Hz, 3H); ^{13}C NMR ($CDCl_3$) δ 155.9, 148.1, 147.3 (d, *J* = 6.0 Hz), 147.0, 146.3,

143.8 (d, J = 4.8 Hz), 143.6 (d, J = 4.9 Hz), 143.4 (d, J = 5.9 Hz), 142.6, 141.0, 128.8, 125.4, 119.8, 114.2 (t, J = 18.1 Hz), 106.3, 102.1, 99.7, 62.3, 57.5 (2C), 52.6 (2C), 52.4, 47.0 (2C), 19.3, 13.4, 13.0, 12.1; HRMS (EI) *m/z* calculated for $C_{29}H_{35}F_2N_5O_5$ $[M]^+$ 571.2606, found 571.2601.

5-((2,6-Dichloro-3,5-dimethoxybenzyl)oxy)-*N*-(5-(4-ethylpiperazin-1-yl)-2-nitrophenyl)-3,4,6-trimethylpyridin-2-amine (22Z)

To a mixture of **12** (200 mg, 0.46 mmol) and **21** (127 mg, 0.51 mmol) in toluene (10 mL) were added Cs_2CO_3 (150 mg, 1.38 mmol), Xantphos (80 mg, 0.14 mmol), and $Pd_2(dba)_3$ (48 mg, 0.05 mmol). The resulting mixture was refluxed for 4 h, and then diluted with DCM. The organic layer was washed with brine, dried over $MgSO_4$, filtered, and concentrated. The residue was purified by silica gel column chromatography with 3% to 5% MeOH/DCM to obtain **22Z** (269 mg, 97%). Yellow solid; TLC R_f 0.45 (DCM/MeOH = 9/1); m.p. 240 °C; MS (EI) *m/z* 603 $[M]^+$; ^1H NMR ($CDCl_3$) δ 10.79 (s, 1H), 8.48 (d, J = 2.6 Hz, 1H), 8.15 (d, J = 9.7 Hz, 1H), 6.61 (s, 1H), 6.39 (dd, J = 9.7, 2.6 Hz, 1H), 5.19 (s, 2H), 3.94 (s, 6H), 3.61 – 3.39 (m, 4H), 2.66–2.53 (m, 4H), 2.52–2.40 (m, 5H), 2.26 (d, J = 4.3 Hz, 6H), 1.13 (t, J = 7.2 Hz, 3H); ^{13}C NMR ($CDCl_3$) δ 164.2, 155.9, 154.7 (2C), 147.7, 147.6, 146.4, 142.7, 141.1, 134.1, 128.8, 125.4, 119.9, 116.8 (2C), 106.2, 99.5, 98.0, 69.9, 56.8 (2C), 52.5 (2C), 52.4, 47.0 (2C), 20.0, 13.6, 13.5, 12.1; HRMS (EI) *m/z* calculated for $C_{29}H_{35}Cl_2N_5O_5$ $[M]^+$ 603.2015, found 603.2007.

5-((2,6-Difluoro-3,5-dimethoxybenzyl)oxy)-*N*-(5-(4-ethylpiperazin-1-yl)-2-nitrophenyl)-4,6-dimethylpyrimidin-2-amine (22AA)

To a mixture of **13** (100 mg, 0.26 mmol) and **21** (71 mg, 0.28 mmol) in toluene (3 mL) were added Cs_2CO_3 (251 mg, 0.77 mmol), Xantphos (45 mg, 0.08 mmol), and $Pd_2(dba)_3$ (27 mg, 0.03 mmol). The resulting mixture was refluxed for 6 h, and then diluted with DCM. The organic layer was washed with brine, dried over $MgSO_4$, filtered, and concentrated. The residue was purified by silica gel column chromatography with 3% to 5% MeOH/DCM to obtain **22AA** (125 mg, 87%). Yellow solid; TLC R_f 0.47 (DCM/MeOH = 20/1); m.p. 198 °C; MS (EI) *m/z* 558 $[M]^+$; ^1H NMR ($CDCl_3$) δ 10.95 (s, 1H), 8.68 (d, J = 2.7 Hz, 1H), 8.15 (d, J = 9.7 Hz, 1H), 6.69 (t, J = 8.2 Hz, 1H), 6.42 (dd, J = 9.7, 2.7 Hz, 1H), 4.93 (d, J = 10.6 Hz, 2H), 3.89 (s, 6H), 3.63–3.42 (m, 4H), 2.69–2.57 (m, 4H), 2.54–2.47 (m, 2H), 2.44 (s, 6H), 1.14 (t, J = 7.2 Hz, 3H); ^{13}C NMR ($CDCl_3$) δ 161.3 (2C), 155.6, 154.8, 147.1 (d, J = 5.8 Hz), 144.5, 143.8 (d, J =

5.0 Hz), 143.7 (d, J = 4.9 Hz), 143.2 (d, J = 5.8 Hz), 140.5, 128.7, 125.9, 113.7 (t, J = 17.9 Hz), 106.5, 102.2, 101.1, 62.5, 57.5 (2C), 52.5 (2C), 52.4, 47.0 (2C), 19.1 (2C), 12.1; HRMS (EI) m/z calculated for $C_{27}H_{32}F_2N_6O_5$ $[M]^+$ 558.2402, found 558.2399.

5-((2,6-Dichloro-3,5-dimethoxybenzyl)oxy)-*N*-(5-(4-ethylpiperazin-1-yl)-2-nitrophenyl)-4,6-dimethylpyrimidin-2-amine (22BB)

To a mixture of **14** (100 mg, 0.24 mmol) and **21** (62 mg, 0.26 mmol) in toluene (3 mL) were added Cs_2CO_3 (232 mg, 0.71 mmol), Xantphos (41 mg, 0.07 mmol), and $Pd_2(dba)_3$ (25 mg, 0.02 mmol). The resulting mixture was refluxed for 8 h, and then diluted with DCM. The organic layer was washed with brine, dried over $MgSO_4$, filtered, and concentrated. The residue was purified by silica gel column chromatography with 3% to 5% MeOH/DCM to obtain **22BB** (125 mg, 89%). Yellow solid; TLC R_f 0.40 (DCM/MeOH = 20/1); m.p. 235 °C; MS (EI) m/z 590 $[M]^+$; ^{1}H NMR ($CDCl_3$) δ 10.93 (s, 1H), 8.70 (d, J = 2.7 Hz, 1H), 8.16 (d, J = 9.7 Hz, 1H), 6.63 (s, 1H), 6.43 (dd, J = 9.7, 2.8 Hz, 1H), 5.22 (s, 2H), 3.93 (s, 6H), 3.60–3.44 (m, 4H), 2.66–2.55 (m, 4H), 2.53–2.45 (m, 2H), 2.43 (s, 6H), 1.13 (t, J = 7.2 Hz, 3H); ^{13}C NMR ($CDCl_3$) δ 161.2 (2C), 155.7, 154.9 (2C), 154.6, 145.0, 140.6, 133.6, 128.7, 126.0, 116.9 (2C), 106.5, 101.3, 98.4, 69.9, 56.8 (2C), 52.6 (2C), 52.4, 47.1 (2C), 19.7 (C), 12.1; HRMS (EI) m/z calculated for $C_{27}H_{32}Cl_2N_6O_5$ $[M]^+$ 590.1811, found 590.1804.

***N*^{3}-(5-((2,6-Difluoro-3,5-dimethoxybenzyl)oxy)-3,4,6-trimethylpyridin-2-yl)-1-methyl-1*H*-pyrazole-3,4-diamine (23A)**

To a mixture of **22A** (100 mg, 0.22 mmol) in EtOH (5 mL) was added $SnCl_2 \cdot 2H_2O$ (223 mg, 0.99 mmol). The resulting mixture was refluxed for 3 h and then concentrated. The residue was diluted with EtOAc and saturated K_2CO_3, and the aqueous layer was extracted with EtOAc. The combined organic solution was dried over $MgSO_4$, filtered, and concentrated. The residue was purified by flash silica gel column chromatography with 5% MeOH/DCM to obtain **23A** (42 mg, 45%). Yellow solid; TLC R_f 0.45 (DCM/MeOH = 20/1); m.p. 135 °C; MS (EI) m/z 433 $[M]^+$; ^{1}H NMR (DMSO-d_6) δ 7.54 (s, 1H), 7.12–7.02 (m, 2H), 4.74 (s, 2H), 3.87 (s, 6H), 3.62 (s, 3H), 2.17 (s, 3H), 2.14 (s, 3H), 2.07 (s, 3H); ^{13}C NMR (DMSO-d_6) δ 150.0, 145.6 (d, J = 6.3 Hz), 144.7, 143.6, 143.3 (d, J = 4.7 Hz), 143.2 (d, J = 4.8 Hz), 141.8 (d, J = 6.2 Hz), 140.1, 139.5, 122.2, 119.5, 114.2, 113.6 (t, J = 18.2 Hz), 101.5, 61.9, 56.9 (2C), 38.3, 18.4, 13.0, 12.2; HRMS (EI) m/z calculated for $C_{21}H_{25}F_2N_5O_3$ $[M]^+$ 433.1925, found 433.1922.

N^3-(5-((2,6-Dichloro-3,5-dimethoxybenzyl)oxy)-3,4,6-trimethylpyridin-2-yl)-1-methyl-1*H*-pyrazole-3,4-diamine (23B)

To a mixture of **22B** (118 mg, 0.24 mmol) in EtOH (5 mL) was added $SnCl_2 \cdot 2H_2O$ (268 mg, 1.19 mmol). The resulting mixture was refluxed for 5 h and then concentrated. The residue was diluted with EtOAc and saturated K_2CO_3, and the aqueous layer was extracted with EtOAc. The combined organic solution was dried over $MgSO_4$, filtered, and concentrated. The residue was purified by flash silica gel column chromatography with 3% MeOH/DCM to obtain **23B** (60 mg, 53%). Brown solid; TLC R_f 0.68 (EtOAc/DCM = 1/3); m.p. 145 °C; MS (EI) *m*/*z* 465 $[M]^+$; ^{1}H NMR ($CDCl_3$) δ 6.95 (s, 1H), 6.59 (s, 1H), 6.27 (s, 1H), 5.11 (s, 2H), 3.92 (s, 6H), 3.70 (s, 3H), 3.03 (s, 2H), 2.38 (s, 3H), 2.24 (s, 3H), 2.11 (s, 3H); ^{13}C NMR ($CDCl_3$) δ 154.6 (2C), 149.4, 146.1, 145.6, 141.1, 140.8, 134.2, 121.5, 121.2, 116.7 (2C), 114.2, 97.8, 69.9, 56.7 (2C), 39.0, 19.7, 13.4, 13.2; HRMS (EI) *m*/*z* calculated for $C_{21}H_{25}Cl_2N_5O_3$ $[M]^+$ 465.1334, found 465.1334.

N^3-(5-((2,6-Difluoro-3,5-dimethoxybenzyl)oxy)-4,6-dimethylpyrimidin-2-yl)-1-methyl-1*H*-pyrazole-3,4-diamine (23C)

To a mixture of **22C** (90 mg, 0.20 mmol) in EtOH (5 mL) was added $SnCl_2 \cdot 2H_2O$ (226 mg, 1.0 mmol). The resulting mixture was refluxed for 12 h and then concentrated. The residue was diluted with EtOAc and saturated K_2CO_3, and the aqueous layer was extracted with EtOAc. The combined organic solution was dried over $MgSO_4$, filtered, and concentrated. The residue was purified by flash silica gel column chromatography with 20% to 50% DCM/hexanes to obtain **23C** (70 mg, 84%). Yellow solid; TLC R_f 0.23 (DCM/MeOH = 20/1); m.p. 218 °C; MS (EI) *m*/*z* 420 $[M]^+$; ^{1}H NMR (DMSO-d_6) δ 8.51 (s, 1H), 7.85 (s, 1H), 7.74 (s, 1H), 7.03 (t, *J* = 8.5 Hz, 1H), 4.74 (s, 2H), 3.86 (s, 6H), 3.69 (s, 3H), 2.12 (s, 6H); ^{13}C NMR (DMSO-d_6) δ 160.6 (2C), 160.5, 156.6, 156.0 (2C), 144.8, 143.2, 142.4, 142.1, 141.7, 138.0, 119.9, 113.2, 101.6, 62.0, 56.9, 56.8, 38.5, 18.3, 18.2; HRMS (EI) *m*/*z* calculated for $C_{19}H_{22}F_2N_6O_3$ $[M]^+$ 420.1721, found 420.1719.

N^3-(5-((2,6-Dichloro-3,5-dimethoxybenzyl)oxy)-4,6-dimethylpyrimidin-2-yl)-1-methyl-1*H*-pyrazole-3,4-diamine (23D)

To a mixture of **22D** (40 mg, 0.08 mmol) in EtOH (5.0 mL) was added $SnCl_2 \cdot 2H_2O$ (94 mg, 0.41 mmol). The resulting mixture was refluxed for 12 h and then concentrated. The residue was diluted with EtOAc and saturated K_2CO_3, and the aqueous layer was extracted with EtOAc. The combined organic solution was dried over $MgSO_4$, filtered, and concentrated. The residue dissolved in a small amount of $CHCl_3$ then hexanes was added to get a precipitate. The precipitate was filtered, washed with hexanes to obtain **23D** (36 mg, 94%). Yellow solid; TLC R_f 0.14 (DCM/MeOH = 20/1); m.p. 255 °C; MS (EI) *m/z* 452 $[M]^+$; ^1H NMR ($CDCl_3$) δ 6.97 (s, 1H), 6.87 (s, 1H), 6.61 (s, 1H), 5.16 (s, 2H), 3.93 (s, 6H), 3.72 (s, 3H), 2.36 (s, 6H); ^{13}C NMR ($CDCl_3$) δ 161.8 (2C), 155.8, 154.8 (2C), 143.7, 139.4, 133.7, 121.7, 121.5 (2C), 116.8, 98.1, 69.9, 56.8 (2C), 39.2, 19.5 (2C); HRMS (EI) *m/z* calculated for $C_{19}H_{22}Cl_2N_6O_3$ $[M]^+$ 452.1130, found 452.1122.

***N^1^*-(5-((2,6-Difluoro-3,5-dimethoxybenzyl)oxy)-3,4,6-trimethylpyridin-2-yl)-6-methylbenzene-1,2-diamine (23E)**

To a mixture of **22E** (180 mg, 0.38 mmol) in EtOH (5 mL) was added $SnCl_2 \cdot 2H_2O$ (429 mg, 1.90 mmol). The resulting mixture was refluxed for 12 h and then concentrated. The residue was diluted with EtOAc and saturated K_2CO_3, and the aqueous layer was extracted with EtOAc. The combined organic solution was dried over $MgSO_4$, filtered, and concentrated. The residue was purified by flash silica gel column chromatography with 50% EtOAc/Hexanes to obtain **23E** (86 mg, 51%). Beige solid; TLC R_f 0.17 (EtOAc/Hexanes = 1/2); m.p. 190 °C; MS (EI) *m/z* 443 $[M]^+$; ^1H NMR ($CDCl_3$) δ 6.96 (t, *J* = 7.7 Hz, 1H), 6.73–6.62 (m, 3H), 5.55 (s, 1H), 4.80 (s, 2H), 3.90 (s, 6H), 2.33 (s, 3H), 2.28 (s, 3H), 2.17 (s, 3H), 2.12 (s, 3H); ^{13}C NMR ($CDCl_3$) δ 150.9, 147.4 (d, *J* = 6.1 Hz), 146.6, 144.7, 144.1, 143.8 (d, *J* = 4.9 Hz), 143.6 (d, *J* = 4.6 Hz), 140.5, 134.6, 127.2, 126.1, 120.8 (2C), 115.0, 114.4 (t, *J* = 18.2 Hz), 114.0, 102.0, 62.3, 57.6 (2C), 19.0, 18.8, 13.1, 12.7; HRMS (EI) *m/z* calculated for $C_{24}H_{27}F_2N_3O_3$ $[M]^+$ 443.2020, found 443.2015.

N^1-(5-((2,6-Dichloro-3,5-dimethoxybenzyl)oxy)-3,4,6-trimethylpyridin-2-yl)-6-methylbenzene-1,2-diamine (23F)

To a mixture of **22F** (90 mg, 0.18 mmol) in EtOH (5 mL) was added $SnCl_2 \cdot 2H_2O$ (201 mg, 0.89 mmol). The resulting mixture was refluxed for 1.5 h and then concentrated. The residue was diluted with EtOAc and saturated K_2CO_3, and the aqueous layer was extracted with EtOAc.

The combined organic solution was dried over $MgSO_4$, filtered, and concentrated. The residue was dissolved in a small amount of EtOAc then hexanes was added to get a precipitate. The precipitate was filtered, washed with hexanes to afford **23F** (72 mg, 85%). Brown solid; TLC R_f 0.53 (DCM/MeOH = 20/1); m.p. 201 °C; MS (EI) *m/z* 475 $[M]^+$; ^1H NMR ($CDCl_3$) δ 6.96 (t, *J* = 7.7 Hz, 1H), 6.67 (dd, *J* = 7.6, 2.4 Hz, 2H), 6.59 (s, 1H), 5.53 (s, 1H), 5.11 (s, 2H), 4.10 (br, s, 1H), 3.93 (s, 6H), 2.31 (s, 3H), 2.25 (s, 3H), 2.17 (s, 3H), 2.10 (s, 3H); ^{13}C NMR ($CDCl_3$) δ 154.6 (2C), 150.6, 146.4, 145.2, 144.0, 140.6, 134.5, 134.3, 127.1, 126.0, 120.7, 116.6, 114.9 (2C), 113.8, 97.8, 69.8, 56.7 (2C), 19.7, 18.8, 13.4, 13.1; HRMS (EI) *m/z* calculated for $C_{24}H_{27}Cl_2N_3O_3$ $[M]^+$ 475.1429, found 475.1425.

***N^1*-(5-((2,6-Difluoro-3,5-dimethoxybenzyl)oxy)-4,6-dimethylpyrimidin-2-yl)-6-methylbenzene-1,2-diamine (23G)**

To a mixture of **22G** (115 mg, 0.25 mmol) in EtOH (5 mL) was added $SnCl_2{\cdot}2H_2O$ (226 mg, 1.0 mmol). The resulting mixture was refluxed for 6 h and then concentrated. The residue was diluted with DCM and saturated K_2CO_3, and the aqueous layer was extracted with EtOAc. The combined organic solution was dried over $MgSO_4$, filtered, and concentrated. The residue was purified by flash silica gel column chromatography with 30% to 60% EtOAc/Hexanes to obtain **23G** (45 mg, 41%). Pale yellow solid; TLC R_f 0.33 (Hexanes/EtOAc = 1/1); m.p. 248 °C; MS (ESI) *m/z* 431 $[M+H]^+$; ^1H NMR ($CDCl_3$) δ 7.00 (t, *J* = 7.7 Hz, 1H), 6.68 (t, *J* = 8.3 Hz, 3H), 6.33 (s, 1H), 4.85 (t, *J* = 1.8 Hz, 2H), 3.89 (s, 6H), 2.34 (s, 6H), 2.19 (s, 3H); ^{13}C NMR ($CDCl_3$) δ 161.9 (2C), 156.9, 147.2 (d, *J* = 6.0 Hz), 144.1, 143.9–143.2 (m), 142.9, 136.4, 127.2 (2C), 124.4, 120.6 (2C), 114.5, 113.9 (t, *J* = 18.0 Hz), 102.2, 62.5, 57.5 (2C), 18.9 (2C), 18.8.

***N^1*-(5-((2,6-Dichloro-3,5-dimethoxybenzyl)oxy)-4,6-dimethylpyrimidin-2-yl)-6-methylbenzene-1,2-diamine (23H)**

To a mixture of **22H** (70 mg, 0.14 mmol) in EtOH (5 mL) was added $SnCl_2{\cdot}2H_2O$ (160 mg, 0.71 mmol). The resulting mixture was refluxed for 9 h and then concentrated. The residue was diluted with DCM and saturated K_2CO_3, and the aqueous layer was extracted with EtOAc. The combined organic solution was dried over $MgSO_4$, filtered, and concentrated. The residue was purified by flash silica gel column chromatography with 30% to 70% EtOAc/Hexanes to obtain **23H** (58 mg, 88%). Pale yellow solid; TLC R_f 0.40 (Hexanes/EtOAc = 1/1); m.p. 228 °C; MS (EI) *m/z* 462 $[M]^+$; ^1H NMR ($CDCl_3$) δ 7.77 (d, *J* = 8.3 Hz, 1H), 7.30 (d, *J* = 7.2 Hz, 1H), 7.22–

7.13 (m, 1H), 6.58 (s, 1H), 5.27 (s, 2H), 3.90 (s, 6H), 2.87 (s, 3H), 2.76 (s, 3H), 2.71 (s, 3H); ^{13}C NMR ($CDCl_3$) δ 162.2, 154.8 (2C), 148.8, 144.8, 140.3, 138.8, 132.9, 130.4, 128.4, 125.5, 120.9, 116.5 (2C), 111.7, 98.1, 71.0, 56.7 (2C), 21.7, 17.2, 14.1; HRMS (EI) *m*/*z* calculated for $C_{22}H_{24}Cl_2N_4O_3$ $[M]^+$ 462.1225, found 462.1222.

***N*^{1}-(5-((2,6-Difluoro-3,5-dimethoxybenzyl)oxy)-3,4,6-trimethylpyridin-2-yl)-2-methylbenzene-1,4-diamine (23I)**

To a mixture of **22I** (83 mg, 0.18 mmol) in EtOH (5 mL) was added $SnCl_2·2H_2O$ (198 mg, 0.88 mmol). The resulting mixture was refluxed for 3 h and then concentrated. The residue was diluted with DCM and saturated K_2CO_3, and the aqueous layer was extracted with EtOAc. The combined organic solution was dried over $MgSO_4$, filtered, and concentrated. The residue was purified by flash silica gel column chromatography with 20% EtOAc/DCM to obtain **23I** (49 mg, 63%). Brown solid; TLC R_f 0.20 (DCM/MeOH = 20/1); m.p. 180 °C; MS (EI) *m*/*z* 443 $[M]^+$; ^{1}H NMR ($CDCl_3$) δ 7.22 (d, *J* = 8.4 Hz, 1H), 6.68 (t, *J* = 8.1 Hz, 1H), 6.62–6.41 (m, 2H), 5.60 (s, 1H), 4.82 (s, 2H), 3.90 (s, 6H), 3.52 (s, 1H), 2.41 (s, 3H), 2.27 (s, 3H), 2.20 (s, 3H), 2.02 (s, 3H); ^{13}C NMR ($CDCl_3$) δ 150.7 (2C), 147.5, 146.7, 145.5 (2C), 143.8, 141.6, 140.4, 132.6, 130.4, 122.4, 117.7, 116.2, 114.5, 113.4, 102.1, 62.2, 57.6 (2C), 19.0, 18.4, 13.7, 12.7; HRMS (EI) *m*/*z* calculated for $C_{24}H_{27}F_2N_3O_3$ $[M]^+$ 443.2020, found 443.2015.

***N*^{1}-(5-((2,6-Dichloro-3,5-dimethoxybenzyl)oxy)-3,4,6-trimethylpyridin-2-yl)-2-methylbenzene-1,4-diamine (23J)**

To a mixture of **22J** (74 mg, 0.16 mmol) in EtOH (5 mL) was added $SnCl_2·2H_2O$ (176 mg, 0.78 mmol). The resulting mixture was refluxed for 5 h and then concentrated. The residue was diluted with DCM and saturated K_2CO_3, and the aqueous layer was extracted with EtOAc. The combined organic solution was dried over $MgSO_4$, filtered, and concentrated. The residue was purified by flash silica gel column chromatography with 30% to 40% EtOAc/DCM to obtain **23J** (52 mg, 70%). Yellow solid; TLC R_f 0.34 (EtOAc/DCM = 1/1); m.p. 191 °C; MS (EI) *m*/*z* 475 $[M]^+$; ^{1}H NMR ($CDCl_3$) δ 7.18 (d, *J* = 8.4 Hz, 1H), 6.60 (s, 1H), 6.57–6.44 (m, 2H), 5.57 (s, 1H), 5.14 (s, 2H), 3.92 (s, 6H), 3.50 (br, s, 2H), 2.38 (s, 3H), 2.23 (s, 3H), 2.20 (s, 3H), 1.99 (s, 3H); ^{13}C NMR ($CDCl_3$) δ 154.7 (2C), 150.5, 146.6, 146.1, 141.4, 140.5, 134.4, 132.8, 130.2, 122.1, 117.7, 116.8, 116.4, 113.4 (2C), 98.0, 69.8, 56.8 (2C), 19.7, 18.4, 13.7, 13.4; HRMS (EI) *m*/*z* calculated for $C_{24}H_{27}Cl_2N_3O_3$ $[M]^+$ 475.1429, found 475.1436.

N^1-(5-((2,6-Difluoro-3,5-dimethoxybenzyl)oxy)-4,6-dimethylpyrimidin-2-yl)-2-methylbenzene-1,4-diamine (23K)

To a mixture of **22K** (100 mg, 0.22 mmol) in EtOH (5 mL) was added $SnCl_2 \cdot 2H_2O$ (245 mg, 1.09 mmol). The resulting mixture was refluxed for 5 h and then concentrated. The residue was diluted with DCM and saturated K_2CO_3, and the aqueous layer was extracted with EtOAc. The combined organic solution was dried over $MgSO_4$, filtered, and concentrated. The residue was purified by flash silica gel column chromatography with 50% EtOAc/Hexanes to obtain **23K** (81 mg, 87%). Grey solid; TLC R_f 0.42 (DCM/MeOH = 20/1); m.p. 188 °C; MS (EI) *m*/*z* 430 $[M]^+$; ^1H NMR ($CDCl_3$) δ 7.59–7.49 (m, 1H), 6.68 (t, *J* = 8.2 Hz, 1H), 6.61 (s, 1H), 6.55 (s, 2H), 4.85 (s, 2H), 3.88 (s, 6H), 3.62 (s, 2H), 2.36 (s, 6H), 2.19 (s, 3H); ^{13}C NMR ($CDCl_3$) δ 161.3, 156.6 (2C), 147.2 (d, *J* = 5.8 Hz), 143.7 (d, *J* = 4.9 Hz), 143.6 (d, *J* = 4.9 Hz), 143.3 (d, *J* = 5.9 Hz), 143.0, 142.7, 132.0, 129.4, 124.6, 117.3, 113.9 (t, *J* = 18.0 Hz), 113.3, 102.1, 62.4, 57.5 (2C), 18.8 (2C), 18.4; HRMS (EI) *m*/*z* calculated for $C_{22}H_{24}F_2N_4O_3$ $[M]^+$ 430.1816, found 430.1811.

N^1-(5-((2,6-Dichloro-3,5-dimethoxybenzyl)oxy)-4,6-dimethylpyrimidin-2-yl)-2-methylbenzene-1,4-diamine (23L)

To a mixture of **22L** (80 mg, 0.16 mmol) in EtOH (5 mL) was added $SnCl_2 \cdot 2H_2O$ (183 mg, 0.81 mmol). The resulting mixture was refluxed for 12 h and then concentrated. The residue was diluted with EtOAc and saturated K_2CO_3, and the aqueous layer was extracted with EtOAc. The combined organic solution was dried over $MgSO_4$, filtered, and concentrated. The residue was purified by flash silica gel column chromatography with 5% to 7% MeOH/DCM to obtain **23L** (49 mg, 62%). Pale yellow solid; TLC R_f 0.48 (DCM/MeOH = 20/1); m.p. 225 °C; MS (EI) *m*/*z* 462 $[M]^+$; ^1H NMR ($CDCl_3$) δ 7.56 (d, *J* = 9.2 Hz, 1H), 6.59 (s, 1H), 6.57–6.50 (m, 2H), 6.47 (s, 1H), 5.15 (s, 2H), 3.92 (s, 6H), 3.51 (s, 2H), 2.33 (s, 6H), 2.19 (s, 3H); ^{13}C NMR ($CDCl_3$) δ 161.2 (2C), 156.6, 154.7 (2C), 143.2, 142.8, 133.8, 131.7, 129.6, 124.4, 117.3, 116.7, 113.3 (2C), 98.1, 69.8, 56.7 (2C), 19.4 (2C), 18.4; HRMS (EI) *m*/*z* calculated for $C_{22}H_{24}Cl_2N_4O_3$ $[M]^+$ 462.1225, found 462.1225.

6-Chloro-N^1-(5-((2,6-difluoro-3,5-dimethoxybenzyl)oxy)-3,4,6-trimethylpyridin-2-yl)-4-fluorobenzene-1,2-diamine (23M)

To a mixture of **22M** (100 mg, 0.20 mmol) in EtOH (5 mL) was added $SnCl_2\cdot 2H_2O$ (221 mg, 0.98 mmol). The resulting mixture was refluxed for 6 h and then concentrated. The residue was diluted with EtOAc and saturated K_2CO_3, and the aqueous layer was extracted with EtOAc. The combined organic solution was dried over $MgSO_4$, filtered, and concentrated. The residual solid was washed with a small amount of $CHCl_3$ and MeOH to obtain **23M** (92 mg, 98%). White solid; TLC R_f 0.27 (EtOAc/Hexanes = 1/2); m.p. 250 °C; MS (EI) *m*/*z* 481 $[M]^+$; ^{1}H NMR (DMSO-d_6) δ 7.07 (t, *J* = 8.5 Hz, 1H), 6.82 (s, 1H), 6.58–6.35 (m, 1H), 5.30 (s, 2H), 4.70 (s, 2H), 3.87 (s, 6H), 2.17 (s, 3H), 2.13 (s, 3H), 2.09 (s, 3H); ^{13}C NMR (DMSO-d_6) δ 158.2, 151.1, 149.1 (d, *J* = 13.2 Hz), 145.6 (d, *J* = 5.1 Hz), 144.8, 143.8, 143.4 (d, *J* = 3.9 Hz), 143.2 (d, *J* = 4.8 Hz), 141.8 (d, *J* = 6.2 Hz), 138.8, 133.7 (d, *J* = 14.4 Hz), 120.8, 114.0, 113.7, 102.6 (d, *J* = 26.0 Hz), 101.5, 99.2 (d, *J* = 24.9 Hz), 61.8, 56.9 (2C), 18.7, 12.9, 12.1; HRMS (EI) *m*/*z* calculated for $C_{25}H_{26}Cl_2N_4O_4$ $[M]^+$ 481.1380, found 481.1374.

6-Chloro-*N*^{1}-(5-((2,6-dichloro-3,5-dimethoxybenzyl)oxy)-3,4,6-trimethylpyridin-2-yl)-4-fluorobenzene-1,2-diamine (23N)

To a mixture of **22N** (140 mg, 0.26 mmol) in EtOH (5 mL) was added $SnCl_2\cdot 2H_2O$ (294 mg, 1.30 mmol). The resulting mixture was refluxed for 5 h and then concentrated. The residue was diluted with EtOAc and saturated K_2CO_3, and the aqueous layer was extracted with EtOAc. The combined organic solution was dried over $MgSO_4$, filtered, and concentrated. The residual solid was washed with a small amount of EtOAc and acetone to obtain **23N** (108 mg, 81%). White solid; TLC R_f 0.57 (DCM/MeOH = 20/1); m.p. 267 °C; MS (EI) *m*/*z* 515 $[M]^+$; ^{1}H NMR (DMSO-d_6) δ 6.98 (s, 1H), 6.77 (s, 1H), 6.53–6.40 (m, 2H), 5.27 (s, 2H), 4.98 (s, 2H), 3.93 (s, 6H), 2.15 (s, 3H), 2.12 (s, 3H), 2.09 (s, 3H); ^{13}C NMR (DMSO-d_6) δ 158.0, 154.2 (2C), 150.5, 148.9, 148.7, 138.8, 133.3, 133.2, 120.8, 115.0 (2C), 113.8, 102.7, 102.3, 99.4, 99.1, 99.0, 69.1, 56.6 (2C), 19.1, 12.7, 12.6; HRMS (EI) *m*/*z* calculated for $C_{25}H_{26}Cl_2N_4O_4$ $[M]^+$ 513.0789, found 513.0788.

6-Chloro-*N*^{1}-(5-((2,6-difluoro-3,5-dimethoxybenzyl)oxy)-4,6-dimethylpyrimidin-2-yl)-4-fluorobenzene-1,2-diamine (23O)

To a mixture of **22O** (120 mg, 0.24 mmol) in EtOH (3 mL) was added $SnCl_2\cdot 2H_2O$ (272 mg, 1.20 mmol). The resulting mixture was refluxed for 12 h and then concentrated. The residue was diluted with EtOAc and saturated K_2CO_3, and the aqueous layer was extracted with EtOAc.

The combined organic solution was dried over $MgSO_4$, filtered, and concentrated. The residual solid was purified by washing with EtOAc/hexanes to obtain **23O** (101 mg, 89%). Grey solid; TLC R_f 0.48 (DCM/MeOH = 20/1); m.p. 260 °C; MS (EI) *m/z* 468 $[M]^+$; ^{1}H NMR (DMSO-d_6) δ 8.04 (s, 1H), 7.07 (t, *J* = 7.9 Hz, 1H), 6.61–6.36 (m, 2H), 5.37 (s, 2H), 4.81 (s, 2H), 3.87 (s, 6H), 2.18 (s, 6H); ^{13}C NMR (DMSO-d_6) δ 162.4, 160.2, 158.5, 157.0, 148.8 (d, *J* = 13.5 Hz), 145.6 (d, *J* = 6.0 Hz), 143.4 (d, *J* = 4.6 Hz), 143.2 (d, *J* = 4.2 Hz), 142.0, 141.7, 134.0 (d, *J* = 14.6 Hz), 118.7, 113.3 (t, *J* = 18.3 Hz), 102.7 (d, *J* = 26.2 Hz), 101.7, 99.3 (d, *J* = 25.0 Hz), 61.9, 56.9 (2C), 18.3 (2C); HRMS (EI) *m/z* calculated for $C_{21}H_{20}ClF_3N_4O_3$ $[M]^+$ 468.1176, found 468.1177.

6-Chloro-*N*^{1}-(5-((2,6-dichloro-3,5-dimethoxybenzyl)oxy)-4,6-dimethylpyrimidin-2-yl)-4-fluorobenzene-1,2-diamine (23P)

To a mixture of **22P** (50 mg, 0.09 mmol) in EtOH (3 mL) was added $SnCl_2·2H_2O$ (106 mg, 0.47 mmol). The resulting mixture was refluxed for 24 h and then concentrated. The residue was diluted with EtOAc and saturated K_2CO_3, and the aqueous layer was extracted with EtOAc. The combined organic solution was dried over $MgSO_4$, filtered, and concentrated. The residual solid was purified by washing with EtOAc/hexanes to obtain **23P** (41 mg, 87%). White solid; TLC R_f 0.32 (DCM/MeOH = 20/1); m.p. 275 °C; MS (EI) *m/z* 500 $[M]^+$; ^{1}H NMR (DMSO-d_6) δ 8.00 (s, 1H), 6.98 (s, 1H), 6.56–6.40 (m, 2H), 5.36 (s, 2H), 5.05 (s, 2H), 3.93 (s, 6H), 2.15 (s, 6H); ^{13}C NMR (DMSO-d_6) δ 160.1 (2C), 156.8, 154.4, 154.3 (2C), 148.8 (d, *J* = 13.4 Hz), 142.3, 134.0 (d, *J* = 14.5 Hz), 132.8, 118.7, 115.0 (2C), 102.7 (d, *J* = 26.3 Hz), 99.5, 99.1, 69.2, 56.7 (2C), 18.7 (2C); HRMS (EI) *m/z* calculated for $C_{21}H_{20}Cl_3FN_4O_3$ $[M]^+$ 500.0585, found 500.0568.

***N*^{1}-(5-((2,6-Difluoro-3,5-dimethoxybenzyl)oxy)-3,4,6-trimethylpyridin-2-yl)-6-methoxybenzene-1,2-diamine (23Q)**

To a mixture of **22Q** (112 mg, 0.23 mmol) in EtOH (5 mL) was added $SnCl_2·2H_2O$ (258 mg, 1.14 mmol). The resulting mixture was refluxed for 12 h and then concentrated. The residue was diluted with EtOAc and saturated K_2CO_3, and the aqueous layer was extracted with EtOAc. The combined organic solution was dried over $MgSO_4$, filtered, and concentrated. The residue was dissolved in a small amount of EtOAc then hexanes was added to get a precipitate. The precipitate was filtered, washed with hexanes to obtain **23Q** (81 mg, 77%). White solid; TLC

R_f 0.67 (DCM/MeOH = 20/1); m.p. 220 °C; MS (EI) *m/z* 459 $[M]^+$; ^{1}H NMR ($CDCl_3$) δ 6.95 (t, *J* = 8.1 Hz, 1H), 6.68 (t, *J* = 8.1 Hz, 1H), 6.49 (d, *J* = 8.0 Hz, 1H), 6.40 (d, *J* = 8.1 Hz, 1H), 6.20 (s, 1H), 4.78 (d, *J* = 1.7 Hz, 2H), 4.54 (s, 2H), 3.89 (s, 6H), 3.80 (s, 3H), 2.38 (s, 3H), 2.29 (s, 3H), 2.21 (s, 3H); ^{13}C NMR ($CDCl_3$) δ 152.9, 151.1, 147.3 (d, *J* = 6.1 Hz), 146.1, 144.8, 143.7 (d, *J* = 4.9 Hz), 143.6 (d, *J* = 4.9 Hz), 143.4, 143.3, 140.4, 124.9, 118.8, 115.3, 114.4 (t, *J* = 18.2 Hz), 111.3, 102.0, 101.3, 62.3, 57.5 (2C), 55.9, 19.0, 13.2, 12.7; HRMS (EI) *m/z* calculated for $C_{24}H_{27}F_2N_3O_4$ $[M]^+$ 459.1970, found 459.1966.

***N*¹-(5-((2,6-Dichloro-3,5-dimethoxybenzyl)oxy)-3,4,6-trimethylpyridin-2-yl)-6-methoxybenzene-1,2-diamine (23R)**

To a mixture of **22R** (120 mg, 0.23 mmol) in EtOH (5 mL) was added $SnCl_2{\cdot}2H_2O$ (259 mg, 1.15 mmol). The resulting mixture was refluxed for 15 h and then concentrated. The residue was diluted with EtOAc and saturated K_2CO_3, and the aqueous layer was extracted with EtOAc. The combined organic solution was dried over $MgSO_4$, filtered, and concentrated. The residue was dissolved in a small amount of EtOAc then hexanes was added to get a precipitate. The precipitate was filtered, washed with hexanes to obtain **23R** (109 mg, 96%). White solid; TLC R_f 0.50 (DCM/MeOH = 20/1); m.p. 238 °C; MS (EI) *m/z* 491 $[M]^+$; ^{1}H NMR ($CDCl_3$) δ 6.94 (t, *J* = 8.1 Hz, 1H), 6.60 (s, 1H), 6.48 (dd, *J* = 8.0, 1.0 Hz, 1H), 6.40 (dd, *J* = 8.2, 1.0 Hz, 1H), 6.18 (s, 1H), 5.10 (s, 2H), 4.52 (s, 2H), 3.93 (s, 6H), 3.80 (s, 3H), 2.37 (s, 3H), 2.27 (s, 3H), 2.20 (s, 3H); ^{13}C NMR ($CDCl_3$) δ 154.7 (2C), 152.9, 150.9, 146.1, 145.4, 143.2, 140.5, 134.4, 124.7, 118.9, 116.8, 115.2, 111.3 (2C), 101.4, 98.0, 69.9, 56.8 (2C), 55.9, 19.8, 13.4, 13.2; HRMS (EI) *m/z* calculated for $C_{24}H_{27}Cl_2N_3O_4$ $[M]^+$ 491.1379, found 491.1377.

***N*¹-(5-((2,6-Difluoro-3,5-dimethoxybenzyl)oxy)-4,6-dimethylpyrimidin-2-yl)-6-methoxybenzene-1,2-diamine (23S)**

To a mixture of **22S** (120 mg, 0.25 mmol) in EtOH (10 mL) was added $SnCl_2{\cdot}2H_2O$ (284 mg, 1.26 mmol). The resulting mixture was refluxed for 5 h and then concentrated. The residue was diluted with DCM and saturated K_2CO_3, and the aqueous layer was extracted with EtOAc. The combined organic solution was dried over $MgSO_4$, filtered, and concentrated. The residue was purified by silica gel column chromatography with 50% EtOAc/Hexanes to obtain **23S** (79 mg, 70%). White solid; TLC R_f 0.40 (DCM/MeOH = 20/1); m.p. 194 °C; MS (EI) *m/z* 446 $[M]^+$; ^{1}H NMR ($CDCl_3$) δ 6.99 (t, *J* = 8.1 Hz, 1H), 6.74–6.61 (m, 2H), 6.45 (d, *J* = 8.1 Hz, 1H), 6.38

(dd, J = 8.2, 0.8 Hz, 1H), 4.83 (t, J = 1.7 Hz, 2H), 3.88 (s, 6H), 3.78 (s, 3H), 2.37 (s, 6H); ^{13}C NMR ($CDCl_3$) δ 168.9, 161.7, 157.2, 154.2 (2C), 147.2 (d, J = 5.9 Hz), 143.8 (d, J = 4.4 Hz)(2C), 143.6 (d, J = 4.9 Hz), 143.3 (d, J = 5.7 Hz), 143.2, 126.2, 116.1, 113.9 (t, J = 18.0 Hz), 110.6, 102.2, 101.3, 62.4, 57.5 (2C), 55.8, 18.9 (2C); HRMS (EI) *m/z* calculated for $C_{22}H_{24}F_2N_4O_4$ $[M]^+$ 446.1766, found 446.1758.

N^1-(5-((2,6-Dichloro-3,5-dimethoxybenzyl)oxy)-4,6-dimethylpyrimidin-2-yl)-6-methoxybenzene-1,2-diamine (23T)

To a mixture of **22T** (120 mg, 0.24 mmol) in EtOH (10 mL) was added $SnCl_2{\cdot}2H_2O$ (266 mg, 1.18 mmol). The resulting mixture was refluxed for 5 h and then concentrated. The residue was diluted with DCM and saturated K_2CO_3, and the aqueous layer was extracted with EtOAc. The combined organic solution was dried over $MgSO_4$, filtered, and concentrated. The residue was purified by silica gel column chromatography with 50% EtOAc/Hexanes to obtain **23T** (72 mg, 63%). Ivory solid; TLC R_f 0.44 (DCM/MeOH = 20/1); m.p. 212 °C; MS (EI) *m/z* 478 $[M]^+$; ^1H NMR ($CDCl_3$) δ 6.98 (t, J = 8.1 Hz, 1H), 6.61 (s, 2H), 6.45 (dd, J = 8.1, 1.0 Hz, 1H), 6.37 (dd, J = 8.2, 1.0 Hz, 1H), 5.14 (s, 2H), 3.92 (s, 6H), 3.79 (s, 3H), 2.36 (s, 6H); ^{13}C NMR ($CDCl_3$) δ 161.6, 157.1, 154.8 (2C), 154.3 (2C), 143.8, 143.6, 133.8, 126.2 (2C), 116.9, 116.3, 110.6, 101.4, 98.3, 69.9, 56.8 (2C), 55.8, 19.5 (2C); HRMS (EI) *m/z* calculated for $C_{22}H_{24}Cl_2N_4O_3$ $[M]^+$ 478.1175, found 478.1178.

N^1-(5-((2,6-Difluoro-3,5-dimethoxybenzyl)oxy)-3,4,6-trimethylpyridin-2-yl)-5-methoxybenzene-1,2-diamine (23U)

To a mixture of **22U** (90 mg, 0.18 mmol) in EtOH (5 mL) was added $SnCl_2{\cdot}2H_2O$ (226 mg, 0.92 mmol). The resulting mixture was refluxed for 5 h and then concentrated. The residue was diluted with EtOAc and saturated K_2CO_3, and the aqueous layer was extracted with EtOAc. The combined organic solution was dried over $MgSO_4$, filtered, and concentrated. The residue was dissolved in a small amount of $CHCl_3$ then hexanes was added to get a precipitate. The precipitate was filtered, washed with hexanes to obtain **23U** (71 mg, 84%). Grey solid; TLC R_f 0.55 (DCM/MeOH = 9/1); m.p. 138 °C; MS (EI) *m/z* 459 $[M]^+$; ^1H NMR ($CDCl_3$) δ 7.17 (d, J = 2.7 Hz, 1H), 6.85–6.63 (m, 2H), 6.47 (dd, J = 8.4, 2.8 Hz, 1H), 6.19 (s, 1H), 4.84 (s, 1H), 3.90 (s, 6H), 3.74 (s, 3H), 2.89 (s, 2H), 2.44 (s, 3H), 2.29 (s, 3H), 2.11 (s, 3H); ^{13}C NMR ($CDCl_3$) δ 154.3, 149.9, 147.4 (d, J = 5.8 Hz), 146.8, 145.8, 143.8 (d, J = 4.8 Hz), 143.6 (d, J

= 5.0 Hz), 143.5, 140.7, 132.9, 130.9, 119.3, 116.7, 114.4, 108.1, 106.8, 102.1, 62.3 (t, *J* = 3.5 Hz), 57.6, 55.8 (2C), 19.1, 13.6, 12.8; HRMS (EI) *m*/*z* calculated for $C_{24}H_{27}F_2N_3O_4$ $[M]^+$ 459.1970, found 459.1966.

***N*[1]-(5-((2,6-Dichloro-3,5-dimethoxybenzyl)oxy)-3,4,6-trimethylpyridin-2-yl)-5-methoxybenzene-1,2-diamine (23V)**

To a mixture of **22V** (40 mg, 0.08 mmol) in EtOH (5 mL) was added $SnCl_2\cdot 2H_2O$ (57 mg, 0.38 mmol). The resulting mixture was refluxed for 12 h and then concentrated. The residue was diluted with EtOAc and saturated K_2CO_3, and the aqueous layer was extracted with EtOAc. The combined organic solution was dried over $MgSO_4$, filtered, and concentrated. The residue was dissolved in a small amount of EtOAc then hexanes was added to get a precipitate. The precipitate was filtered, washed with hexane to obtain **23V** (35 mg, 94%). Green solid; TLC R_f 0.42 (DCM/MeOH = 20/1); m.p. 108 °C; MS (EI) *m*/*z* 491 $[M]^+$; ^1H NMR ($CDCl_3$) δ 7.13 (d, *J* = 2.7 Hz, 1H), 6.73 (d, *J* = 8.5 Hz, 1H), 6.60 (s, 1H), 6.45 (dd, *J* = 8.5, 2.7 Hz, 1H), 6.18 (s, 1H), 5.16 (s, 2H), 3.92 (s, 6H), 3.73 (s, 3H), 3.39 (s, 1H), 2.42 (s, 3H), 2.23 (s, 3H), 2.07 (s, 3H); ^{13}C NMR ($CDCl_3$) δ 154.7 (2C), 154.3, 149.6, 146.7, 146.4, 140.9, 134.3, 132.9, 130.9, 119.1, 116.8, 116.8 (2C), 108.0, 106.6, 98.0, 69.8, 56.8 (2C), 55.7, 19.7, 13.6, 13.5; HRMS (EI) *m*/*z* calculated for $C_{24}H_{27}Cl_2N_3O_4$ $[M]^+$ 491.1379, found 491.1382.

***N*[1]-(5-((2,6-Difluoro-3,5-dimethoxybenzyl)oxy)-4,6-dimethylpyrimidin-2-yl)-5-methoxybenzene-1,2-diamine (23W)**

To a mixture of **22W** (90 mg, 0.19 mmol) in EtOH (10 mL) was added $SnCl_2\cdot 2H_2O$ (213 mg, 0.95 mmol). The resulting mixture was refluxed for 12 h and then concentrated. The residue was diluted with DCM and saturated K_2CO_3, and the aqueous layer was extracted with EtOAc. The combined organic solution was dried over $MgSO_4$, filtered, and concentrated. The residual solid was purified by washing with MeOH to obtain **23W** (76 mg, 90%). Yellow solid; TLC R_f 0.37 (DCM/MeOH = 20/1); m.p. 255 °C; MS (ESI) *m*/*z* 449 $[M+H]^+$; ^1H NMR ($CDCl_3$) δ 7.86 (d, *J* = 9.0 Hz, 1H), 7.42 (dd, *J* = 23.4, 2.4 Hz, 1H), 7.18 (dd, *J* = 9.0, 2.4 Hz, 1H), 6.70 (t, *J* = 8.2 Hz, 1H), 4.98 (s, 2H), 3.91 (s, 3H), 3.89 (s, 6H), 2.98 (s, 3H), 2.69 (s, 3H); ^{13}C NMR ($CDCl_3$) δ 161.6, 158.4, 155.1 (2C), 149.0, δ 143.8 (dd, *J* = 10.3, 5.0 Hz), 140.3, 139.8, 138.3, 129.0, 121.0 (2C), 114.7, 112.0, 102.2, 98.4, 63.8, 57.5 (2C), 56.3, 21.1, 13.5.

***N*^{1}-(5-((2,6-Dichloro-3,5-dimethoxybenzyl)oxy)-4,6-dimethylpyrimidin-2-yl)-5-methoxybenzene-1,2-diamine (23X)**

To a mixture of **22X** (80 mg, 0.16 mmol) in EtOH (5 mL) was added $SnCl_2 \cdot 2H_2O$ (177 mg, 0.79 mmol). The resulting mixture was refluxed for 24 h and then concentrated. The residue was diluted with DCM and saturated K_2CO_3, and the aqueous layer was extracted with EtOAc. The combined organic solution was dried over $MgSO_4$, filtered, and concentrated. The residue was purified by silica gel column chromatography with 5% to 10% MeOH/DCM to obtain **23X** (68 mg, 90%). Yellow solid; TLC R_f 0.38 (DCM/MeOH = 20/1); m.p. 241 °C; MS (EI) *m/z* 478 $[M]^+$; ^{1}H NMR ($CDCl_3$) δ 7.77 (dd, *J* = 19.7, 9.1 Hz, 1H), 7.33 (dd, *J* = 11.5, 2.3 Hz, 1H), 6.98 (ddd, *J* = 71.5, 9.1, 2.4 Hz, 1H), 6.54 (s, 1H), 5.22 (s, 2H), 3.87 (s, 9H), 2.81 (s, 3H), 2.69 (s, 3H); ^{13}C NMR ($CDCl_3$) δ 161.2, 154.7 (2C), 149.6, 147.1, 139.9, 132.9, 128.9, 123.1, 120.7, 116.4 (2C), 114.9, 111.7, 101.1, 98.0, 71.0, 56.7 (2C), 55.7, 21.5, 14.1.

***N*^{1}-(5-((2,6-Difluoro-3,5-dimethoxybenzyl)oxy)-3,4,6-trimethylpyridin-2-yl)-5-(4-ethylpiperazin-1-yl)benzene-1,2-diamine (23Y)**

To a mixture of **22Y** (150 mg, 0.26 mmol) in EtOH (5 mL) was added $SnCl_2 \cdot 2H_2O$ (296 mg, 1.31 mmol). The resulting mixture was refluxed for 12 h and then concentrated. The residue was diluted with EtOAc and saturated K_2CO_3, and the aqueous layer was extracted with EtOAc. The combined organic solution was dried over $MgSO_4$, filtered, and concentrated. The residual crude solid (110 mg, brown solid) was used in the next step reaction without further purification. Brown solid; TLC R_f 0.37 (DCM/MeOH = 9/1).

***N*^{1}-(5-((2,6-Dichloro-3,5-dimethoxybenzyl)oxy)-3,4,6-trimethylpyridin-2-yl)-5-(4-ethylpiperazin-1-yl)benzene-1,2-diamine (23Z)**

To a mixture of **22Z** (50 mg, 0.08 mmol) in EtOH (5 mL) was added $SnCl_2 \cdot 2H_2O$ (93 mg, 0.41 mmol). The resulting mixture was refluxed for 24 h and then concentrated. The residue was diluted with EtOAc and saturated K_2CO_3, and the aqueous layer was extracted with EtOAc. The combined organic solution was dried over $MgSO_4$, filtered, and concentrated. The residual crude solid (41 mg) was used in the next step reaction without further purification. Brown solid; TLC R_f 0.17 (DCM/MeOH = 9/1).

N^1-(5-((2,6-difluoro-3,5-dimethoxybenzyl)oxy)-4,6-dimethylpyrimidin-2-yl)-5-(4-ethylpiperazin-1-yl)benzene-1,2-diamine (23AA)

To a mixture of **22AA** (100 mg, 0.18 mmol) in DCM (5 mL) were added zinc dust (117 mg, 1.79 mmol) and acetic acid (205 μL, 3.58 mmol). The resulting mixture was stirred at room temperature for 1 h, and then diluted with DCM and saturated K_2CO_3. The aqueous layer was extracted with DCM, and the combined organic solution was dried over $MgSO_4$, filtered, and concentrated. The residue was purified by flash silica gel column chromatography with 2% to 4% MeOH/DCM to obtain **23AA** (52 mg, 55%). Grey solid; TLC R_f 0.26 (DCM/MeOH = 9/1); m.p. 198 °C; MS (EI) *m/z* 528 $[M]^+$; ^{1}H NMR ($CDCl_3$) δ 7.41 (d, *J* = 2.6 Hz, 1H), 6.78 (d, *J* = 4.8 Hz, 1H), 6.73 (d, *J* = 2.9 Hz, 1H), 6.67 (d, *J* = 8.2 Hz, 1H), 6.62 (dd, *J* = 8.5, 2.7 Hz, 1H), 4.87 (t, *J* = 1.8 Hz, 2H), 3.89 (s, 6H), 3.32–3.09 (m, 4H), 2.64 (dd, *J* = 9.5, 4.7 Hz, 4H), 2.51 (q, *J* = 7.2 Hz, 2H), 2.40 (d, *J* = 7.9 Hz, 6H), 1.15 (t, *J* = 7.2 Hz, 3H); ^{13}C NMR ($CDCl_3$) δ 161.5 (2C), 156.3 (2C), 147.2 (d, *J* = 5.9 Hz), 145.7, 143.8 (d, *J* = 4.8 Hz), 143.6 (d, *J* = 4.8 Hz), 143.3 (d, *J* = 5.8 Hz), 143.2, 132.3, 129.1, 119.2, 113.8, 112.2, 102.2, 62.5, 57.5 (2C), 53.1 (2C), 52.5, 50.5 (2C), 19.0 (2C), 12.0; HRMS (EI) *m/z* calculated for $C_{27}H_{34}F_2N_6O_3$ $[M]^+$ 528.2660, found 528.2660.

N^1-(5-((2,6-Dichloro-3,5-dimethoxybenzyl)oxy)-4,6-dimethylpyrimidin-2-yl)-5-(4-ethylpiperazin-1-yl)benzene-1,2-diamine (23BB)

To a mixture of **22BB** (120 mg, 0.20 mmol) in EtOH (5 mL) was added $SnCl_2{\cdot}2H_2O$ (229 mg, 0.01 mmol). The resulting mixture was refluxed for 12 h and then concentrated. The residue was diluted with DCM and saturated K_2CO_3, and the aqueous layer was extracted with EtOAc. The combined organic solution was dried over $MgSO_4$, filtered, and concentrated. The residue was purified by silica gel column chromatography with 3% to 7% MeOH/DCM to obtain **23BB** (45 mg, 39%). Grey solid; TLC R_f 0.32 (DCM/MeOH = 9/1); m.p. 115 °C; MS (EI) *m/z* 560 $[M]^+$; ^{1}H NMR ($CDCl_3$) δ 7.42 (d, *J* = 2.6 Hz, 1H), 6.77 (d, *J* = 5.8 Hz, 1H), 6.73 (s, 1H), 6.65–6.56 (m, 2H), 5.17 (s, 2H), 3.93 (s, 6H), 3.20–3.08 (m, 4H), 2.63 (dd, *J* = 10.7, 6.0 Hz, 4H), 2.56–2.42 (m, 2H), 2.36 (s, 6H), 1.14 (t, *J* = 7.2 Hz, 3H); ^{13}C NMR ($CDCl_3$) δ 161.5 (2C), 156.1, 154.8 (2C), 145.7, 143.6, 133.7, 132.2, 129.2, 119.1, 116.8 (2C), 113.7, 112.2, 98.1, 69.9, 56.8 (2C), 53.1 (2C), 52.5, 50.6 (2C), 19.6 (2C), 12.0; HRMS (EI) *m/z* calculated for $C_{27}H_{34}Cl_2N_6O_3$ $[M]^+$ 560.2069, found 560.2065.

(3,5-Dimethoxyphenyl)methanol (24) [CAS RN: 705-76-0]

To a mixture of methyl 3,5-dimethoxybenzoate (**23**) (5.0 g, 25.48 mmol) in THF (10 mL) was added $LiAlH_4$ (1 M in THF, 68.8 mL, 68.80 mmol) dropwise over 30 min at −20 °C. The resulting mixture was stirred at room temperature for 12 h and then cooled to 0 °C. After dropwise addition of EtOAc, the mixture was diluted with EtOAc and saturated Rochelle solution. The mixture was then stirred at room temperature until two layers separated clearly. The aqueous layer was extracted with EtOAc and the combined organic solution was dried over $MgSO_4$, filtered, and concentrated to obtain **24** (4.14 g, 97%) as a white solid.

1-(Bromomethyl)-3,5-dimethoxybenzene (25) [CAS RN: 877-88-3]

To a solution of (3,5-dimethoxyphenyl)methanol (**24**) (2.6 g, 15.46 mmol) in DCM (10 mL) was added PBr_3 (1 M in DCM, 16.3 mL, 16.23 mmol) dropwise at 0 °C. The resulting mixture was stirred at room temperature for 15 min and quenched with ice water at 0 °C. The mixture was extracted with EtOAc and the combined organic solution was dried over $MgSO_4$, filtered, and concentrated. The crude product **25** was used in the next step reaction without further purification.

4,5-Bis(chloromethyl)-2-methylpyridin-3-ol hydrochloride (27) [CAS RN: 39984-50-4]

To a suspension of pyridoxine·HCl (**26**) (10.0 g, 48.63 mmol) in thionyl chloride (15 mL) was added DMF (0.38 mL, 4.86 mmol). The mixture was refluxed for 5 h. Ethyl ether (140 mL) was added and stirred for 1 h in an ice bath. The mixture was filtered, and the filter cake was washed with ethyl ether. The filter cake was dried to obtain **27** (11.3 g, 96% yield) as a brown solid.

2,4,5-Trimethylpyridin-3-ol (28) [CAS RN: 5622-78-6]

To a suspension of 4,5-bis(chloromethyl)-2-methylpyridin-3-ol hydrochloride (**27**) (11.3 g, 46.59 mmol) in acetic acid (50 mL) was added zinc dust (9.2 g, 139.80 mmol) in small portions. The mixture was refluxed for 2 h and then cooled to room temperature. The mixture was filtered and washed with acetic acid. The pH of the filtrate was adjusted to 6 with 10 M NaOH and brine. The aqueous layer was extracted with EtOAc. The combined organic solution was dried over $MgSO_4$, filtered, and concentrated. The residue was purified by flash silica gel column chromatography with 3% to 5% MeOH/$CHCl_3$ to obtain **28** (3.6 g, 56% yield) as an ivory solid.

4,6-Dimethylpyrimidin-5-ol (31) [CAS RN: 70345-38-9]

To a solution of 3-chloro-2,4-pentadione (**29**) (20 mL, 177.17 mmol) in formic acid (30 mL) was added formamide (16.0 ml, 402.84 mmol) and refluxed for 12 h. The mixture was cooled to room temperature and aqueous NH_4OH was added dropwise until the basic pH was maintained. The mixture was refluxed for 5 h and then cooled to room temperature, and concentrated. The residue was washed with acetone to acquire product in acetone. The acetone fraction was then concentrated, the residue was purified by flash silica gel column chromatography with EtOAc to obtain **31** (7.4 g, 34%, for two steps) as a pale yellow solid.

***N*-(1-Methyl-1*H*-pyrazol-3-yl)acetamide hydrochloride (33) [CAS RN: 2020396-21-6]**

To a solution of 1-methyl-1*H*-pyrazol-3-amine (**32**) (4.9 g, 50.45 mmol) in THF (50 mL) was added acetyl chloride (7.2 mL, 101.26 mmol) dropwise at 0 °C. The resulting mixture was stirred at room temperature for 2 h. The precipitate formed was collected to obtain **33** (8.8 g) as a white solid then used in the next step reaction without further purification.

***N*-(2-Chloro-4-fluorophenyl)acetamide (36) [CAS RN: 399-35-9]**

To a solution of 2-chloro-4-fluoroaniline (**35**) (5.0 mL, 41.87 mmol) in THF (10 mL) was added acetyl chloride (5.9 mL, 83.75 mmol) at 0 °C. The mixture was stirred at room temperature for 5 h. The precipitate formed was collected to obtain **36** (8.2 g) as a white solid then used in the next step reaction without further purification.

***N*-(3-Methoxyphenyl)acetamide (39) [CAS RN: 588-16-9]**

A solution of *m*-anisidine (**38**) (5.0 g, 40.6 mmol) in THF (20 mL) was added acetyl chloride (5.8 mL, 81.20 mmol) dropwise at 0 °C. The mixture was stirred for 30 min then concentrated. The residue was dissolved in EtOAc and washed with saturated $NaHCO_3$. The organic layer was dried over $MgSO_4$, filtered, and concentrated. The residue was purified by flash silica gel column chromatography with 10% to 50% EtOAc/Hexanes to obtain **39** (5.9 g, 88%) as a beige solid.

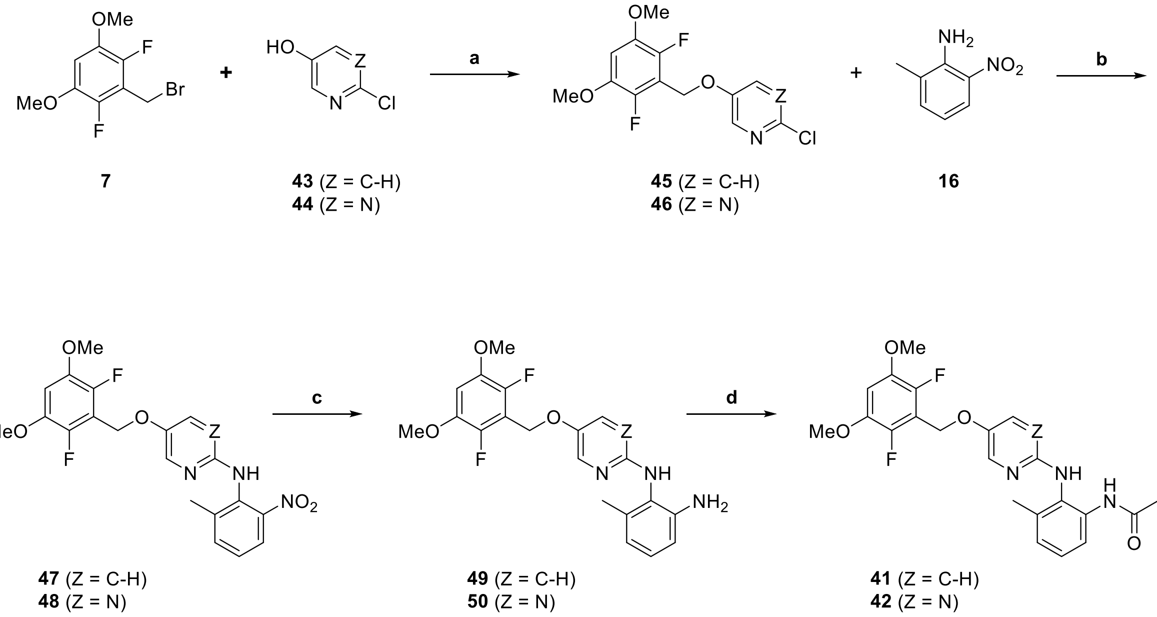

Scheme S1. Synthetic scheme for **41** and **42**. *Reagents and Conditions*: (a) K_2CO_3, DMF, 80 °C; (b) $Pd_2(dba)_3$, Xantphos, Cs_2CO_3, PhMe, reflux; (c) Zn, AcOH, DCM, r.t.; (d) acryloyl chloride, Et_3N, DCM, 0 °C to r.t.

***N*-(2-((5-((2,6-Difluoro-3,5-dimethoxybenzyl)oxy)pyridin-2-yl)amino)-3-methylphenyl)acrylamide (41) [CAS RN: 2020395-19-9]**

To a solution of **49** (70 mg, 0.17 mmol) in DCM (2 mL) were added Et_3N (73 µL, 0.52 mmol) and acryloyl chloride (18 µL, 0.23 mmol) dropwise at 0 °C. The resulting mixture was stirred at room temperature for 30 min and then diluted with DCM and saturated $NaHCO_3$. The aqueous layer was extracted with DCM and the combined organic solution was dried over $MgSO_4$, filtered, and concentrated. The residue was washed with Et_2O and collected to obtain **41** (6 mg, 7%). Yellow solid; TLC R_f 0.19 (EtOAc/Hexanes = 1/2); m.p. 148 °C; MS (EI) *m*/*z* 455 $[M]^+$; ^{1}H NMR ($CDCl_3$) δ 8.36–8.19 (m, 2H), 7.97 (d, *J* = 2.5 Hz, 1H), 7.25–7.10 (m, 2H), 7.03 (d, *J* = 7.6 Hz, 1H), 6.66 (t, *J* = 8.1 Hz, 1H), 6.36–6.25 (m, 1H), 6.22–6.06 (m, 2H), 5.75 (s, 1H), 5.67 (dd, *J* = 10.0, 1.6 Hz, 1H), 5.09 (t, *J* = 1.5 Hz, 2H), 3.88 (s, 6H), 2.18 (s, 3H); ^{13}C NMR ($CDCl_3$) δ 163.6, 152.6, 149.2 (2C), 143.6 (d, *J* = 5.2 Hz), 143.4 (d, *J* = 4.9 Hz), 138.7, 136.3 (2C), 131.5, 127.6, 127.4, 127.2, 126.3, 118.8, 113.7, 107.3, 101.8, 60.1, 57.3 (2C), 18.2; HRMS (EI) *m*/*z* calculated for $C_{24}H_{23}F_2N_3O_4$ $[M]^+$ 455.1657, found 455.1659.

***N*-(2-((5-((2,6-Difluoro-3,5-dimethoxybenzyl)oxy)pyrimidin-2-yl)amino)-3-methylphenyl)acrylamide (42) [CAS RN: 2020394-83-4]**

To a solution of **50** (90 mg, 0.22 mmol) in DCM (1 mL) were added Et_3N (63 µL, 0.45 mmol) and acryloyl chloride (24 µL, 0.29 mmol) dropwise at 0 °C. The resulting mixture was stirred at room temperature for 30 min and diluted with DCM and saturated $NaHCO_3$. The aqueous layer was extracted with DCM and the combined organic solution was dried over $MgSO_4$, filtered, and concentrated. The residue was purified by flash silica gel column chromatography with 30% to 40% EtOAc/Hexanes to obtain **42** (49 mg, 47%). Pale yellow solid; TLC R_f 0.23 (EtOAc/Hexanes = 1/1); m.p. 130 °C; MS (EI) *m/z* 456 $[M]^+$; ^1H NMR ($CDCl_3$) δ 8.22 (s, 1H), 8.16 (s, 2H), 8.02 (d, *J* = 7.0 Hz, 1H), 7.22 (t, *J* = 7.9 Hz, 1H), 7.05 (d, *J* = 7.3 Hz, 1H), 6.67 (t, *J* = 8.2 Hz, 1H), 6.47 (s, 1H), 6.31 (dd, *J* = 16.9, 1.6 Hz, 1H), 6.14 (dd, *J* = 16.9, 10.0 Hz, 1H), 5.67 (dd, *J* = 10.0, 1.6 Hz, 1H), 5.11 (t, *J* = 1.5 Hz, 2H), 3.88 (s, 6H), 2.22 (s, 3H); ^{13}C NMR ($CDCl_3$) δ 168.2, 164.0, 157.3, 147.4 (2C), 146.8 (d, *J* = 4.2 Hz), 143.7 (d, *J* = 5.0 Hz), 143.6 (d, *J* = 5.0 Hz), 142.9 (d, *J* = 5.7 Hz), 135.9 (2C), 135.0, 131.7, 128.8, 127.2 (d, *J* = 6.4 Hz), 126.9, 120.8, 113.2 (t, *J* = 17.4 Hz), 102.0, 60.8, 57.4 (2C), 18.6; HRMS (EI) *m/z* calculated for $C_{23}H_{22}F_2N_4O_4$ $[M]^+$ 456.1609, found 456.1617.

2-Chloro-5-((2,6-difluoro-3,5-dimethoxybenzyl)oxy)pyridine (45) [CAS RN: 2020396-06-7]

To a solution of 6-chloropyridin-3-ol (**43**) (150 mg, 1.16 mmol) in DMF (2 mL) were added K_2CO_3 (799 mg, 5.79 mmol) and 3-(bromomethyl)-2,4-difluoro-1,5-dimethoxybenzene (**7**) (402 mg, 1.51 mmol). The resulting mixture was stirred at 80 °C for 6 h and then cooled to room temperature. After addition of ice water, the mixture was stirred at 0 °C for 15 min and extracted with EtOAc. The combined organic solution was dried over $MgSO_4$, filtered, and concentrated. The residue was purified by flash silica gel column chromatography with 5% to 10% EtOAc/Hexanes to obtain **45** (363 mg, 99%). Pale yellow solid; TLC R_f 0.5 (EtOAc/Hexanes = 1/2).

2-Chloro-5-((2,6-difluoro-3,5-dimethoxybenzyl)oxy)pyrimidine (46) [CAS RN: 1453211-61-4]

To a solution of 2-chloropyrimidin-5-ol (**44**) (250 mg, 1.92 mmol) in DMF (2 mL) were added K_2CO_3 (1.3 g, 9.58 mmol) and 3-(bromomethyl)-2,4-difluoro-1,5-dimethoxybenzene (**7**) (665 mg, 2.49 mmol). The resulting mixture was stirred at 80 °C for 6 h and then cooled to room temperature. After addition of ice water, the mixture was stirred at 0 °C for 15 min and

extracted with EtOAc. The combined organic solution was dried over $MgSO_4$, filtered, and concentrated. The residue was purified by flash silica gel column chromatography with 5% to 10% EtOAc/Hexanes to obtain **46** (574 mg, 95%). Pale yellow solid; TLC R_f 0.38 (EtOAc/Hexanes = 1/2).

5-((2,6-Difluoro-3,5-dimethoxybenzyl)oxy)-*N*-(2-methyl-6-nitrophenyl)pyridin-2-amine (47)

To a mixture of **45** (200 mg, 0.63 mmol) and 2-methyl-6-nitroaniline (**16**) (115 mg, 0.76 mmol) in toluene (3 mL) were added Cs_2CO_3 (618 mg, 1.90 mmol), Xantphos (110 mg, 0.19 mmol), and $Pd_2(dba)_3$ (65.4 mg, 0.06 mmol). The resulting mixture was refluxed for 5 h and then diluted with EtOAc. The organic layer was washed with brine, dried over $MgSO_4$, filtered, and concentrated. The residue was purified by silica gel column chromatography with 5% to 10% EtOAc/Hexanes to obtain **47** (140 mg, 51%). Red solid; TLC R_f 0.60 (EtOAc/Hexanes = 1/1); m.p. 75 °C; MS (EI) *m/z* 431 $[M]^+$; ^1H NMR ($CDCl_3$) δ 8.32 (s, 1H), 7.94 (t, *J* = 6.1 Hz, 2H), 7.47 (d, *J* = 7.4 Hz, 1H), 7.29 (d, *J* = 3.0 Hz, 1H), 7.14 (t, *J* = 7.9 Hz, 1H), 6.67 (t, *J* = 8.1 Hz, 1H), 6.58 (d, *J* = 8.9 Hz, 1H), 5.11 (t, *J* = 1.5 Hz, 2H), 3.88 (s, 6H), 2.17 (s, 3H); ^{13}C NMR ($CDCl_3$) δ 149.9 (2C), 147.1 (d, *J* = 5.9 Hz), 143.8 (d, *J* = 5.0 Hz), 143.6 (d, *J* = 4.9 Hz), 143.2 (d, *J* = 5.9 Hz), 136.8 (2C), 136.5, 135.1, 134.8, 127.2, 123.7, 123.6, 113.7 (t, *J* = 17.4 Hz), 111.9, 102.1, 60.0, 57.5 (2C), 19.8; HRMS (EI) *m/z* calculated for $C_{21}H_{19}F_2N_3O_5$ $[M]^+$ 431.1293, found 431.1290.

5-((2,6-Difluoro-3,5-dimethoxybenzyl)oxy)-*N*-(2-methyl-6-nitrophenyl)pyrimidin-2-amine (48) [CAS RN: 2097619-50-4]

To a mixture of **46** (200 mg, 0.79 mmol) and 2-methyl-6-nitroaniline (**16**) (144 mg, 0.95 mmol) in toluene (5 mL) were added Cs_2CO_3 (771 mg, 2.37 mmol), Xantphos (137 mg, 0.24 mmol), and $Pd_2(dba)_3$ (82 mg, 0.08 mmol). The resulting mixture was refluxed for 12 h and then diluted with DCM. The organic layer was washed with brine, dried over $MgSO_4$, filtered, and concentrated. The residue was purified by silica gel column chromatography with 10% to 20% EtOAc/Hexanes to obtain **48** (158 mg, 46%). Yellow solid; TLC R_f 0.23 (EtOAc/Hexanes = 1/2); m.p. 117 °C; MS (EI) *m/z* 432 $[M]^+$; ^1H NMR ($CDCl_3$) δ 8.15 (s, 2H), 7.99 (s, 1H), 7.84 (dd, *J* = 8.2, 1.0 Hz, 1H), 7.49 (d, *J* = 7.1 Hz, 1H), 7.19 (t, *J* = 7.9 Hz, 1H), 6.66 (t, *J* = 8.2 Hz, 1H), 5.10 (t, *J* = 1.4 Hz, 2H), 3.86 (s, 6H), 2.28 (s, 3H); ^{13}C NMR ($CDCl_3$) δ 155.5, 147.5,

146.9 (2C), 144.8, 143.8 (d, J = 5.0 Hz), 143.7 (d, J = 4.9 Hz), 143.0 (d, J = 5.7 Hz), 137.5, 136.1 (2C), 132.6, 124.9, 123.2, 113.3 (t, J = 17.4 Hz), 102.2, 60.7, 57.5 (2C), 19.4; HRMS (EI) m/z calculated for $C_{20}H_{18}F_2N_4O_5$ $[M]^+$ 432.1245, found 432.1245.

N^1-(5-((2,6-Difluoro-3,5-dimethoxybenzyl)oxy)pyridin-2-yl)-6-methylbenzene-1,2-diamine (49)

To a mixture of **47** (130 mg, 0.30 mmol) in DCM (2 mL) were added zinc dust (99 mg, 1.51 mmol) and acetic acid (173 µL, 3.01 mmol). The mixture was stirred at room temperature for 1 h, and then diluted with DCM and saturated K_2CO_3. The aqueous layer was extracted with DCM and the combined organic solution was dried over $MgSO_4$, filtered, and concentrated. The residue was purified by silica gel column chromatography with 2% to 4% MeOH/DCM to obtain **49** (90 mg, 75%). Grey solid; TLC R_f 0.52 (DCM/MeOH = 20/1); m.p. 150 °C; MS (EI) m/z 401 $[M]^+$; ^{1}H NMR ($CDCl_3$) δ 7.94 (s, 1H), 7.20–7.10 (m, 1H), 7.02 (t, J = 7.6 Hz, 1H), 6.66 (s, 3H), 6.12 (d, J = 9.0 Hz, 1H), 5.69 (s, 1H), 5.08 (s, 2H), 3.88 (s, 6H), 2.16 (s, 3H); ^{13}C NMR ($CDCl_3$) δ 153.2, 148.6, 147.1 (d, J = 5.9 Hz), 145.0, 143.8 (d, J = 4.9 Hz), 143.6 (d, J = 4.9 Hz), 143.3 (d, J = 5.8 Hz), 137.5, 136.2, 127.8, 127.4, 124.5, 120.4, 114.1 (t, J = 17.5 Hz), 113.6, 106.8, 102.0, 60.4, 57.5 (2C), 18.3; HRMS (EI) m/z calculated for $C_{21}H_{21}F_2N_3O_3$ $[M]^+$ 401.1551, found 401.1547.

N^1-(5-((2,6-Difluoro-3,5-dimethoxybenzyl)oxy)pyrimidin-2-yl)-6-methylbenzene-1,2-diamine (50) [CAS RN: 2097517-12-7]

To a mixture of **48** (150 mg, 0.35 mmol) in DCM (2 mL) were added zinc dust (114 mg, 1.74 mmol) and acetic acid (199 µL, 3.47 mmol). The mixture was stirred at room temperature for 1 h and then diluted with DCM and saturated K_2CO_3. The aqueous layer was extracted with DCM and the combined organic solution was dried over $MgSO_4$, filtered, and concentrated. The residue was purified by silica gel column chromatography with 10% to 25% EtOAc/Hexanes to obtain **50** (109 mg, 78%). Yellow solid; TLC R_f 0.50 (DCM/MeOH = 20/1); m.p. 170 °C; MS (EI) m/z 402 $[M]^+$; ^{1}H NMR ($CDCl_3$) δ 8.15 (s, 2H), 7.02 (t, J = 7.7 Hz, 1H), 6.73–6.60 (m, 3H), 6.23 (s, 1H), 5.09 (s, 2H), 3.88 (s, 6H), 2.17 (s, 3H); ^{13}C NMR ($CDCl_3$) δ 157.8 (2C), 147.9, 147.0 (d, J = 5.7 Hz), 146.6, 144.5, 143.8 (d, J = 4.9 Hz), 143.6 (d, J = 5.0 Hz), 143.1 (d, J = 5.7 Hz), 137.0, 127.9, 123.9, 120.6, 114.1, 113.5 (t, J = 17.4 Hz), 102.1, 61.1, 57.5 (2C), 18.5; HRMS (EI) m/z calculated for $C_{20}H_{20}F_2N_4O_3$ $[M]^+$ 402.4018, found 402.1504.

NMR spectra

6A

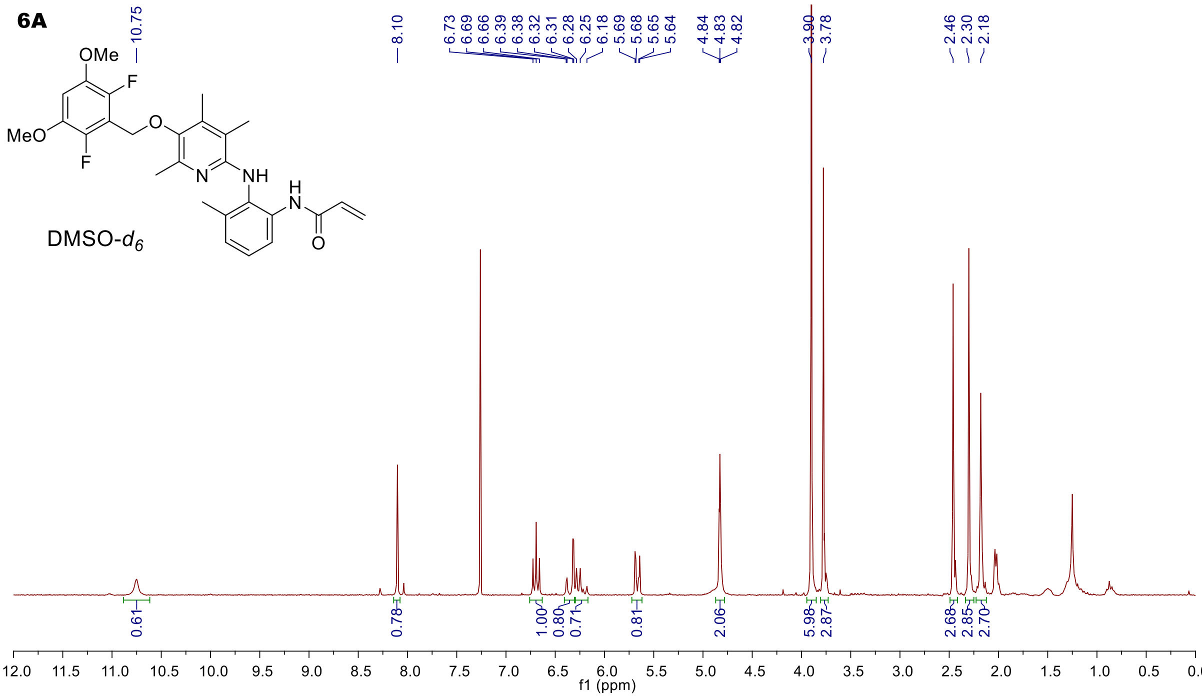

6A

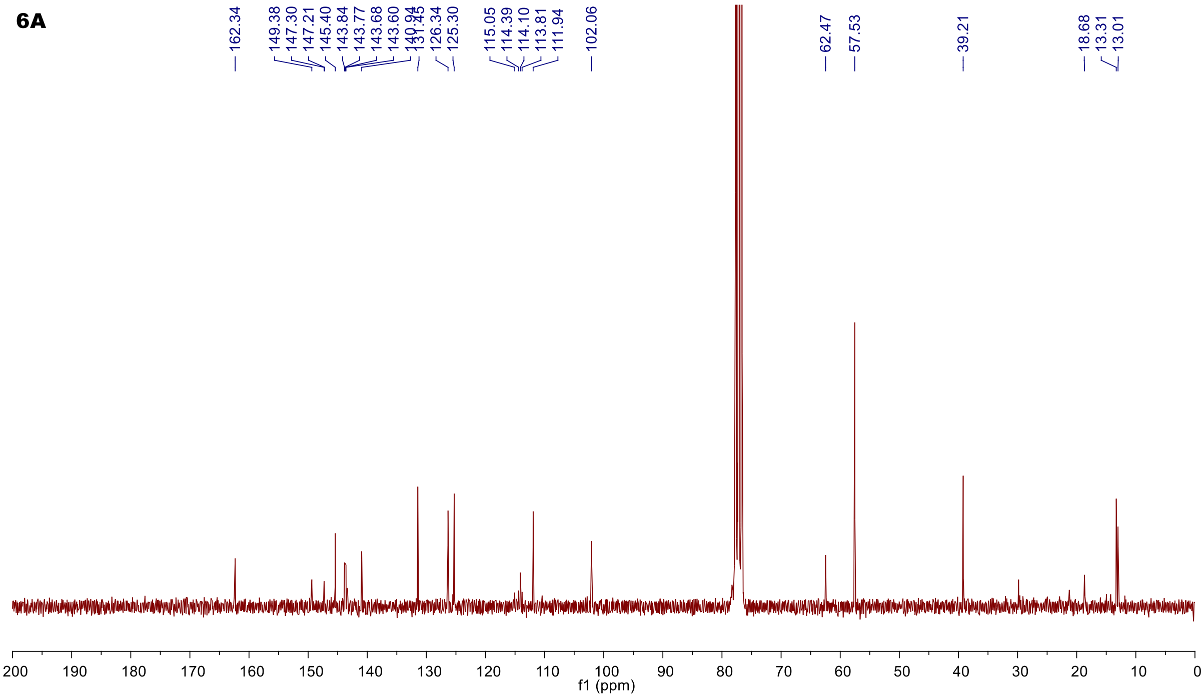

6B

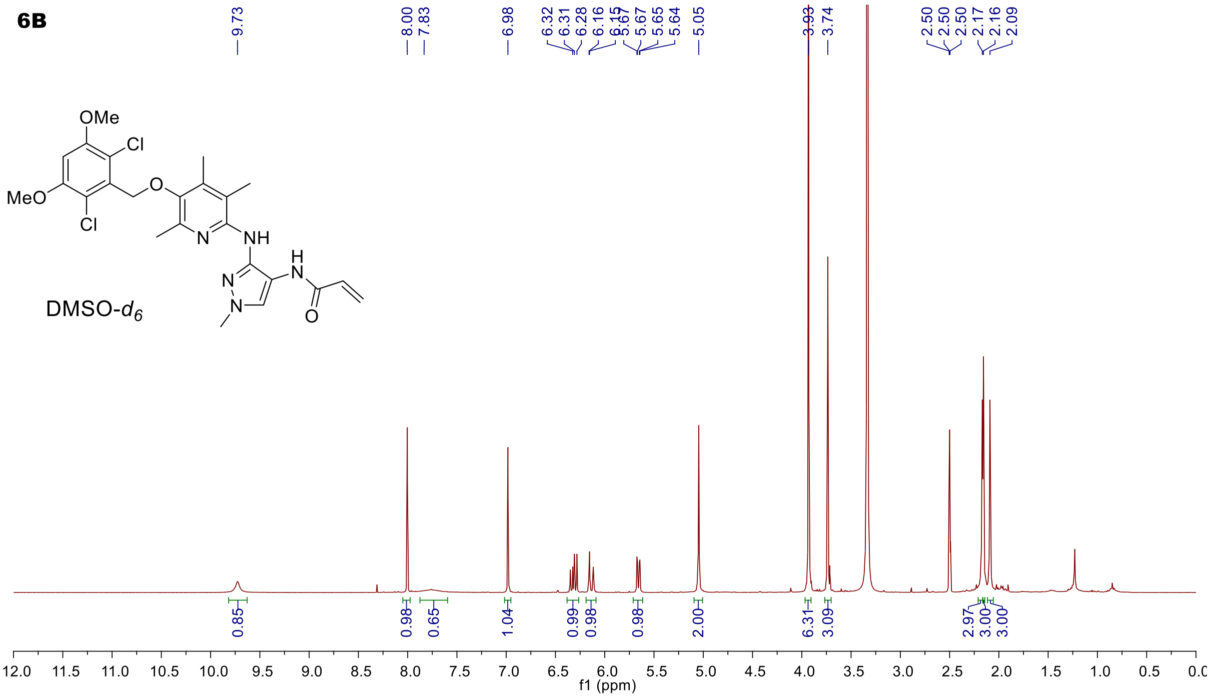
9.73
8.00
7.83
6.98
6.32
6.31
6.28
6.16
6.15
5.67
5.67
5.65
5.64
5.05
3.93
3.74
2.50
2.50
2.50
2.17
2.16
2.09
OMe
Cl
MeO
Cl
O
N
NH
H
N
N
N
O
DMSO-d_6
0.85
0.98
0.65
1.04
0.99
0.98
0.98
2.00
6.31
3.09
2.97
3.00
3.00
12.0 11.5 11.0 10.5 10.0 9.5 9.0 8.5 8.0 7.5 7.0 6.5 6.0 5.5 5.0 4.5 4.0 3.5 3.0 2.5 2.0 1.5 1.0 0.5 0.0
f1 (ppm)

6B

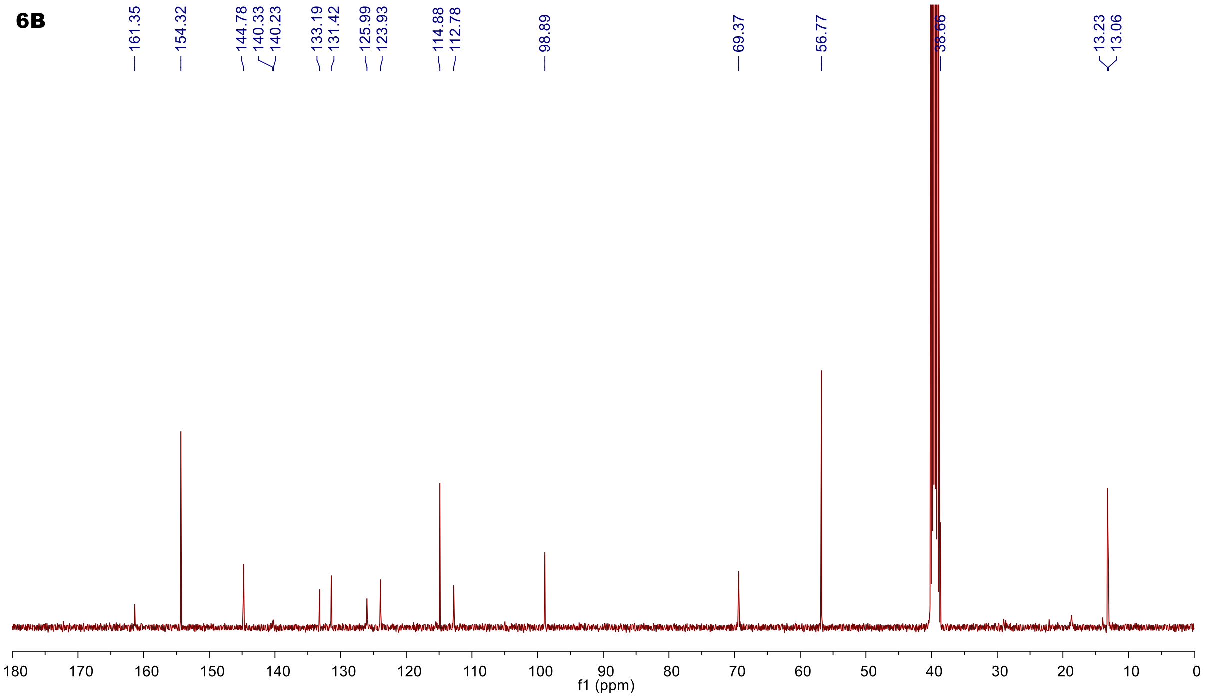
161.35
154.32
144.78
140.33
140.23
133.19
131.42
125.99
123.93
114.88
112.78
98.89
69.37
56.77
38.66
13.23
13.06
180 170 160 150 140 130 120 110 100 90 80 70 60 50 40 30 20 10 0
f1 (ppm)

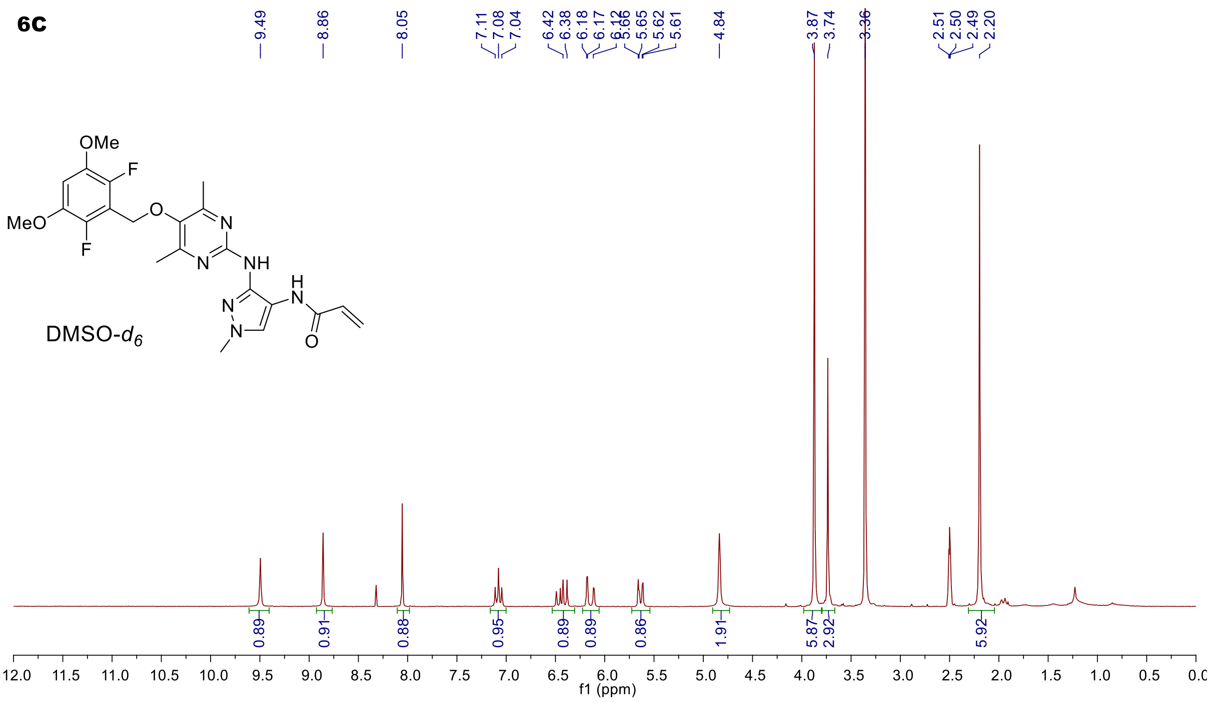
6C
DMSO-d_6
9.49
8.86
8.05
7.11
7.08
7.04
6.42
6.38
6.18
6.17
6.12
5.66
5.65
5.62
5.61
4.84
3.87
3.74
3.36
2.51
2.50
2.49
2.20
0.89
0.91
0.88
0.95
0.89
0.89
0.86
1.91
5.87
2.92
5.92
f1 (ppm)

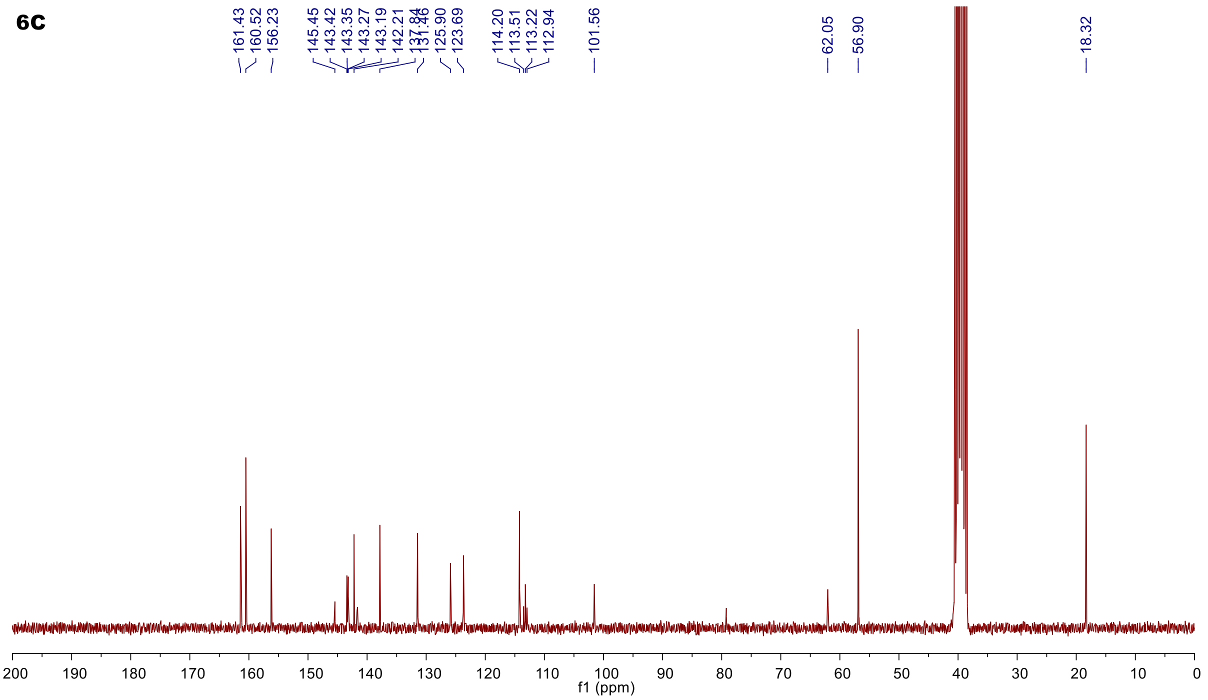
6C
161.43
160.52
156.23
145.45
143.42
143.35
143.27
143.19
142.21
137.84
131.46
125.90
123.69
114.20
113.51
113.22
112.94
101.56
62.05
56.90
18.32
f1 (ppm)

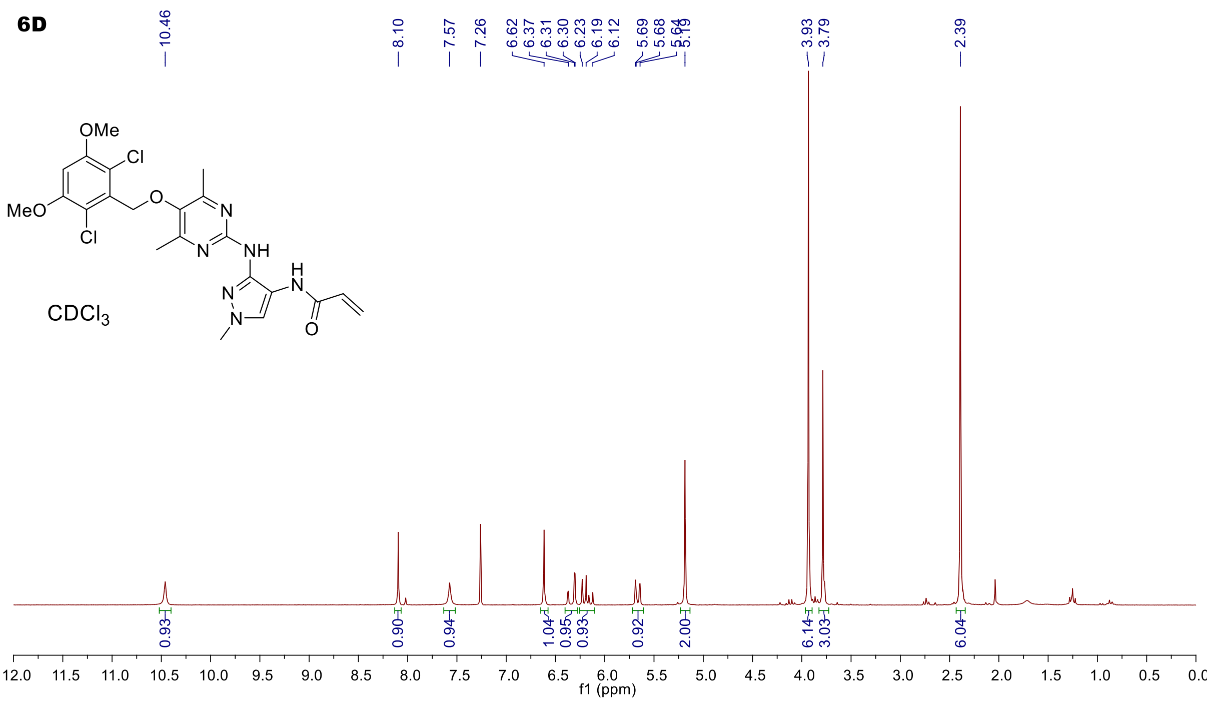
6D
OMe
Cl
MeO
O
Cl
N
N
NH
H
N
N
N
O
CDCl3
10.46
8.10
7.57
7.26
6.62
6.37
6.31
6.30
6.23
6.19
6.12
5.69
5.68
5.64
5.19
3.93
3.79
2.39
0.93
0.90
0.94
1.04
0.95
0.93
0.92
2.00
6.14
3.03
6.04
f1 (ppm)

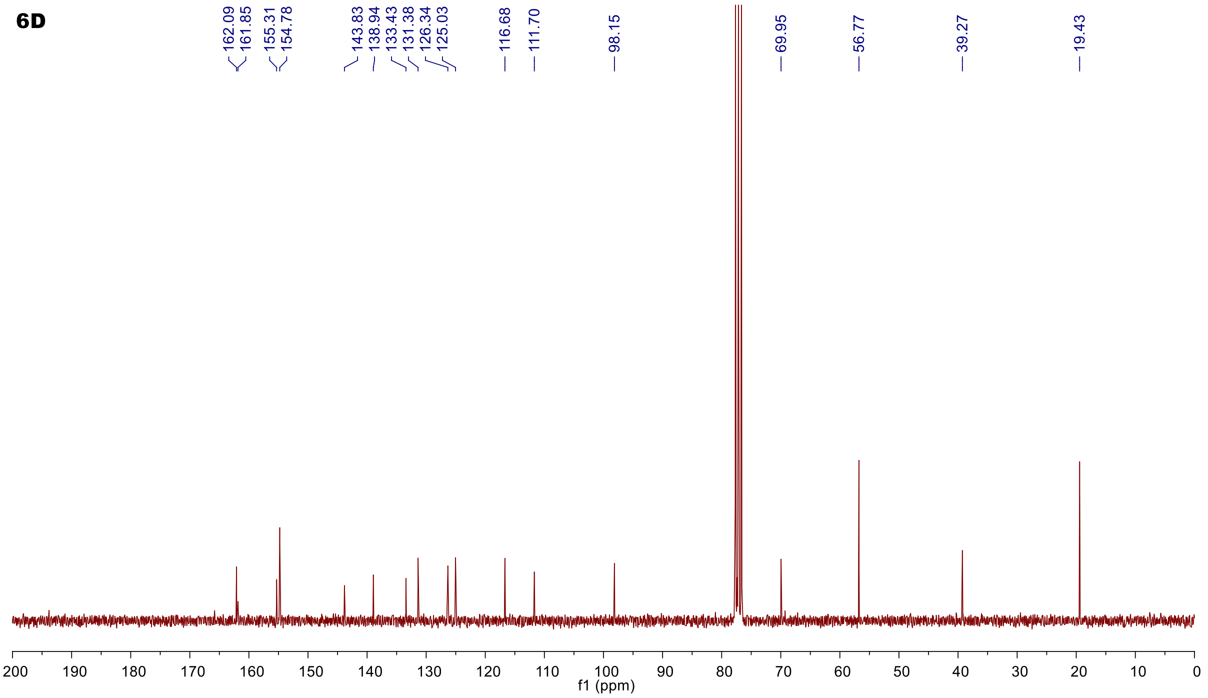
6D
162.09
161.85
155.31
154.78
143.83
138.94
133.43
131.38
126.34
125.03
116.68
111.70
98.15
69.95
56.77
39.27
19.43
f1 (ppm)

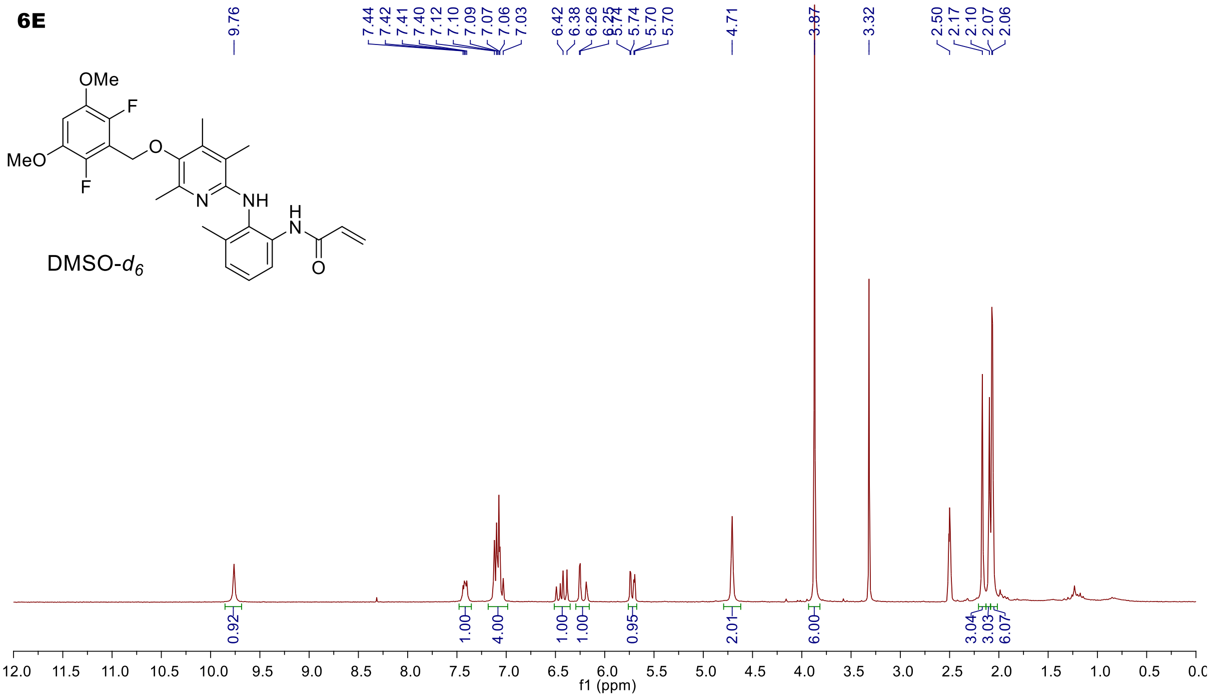
6E
OMe
F
MeO
F
O
N
NH
H
N
O
DMSO-d6
9.76
7.44
7.42
7.41
7.40
7.12
7.10
7.09
7.07
7.06
7.03
6.42
6.38
6.26
5.74
5.70
5.70
4.71
3.87
3.32
2.50
2.17
2.10
2.07
2.06
0.92
1.00
4.00
1.00
1.00
0.95
2.01
6.00
3.04
3.03
6.07
f1 (ppm)

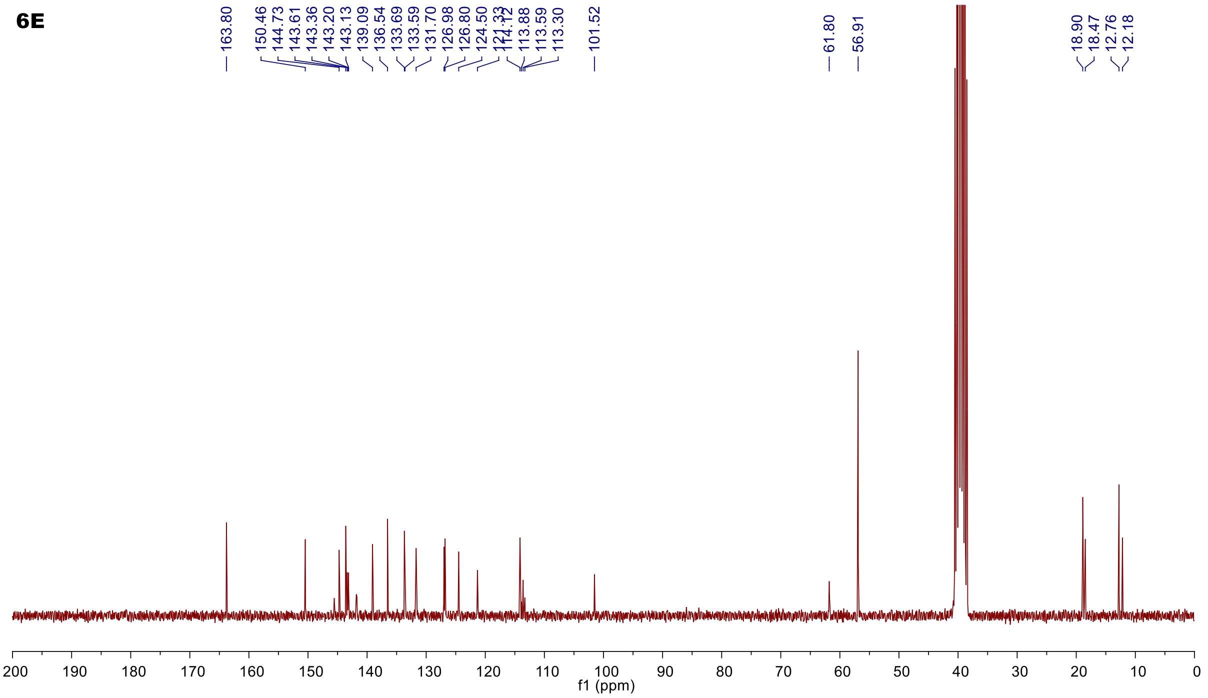
6E
163.80
150.46
144.73
143.61
143.36
143.20
143.13
139.09
136.54
133.69
133.59
131.70
126.98
126.80
124.50
114.12
113.88
113.59
113.30
101.52
61.80
56.91
18.90
18.47
12.76
12.18
f1 (ppm)

6F

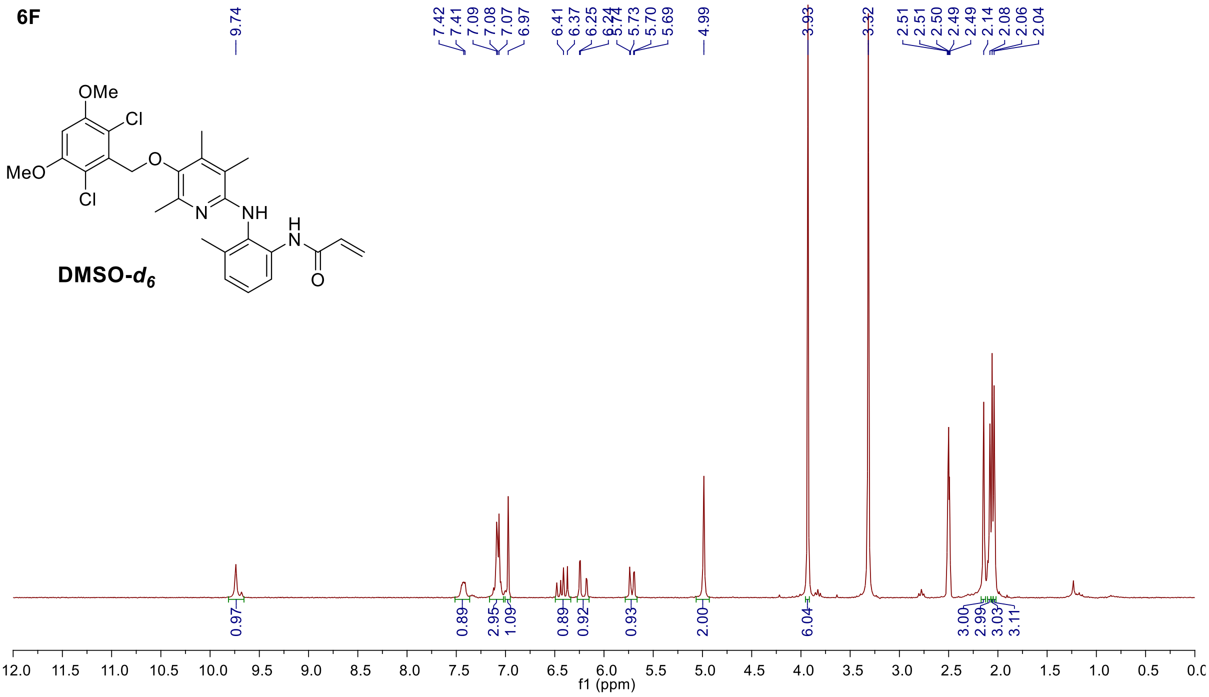
9.74
7.42
7.41
7.09
7.08
7.07
6.97
6.41
6.37
6.25
6.24
5.74
5.73
5.70
5.69
4.99
3.93
3.32
2.51
2.51
2.50
2.49
2.49
2.14
2.08
2.06
2.04
OMe
Cl
MeO
O
Cl
N
NH
H
N
O
DMSO-d_6
0.97
0.89
2.95
1.09
0.89
0.92
0.93
2.00
6.04
3.00
2.99
3.03
3.11
f1 (ppm)

6F

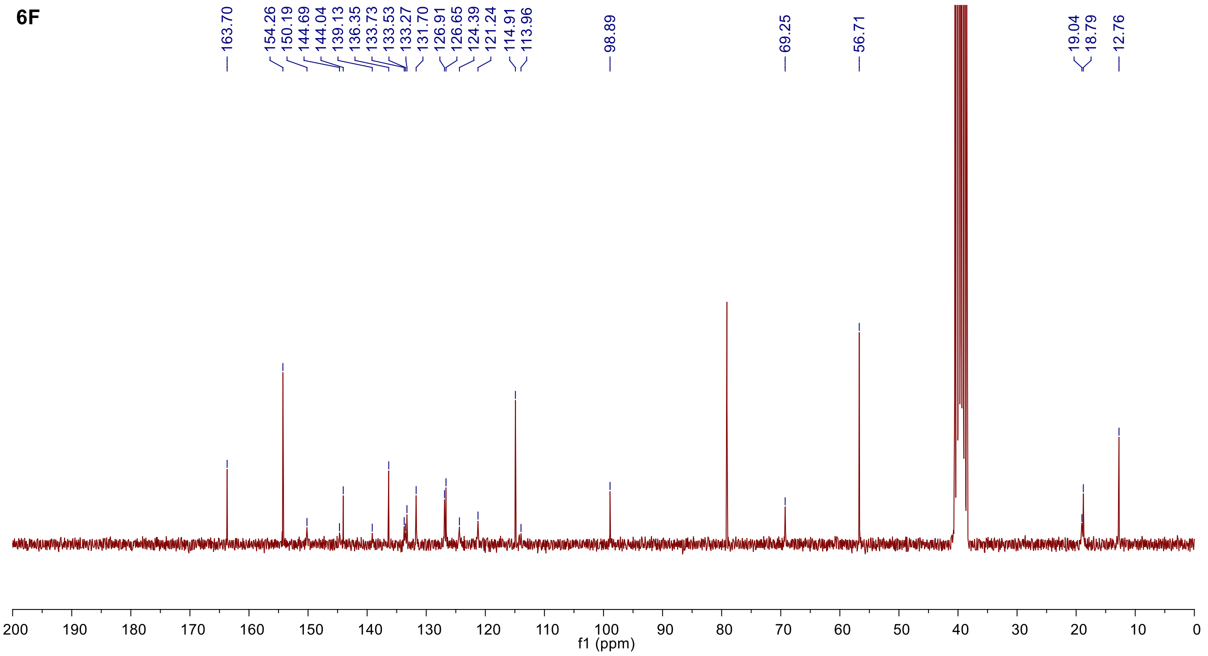
163.70
154.26
150.19
144.69
144.04
139.13
136.35
133.73
133.53
133.27
131.70
126.91
126.65
124.39
121.24
114.91
113.96
98.89
69.25
56.71
19.04
18.79
12.76
f1 (ppm)

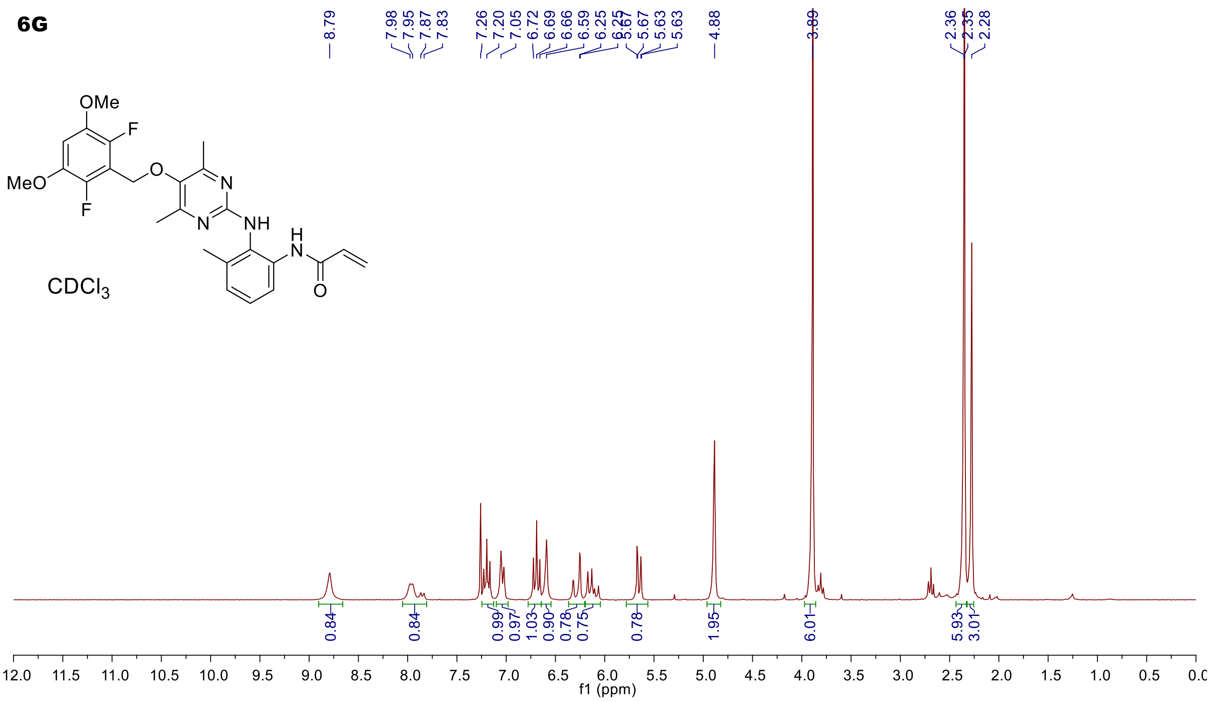
6G
OMe
F
MeO
F
O
N
N
NH
H
N
O
CDCl3
8.79
7.98
7.95
7.87
7.83
7.26
7.20
7.05
6.72
6.69
6.66
6.59
6.25
5.67
5.63
5.63
4.88
3.89
2.36
2.35
2.28
0.84
0.84
0.99
0.97
1.03
0.90
0.78
0.75
0.78
1.95
6.01
5.93
3.01
f1 (ppm)

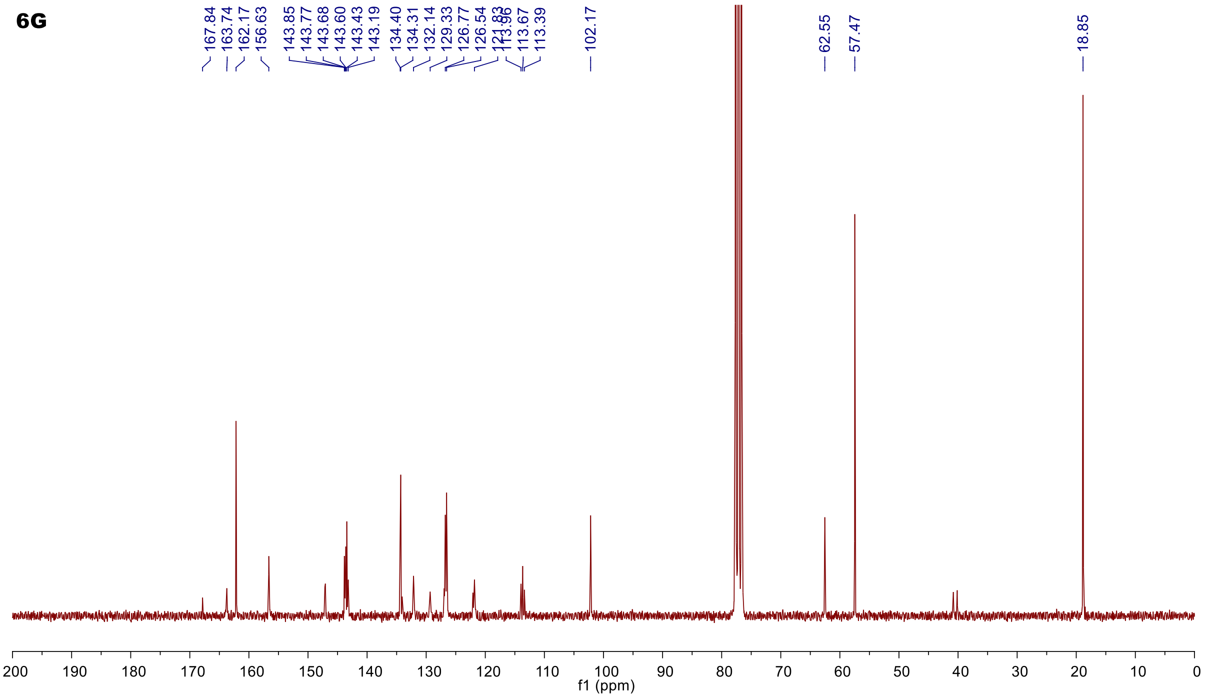
6G
167.84
163.74
162.17
156.63
143.85
143.77
143.68
143.60
143.43
143.19
134.40
134.31
132.14
129.33
126.77
126.54
113.67
113.39
102.17
62.55
57.47
18.85
f1 (ppm)

6H

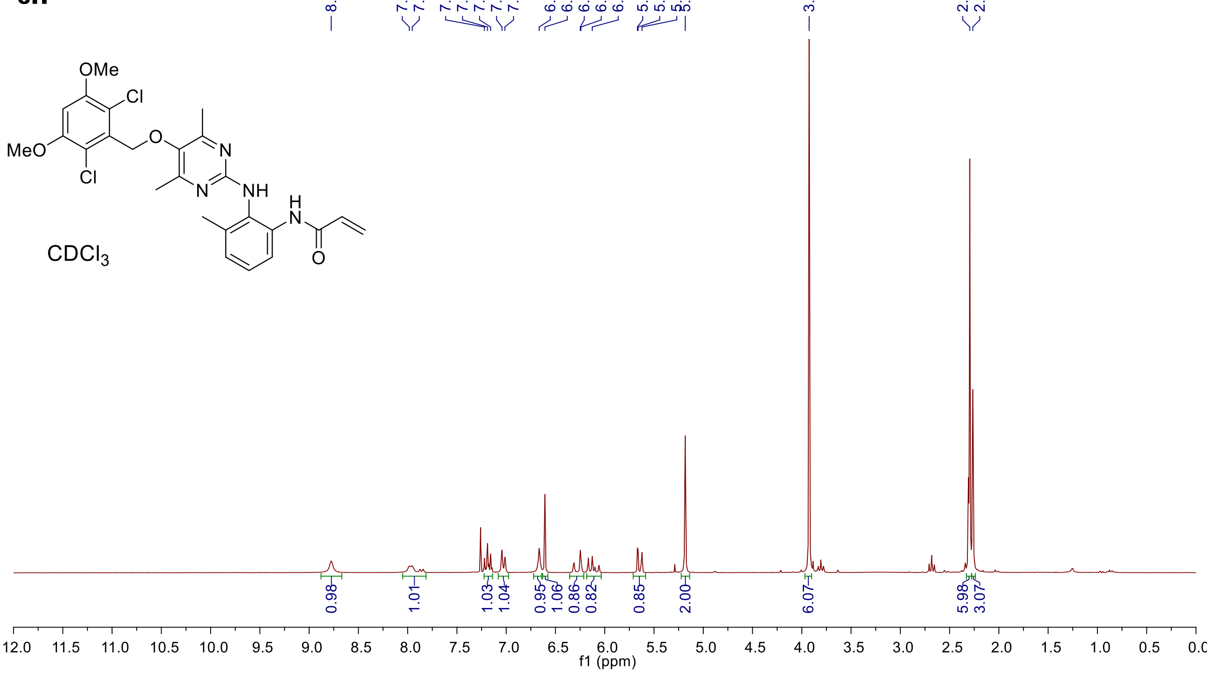
8.78
7.98
7.95
7.22
7.19
7.16
7.04
7.01
6.67
6.61
6.25
6.24
6.13
5.67
5.66
5.62
5.18
3.93
2.30
2.26
OMe
Cl
MeO
O
N
Cl
N
NH
H
N
O
CDCl$_3$
0.98
1.01
1.03
1.04
0.95
1.06
0.86
0.82
0.85
2.00
6.07
5.98
3.07
12.0 11.5 11.0 10.5 10.0 9.5 9.0 8.5 8.0 7.5 7.0 6.5 6.0 5.5 5.0 4.5 4.0 3.5 3.0 2.5 2.0 1.5 1.0 0.5 0.0
f1 (ppm)

6H

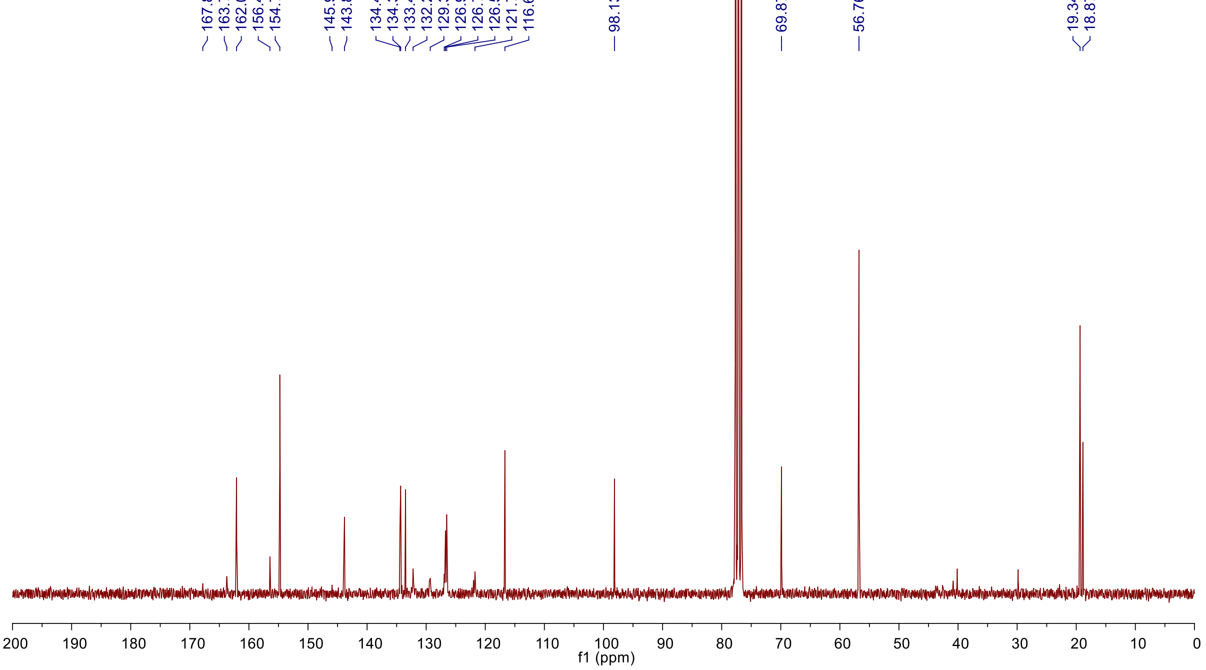
167.802
163.740
162.089
156.444
154.768
145.926
143.823
134.463
134.319
133.493
132.215
129.308
126.941
126.733
126.530
121.744
116.682
98.136
69.874
56.767
19.346
18.873
200 190 180 170 160 150 140 130 120 110 100 90 80 70 60 50 40 30 20 10 0
f1 (ppm)

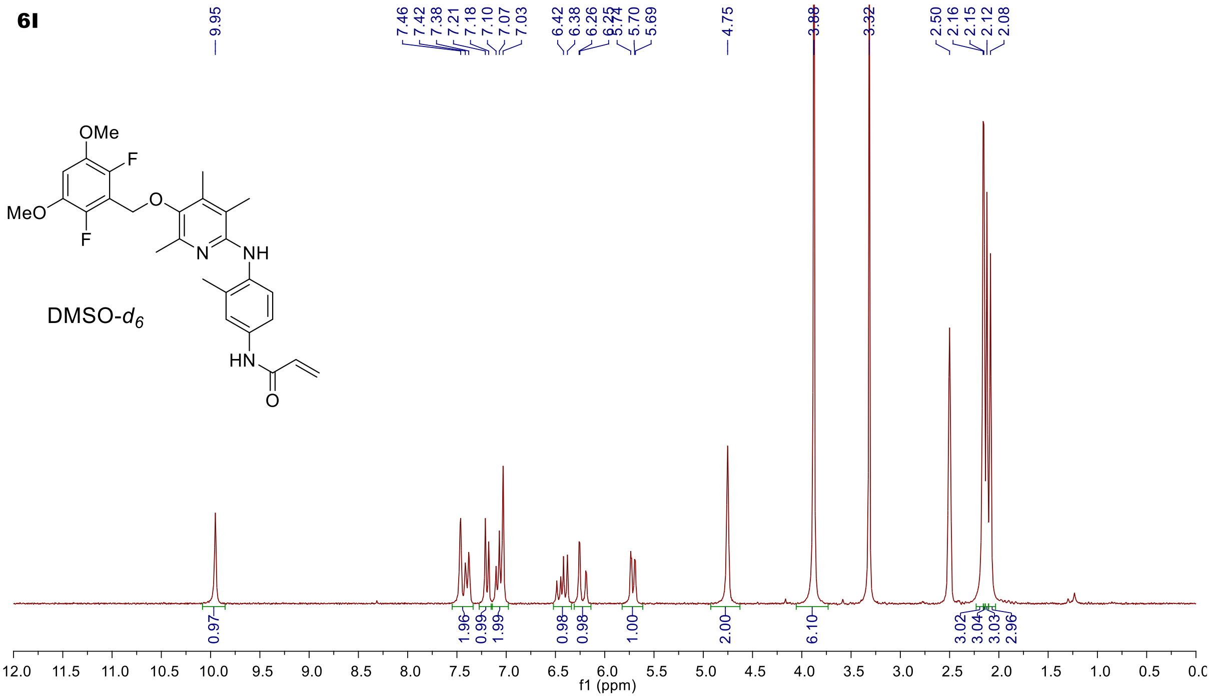
6l
9.95
7.46
7.42
7.38
7.21
7.18
7.10
7.07
7.03
6.42
6.38
6.26
6.25
5.74
5.70
5.69
4.75
3.88
3.32
2.50
2.16
2.15
2.12
2.08
OMe
F
MeO
F
O
N
NH
HN
O
DMSO-d6
0.97
1.96
0.99
1.99
0.98
0.98
1.00
2.00
6.10
3.02
3.04
3.03
2.96
f1 (ppm)

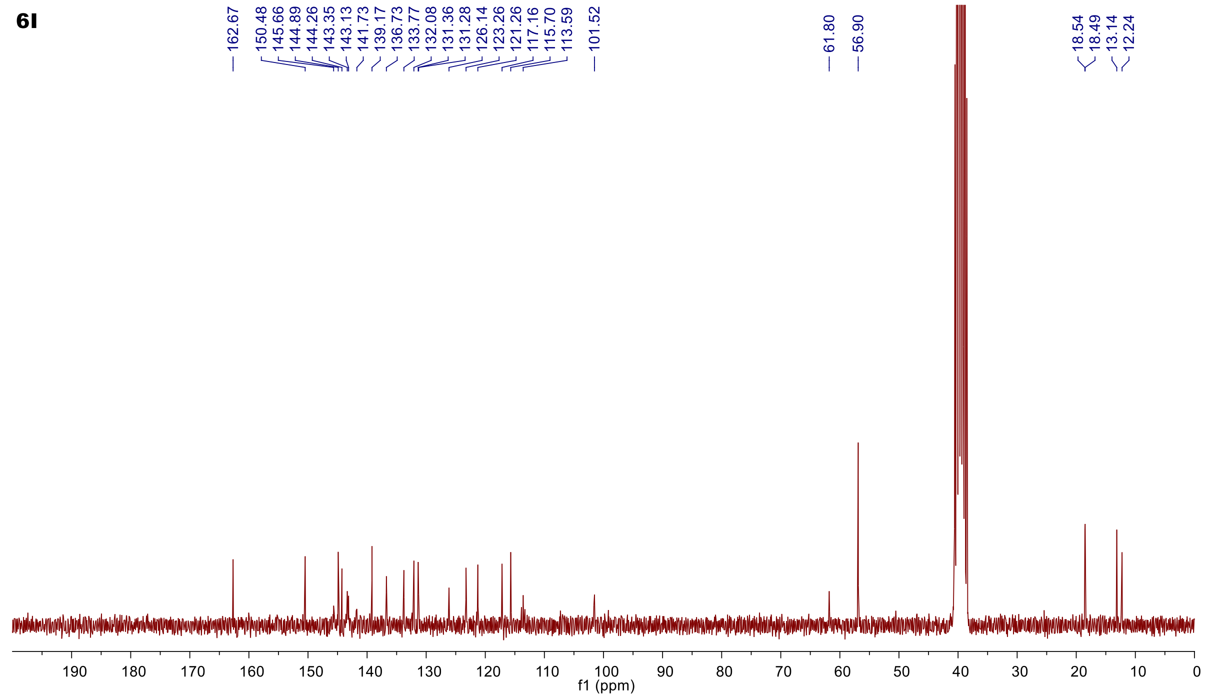
6l
162.67
150.48
145.66
144.89
144.26
143.35
143.13
141.73
139.17
136.73
133.77
132.08
131.36
131.28
126.14
123.26
121.26
117.16
115.70
113.59
101.52
61.80
56.90
18.54
18.49
13.14
12.24
f1 (ppm)

6J

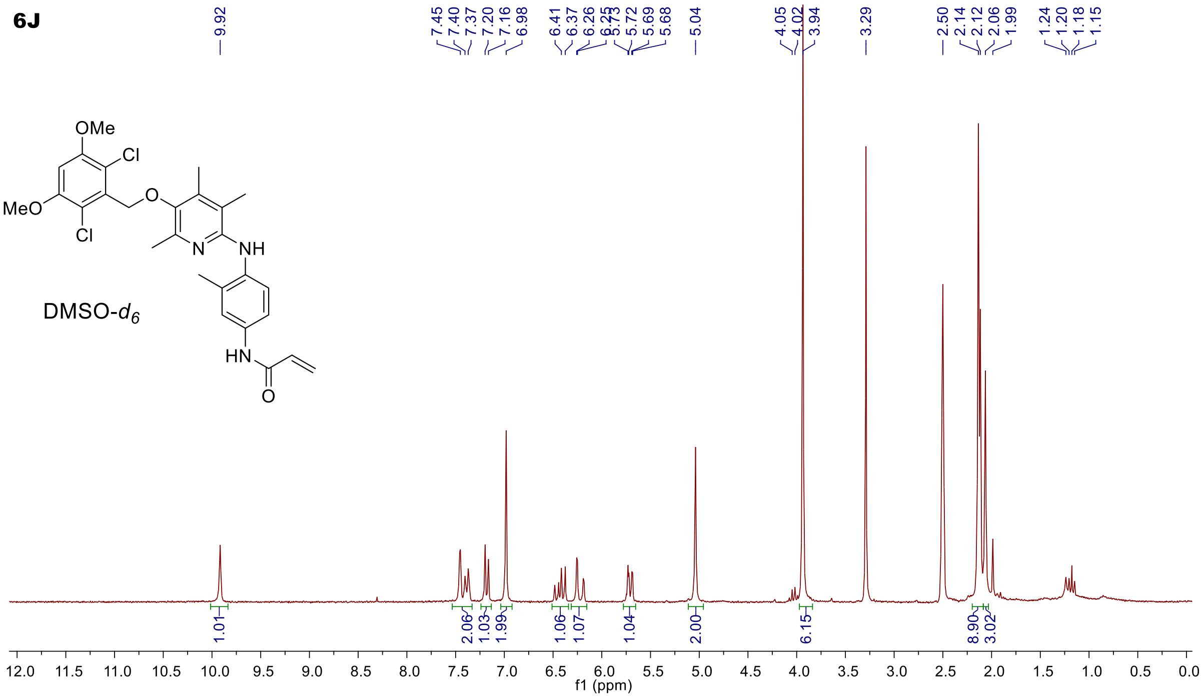
DMSO-d_6
9.92
7.45
7.40
7.37
7.20
7.16
6.98
6.41
6.37
6.26
6.25
5.73
5.72
5.69
5.68
5.04
4.05
4.02
3.94
3.29
2.50
2.14
2.12
2.06
1.99
1.24
1.20
1.18
1.15
1.01
2.06
1.03
1.99
1.06
1.07
1.04
2.00
6.15
8.90
3.02
f1 (ppm)

6J

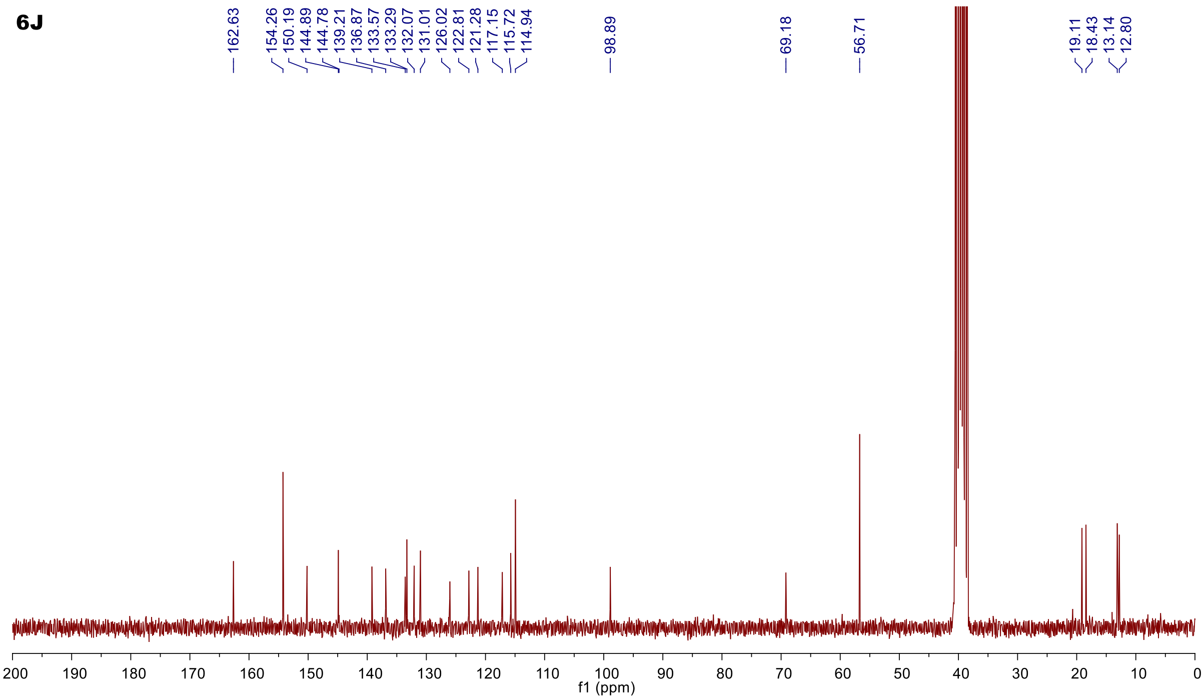
162.63
154.26
150.19
144.89
144.78
139.21
136.87
133.57
133.29
132.07
131.01
126.02
122.81
121.28
117.15
115.72
114.94
98.89
69.18
56.71
19.11
18.43
13.14
12.80
f1 (ppm)

6K

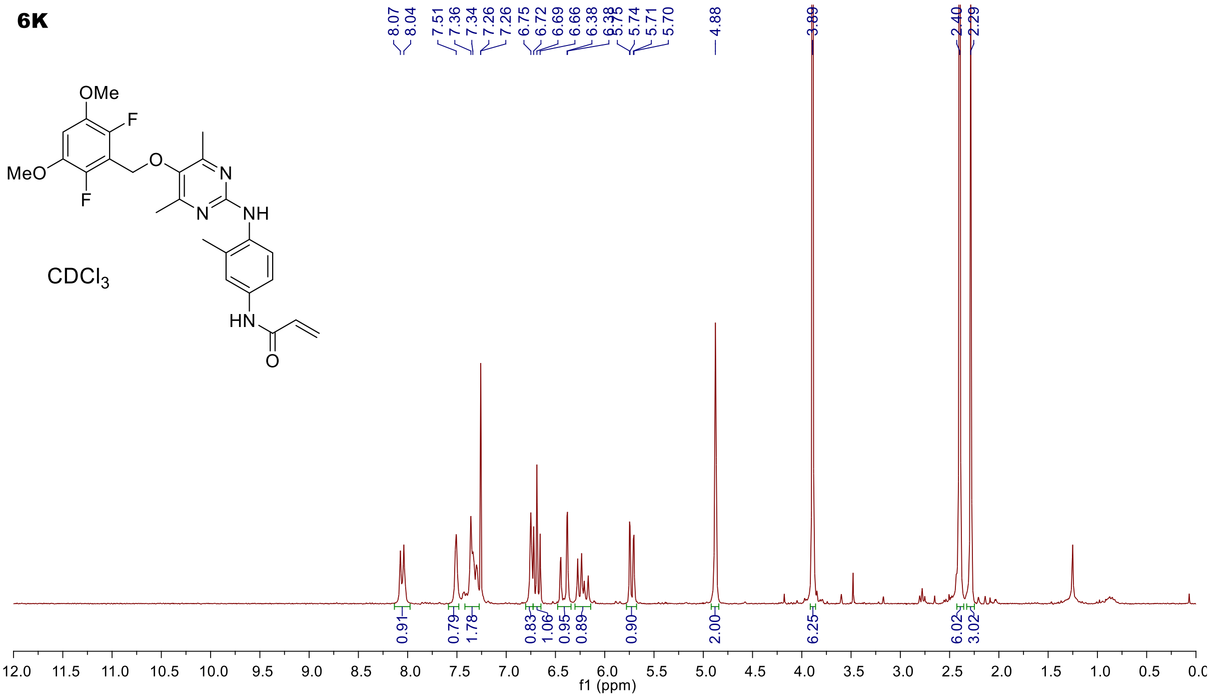
OMe
F
MeO
F
O
N
N
NH
HN
O
CDCl3
8.07
8.04
7.51
7.36
7.34
7.26
7.26
6.75
6.72
6.69
6.66
6.38
6.38
5.75
5.74
5.71
5.70
4.88
3.89
2.40
2.29
0.91
0.79
1.78
0.83
1.06
0.95
0.89
0.90
2.00
6.25
6.02
3.02
f1 (ppm)

6K

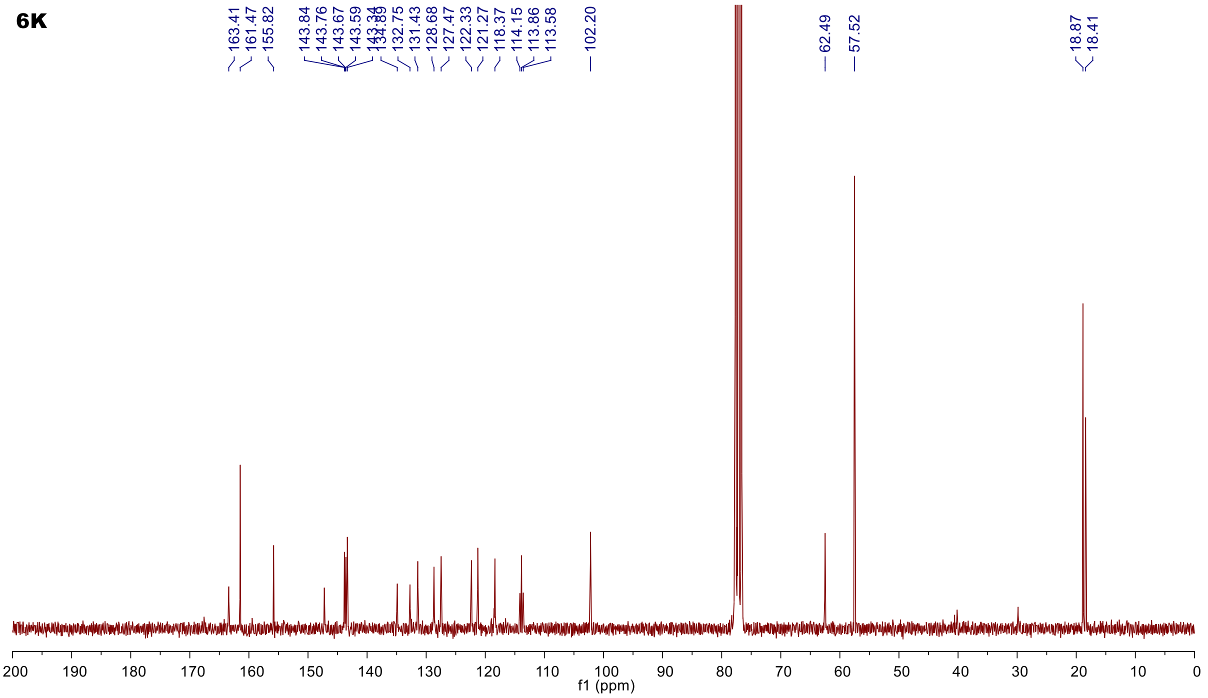
163.41
161.47
155.82
143.84
143.76
143.67
143.59
132.75
131.43
128.68
127.47
122.33
121.27
118.37
114.15
113.86
113.58
102.20
62.49
57.52
18.87
18.41
f1 (ppm)

6L

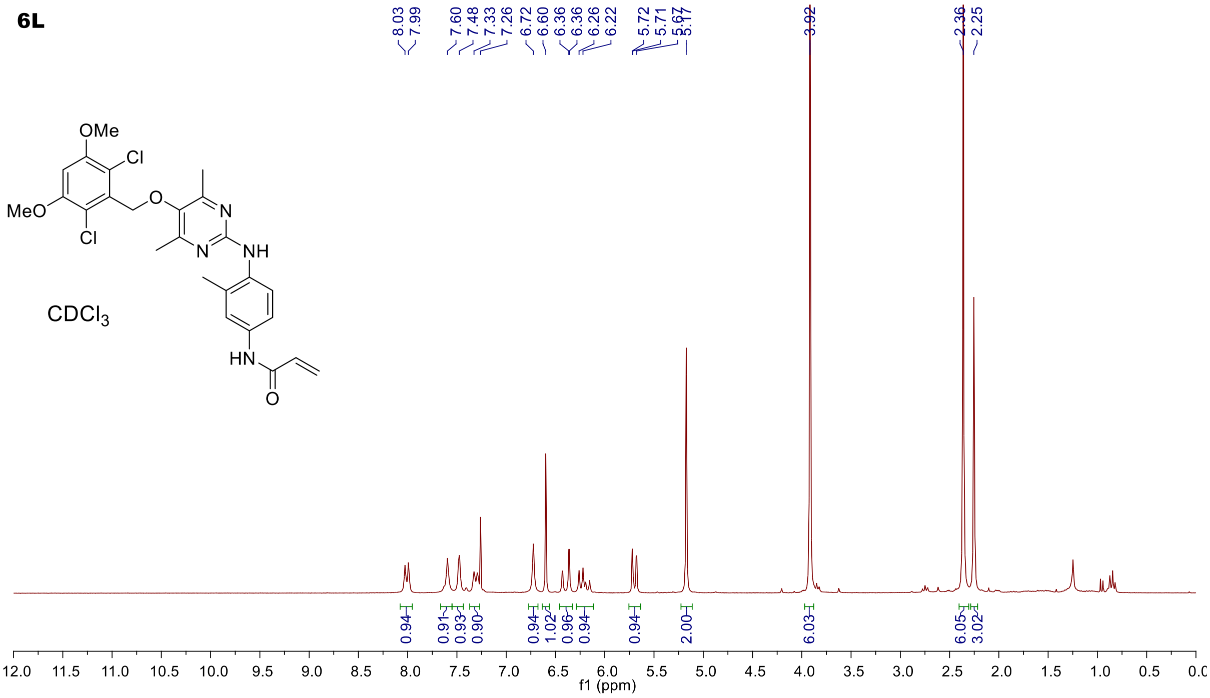

8.03
7.99
7.60
7.48
7.33
7.26
6.72
6.60
6.36
6.36
6.26
6.22
5.72
5.71
5.67
5.17
3.92
2.36
2.25
OMe
Cl
MeO
O
N
N
NH
Cl
CDCl3
HN
O
0.94
0.91
0.93
0.90
0.94
1.02
0.96
0.94
0.94
2.00
6.03
6.05
3.02
f1 (ppm)

6L

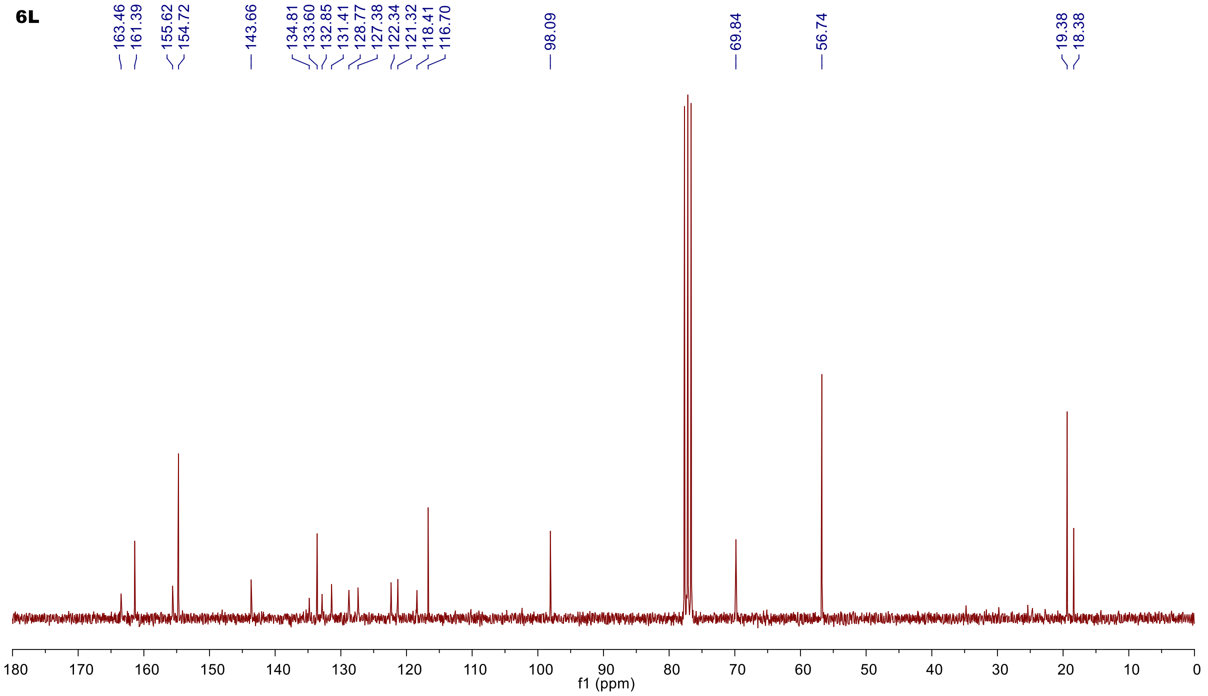

163.46
161.39
155.62
154.72
143.66
134.81
133.60
132.85
131.41
128.77
127.38
122.34
121.32
118.41
116.70
98.09
69.84
56.74
19.38
18.38
f1 (ppm)

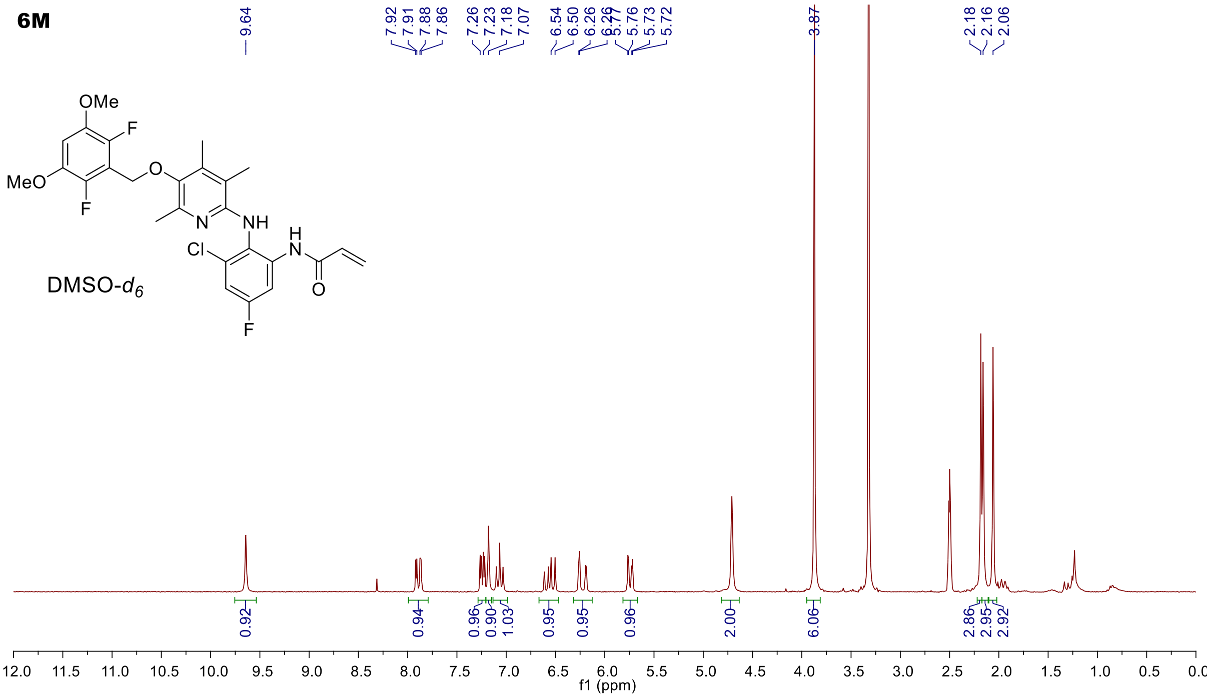
6M
DMSO-d6
OMe
F
MeO
F
O
N
NH
H
N
Cl
O
F
9.64
7.92 7.91 7.88 7.86
7.26 7.23 7.18 7.07
6.54 6.50 6.26
5.76 5.73 5.72
3.87
2.18 2.16 2.06
0.92 0.94 0.96 0.90 1.03 0.95 0.95 0.96 2.00 6.06 2.86 2.95 2.92
f1 (ppm)

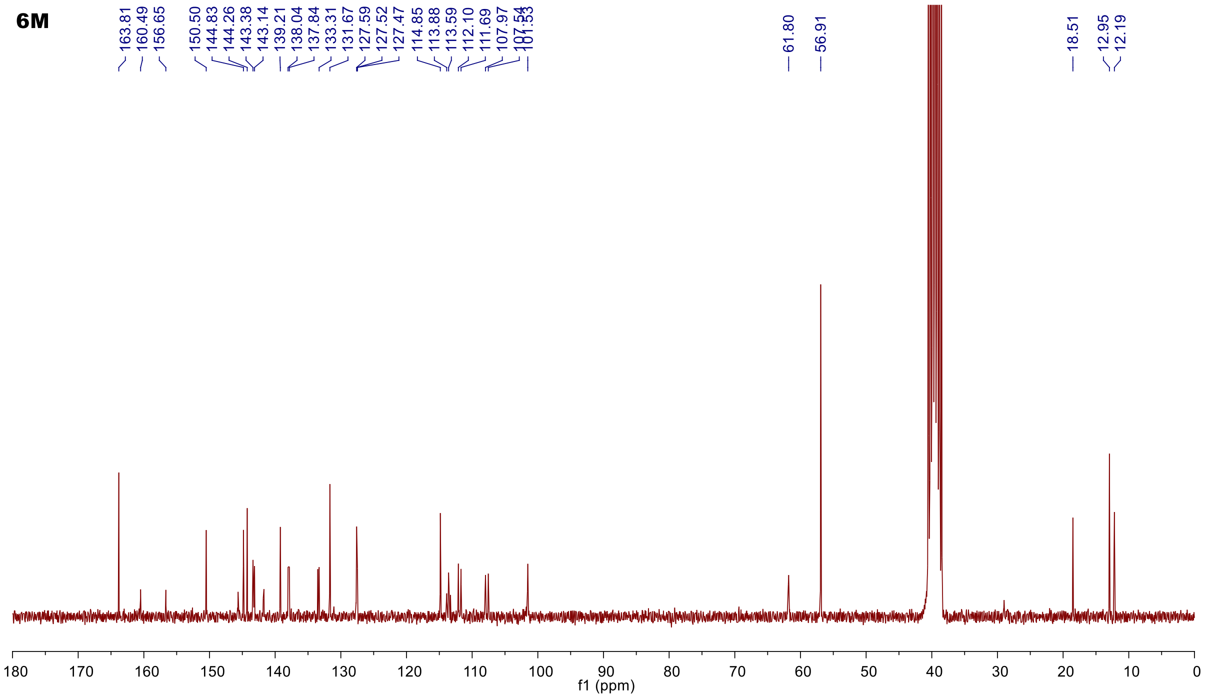
6M
163.81 160.49 156.65
150.50 144.83 144.26 143.38 143.14 139.21 138.04 137.84 133.31 131.67 127.59 127.52 127.47 114.85 113.88 113.59 112.10 111.69 107.97
61.80
56.91
18.51
12.95 12.19
f1 (ppm)

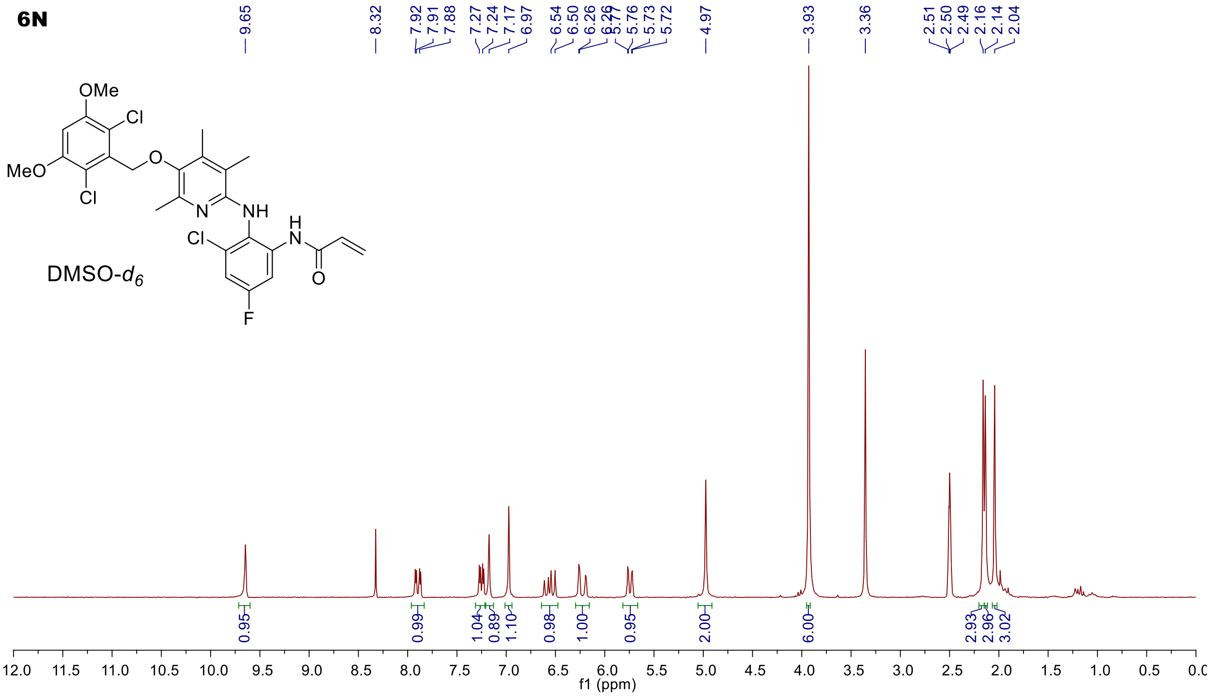
6N
DMSO-d_6
OMe
Cl
MeO
Cl
O
N
NH
H
N
Cl
O
F
9.65
8.32
7.92
7.91
7.88
7.27
7.24
7.17
6.97
6.54
6.50
6.26
6.26
5.77
5.76
5.73
5.72
4.97
3.93
3.36
2.51
2.50
2.49
2.16
2.14
2.04
0.95
0.99
1.04
0.89
1.10
0.98
1.00
0.95
2.00
6.00
2.93
2.96
3.02
f1 (ppm)

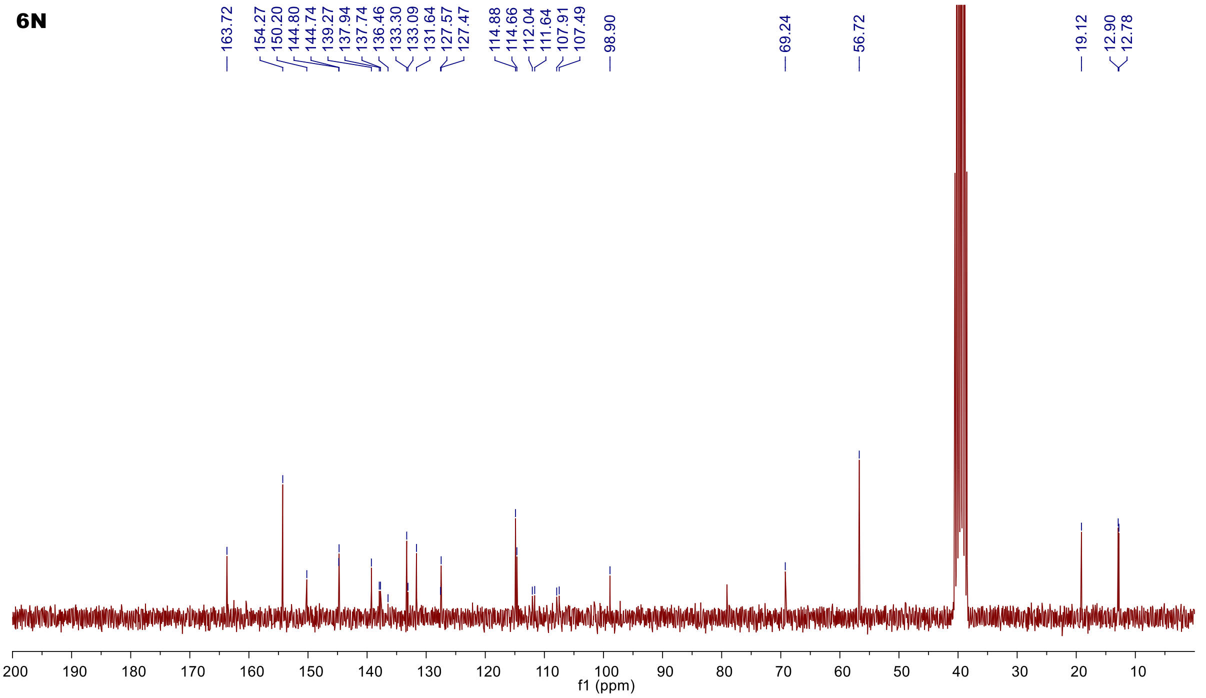
6N
163.72
154.27
150.20
144.80
144.74
139.27
137.94
137.74
136.46
133.30
133.09
131.64
127.57
127.47
114.88
114.66
112.04
111.64
107.91
107.49
98.90
69.24
56.72
19.12
12.90
12.78
f1 (ppm)

6O

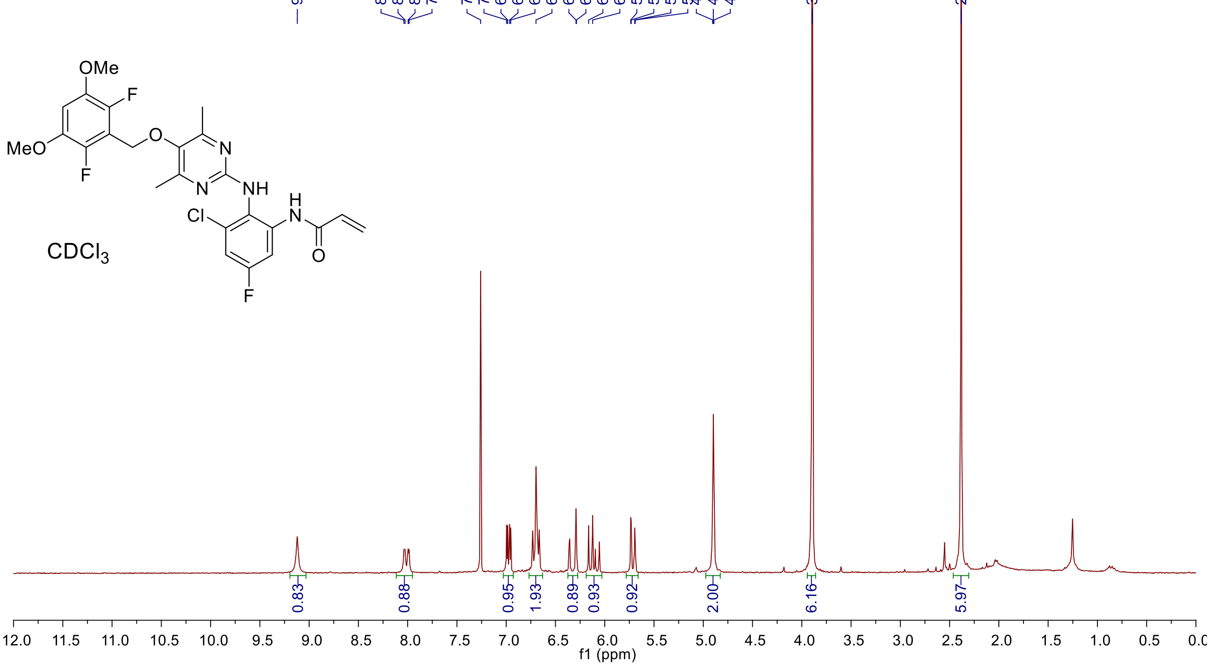

CDCl3
OMe
F
MeO
F
O
N
N
NH
H
N
Cl
O
F
9.12
8.04
8.03
8.00
7.99
7.26
7.00
6.98
6.97
6.95
6.70
6.29
6.29
6.16
6.12
5.74
5.73
5.70
4.90
4.89
0.83
0.88
0.95
1.93
0.89
0.93
0.92
2.00
6.16
5.97
f1 (ppm)

6O

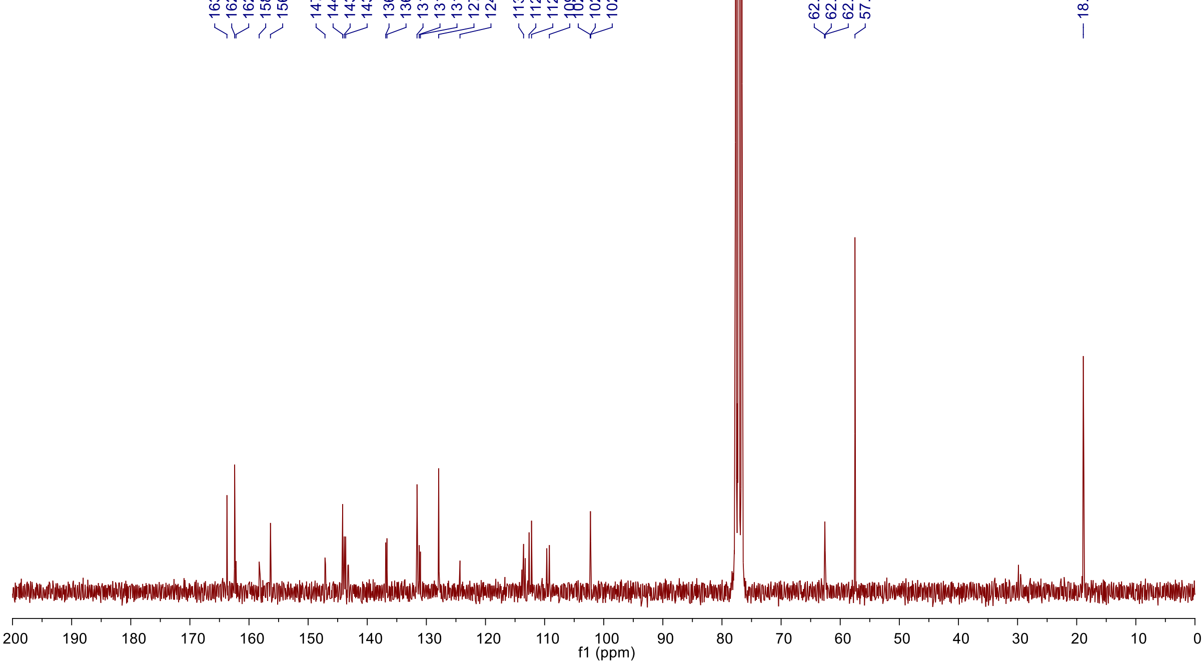

163.73
162.43
162.20
158.28
156.37
147.15
144.15
143.89
143.64
136.87
136.66
131.58
131.20
131.00
127.91
124.31
113.56
112.63
112.21
102.25
102.21
62.66
62.60
62.54
57.50
18.88
f1 (ppm)

6P

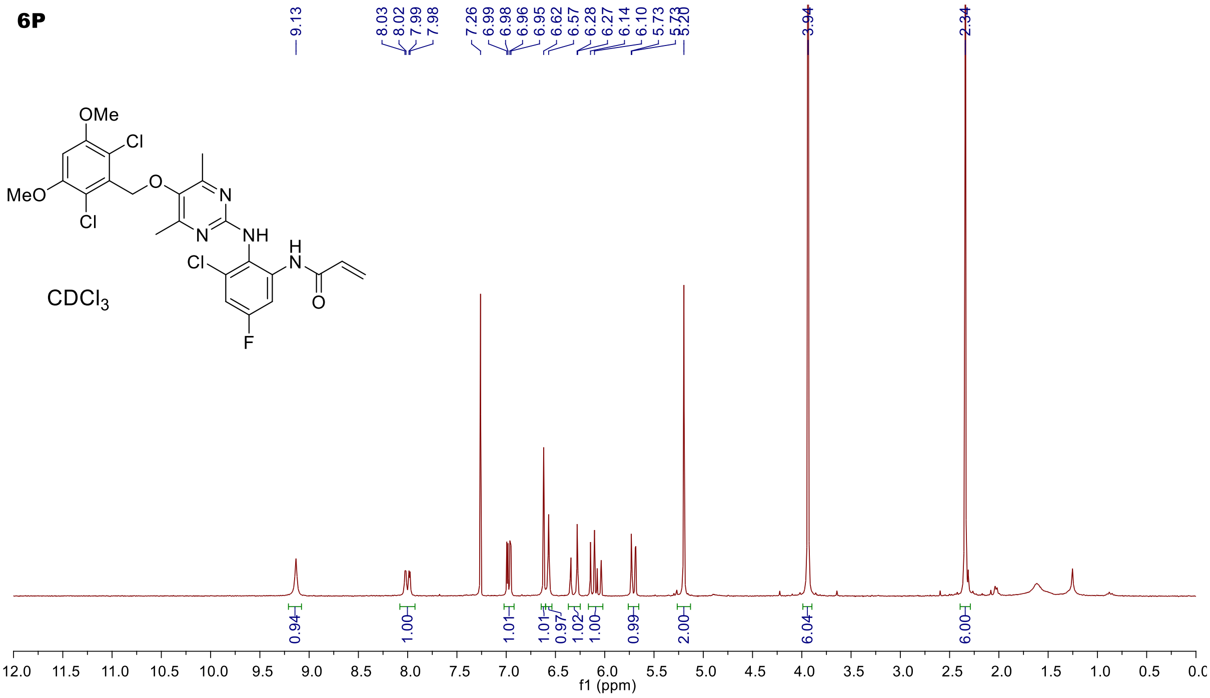

9.13
8.03
8.02
7.99
7.98
7.26
6.99
6.98
6.96
6.95
6.62
6.57
6.28
6.27
6.14
6.10
5.73
5.73
5.20
3.94
2.34
OMe
Cl
MeO
O
N
Cl
N
NH
H
N
Cl
O
F
CDCl$_3$
0.94
1.00
1.01
1.01
0.97
1.02
1.00
0.99
2.00
6.04
6.00
12.0 11.5 11.0 10.5 10.0 9.5 9.0 8.5 8.0 7.5 7.0 6.5 6.0 5.5 5.0 4.5 4.0 3.5 3.0 2.5 2.0 1.5 1.0 0.5 0.0
f1 (ppm)

6P

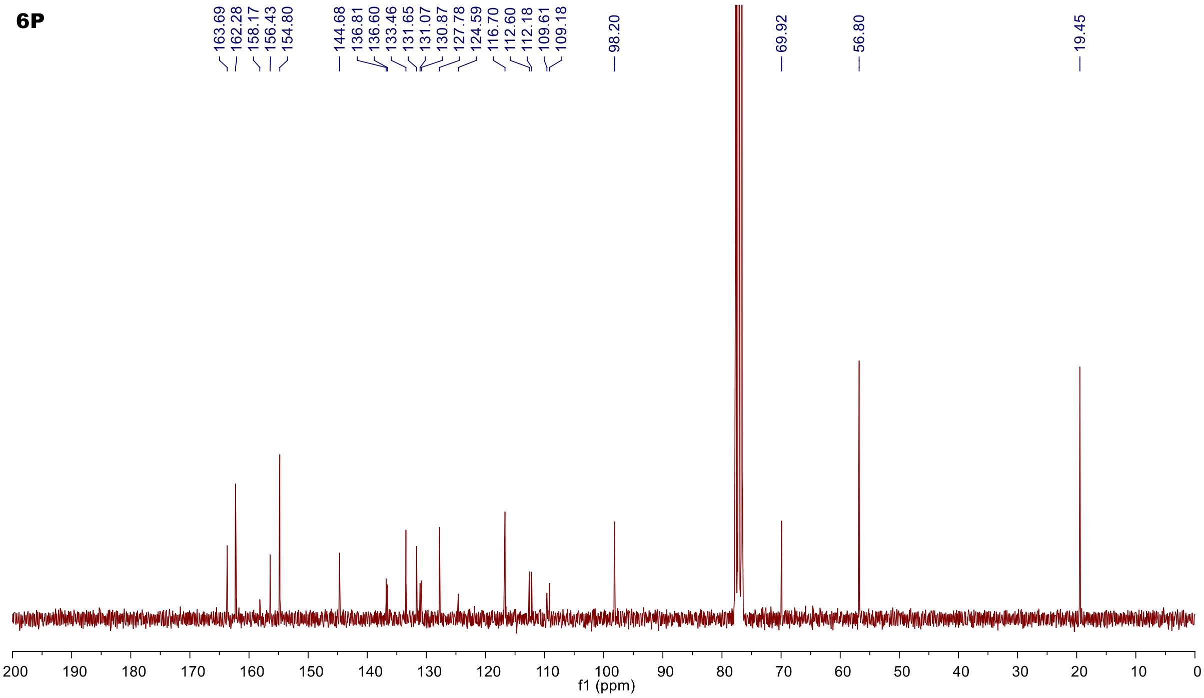

163.69
162.28
158.17
156.43
154.80
144.68
136.81
136.60
133.46
131.65
131.07
130.87
127.78
124.59
116.70
112.60
112.18
109.61
109.18
98.20
69.92
56.80
19.45
200 190 180 170 160 150 140 130 120 110 100 90 80 70 60 50 40 30 20 10 0
f1 (ppm)

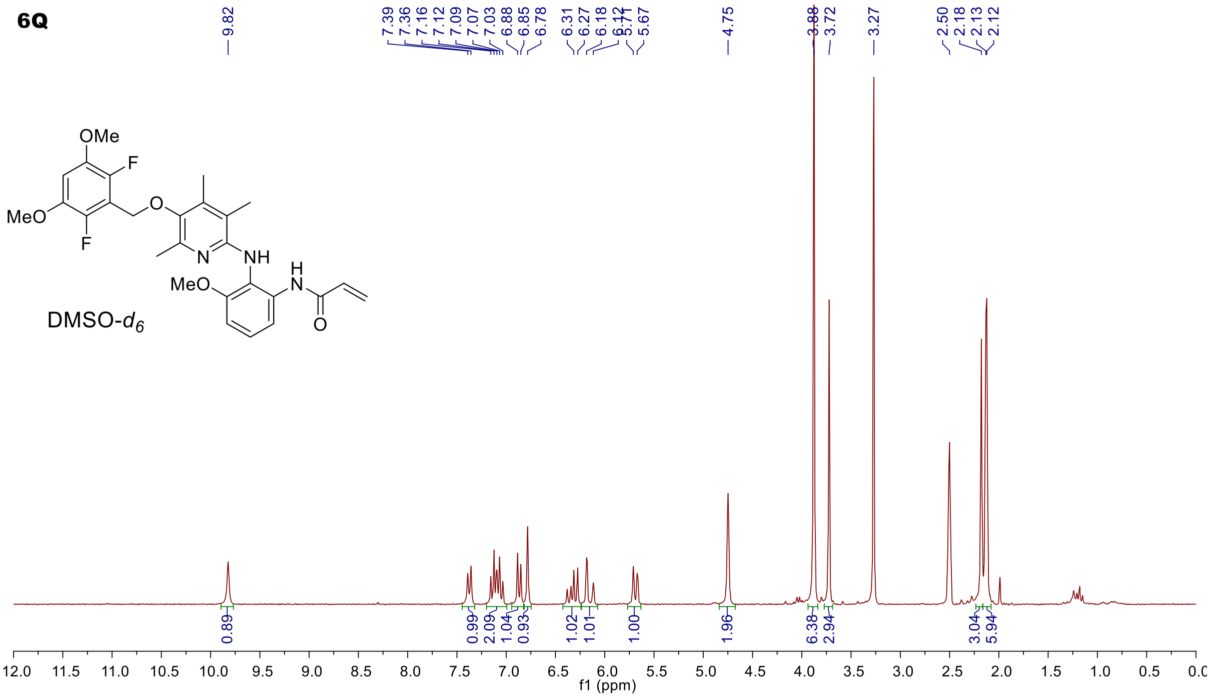
6Q
DMSO-d_6
9.82
7.39
7.36
7.16
7.12
7.09
7.07
7.03
6.88
6.85
6.78
6.31
6.27
6.18
6.12
5.71
5.67
4.75
3.88
3.72
3.27
2.50
2.18
2.13
2.12
0.89
0.99
2.09
1.04
0.93
1.02
1.01
1.00
1.96
6.38
2.94
3.04
5.94
f1 (ppm)

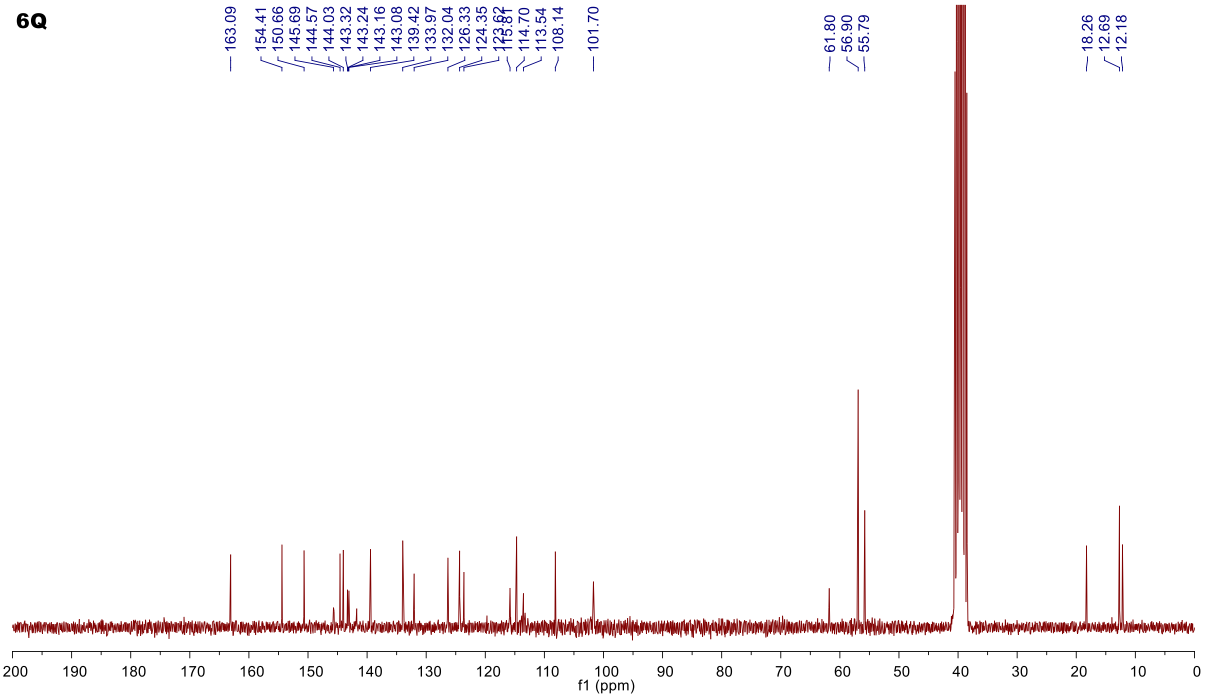
6Q
163.09
154.41
150.66
145.69
144.57
144.03
143.32
143.24
143.16
143.08
139.42
133.97
132.04
126.33
124.35
123.62
115.81
114.70
113.54
108.14
101.70
61.80
56.90
55.79
18.26
12.69
12.18
f1 (ppm)

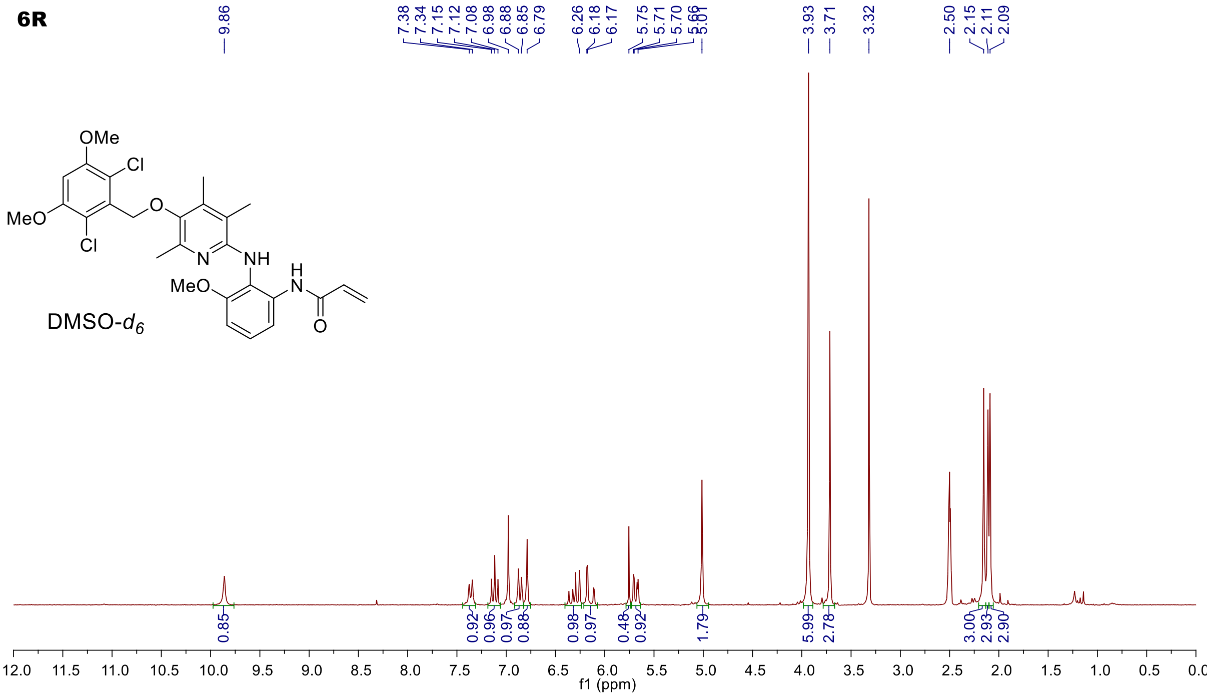
6R
DMSO-d_6
9.86
7.38 7.34 7.15 7.12 7.08 6.98 6.88 6.85 6.79
6.26 6.18 6.17
5.75 5.71 5.70 5.66 5.01
3.93 3.71 3.32
2.50 2.15 2.11 2.09
0.85 0.92 0.96 0.97 0.88 0.98 0.97 0.48 0.92 1.79 5.99 2.78 3.00 2.93 2.90
f1 (ppm)

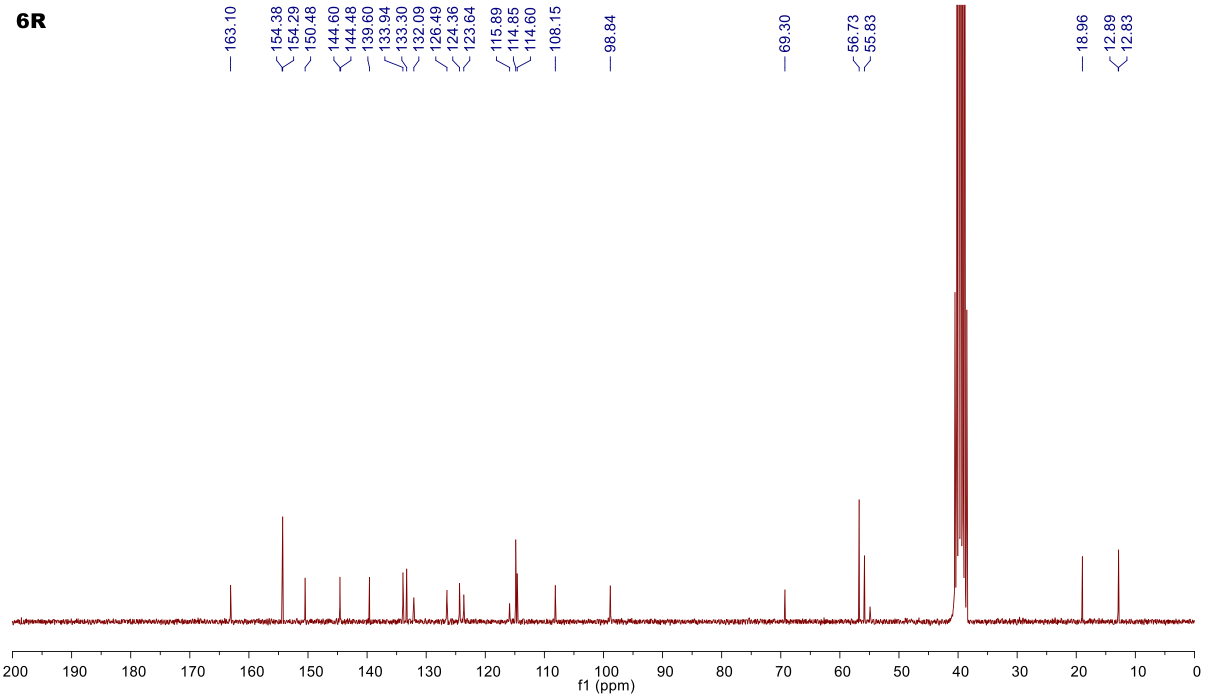
6R
163.10
154.38 154.29 150.48 144.60 144.48 139.60 133.94 133.30 132.09 126.49 124.36 123.64 115.89 114.85 114.60 108.15
98.84
69.30
56.73 55.83
18.96 12.89 12.83
f1 (ppm)

6S

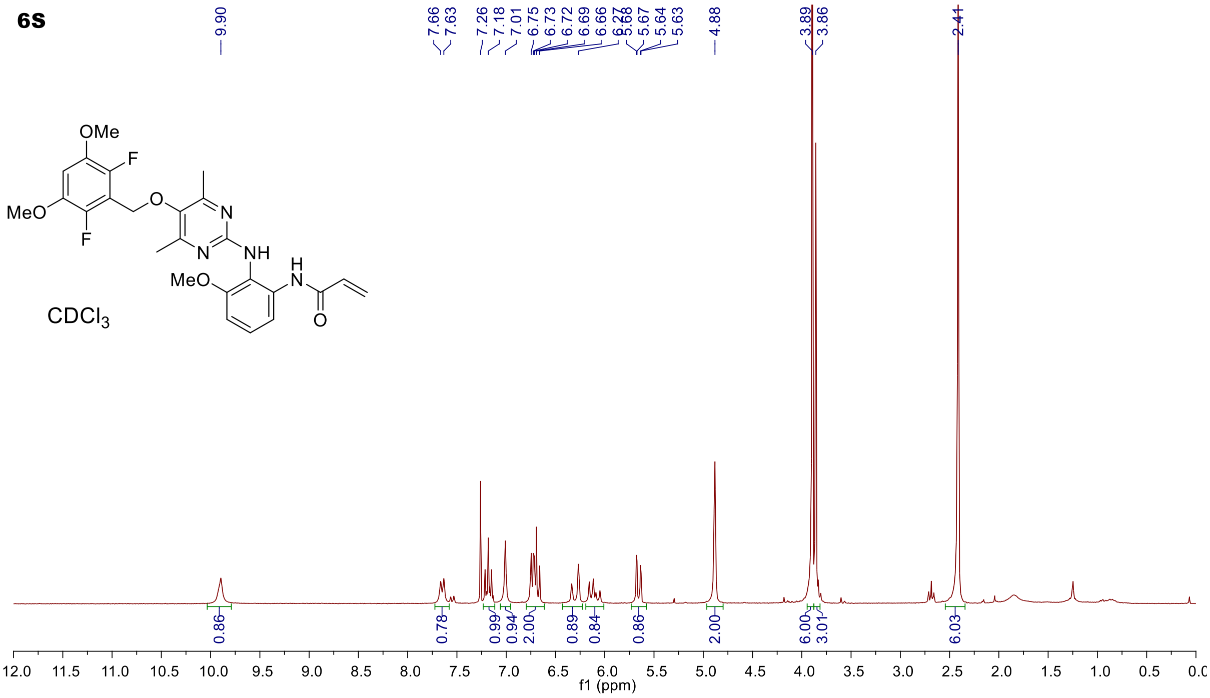
9.90
7.66
7.63
7.26
7.18
7.01
6.75
6.73
6.72
6.69
6.66
6.27
5.68
5.67
5.64
5.63
4.88
3.89
3.86
2.41
OMe
F
O
MeO
F
N
N
NH
H
N
MeO
O
CDCl3
0.86
0.78
0.99
0.94
2.00
0.89
0.84
0.86
2.00
6.00
3.01
6.03
12.0 11.5 11.0 10.5 10.0 9.5 9.0 8.5 8.0 7.5 7.0 6.5 6.0 5.5 5.0 4.5 4.0 3.5 3.0 2.5 2.0 1.5 1.0 0.5 0.0
f1 (ppm)

6S

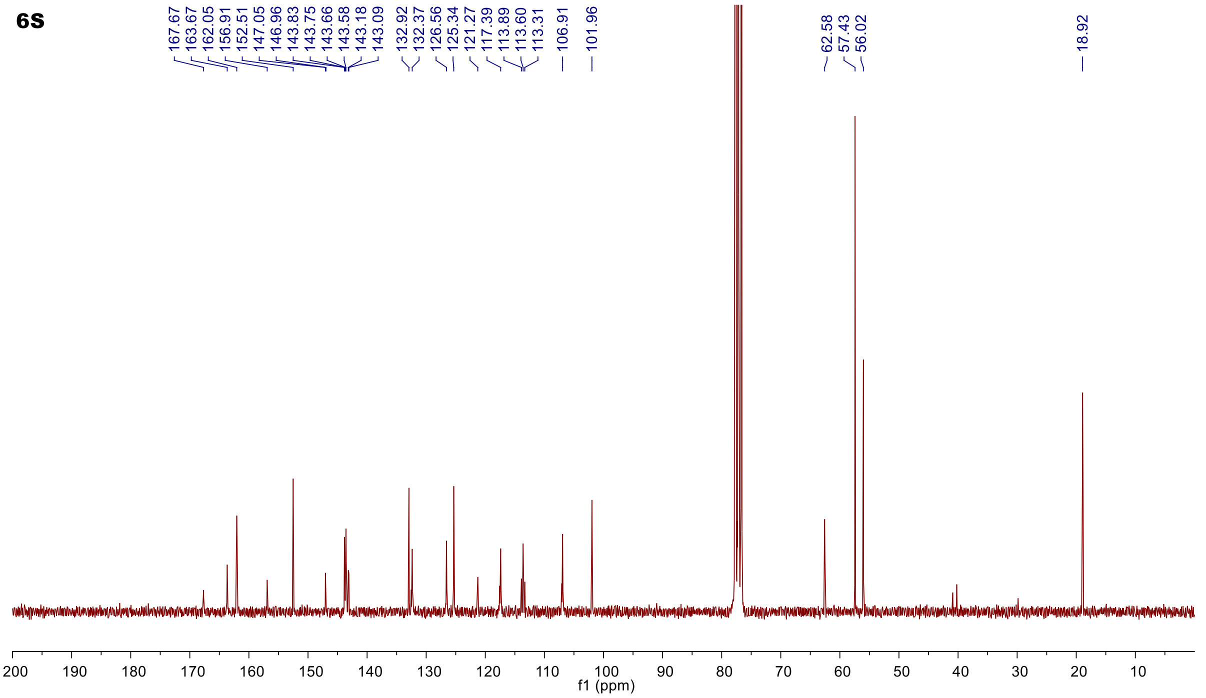
167.67
163.67
162.05
156.91
152.51
147.05
146.96
143.83
143.75
143.66
143.58
143.18
143.09
132.92
132.37
126.56
125.34
121.27
117.39
113.89
113.60
113.31
106.91
101.96
62.58
57.43
56.02
18.92
200 190 180 170 160 150 140 130 120 110 100 90 80 70 60 50 40 30 20 10
f1 (ppm)

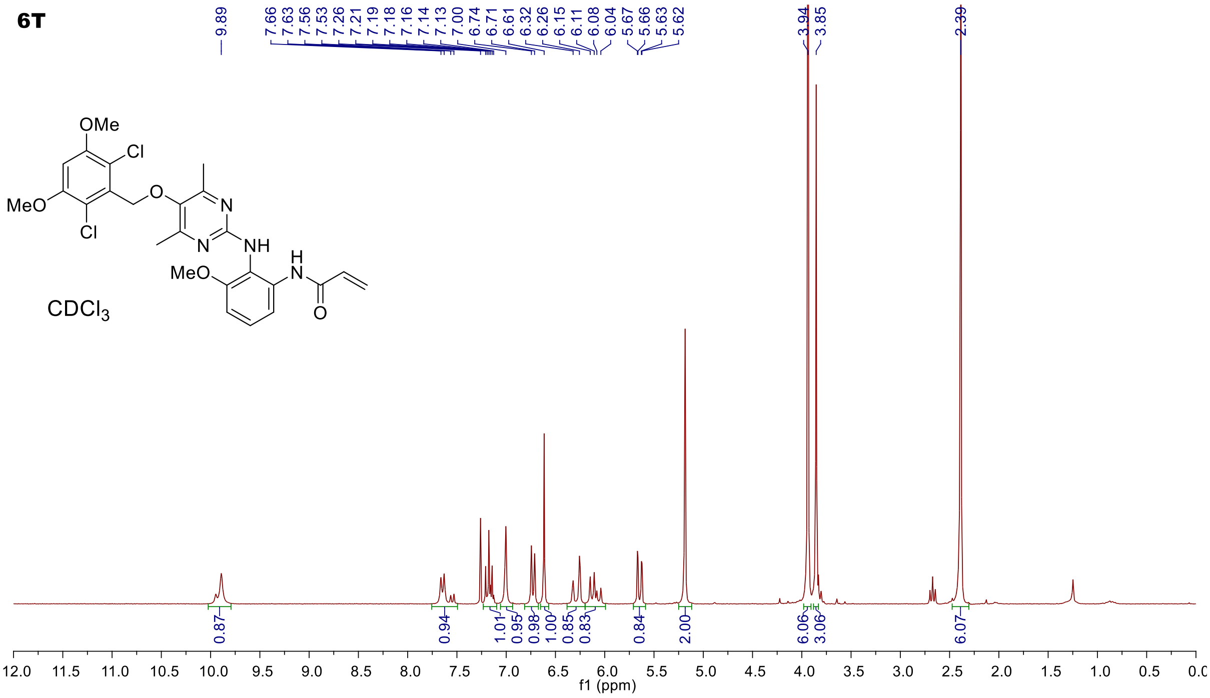
6T
9.89
7.66 7.63 7.56 7.53 7.26 7.21 7.19 7.18 7.16 7.14 7.13 7.00 6.74 6.71 6.61 6.32 6.26 6.15 6.11 6.08 6.04 5.67 5.66 5.63 5.62
3.94 3.85
2.39
OMe
Cl
MeO
O
N
Cl
N
NH
H
N
MeO
O
CDCl$_3$
0.87 0.94 1.01 0.95 0.98 1.00 0.85 0.83 0.84 2.00 6.06 3.06 6.07
f1 (ppm)

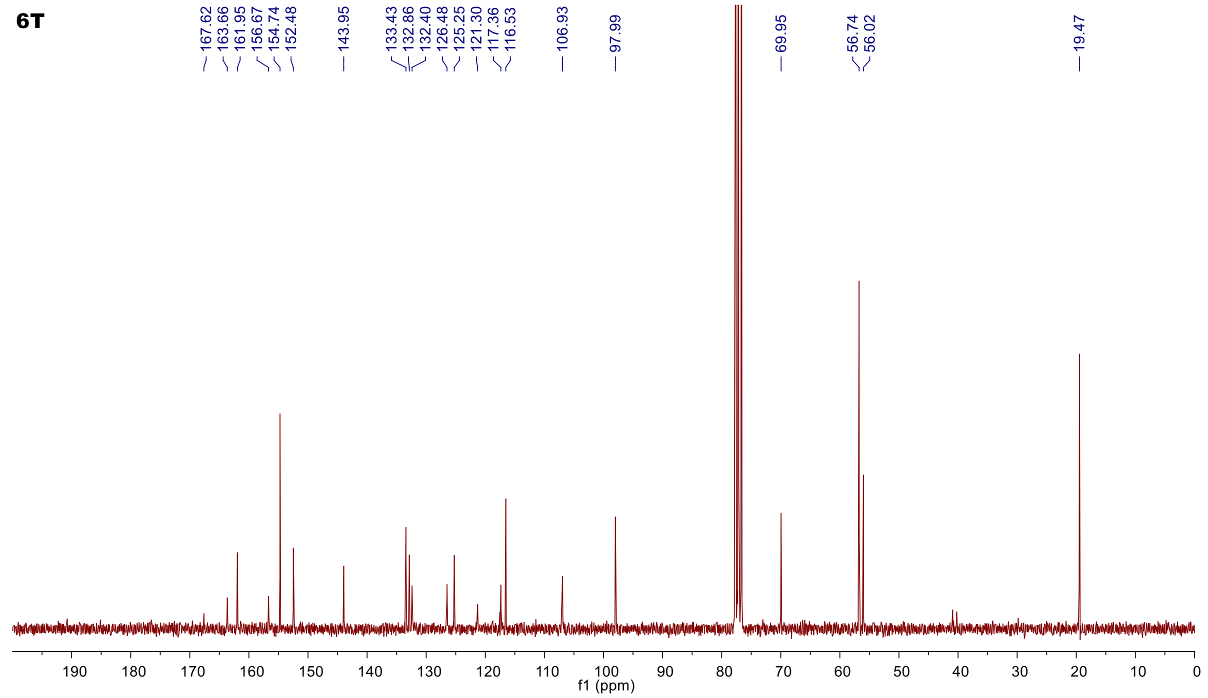
6T
167.62 163.66 161.95 156.67 154.74 152.48
143.95
133.43 132.86 132.40 126.48 125.25 121.30 117.36 116.53
106.93
97.99
69.95
56.74 56.02
19.47
f1 (ppm)

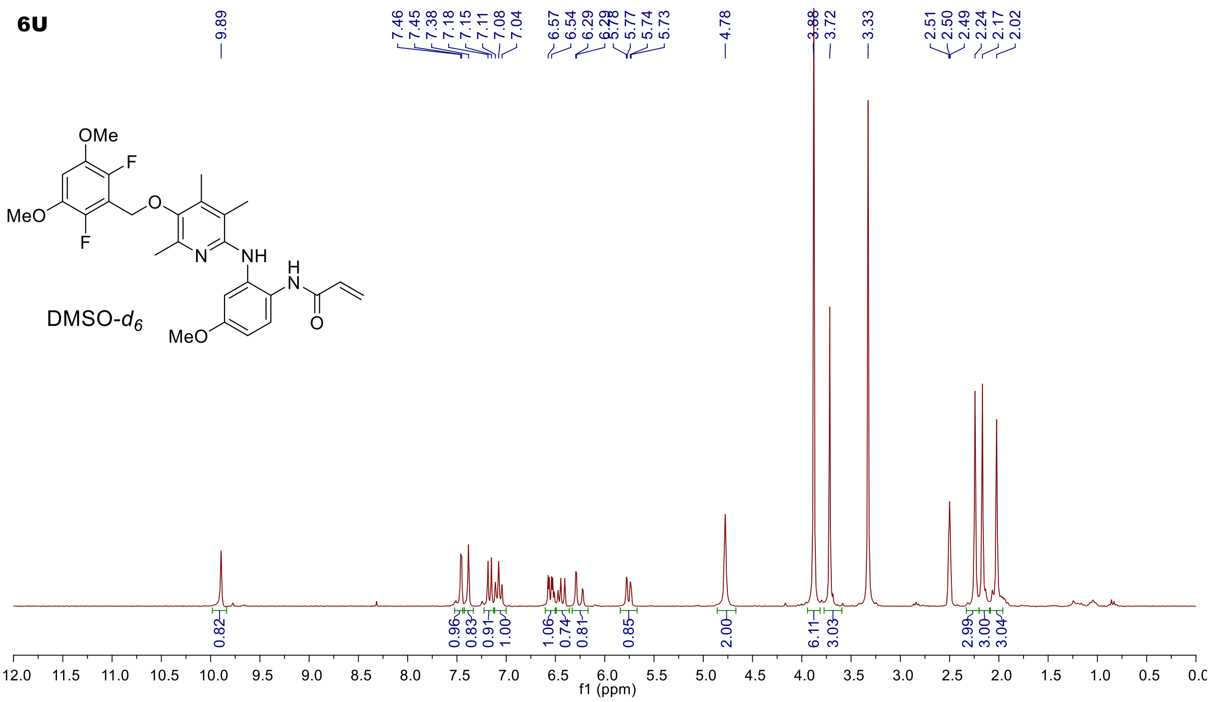

6U
9.89
7.46
7.45
7.38
7.18
7.15
7.11
7.08
7.04
6.57
6.54
6.29
6.29
5.78
5.77
5.74
5.73
4.78
3.88
3.72
3.33
2.51
2.50
2.49
2.24
2.17
2.02
OMe
F
MeO
F
O
N
NH
H
N
O
MeO
DMSO-d_6
0.82
0.96
0.83
0.91
1.00
1.06
0.74
0.81
0.85
2.00
6.11
3.03
2.99
3.00
3.04
12.0 11.5 11.0 10.5 10.0 9.5 9.0 8.5 8.0 7.5 7.0 6.5 6.0 5.5 5.0 4.5 4.0 3.5 3.0 2.5 2.0 1.5 1.0 0.5 0.0
f1 (ppm)

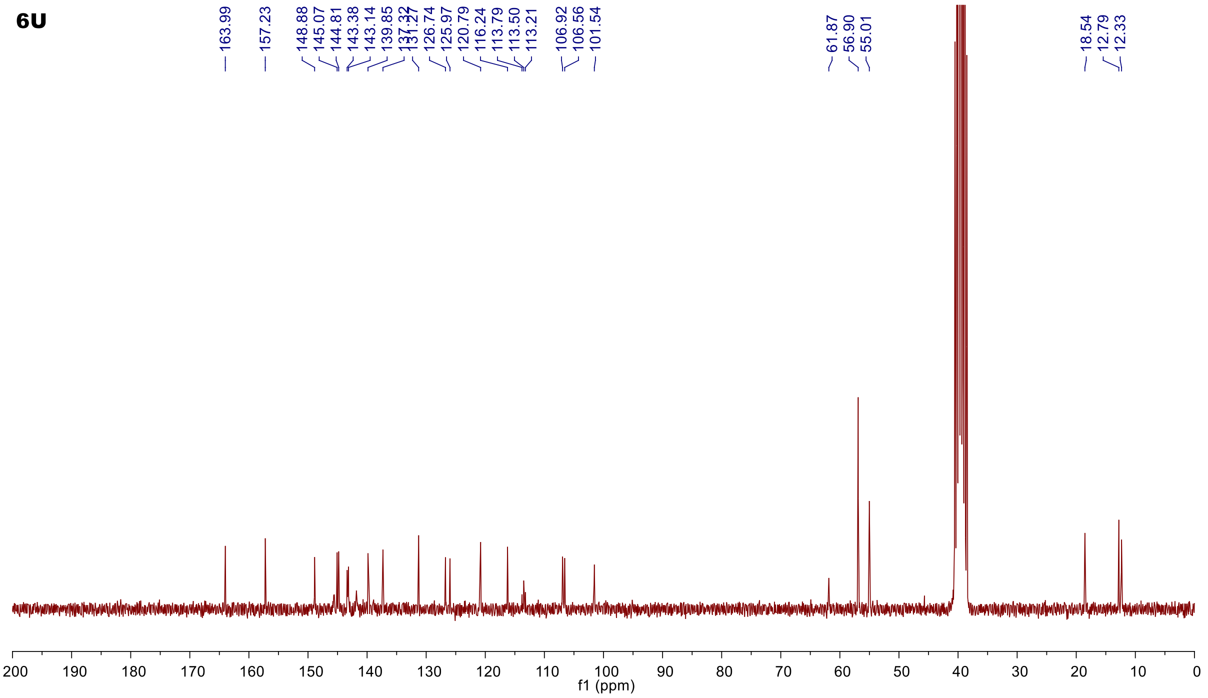

6U
163.99
157.23
148.88
145.07
144.81
143.38
143.14
139.85
137.32
131.27
126.74
125.97
120.79
116.24
113.79
113.50
113.21
106.92
106.56
101.54
61.87
56.90
55.01
18.54
12.79
12.33
200 190 180 170 160 150 140 130 120 110 100 90 80 70 60 50 40 30 20 10 0
f1 (ppm)

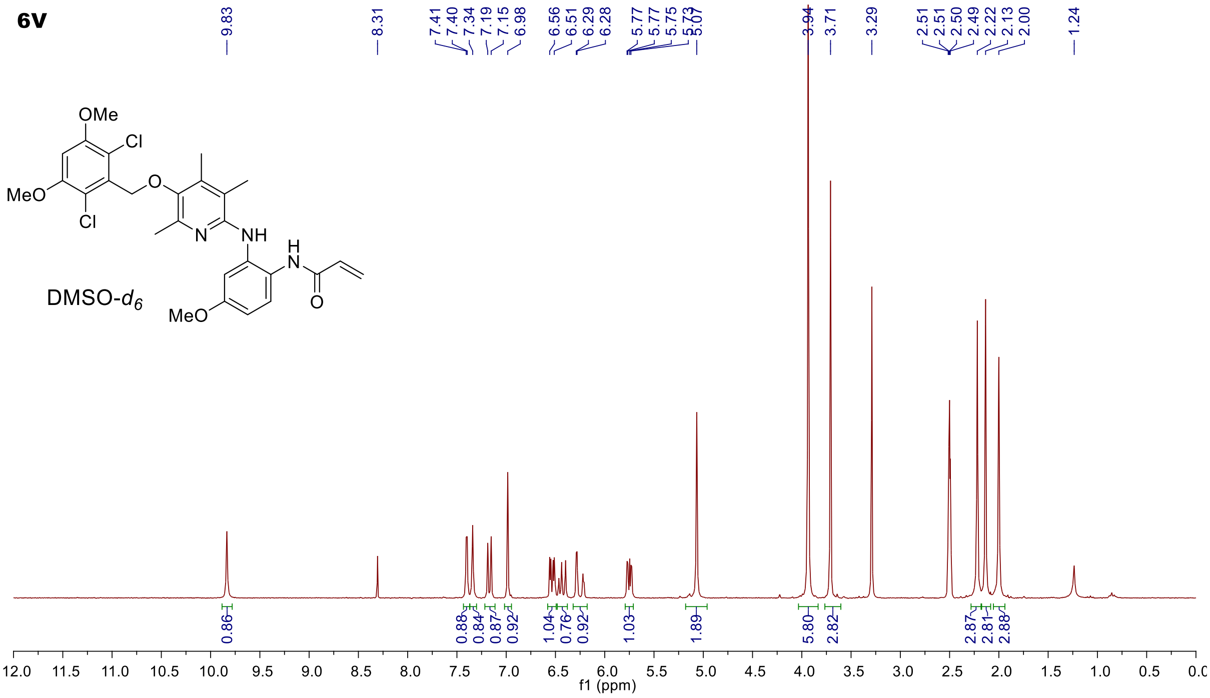
6V
DMSO-d_6
OMe
Cl
MeO
Cl
O
N
NH
H
N
O
MeO
9.83
8.31
7.41
7.40
7.34
7.19
7.15
6.98
6.56
6.51
6.29
6.28
5.77
5.77
5.75
5.73
5.07
3.94
3.71
3.29
2.51
2.51
2.50
2.49
2.22
2.13
2.00
1.24
0.86
0.88
0.84
0.87
0.92
1.04
0.76
0.92
1.03
1.89
5.80
2.82
2.87
2.81
2.88
12.0 11.5 11.0 10.5 10.0 9.5 9.0 8.5 8.0 7.5 7.0 6.5 6.0 5.5 5.0 4.5 4.0 3.5 3.0 2.5 2.0 1.5 1.0 0.5 0.0
f1 (ppm)

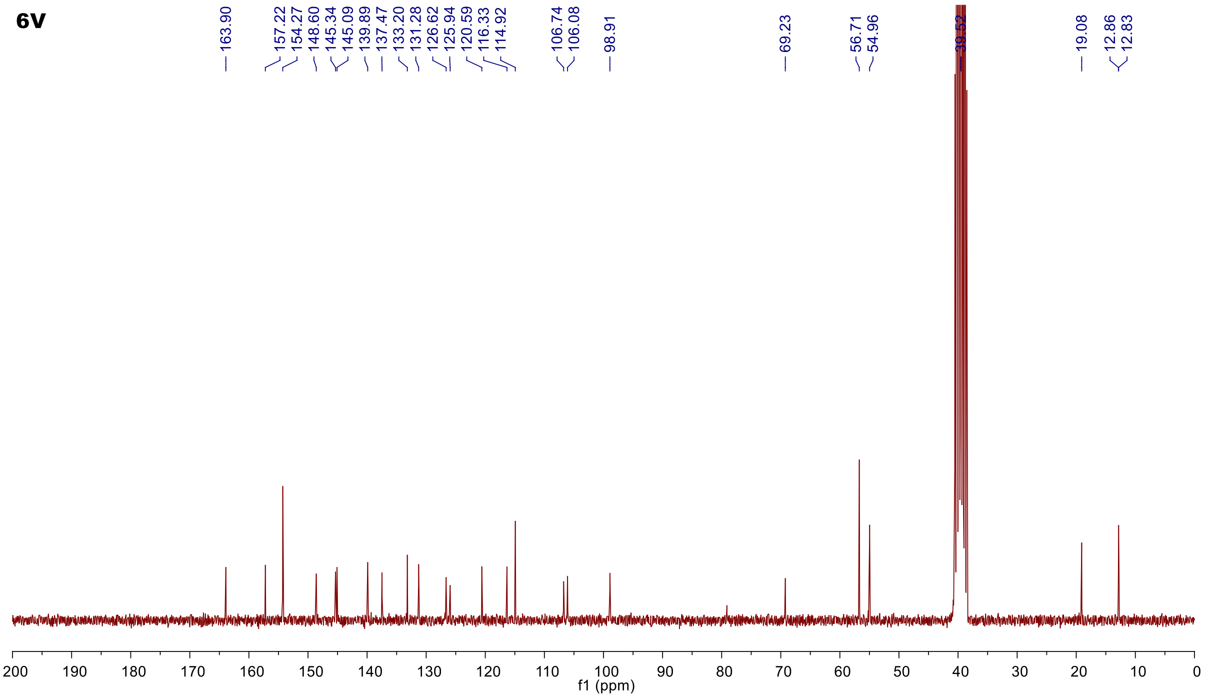
6V
163.90
157.22
154.27
148.60
145.34
145.09
139.89
137.47
133.20
131.28
126.62
125.94
120.59
116.33
114.92
106.74
106.08
98.91
69.23
56.71
54.96
39.52
19.08
12.86
12.83
200 190 180 170 160 150 140 130 120 110 100 90 80 70 60 50 40 30 20 10 0
f1 (ppm)

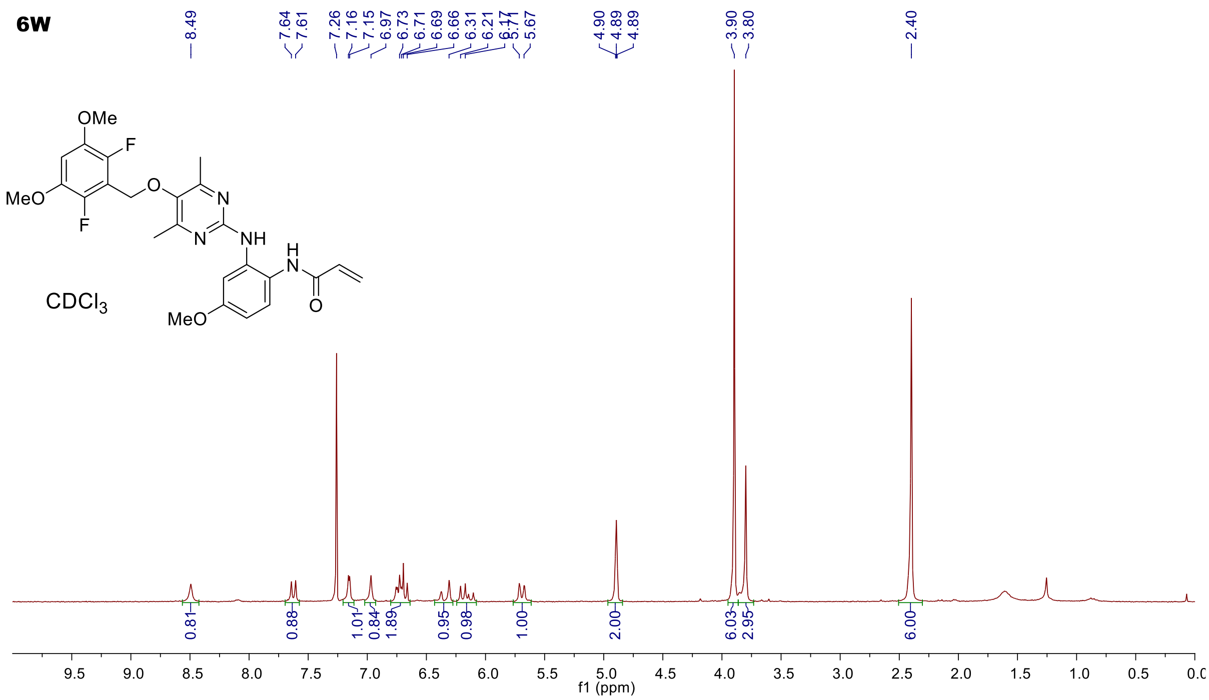
6W
CDCl3
8.49
7.64
7.61
7.26
7.16
7.15
6.97
6.73
6.71
6.69
6.66
6.31
6.21
6.17
5.71
5.67
4.90
4.89
4.89
3.90
3.80
2.40
0.81
0.88
1.01
0.84
1.89
0.95
0.98
1.00
2.00
6.03
2.95
6.00
f1 (ppm)

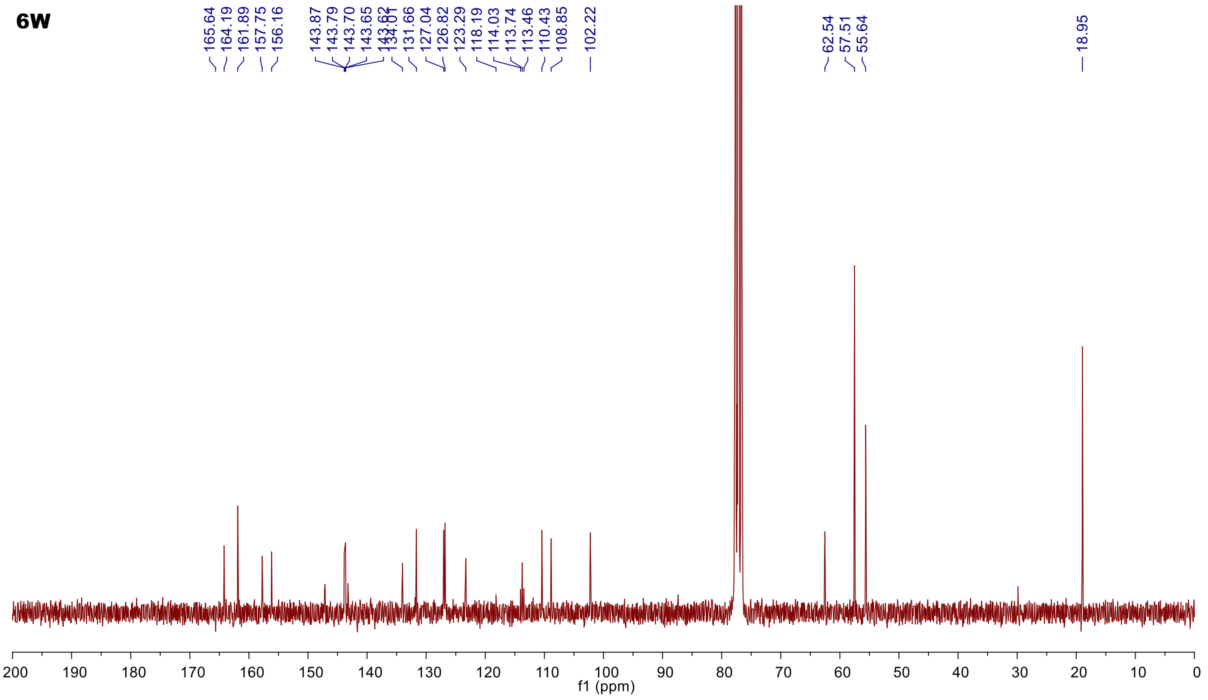
6W
165.64
164.19
161.89
157.75
156.16
143.87
143.79
143.70
143.65
143.62
134.01
131.66
127.04
126.82
123.29
118.19
114.03
113.74
113.46
110.43
108.85
102.22
62.54
57.51
55.64
18.95
f1 (ppm)

6X

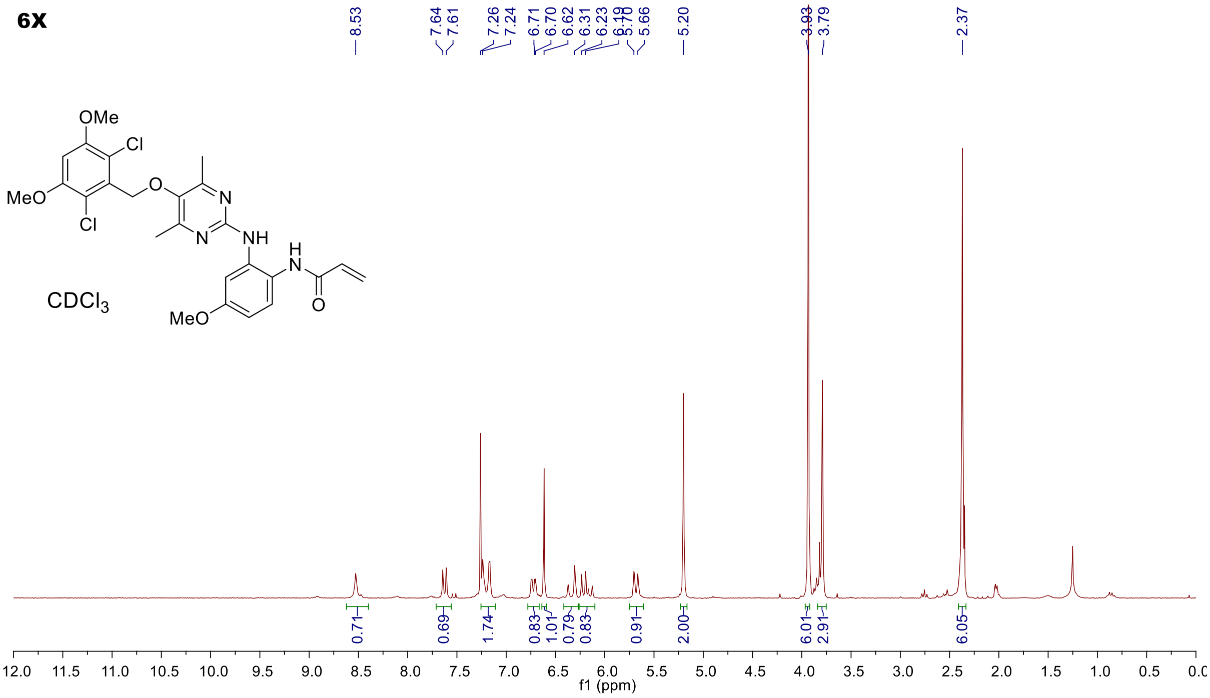
8.53
7.64
7.61
7.26
7.24
6.71
6.70
6.62
6.31
6.23
6.19
5.70
5.66
5.20
3.93
3.79
2.37
OMe
Cl
MeO
O
N
N
NH
Cl
H
N
O
MeO
CDCl3
0.71
0.69
1.74
0.83
1.01
0.79
0.83
0.91
2.00
6.01
2.91
6.05
f1 (ppm)

6X

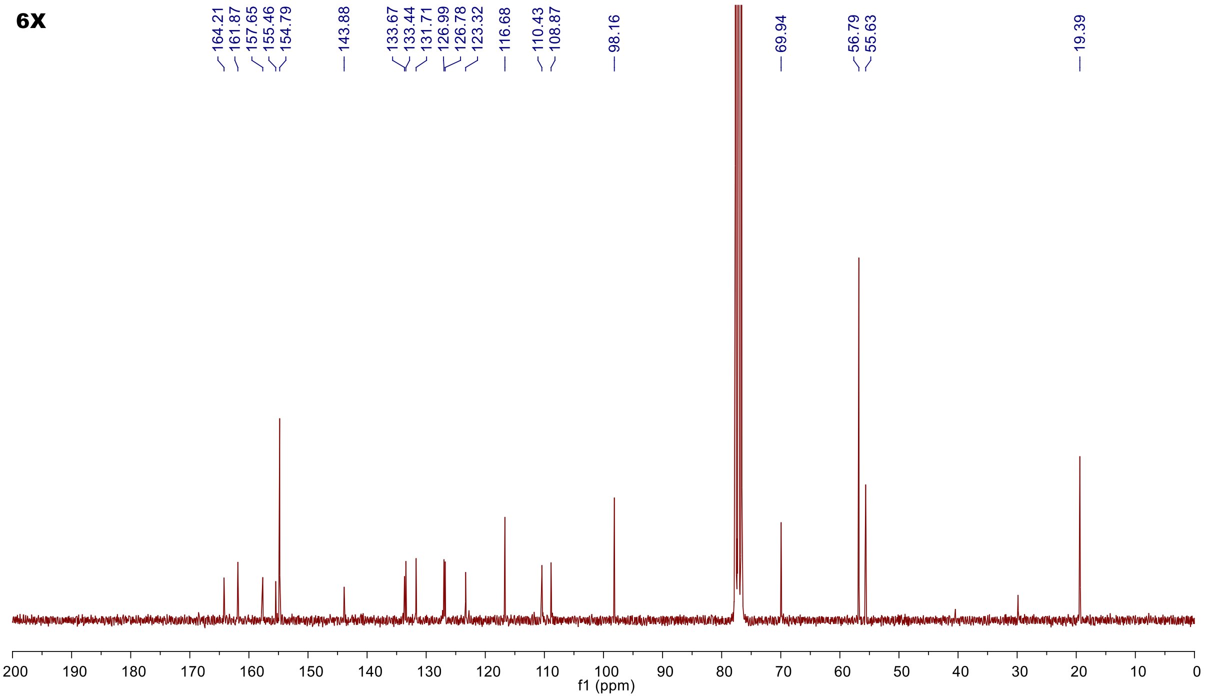
164.21
161.87
157.65
155.46
154.79
143.88
133.67
133.44
131.71
126.99
126.78
123.32
116.68
110.43
108.87
98.16
69.94
56.79
55.63
19.39
f1 (ppm)

6Y

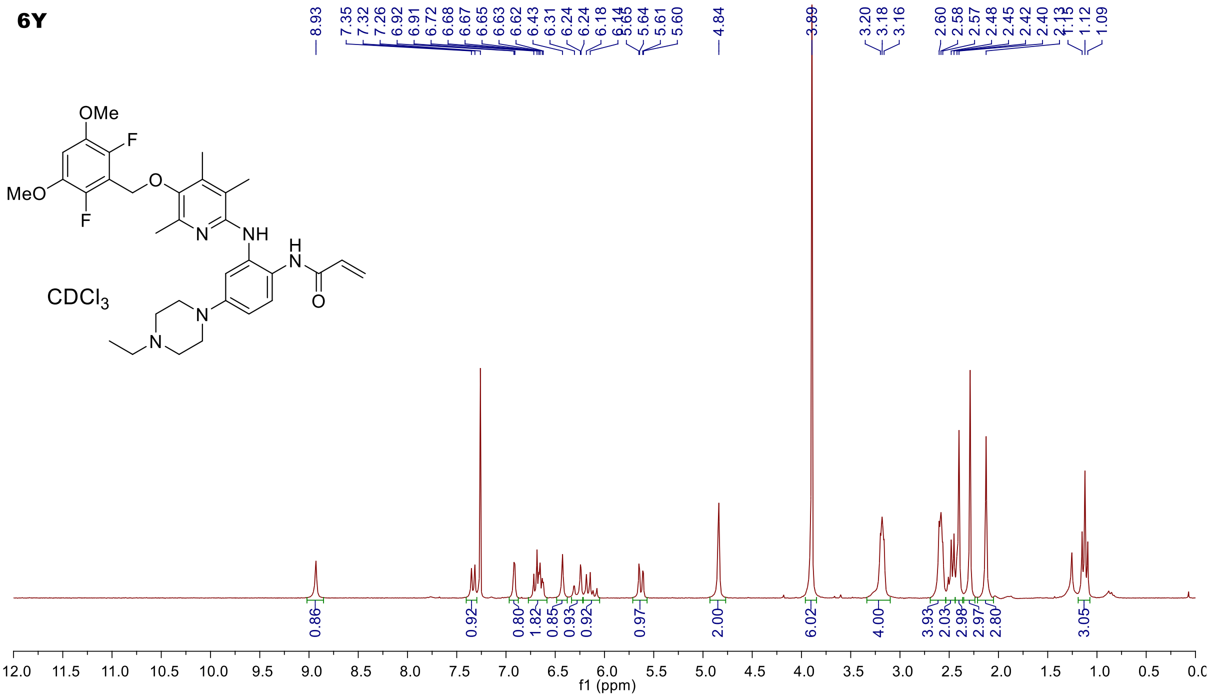

8.93
7.35
7.32
7.26
6.92
6.91
6.72
6.68
6.67
6.65
6.63
6.62
6.43
6.31
6.24
6.24
6.18
6.14
5.65
5.64
5.61
5.60
4.84
3.89
3.20
3.18
3.16
2.60
2.58
2.57
2.48
2.45
2.42
2.40
2.13
1.15
1.12
1.09
OMe
F
MeO
O
F
N
NH
H
N
O
N
N
CDCl3
0.86
0.92
0.80
1.82
0.85
0.93
0.92
0.97
2.00
6.02
4.00
3.93
2.03
2.98
2.97
2.80
3.05
12.0 11.5 11.0 10.5 10.0 9.5 9.0 8.5 8.0 7.5 7.0 6.5 6.0 5.5 5.0 4.5 4.0 3.5 3.0 2.5 2.0 1.5 1.0 0.5 0.0
f1 (ppm)

6Y

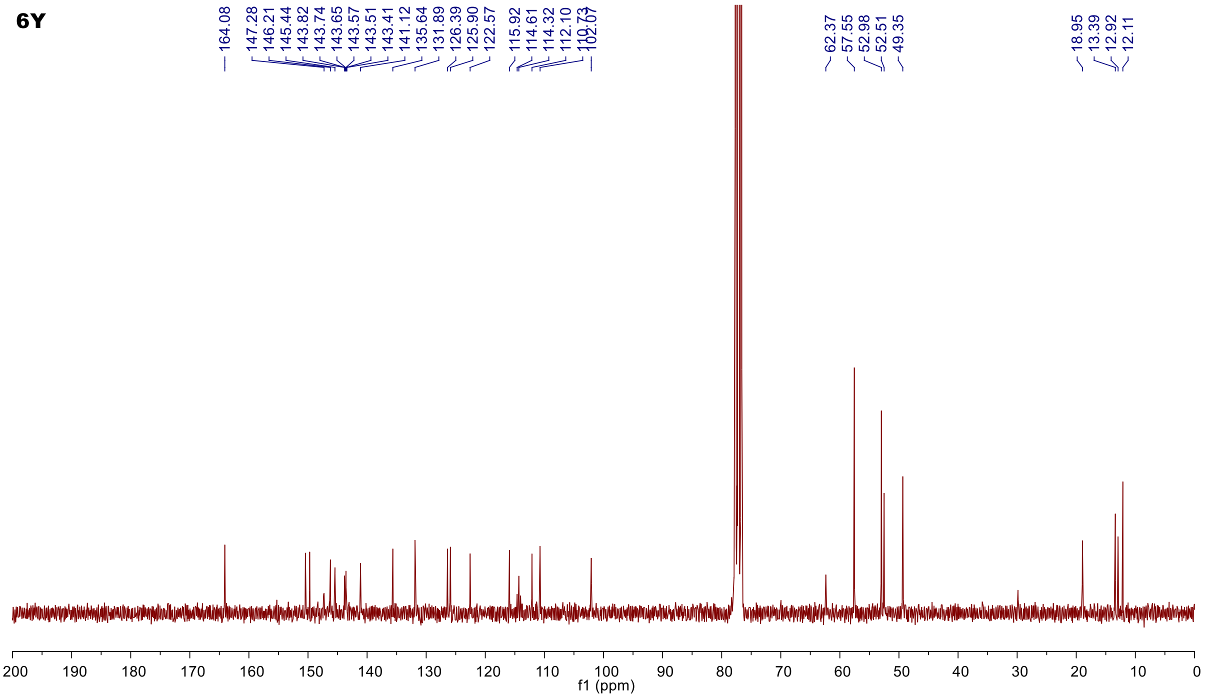

164.08
147.28
146.21
145.44
143.82
143.74
143.65
143.57
143.51
143.41
141.12
135.64
131.89
126.39
125.90
122.57
115.92
114.61
114.32
112.10
110.73
102.07
62.37
57.55
52.98
52.51
49.35
18.95
13.39
12.92
12.11
200 190 180 170 160 150 140 130 120 110 100 90 80 70 60 50 40 30 20 10 0
f1 (ppm)

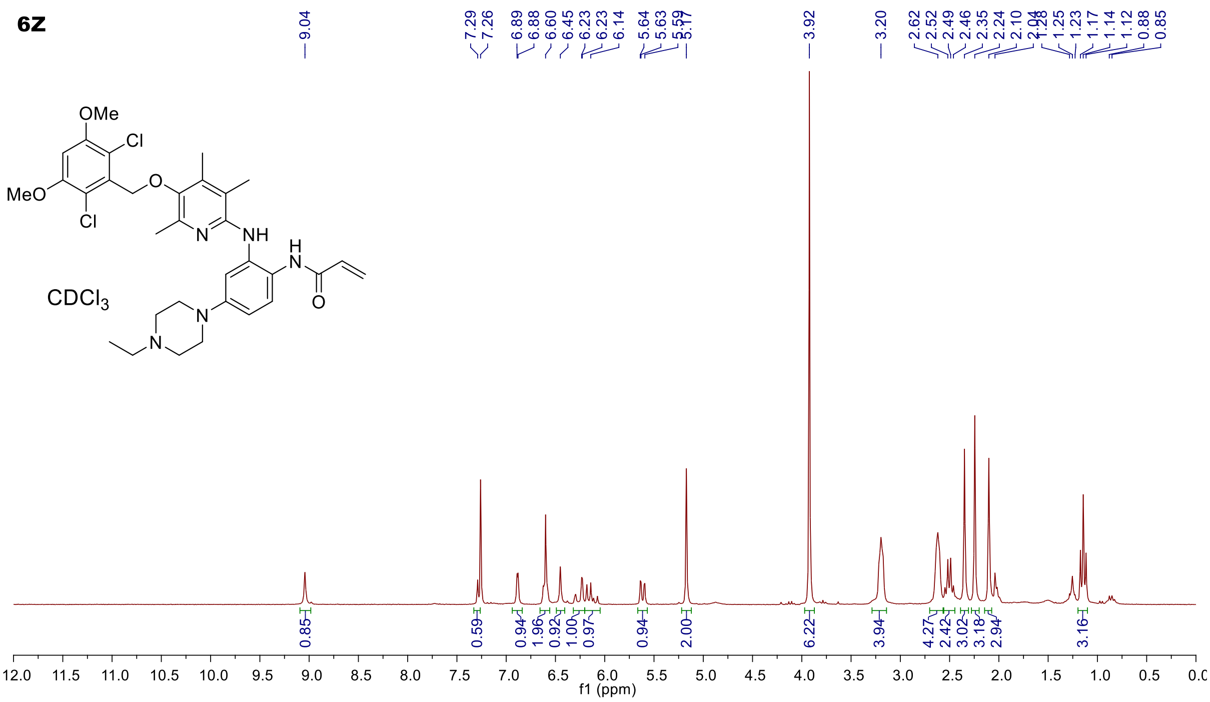
6Z
9.04
7.29
7.26
6.89
6.88
6.60
6.45
6.23
6.23
6.14
5.64
5.63
5.17
3.92
3.20
2.62
2.52
2.49
2.46
2.35
2.24
2.10
1.25
1.23
1.17
1.14
1.12
0.88
0.85
OMe
Cl
MeO
O
Cl
N
NH
H
N
O
N
N
CDCl3
0.85
0.59
0.94
1.96
0.92
1.00
0.97
0.94
2.00
6.22
3.94
4.27
2.42
3.02
3.18
2.94
3.16
f1 (ppm)

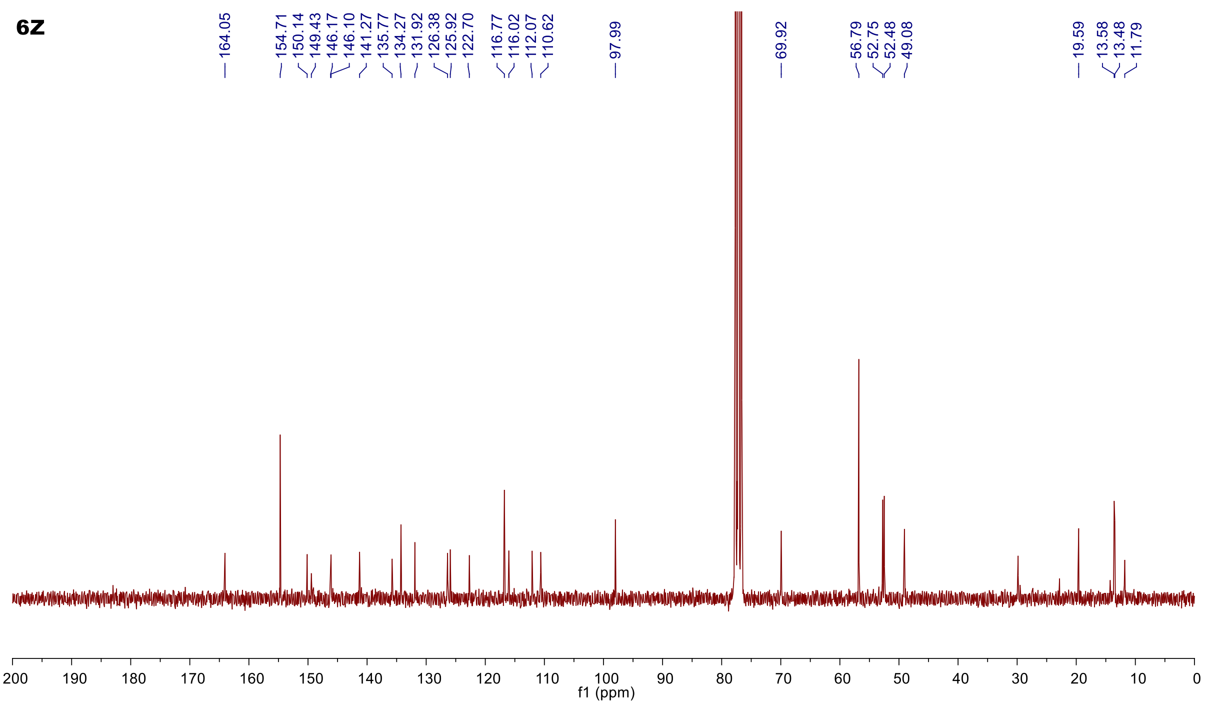
6Z
164.05
154.71
150.14
149.43
146.17
146.10
141.27
135.77
134.27
131.92
126.38
125.92
122.70
116.77
116.02
112.07
110.62
97.99
69.92
56.79
52.75
52.48
49.08
19.59
13.58
13.48
11.79
f1 (ppm)

6AA

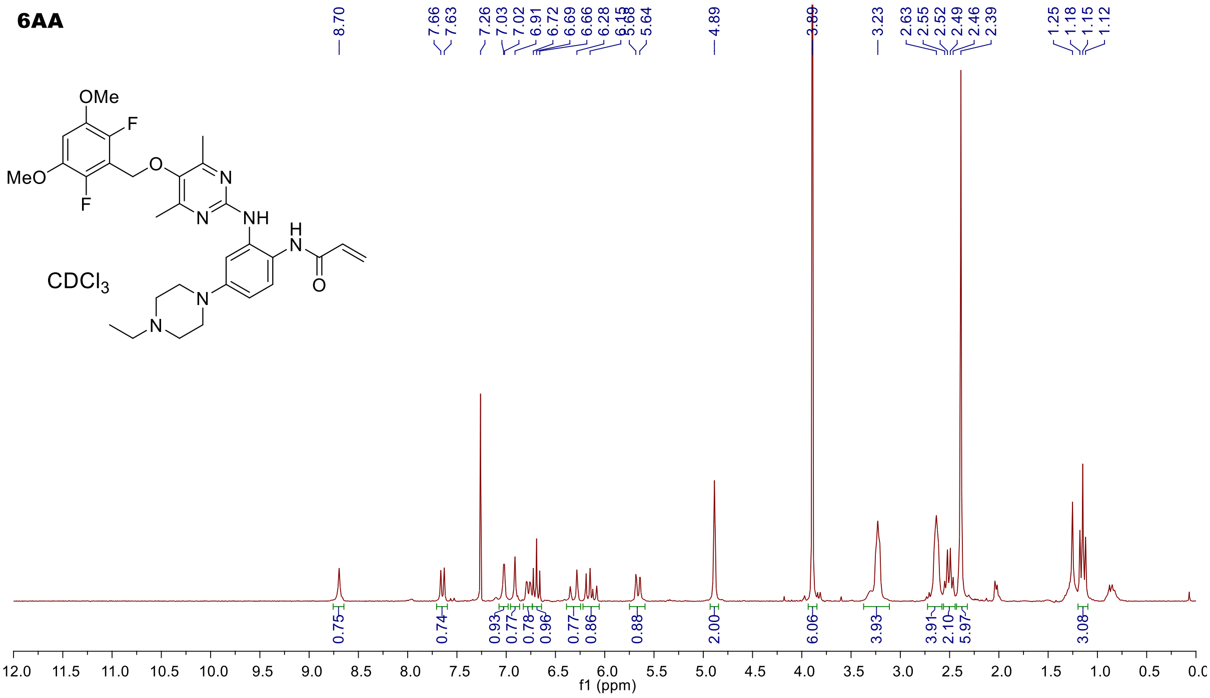

CDCl3
8.70
7.66
7.63
7.26
7.03
7.02
6.91
6.72
6.69
6.66
6.28
6.15
5.68
5.64
4.89
3.89
3.23
2.63
2.55
2.52
2.49
2.46
2.39
1.25
1.18
1.15
1.12
0.75
0.74
0.93
0.77
0.78
0.96
0.77
0.86
0.88
2.00
6.06
3.93
3.91
2.10
5.97
3.08
f1 (ppm)

6AA

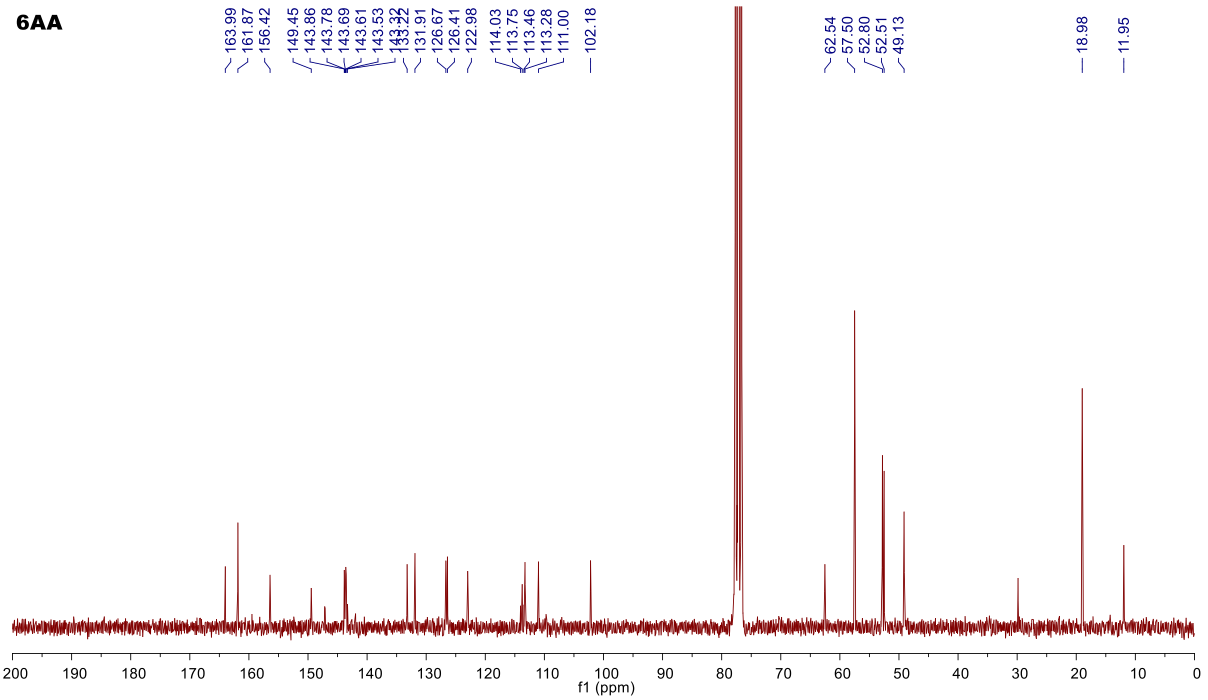

163.99
161.87
156.42
149.45
143.86
143.78
143.69
143.61
143.53
133.32
133.22
131.91
126.67
126.41
122.98
114.03
113.75
113.46
113.28
111.00
102.18
62.54
57.50
52.80
52.51
49.13
18.98
11.95
f1 (ppm)

6BB

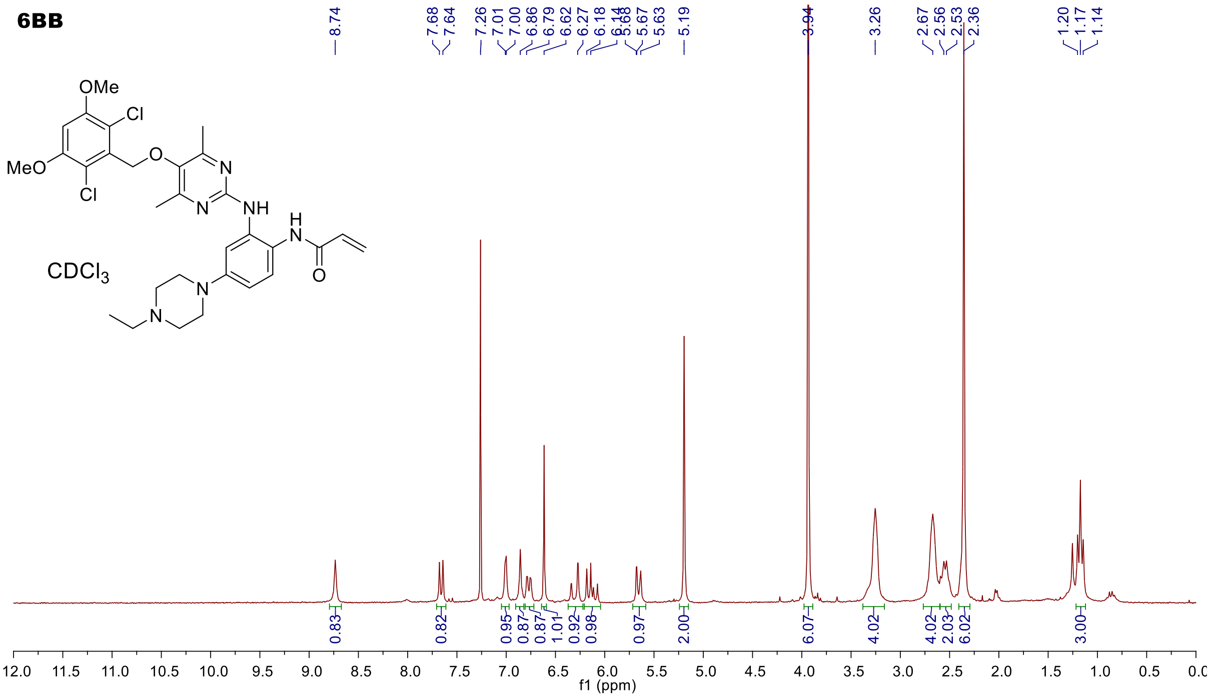
8.74
7.68
7.64
7.26
7.01
7.00
6.86
6.79
6.62
6.27
6.18
6.14
5.68
5.67
5.63
5.19
3.94
3.26
2.67
2.56
2.53
2.36
1.20
1.17
1.14
OMe
Cl
MeO
O
N
N
NH
Cl
H
N
O
N
N
CDCl$_3$
0.83
0.82
0.95
0.87
0.87
1.01
0.92
0.98
0.97
2.00
6.07
4.02
4.02
2.03
6.02
3.00
12.0 11.5 11.0 10.5 10.0 9.5 9.0 8.5 8.0 7.5 7.0 6.5 6.0 5.5 5.0 4.5 4.0 3.5 3.0 2.5 2.0 1.5 1.0 0.5 0.0
f1 (ppm)

6BB

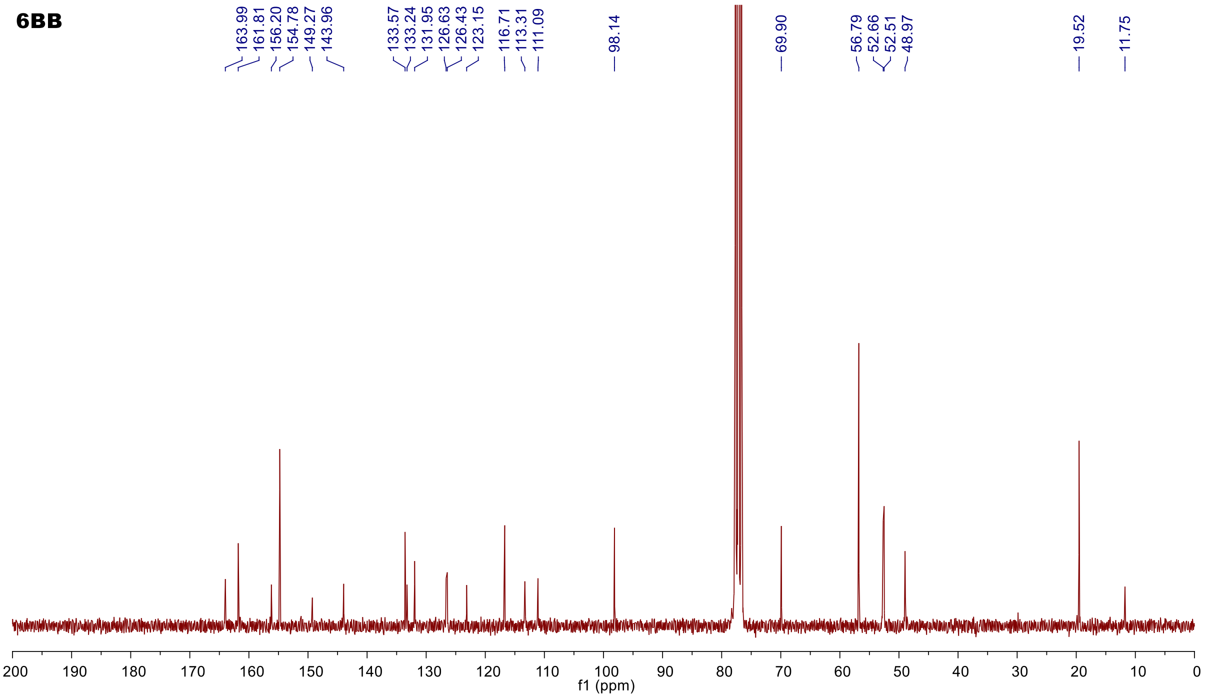
163.99
161.81
156.20
154.78
149.27
143.96
133.57
133.24
131.95
126.63
126.43
123.15
116.71
113.31
111.09
98.14
69.90
56.79
52.66
52.51
48.97
19.52
11.75
200 190 180 170 160 150 140 130 120 110 100 90 80 70 60 50 40 30 20 10 0
f1 (ppm)

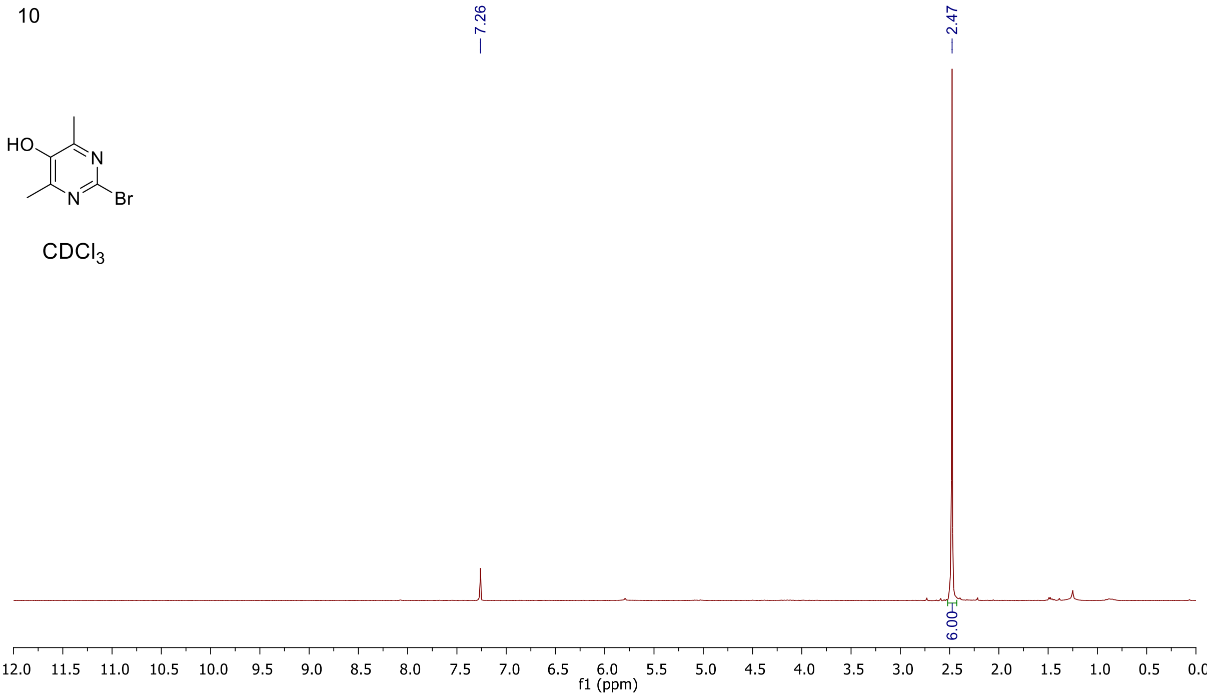
10
7.26
2.47
HO
N
N
Br
$CDCl_3$
6.00
12.0 11.5 11.0 10.5 10.0 9.5 9.0 8.5 8.0 7.5 7.0 6.5 6.0 5.5 5.0 4.5 4.0 3.5 3.0 2.5 2.0 1.5 1.0 0.5 0.0
f1 (ppm)

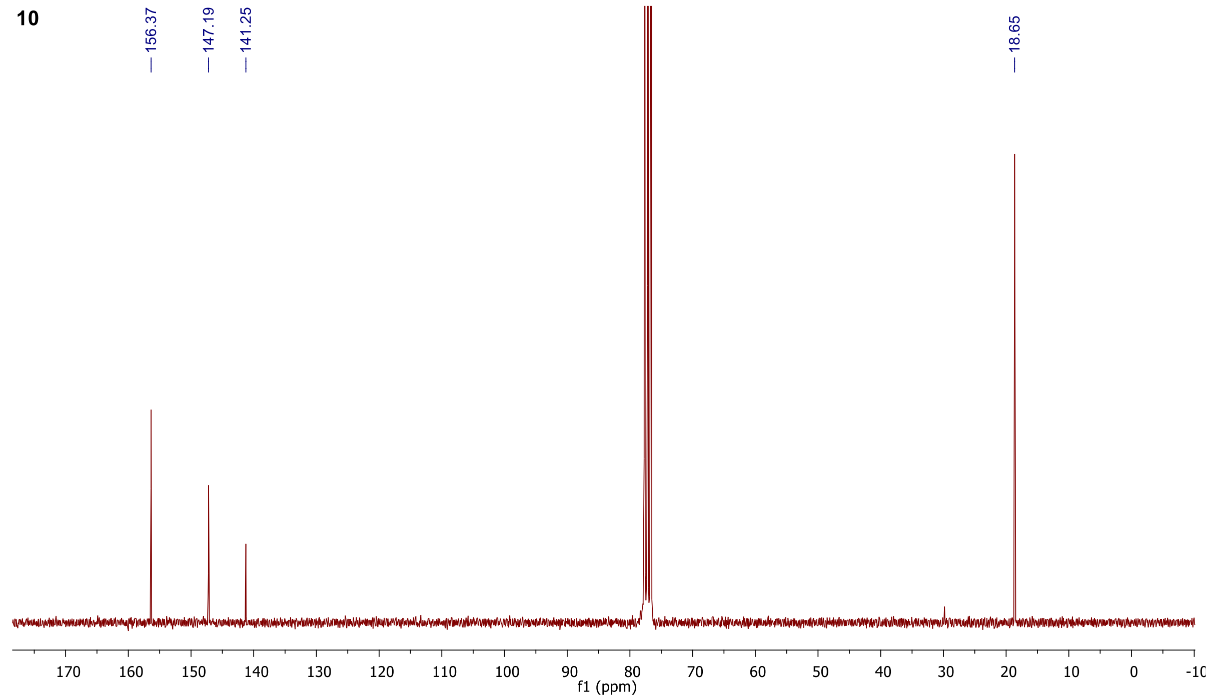
10
156.37
147.19
141.25
18.65
170 160 150 140 130 120 110 100 90 80 70 60 50 40 30 20 10 0 -10
f1 (ppm)

11

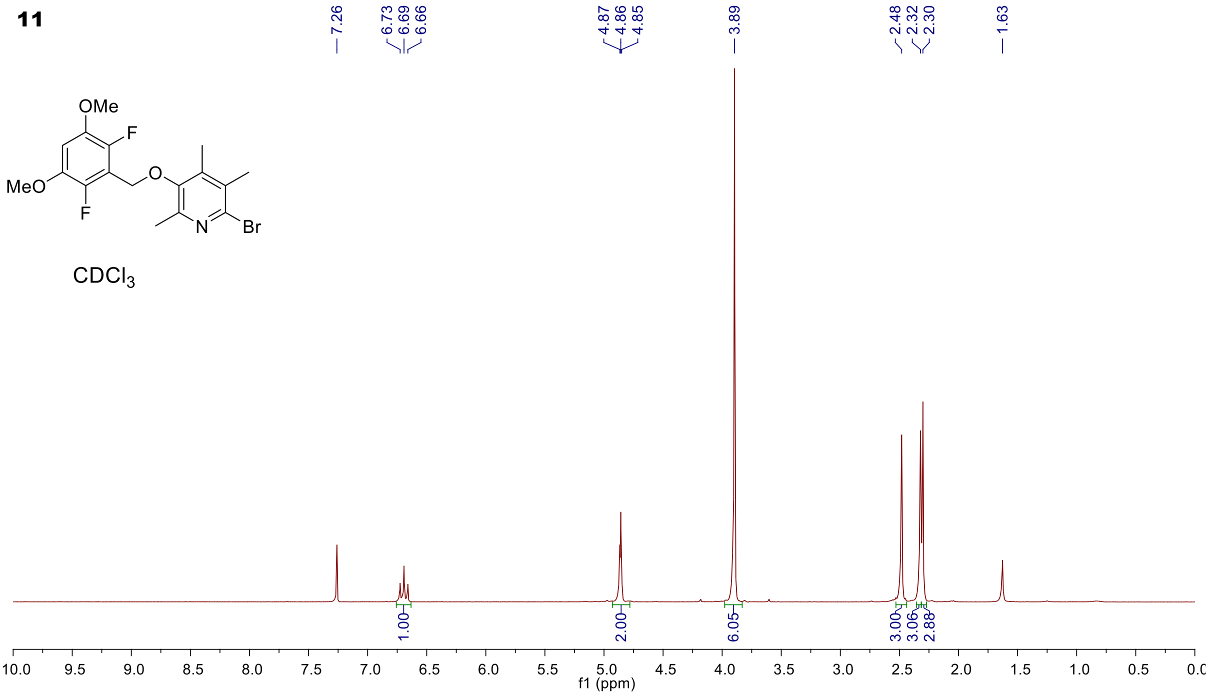
OMe
F
MeO
F
O
N
Br
7.26
6.73
6.69
6.66
4.87
4.86
4.85
3.89
2.48
2.32
2.30
1.63
1.00
2.00
6.05
3.00
3.06
2.88
f1 (ppm)

$CDCl_3$

11

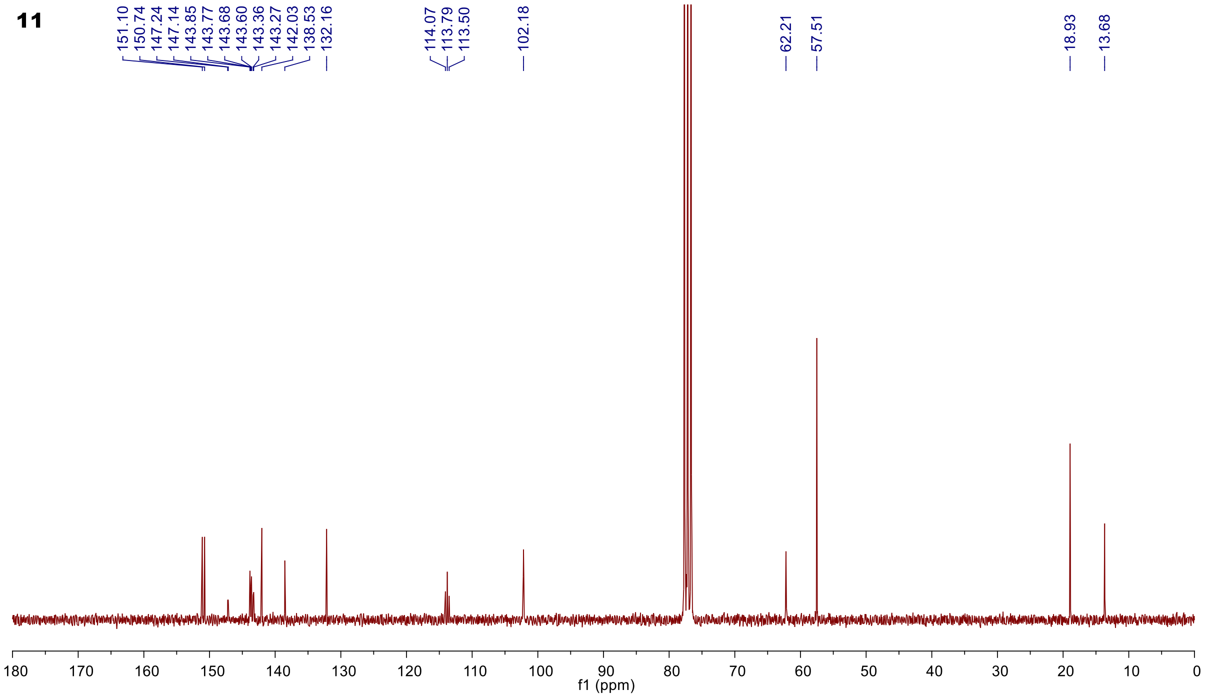
151.10
150.74
147.24
147.14
143.85
143.77
143.68
143.60
143.36
143.27
142.03
138.53
132.16
114.07
113.79
113.50
102.18
62.21
57.51
18.93
13.68
f1 (ppm)

12

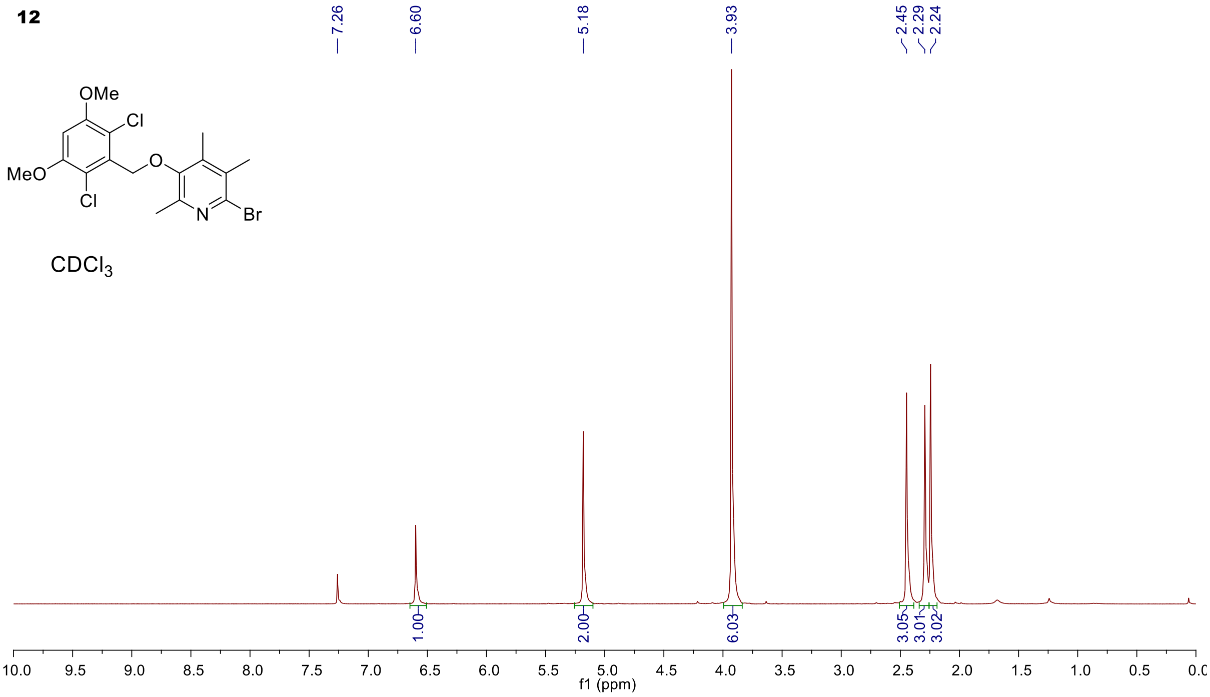
OMe
Cl
MeO
Cl
O
N
Br
CDCl3
7.26
6.60
5.18
3.93
2.45
2.29
2.24
1.00
2.00
6.03
3.05
3.01
3.02
f1 (ppm)

12

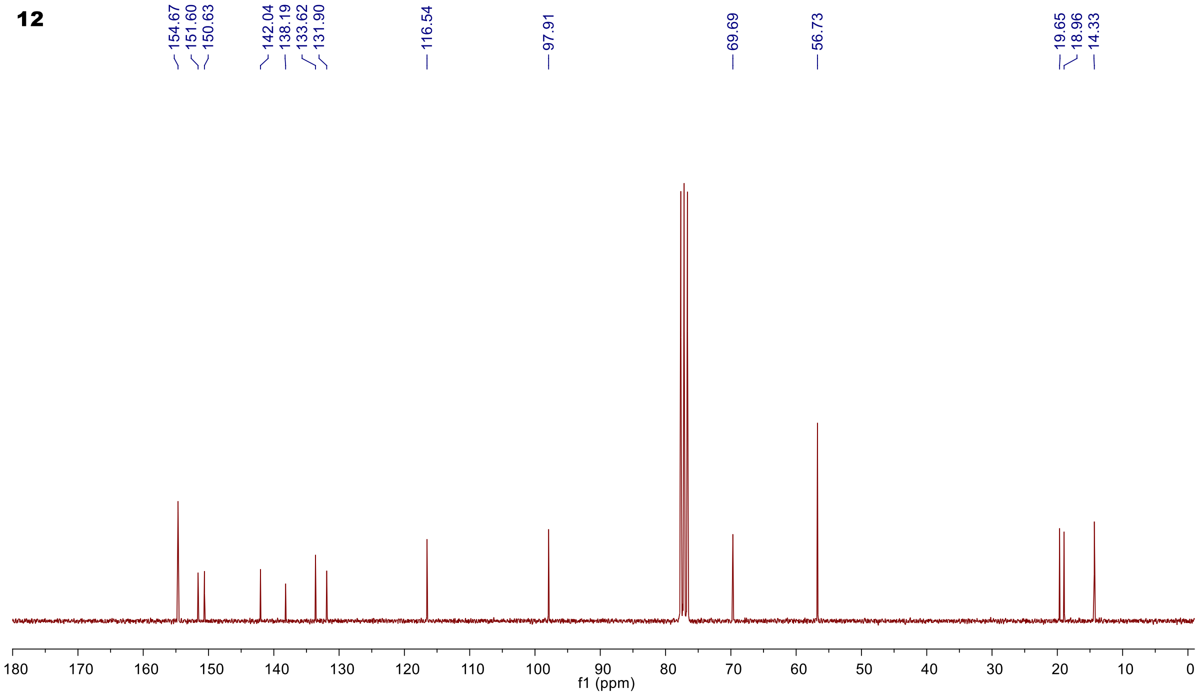
154.67
151.60
150.63
142.04
138.19
133.62
131.90
116.54
97.91
69.69
56.73
19.65
18.96
14.33
f1 (ppm)

13

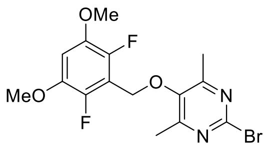
OMe
F
MeO
O
N
F
N
Br

CDCl$_3$

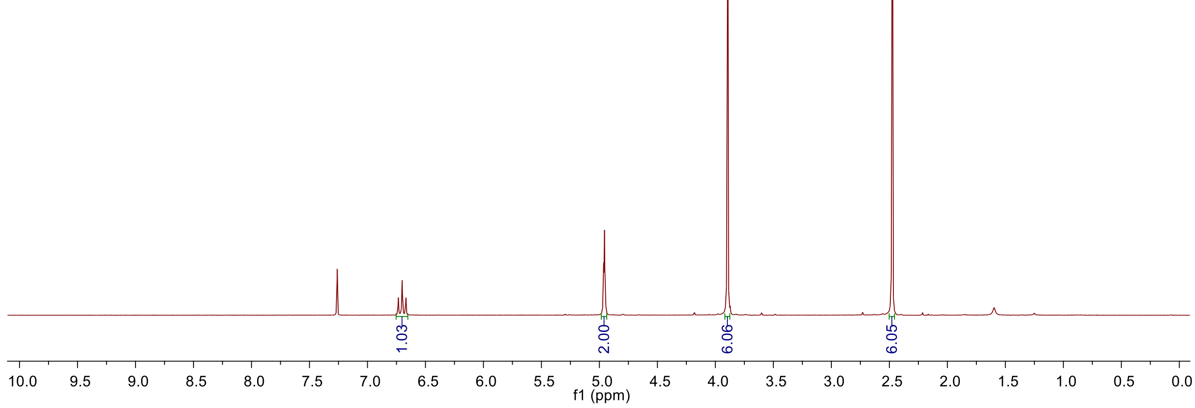
7.26
6.73
6.70
6.67
4.96
4.96
4.95
3.89
2.47
1.03
2.00
6.06
6.05
10.0 9.5 9.0 8.5 8.0 7.5 7.0 6.5 6.0 5.5 5.0 4.5 4.0 3.5 3.0 2.5 2.0 1.5 1.0 0.5 0.0
f1 (ppm)

13

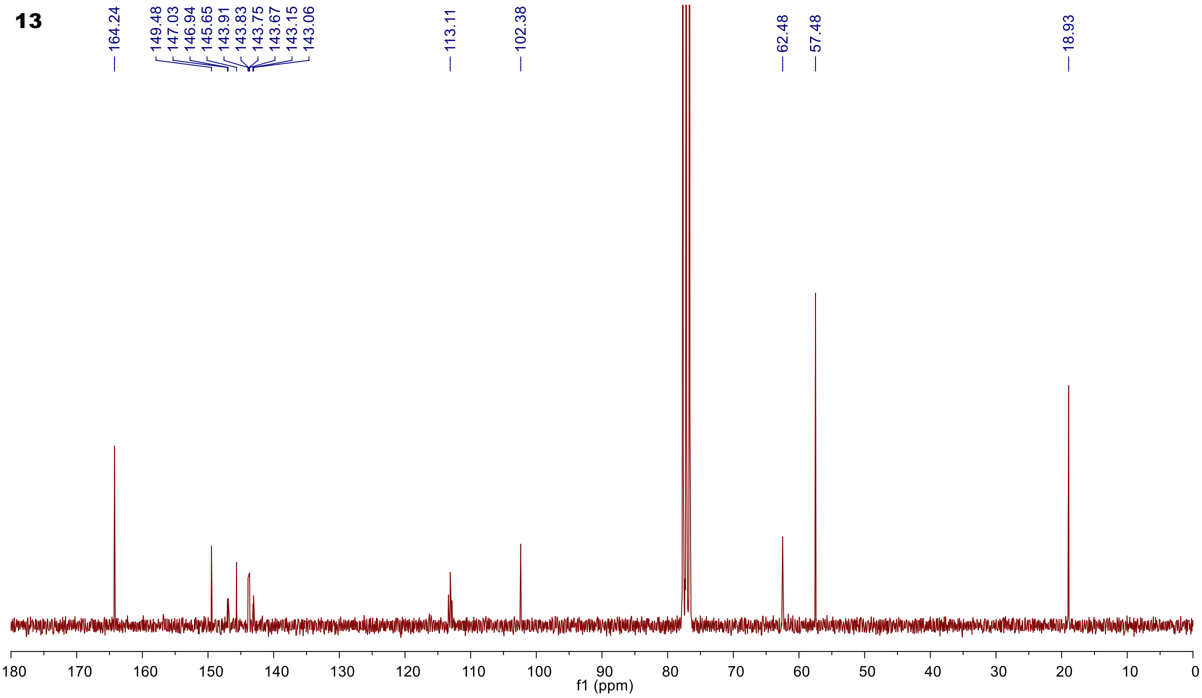
164.24
149.48
147.03
146.94
145.65
143.91
143.83
143.75
143.67
143.15
143.06
113.11
102.38
62.48
57.48
18.93
180 170 160 150 140 130 120 110 100 90 80 70 60 50 40 30 20 10 0
f1 (ppm)

14

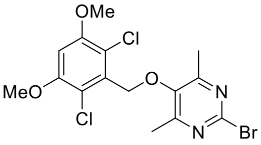

OMe
Cl
MeO
Cl
O
N
N
Br

$CDCl_3$

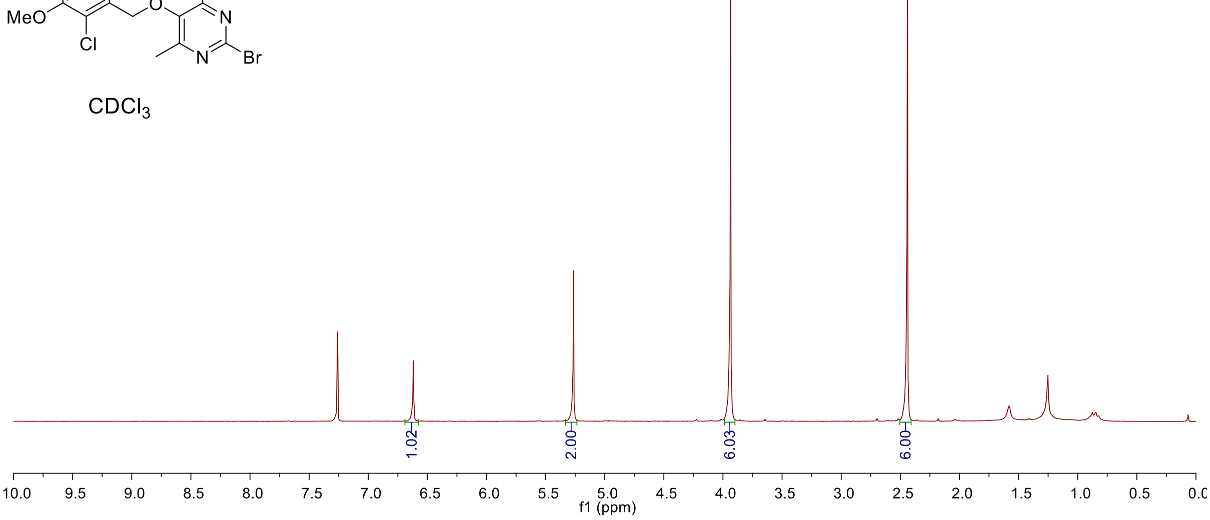

7.26
6.62
5.26
3.94
2.44
1.02
2.00
6.03
6.00
10.0
9.5
9.0
8.5
8.0
7.5
7.0
6.5
6.0
5.5
5.0
4.5
4.0
3.5
3.0
2.5
2.0
1.5
1.0
0.5
f1 (ppm)

14

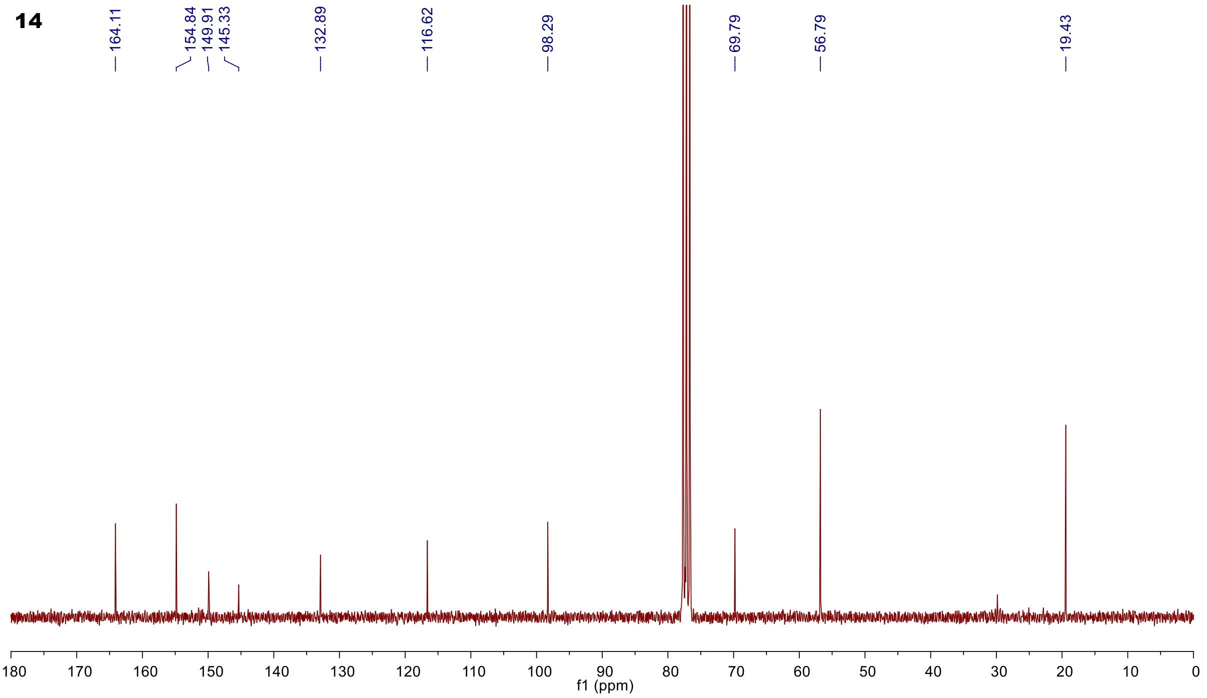

164.11
154.84
149.91
145.33
132.89
116.62
98.29
69.79
56.79
19.43
180
170
160
150
140
130
120
110
100
90
80
70
60
50
40
30
20
10
0
f1 (ppm)

22A

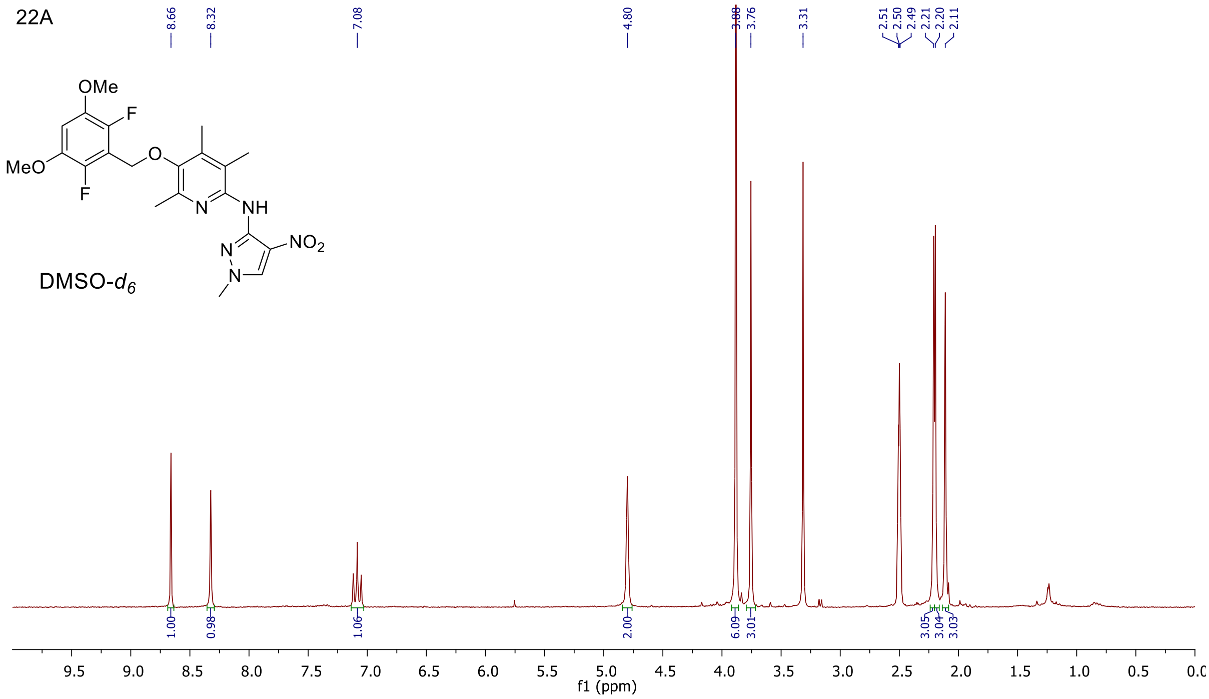

OMe
F
MeO
F
O
N
NH
N
N
NO2
DMSO-d6
8.66
8.32
7.08
4.80
3.88
3.76
3.31
2.51
2.50
2.49
2.21
2.20
2.11
1.00
0.98
1.06
2.00
6.09
3.01
3.05
3.04
3.03
f1 (ppm)

22A

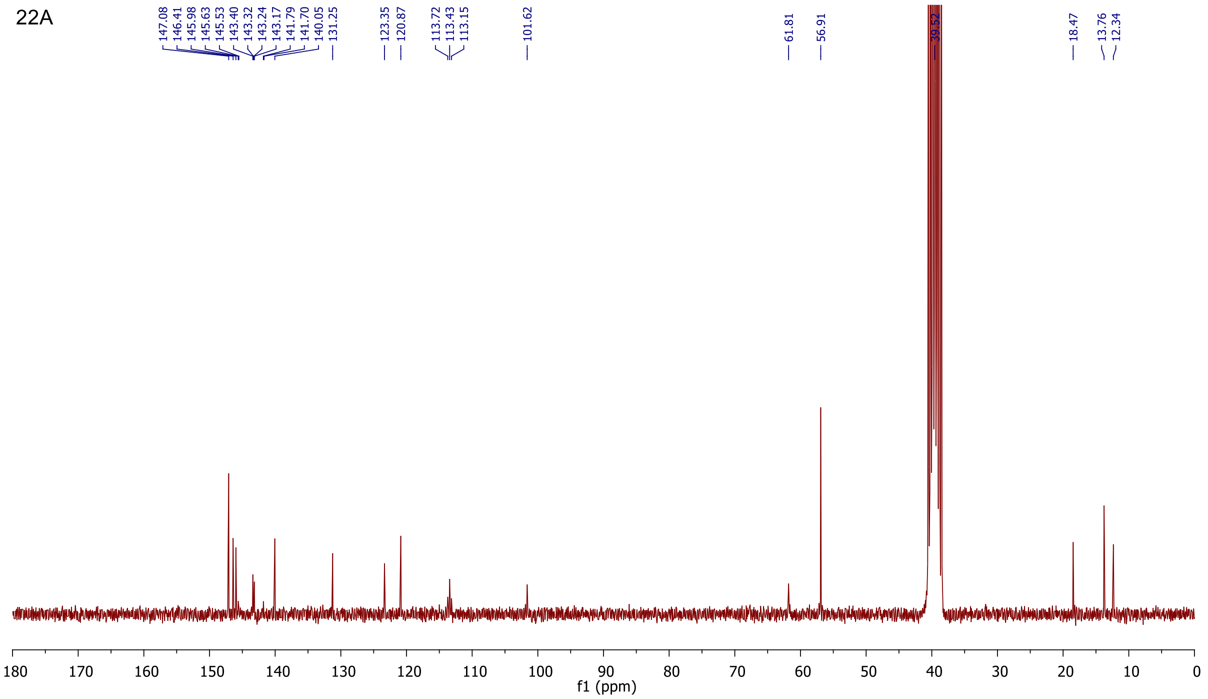

147.08
146.41
145.98
145.63
145.53
143.40
143.32
143.24
143.17
141.79
141.70
140.05
131.25
123.35
120.87
113.72
113.43
113.15
101.62
61.81
56.91
39.52
18.47
13.76
12.34
f1 (ppm)

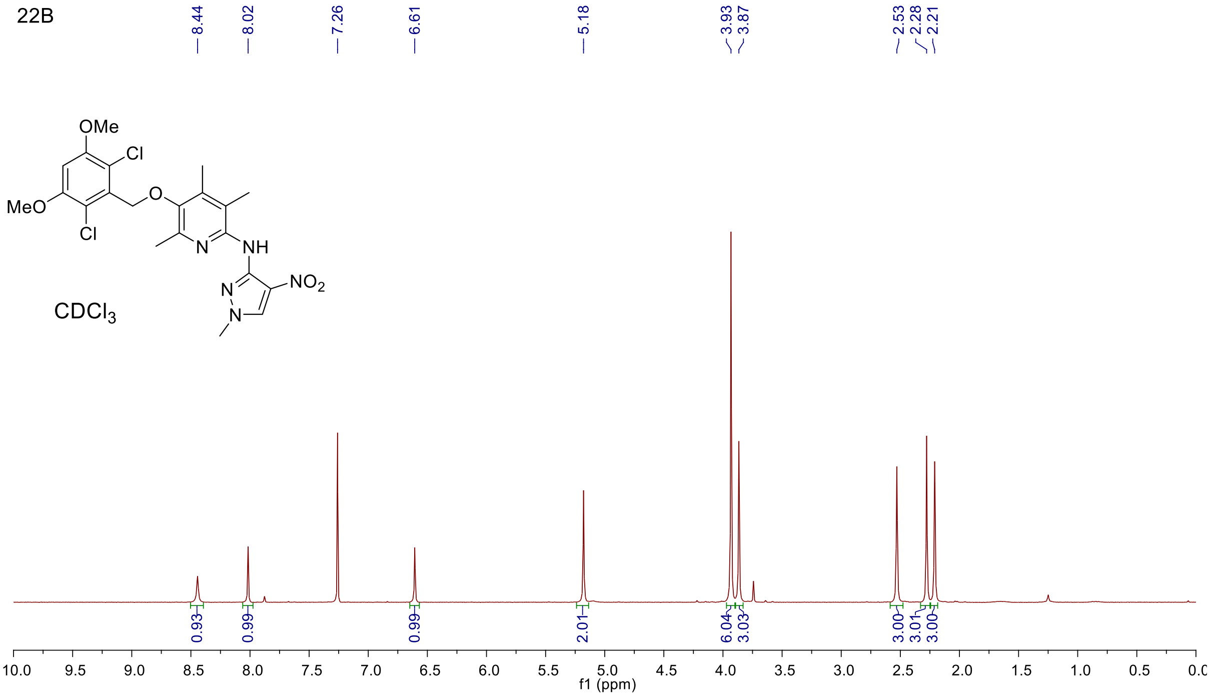
22B
8.44
8.02
7.26
6.61
5.18
3.93
3.87
2.53
2.28
2.21
OMe
Cl
MeO
O
Cl
N
NH
NO2
N
N
CDCl3
0.93
0.99
0.99
2.01
6.04
3.03
3.00
3.01
3.00
f1 (ppm)

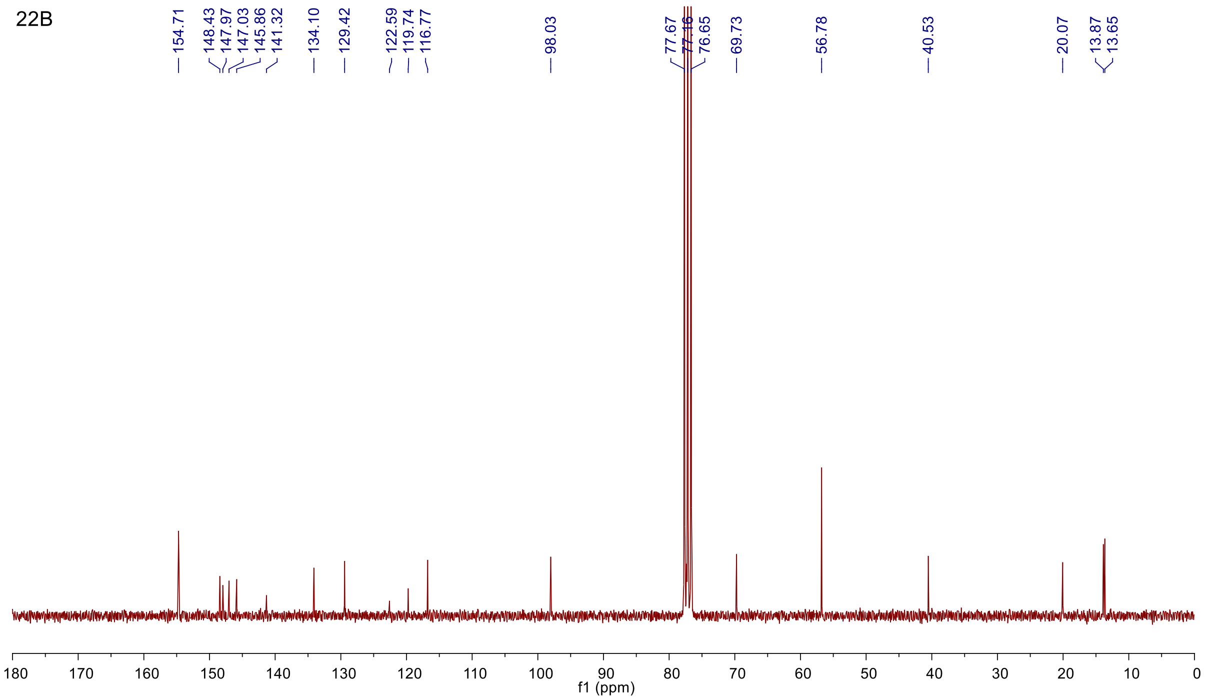
22B
154.71
148.43
147.97
147.03
145.86
141.32
134.10
129.42
122.59
119.74
116.77
98.03
77.67
77.16
76.65
69.73
56.78
40.53
20.07
13.87
13.65
f1 (ppm)

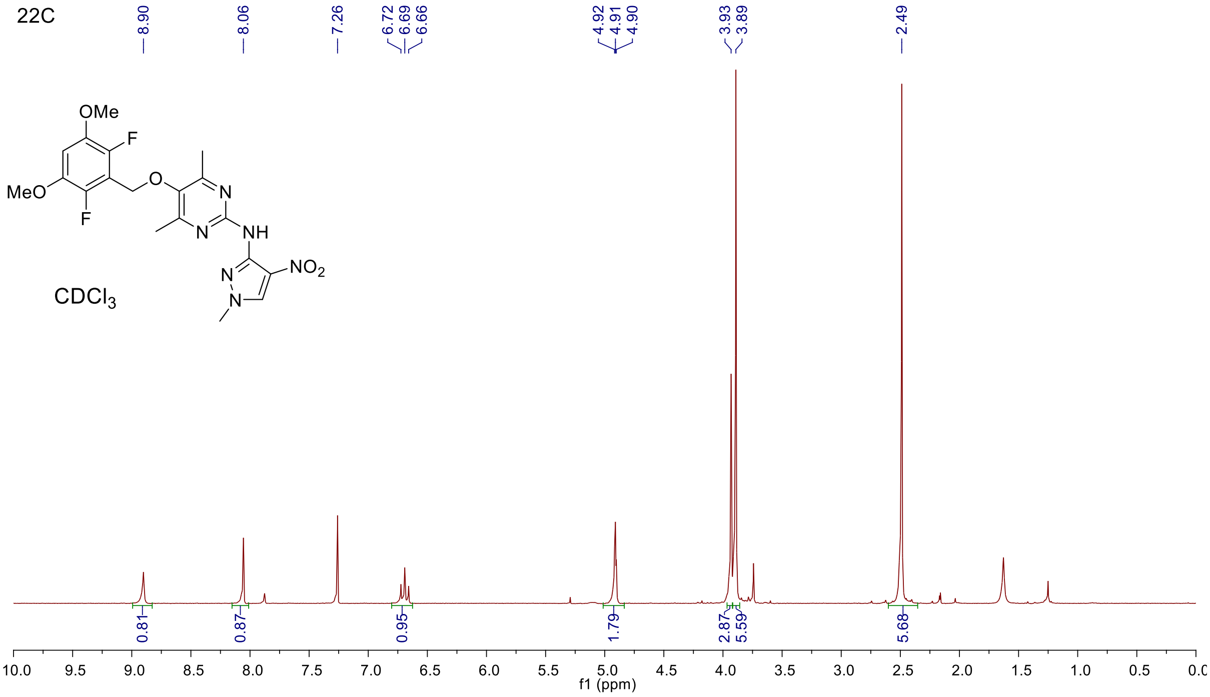
22C
8.90
8.06
7.26
6.72
6.69
6.66
4.92
4.91
4.90
3.93
3.89
2.49
OMe
F
MeO
F
O
N
N
NH
N
N
NO2
CDCl3
0.81
0.87
0.95
1.79
2.87
5.59
5.68
10.0 9.5 9.0 8.5 8.0 7.5 7.0 6.5 6.0 5.5 5.0 4.5 4.0 3.5 3.0 2.5 2.0 1.5 1.0 0.5 0.0
f1 (ppm)

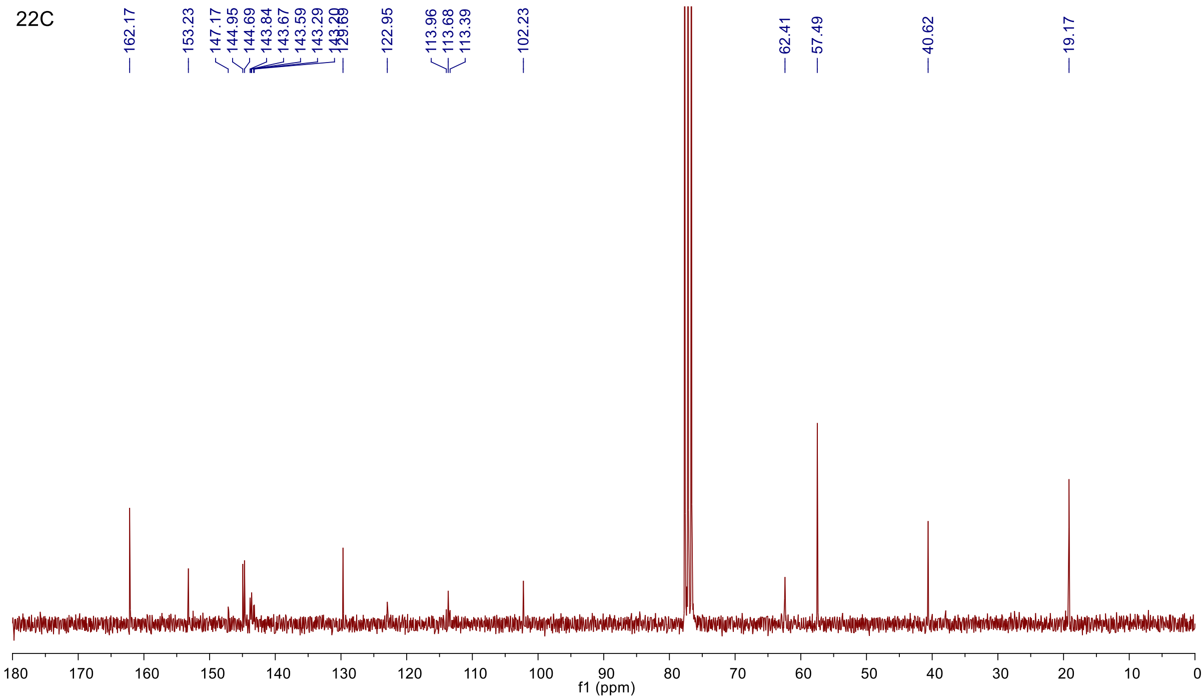
22C
162.17
153.23
147.17
144.95
144.69
143.84
143.67
143.59
143.29
122.95
113.96
113.68
113.39
102.23
62.41
57.49
40.62
19.17
180 170 160 150 140 130 120 110 100 90 80 70 60 50 40 30 20 10 0
f1 (ppm)

22D

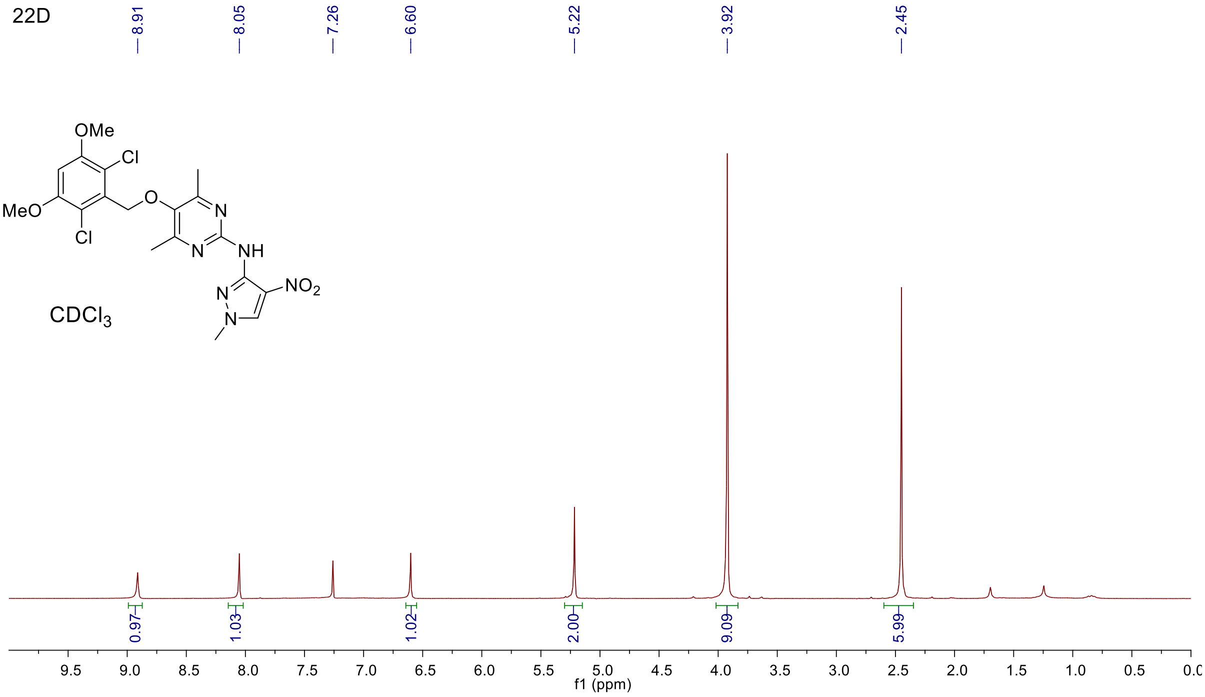
8.91
8.05
7.26
6.60
5.22
3.92
2.45
OMe
Cl
MeO
O
N
N
NH
Cl
NO2
N
N
CDCl3
0.97
1.03
1.02
2.00
9.09
5.99
9.5
9.0
8.5
8.0
7.5
7.0
6.5
6.0
5.5
5.0
4.5
4.0
3.5
3.0
2.5
2.0
1.5
1.0
0.5
f1 (ppm)

22D

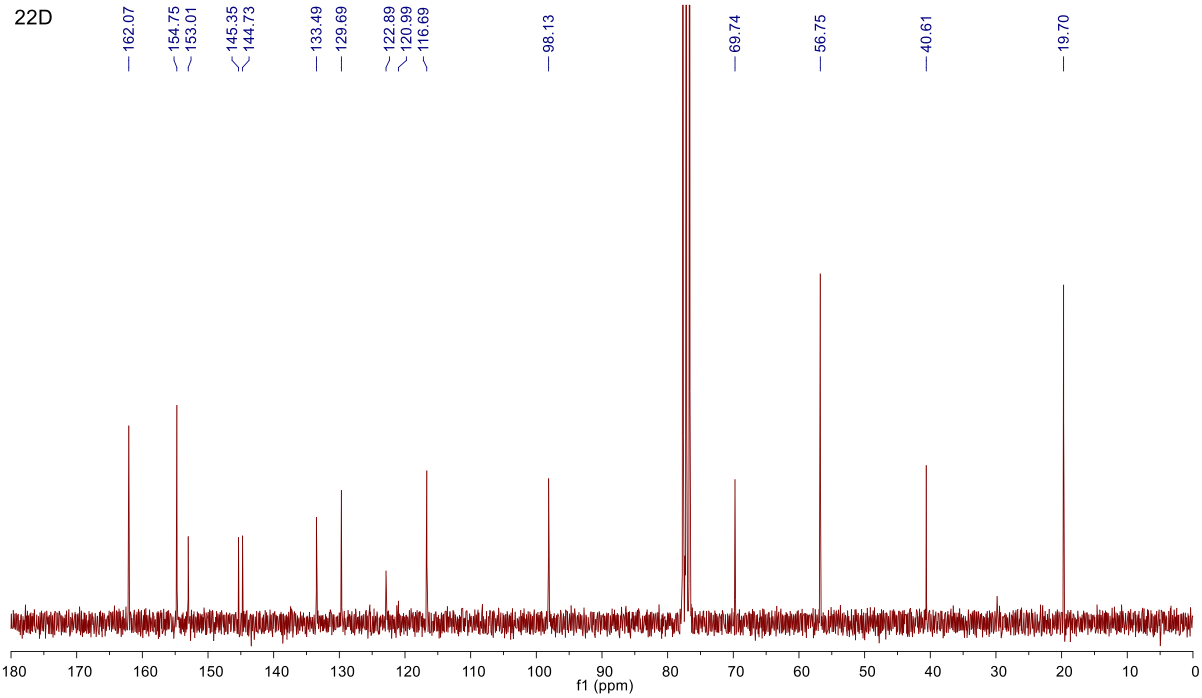
162.07
154.75
153.01
145.35
144.73
133.49
129.69
122.89
120.99
116.69
98.13
69.74
56.75
40.61
19.70
180
170
160
150
140
130
120
110
100
90
80
70
60
50
40
30
20
10
0
f1 (ppm)

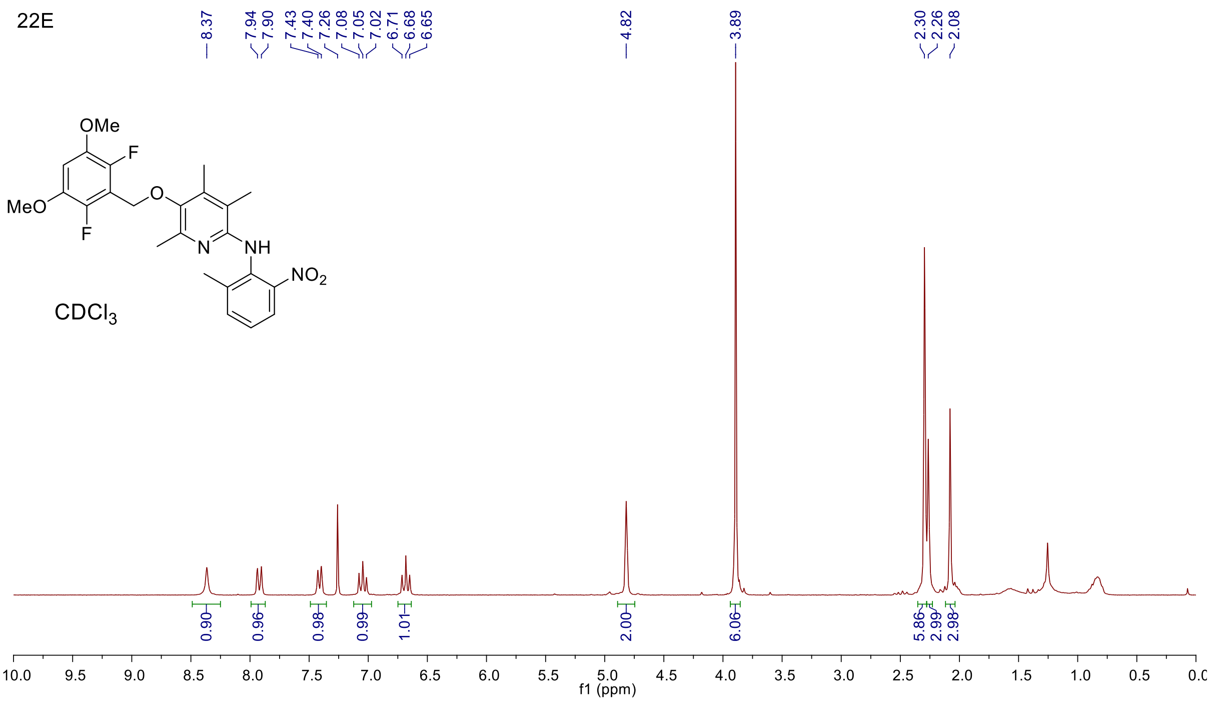
22E
8.37
7.94
7.90
7.43
7.40
7.26
7.08
7.05
7.02
6.71
6.68
6.65
4.82
3.89
2.30
2.26
2.08
OMe
F
MeO
F
O
N
NH
NO2
CDCl3
0.90
0.96
0.98
0.99
1.01
2.00
6.06
5.86
2.99
2.98
f1 (ppm)

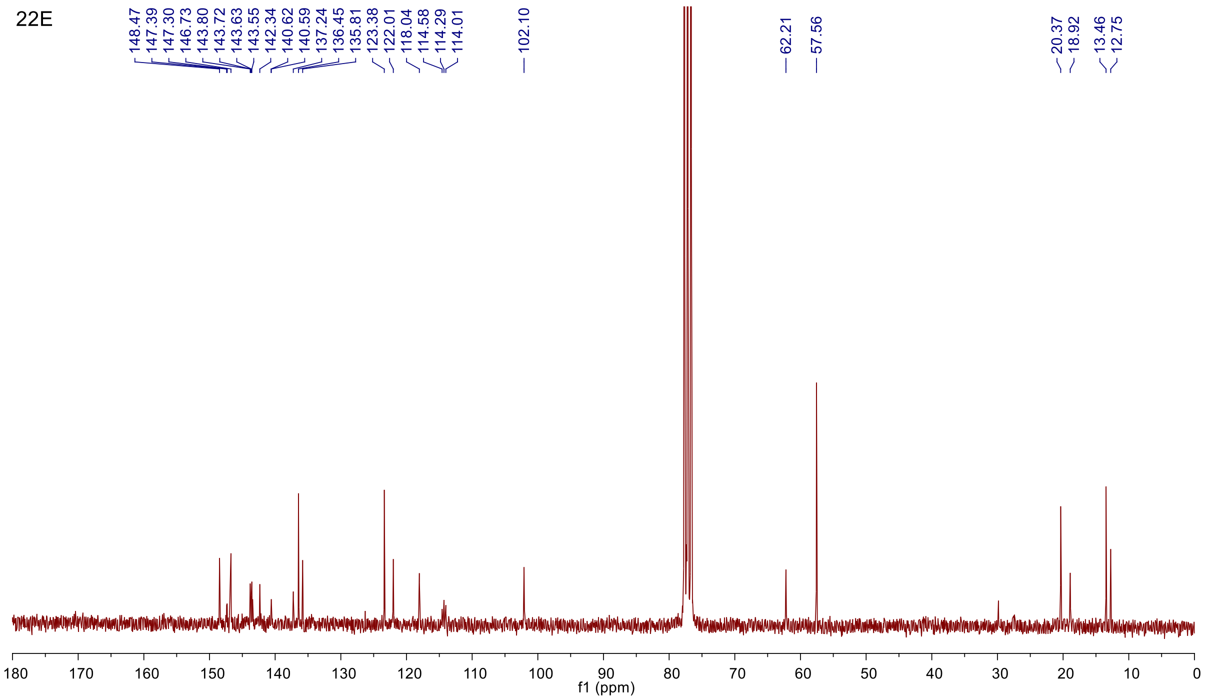
22E
148.47
147.39
147.30
146.73
143.80
143.72
143.63
143.55
142.34
140.62
140.59
137.24
136.45
135.81
123.38
122.01
118.04
114.58
114.29
114.01
102.10
62.21
57.56
20.37
18.92
13.46
12.75
f1 (ppm)

22F

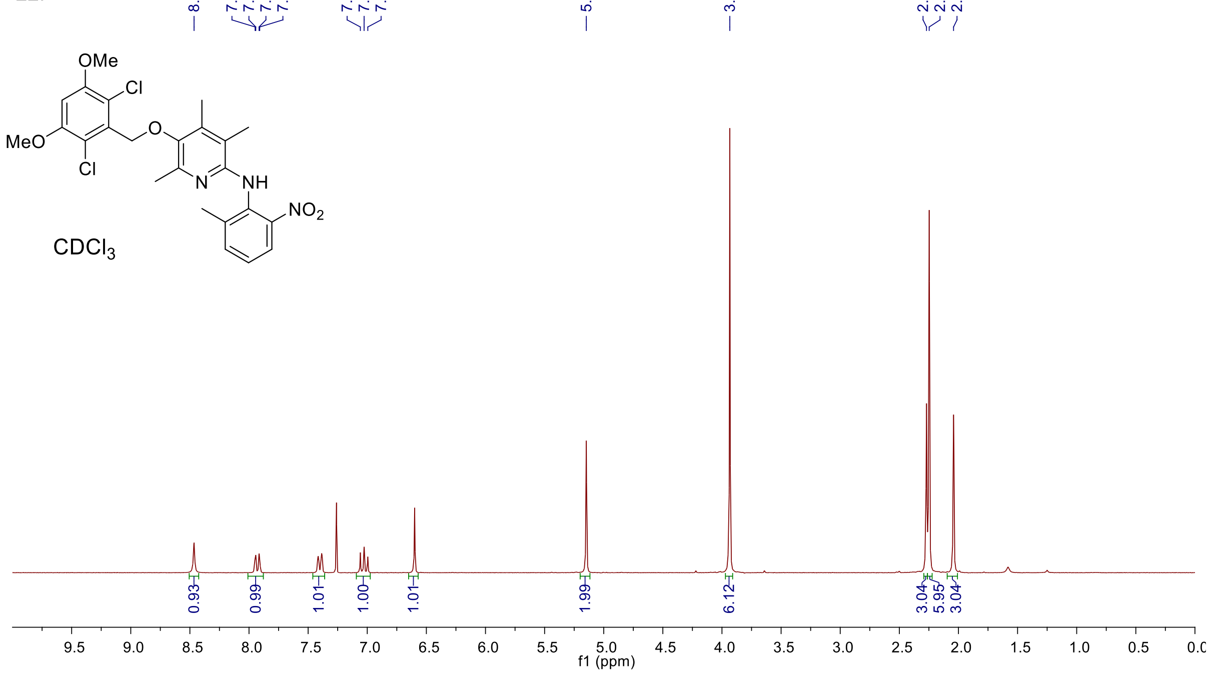
8.46
7.95
7.94
7.92
7.91
7.06
7.03
7.00
5.15
3.93
2.27
2.25
2.04
OMe
Cl
MeO
O
Cl
N
NH
NO2
CDCl3
0.93
0.99
1.01
1.00
1.01
1.99
6.12
3.04
5.95
3.04
9.5
9.0
8.5
8.0
7.5
7.0
6.5
6.0
5.5
5.0
4.5
4.0
3.5
3.0
2.5
2.0
1.5
1.0
0.5
0.0
f1 (ppm)

22F

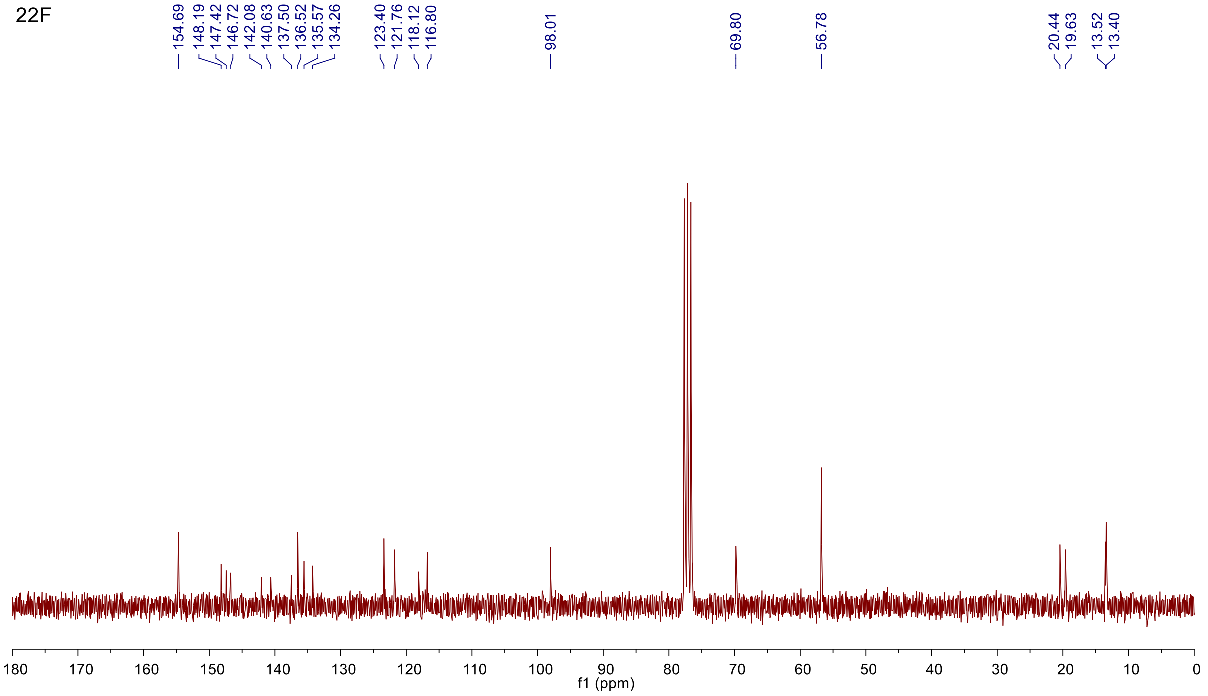
154.69
148.19
147.42
146.72
142.08
140.63
137.50
136.52
135.57
134.26
123.40
121.76
118.12
116.80
98.01
69.80
56.78
20.44
19.63
13.52
13.40
180
170
160
150
140
130
120
110
100
90
80
70
60
50
40
30
20
10
0
f1 (ppm)

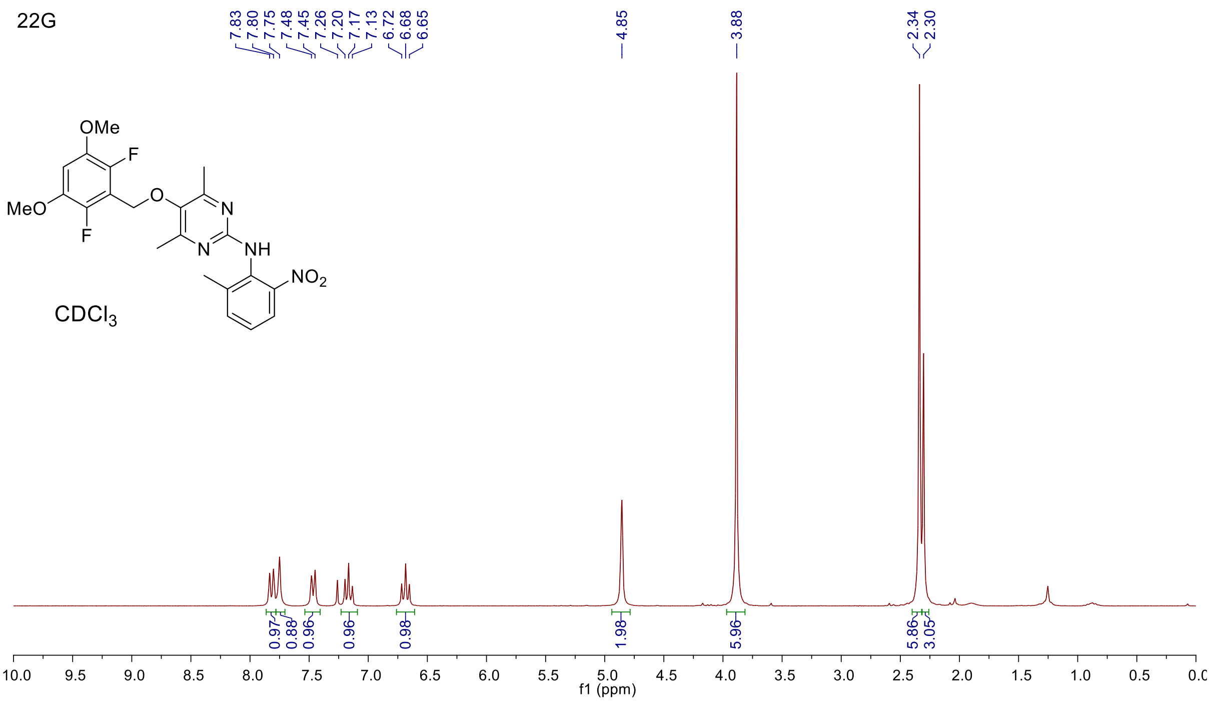
22G
7.83
7.80
7.75
7.48
7.45
7.26
7.20
7.17
7.13
6.72
6.68
6.65
4.85
3.88
2.34
2.30
OMe
F
MeO
O
N
N
NH
F
NO2
CDCl3
0.97
0.88
0.96
0.96
0.98
1.98
5.96
5.86
3.05
f1 (ppm)

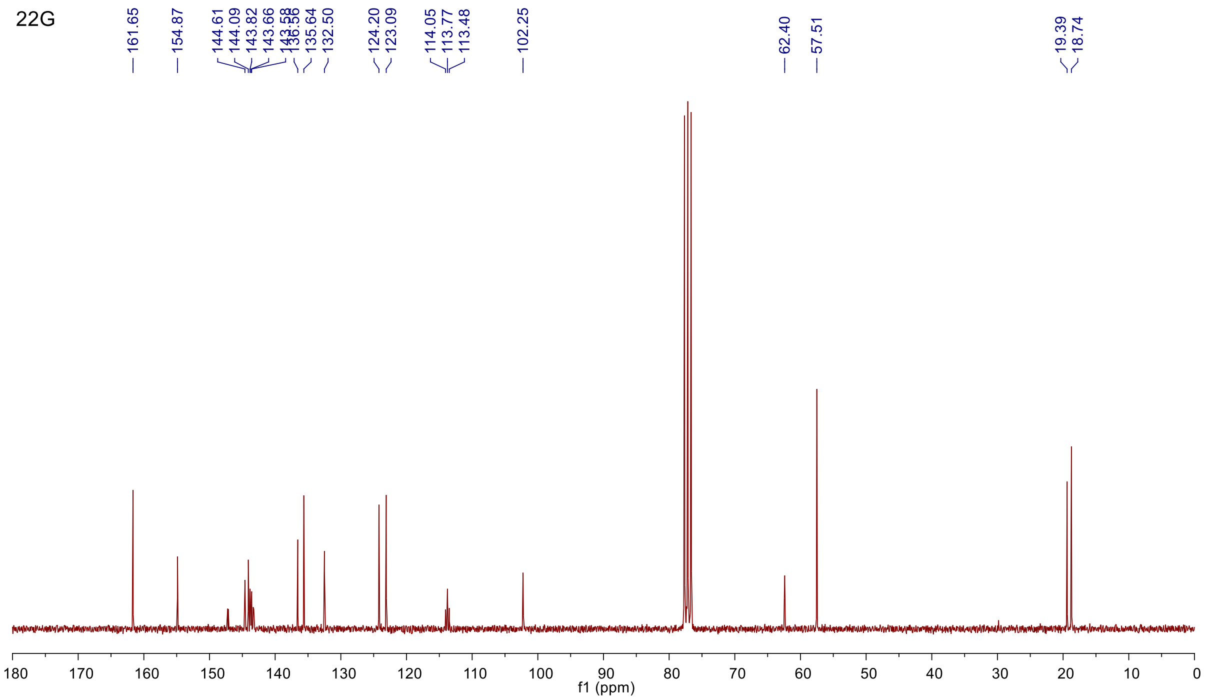
22G
161.65
154.87
144.61
144.09
143.82
143.66
143.58
136.56
135.64
132.50
124.20
123.09
114.05
113.77
113.48
102.25
62.40
57.51
19.39
18.74
f1 (ppm)

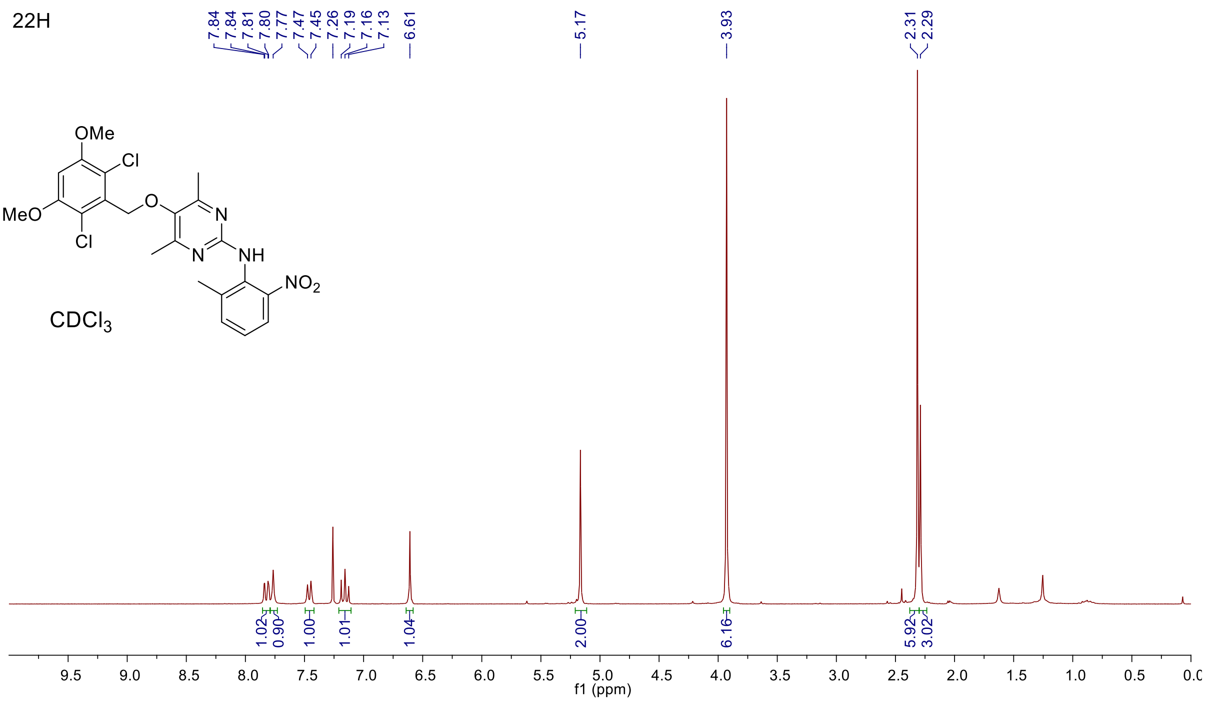
22H
7.84
7.84
7.81
7.80
7.77
7.47
7.45
7.26
7.19
7.16
7.13
6.61
5.17
3.93
2.31
2.29
OMe
Cl
MeO
Cl
O
N
N
NH
NO2
CDCl3
1.02
0.90
1.00
1.01
1.04
2.00
6.16
5.92
3.02
f1 (ppm)

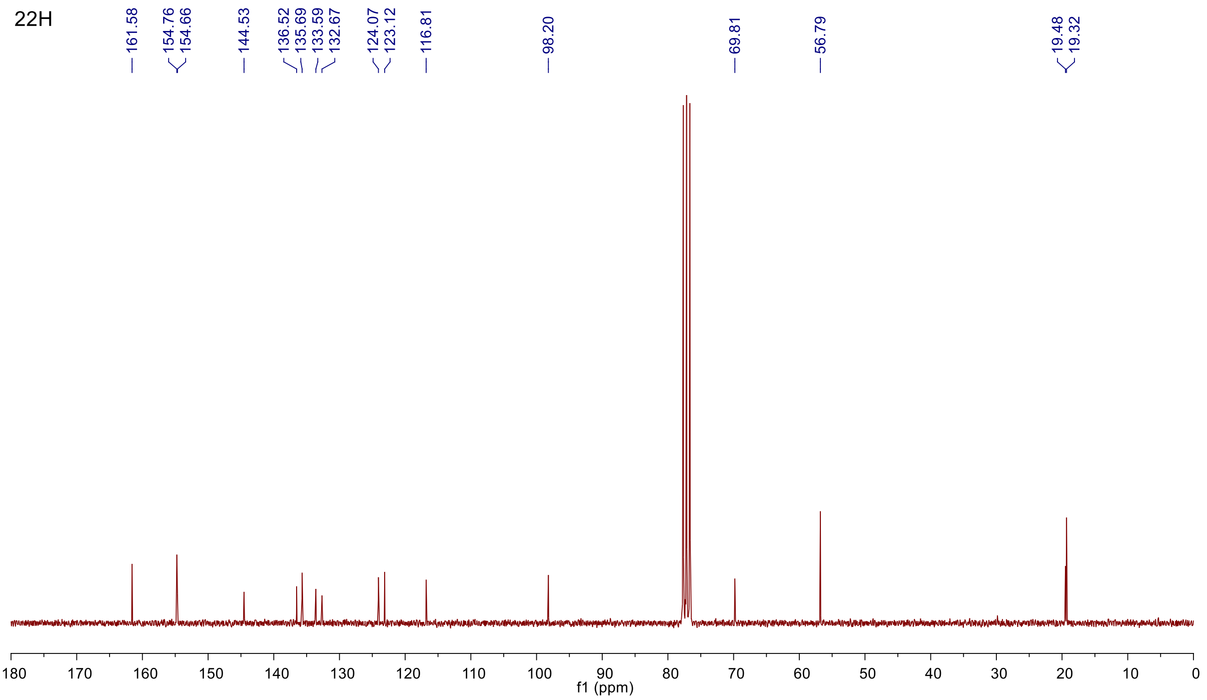
22H
161.58
154.76
154.66
144.53
136.52
135.69
133.59
132.67
124.07
123.12
116.81
98.20
69.81
56.79
19.48
19.32
f1 (ppm)

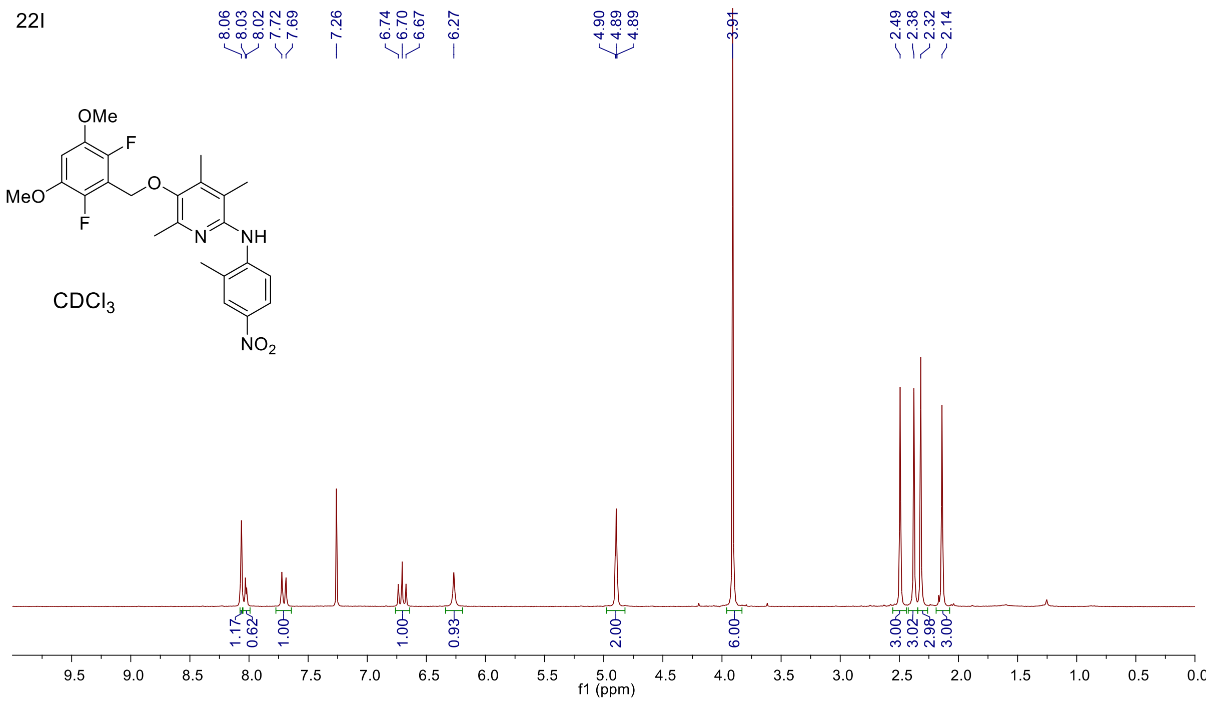

22l
8.06
8.03
8.02
7.72
7.69
7.26
6.74
6.70
6.67
6.27
4.90
4.89
4.89
3.91
2.49
2.38
2.32
2.14
OMe
F
MeO
O
F
N
NH
NO2
CDCl3
1.17
0.62
1.00
1.00
0.93
2.00
6.00
3.00
3.02
2.98
3.00
f1 (ppm)

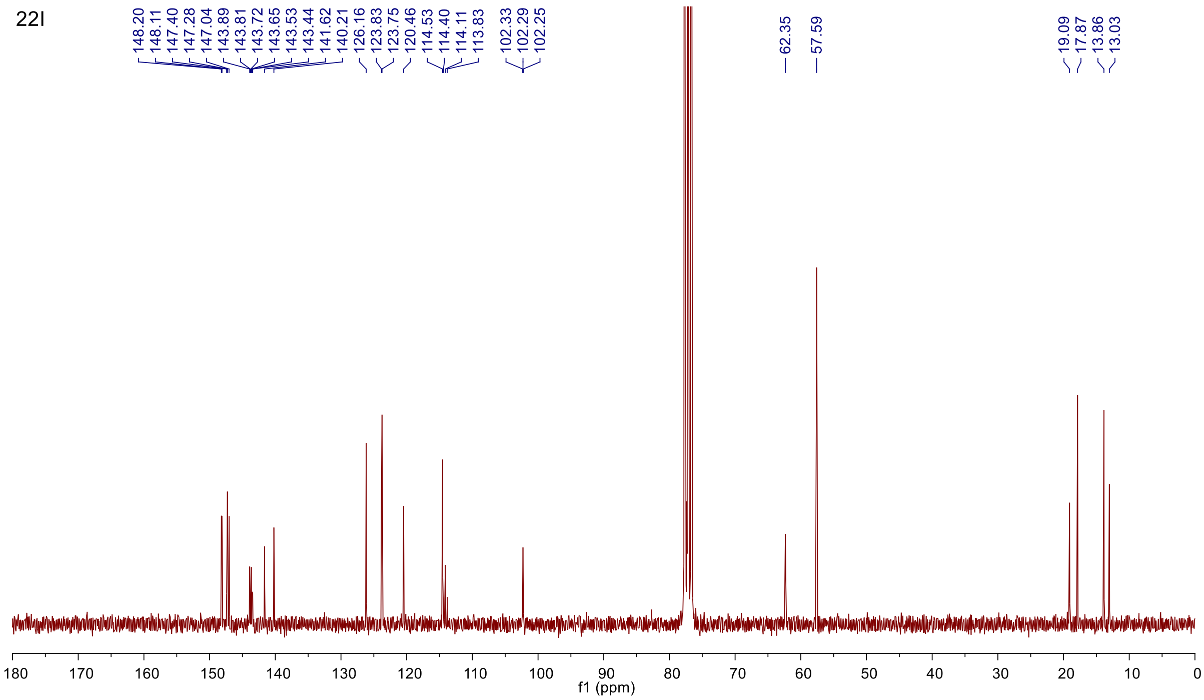

22l
148.20
148.11
147.40
147.28
147.04
143.89
143.81
143.72
143.65
143.53
143.44
141.62
140.21
126.16
123.83
123.75
120.46
114.53
114.40
114.11
113.83
102.33
102.29
102.25
62.35
57.59
19.09
17.87
13.86
13.03
f1 (ppm)

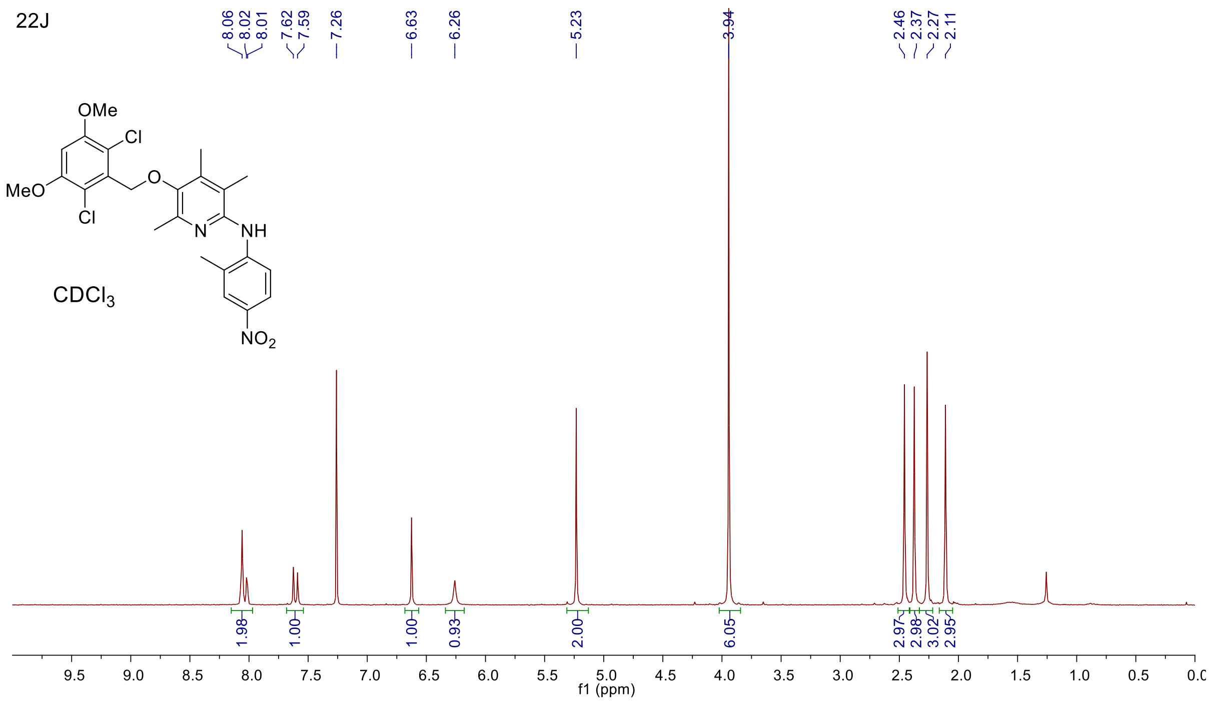
22J
8.06
8.02
8.01
7.62
7.59
7.26
6.63
6.26
5.23
3.94
2.46
2.37
2.27
2.11
OMe
Cl
MeO
O
Cl
N
NH
CDCl3
NO2
1.98
1.00
1.00
0.93
2.00
6.05
2.97
2.98
3.02
2.95
f1 (ppm)

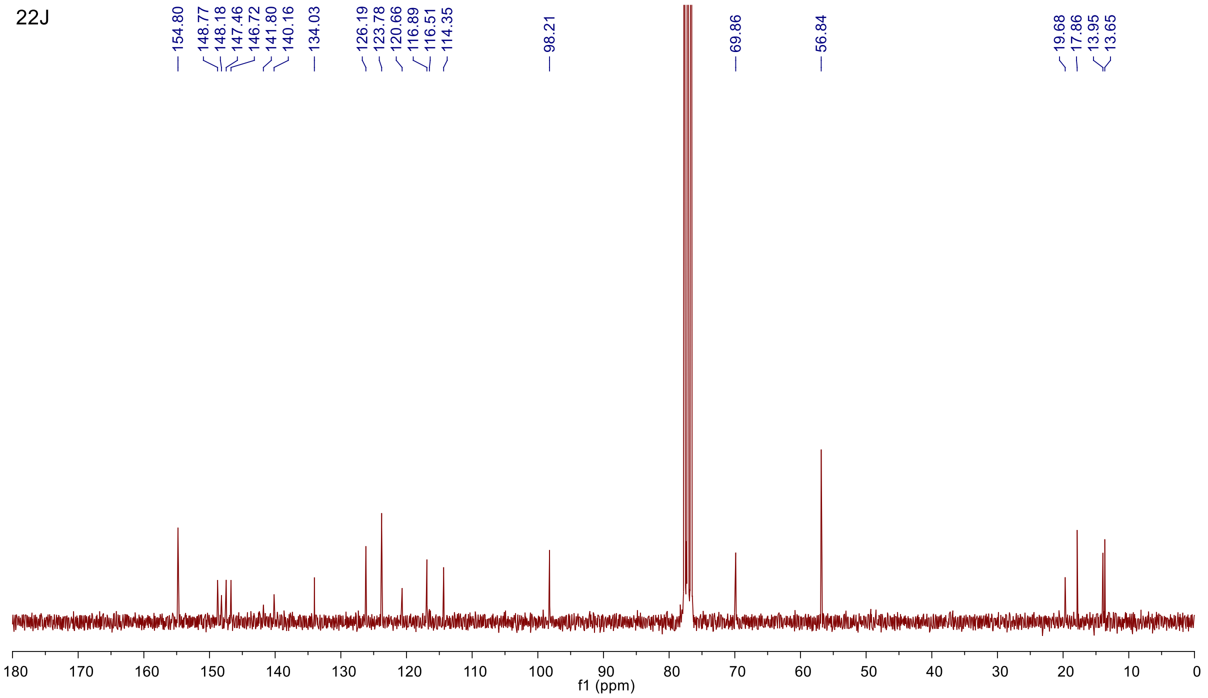
22J
154.80
148.77
148.18
147.46
146.72
141.80
140.16
134.03
126.19
123.78
120.66
116.89
116.51
114.35
98.21
69.86
56.84
19.68
17.86
13.95
13.65
f1 (ppm)

22K

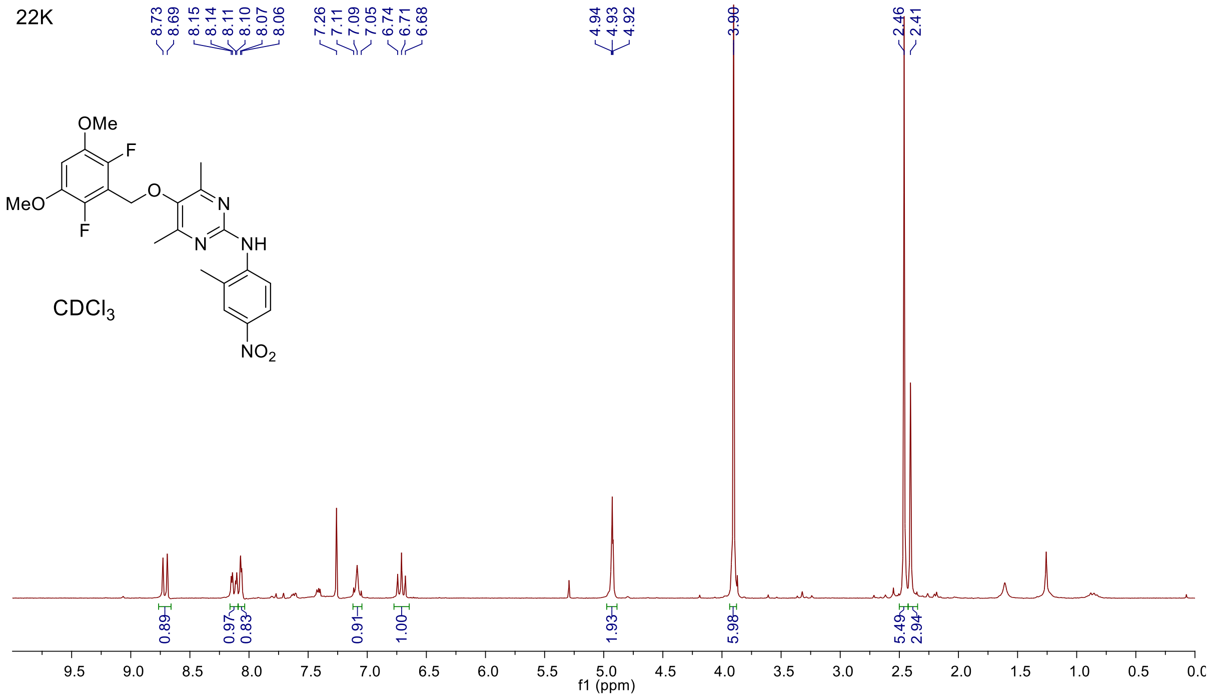

8.73
8.69
8.15
8.14
8.11
8.10
8.07
8.06
7.26
7.11
7.09
7.05
6.74
6.71
6.68
4.94
4.93
4.92
3.90
2.46
2.41
OMe
F
MeO
O
N
N
NH
F
NO2
CDCl3
0.89
0.97
0.83
0.91
1.00
1.93
5.98
5.49
2.94
f1 (ppm)

22K

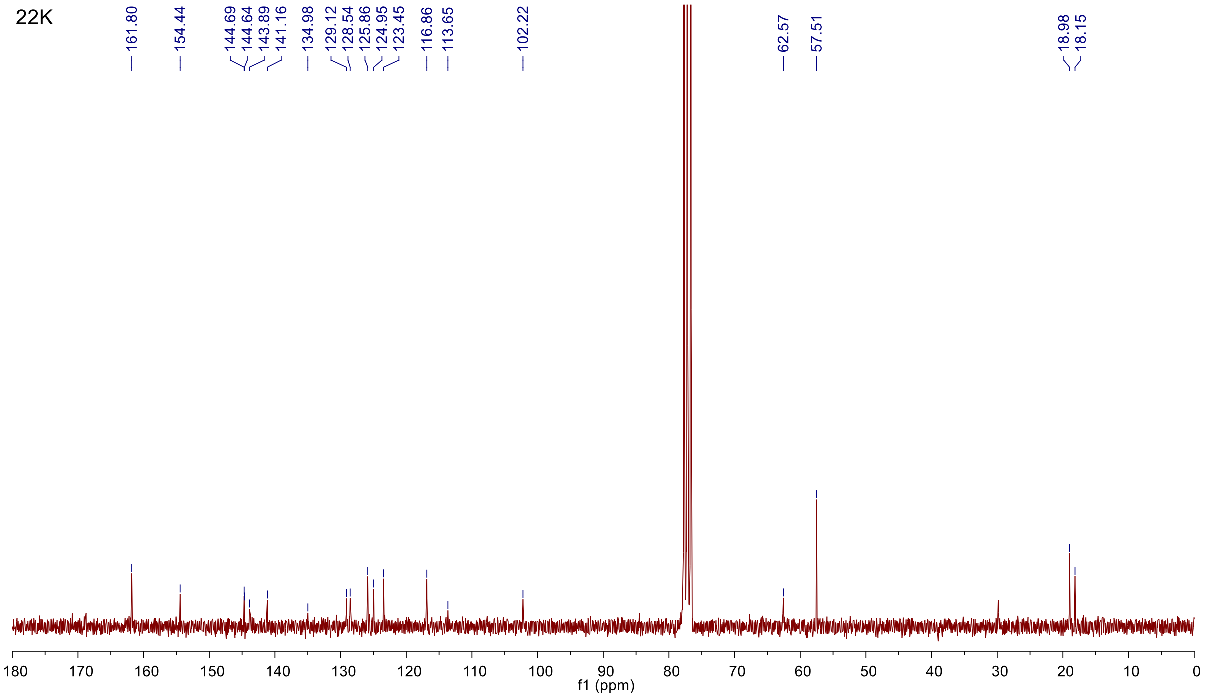

161.80
154.44
144.69
144.64
143.89
141.16
134.98
129.12
128.54
125.86
124.95
123.45
116.86
113.65
102.22
62.57
57.51
18.98
18.15
f1 (ppm)

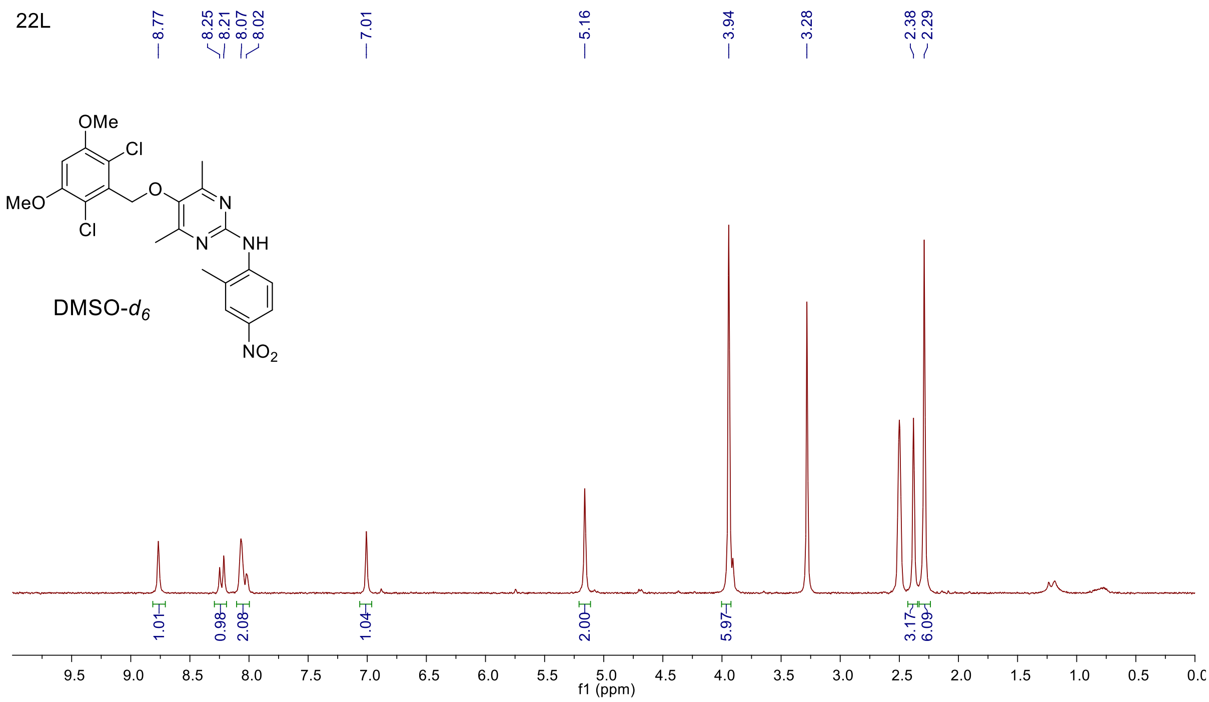
22L
8.77
8.25
8.21
8.07
8.02
7.01
5.16
3.94
3.28
2.38
2.29
OMe
Cl
MeO
O
N
N
NH
Cl
NO2
DMSO-d6
1.01
0.98
2.08
1.04
2.00
5.97
3.17
6.09
f1 (ppm)

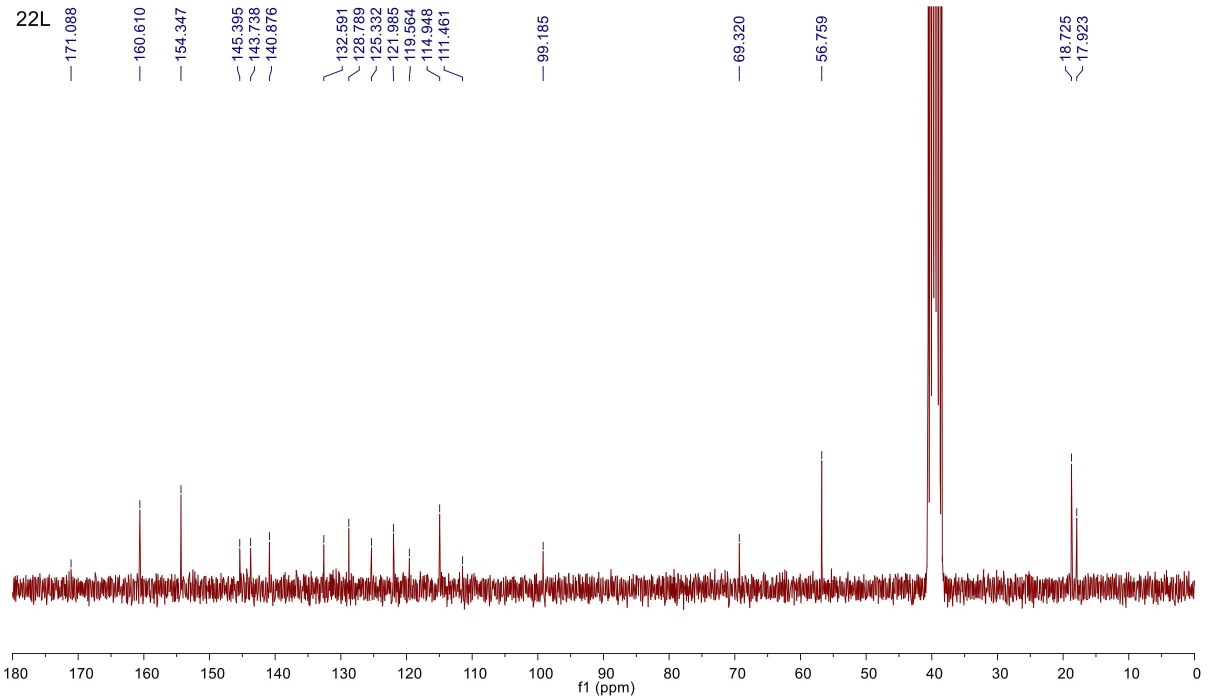
22L
171.088
160.610
154.347
145.395
143.738
140.876
132.591
128.789
125.332
121.985
119.564
114.948
111.461
99.185
69.320
56.759
18.725
17.923
f1 (ppm)

22M

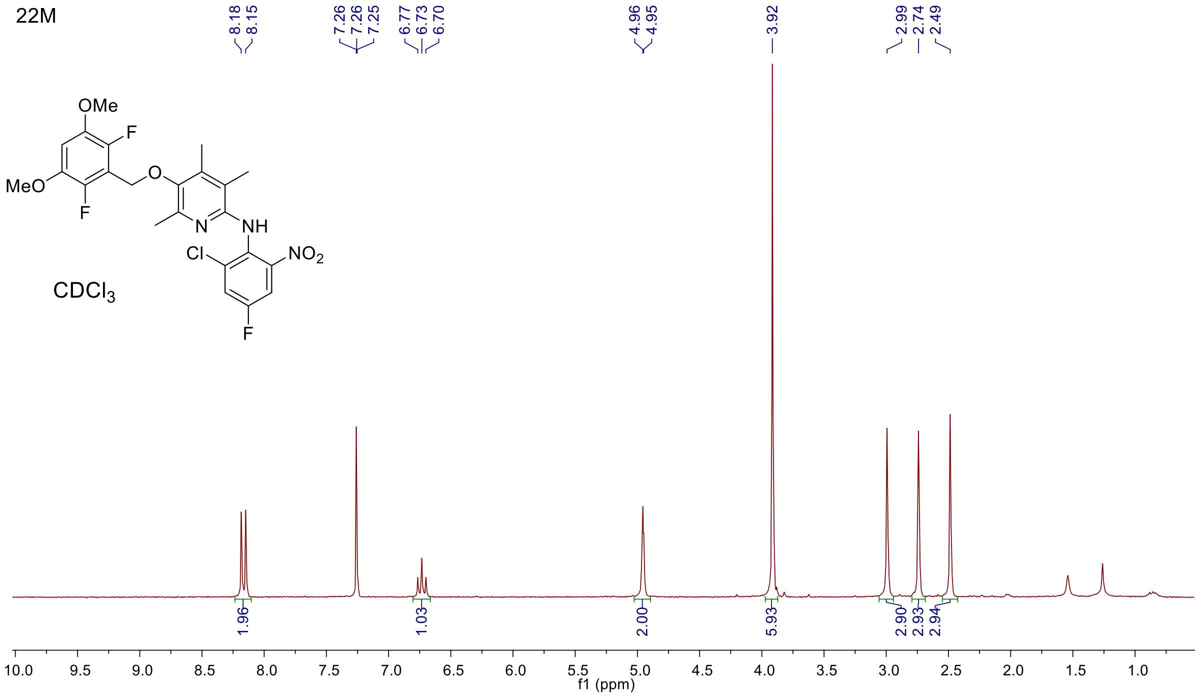

22M

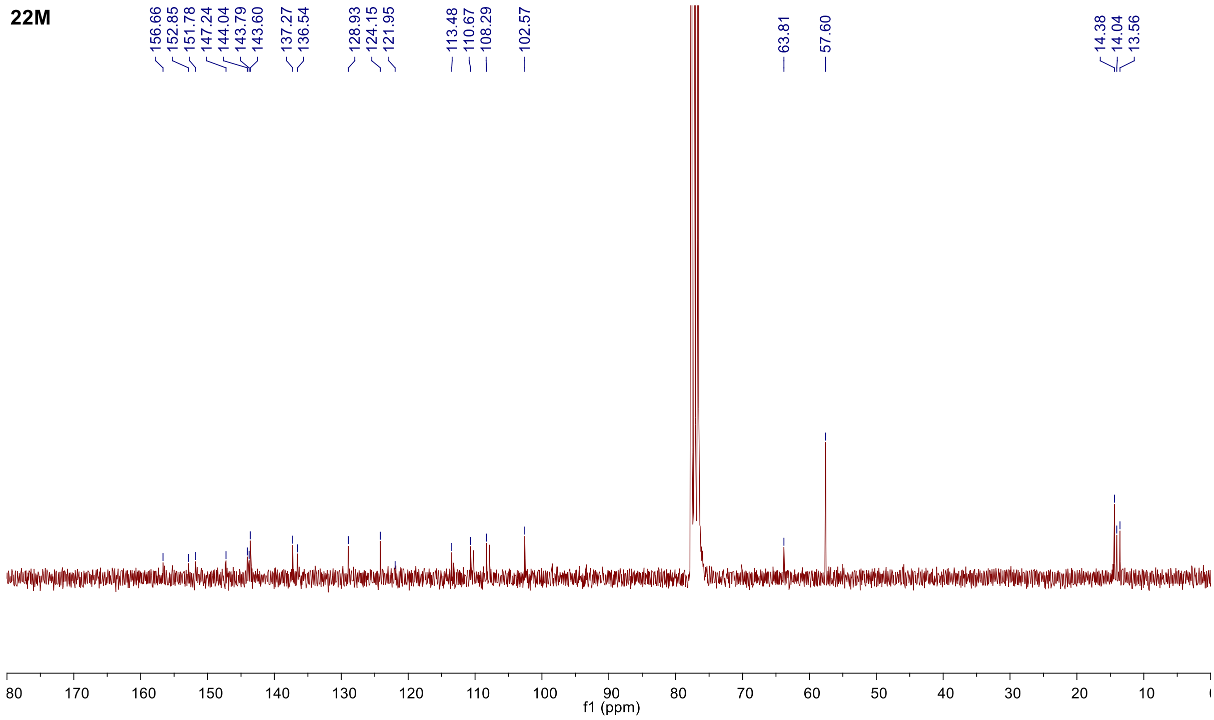

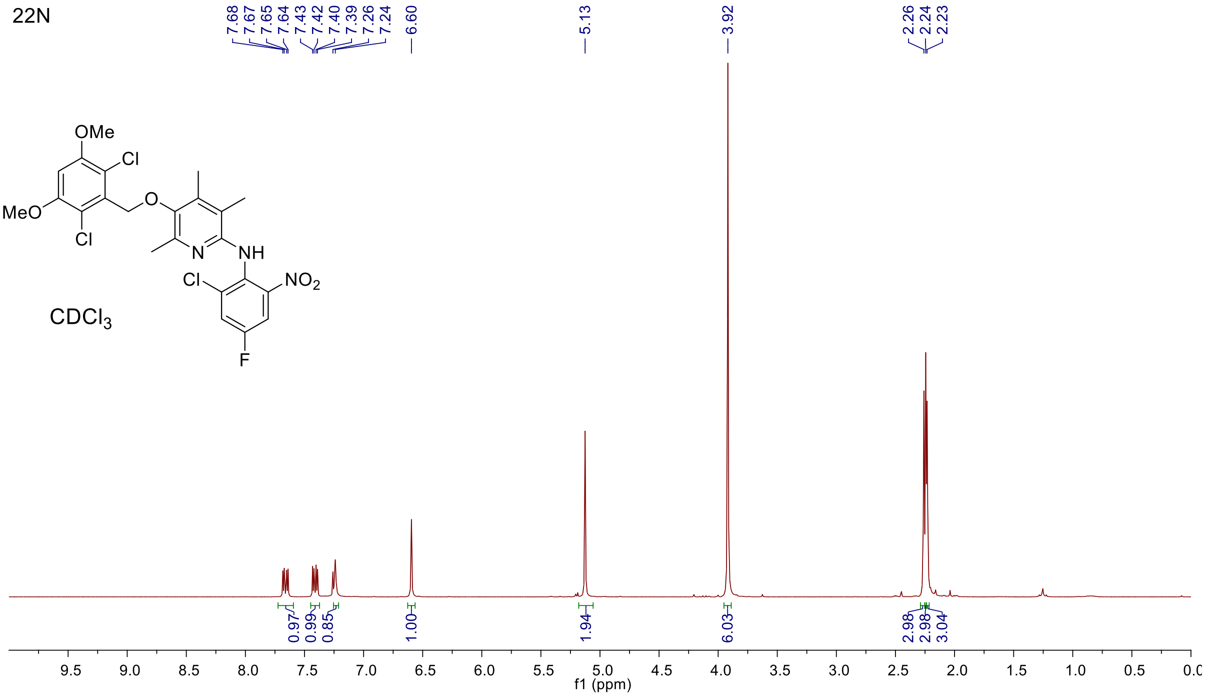
22N
7.68
7.67
7.65
7.64
7.43
7.42
7.40
7.39
7.26
7.24
6.60
5.13
3.92
2.26
2.24
2.23
OMe
Cl
MeO
O
Cl
N
NH
Cl
NO2
F
CDCl3
0.97
0.99
0.85
1.00
1.94
6.03
2.98
2.98
3.04
9.5 9.0 8.5 8.0 7.5 7.0 6.5 6.0 5.5 5.0 4.5 4.0 3.5 3.0 2.5 2.0 1.5 1.0 0.5
f1 (ppm)

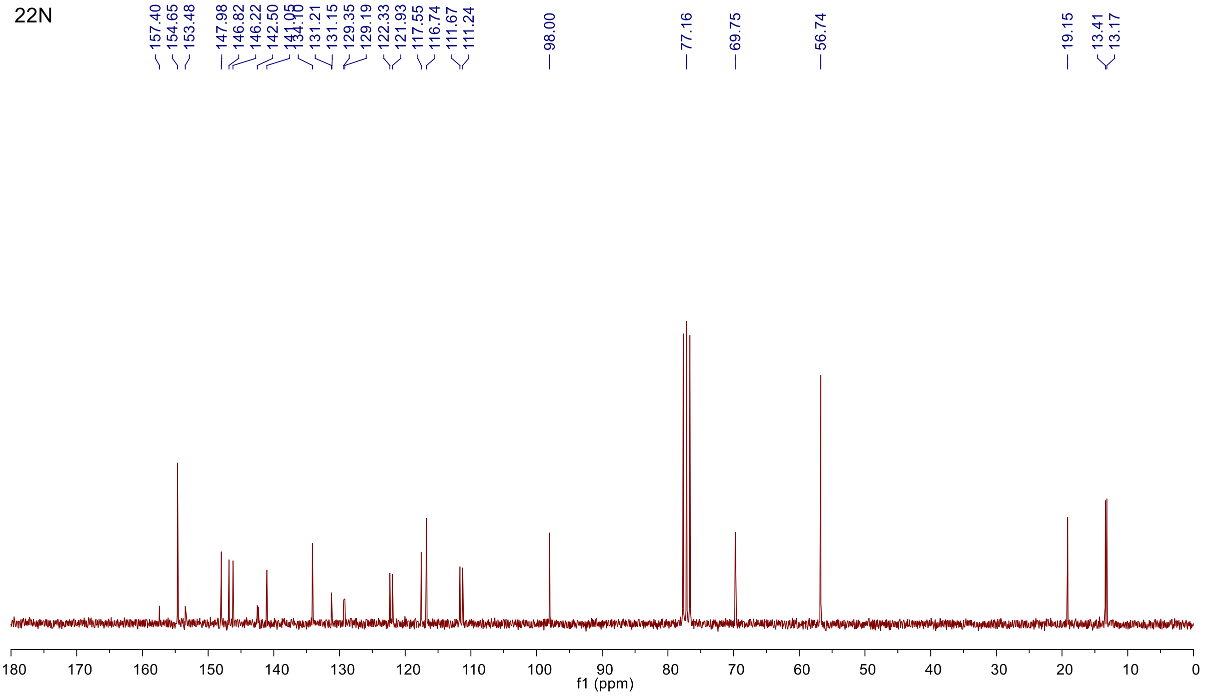
22N
157.40
154.65
153.48
147.98
146.82
146.22
142.50
131.21
131.15
129.35
129.19
122.33
121.93
117.55
116.74
111.67
111.24
98.00
77.16
69.75
56.74
19.15
13.41
13.17
180 170 160 150 140 130 120 110 100 90 80 70 60 50 40 30 20 10 0
f1 (ppm)

22O

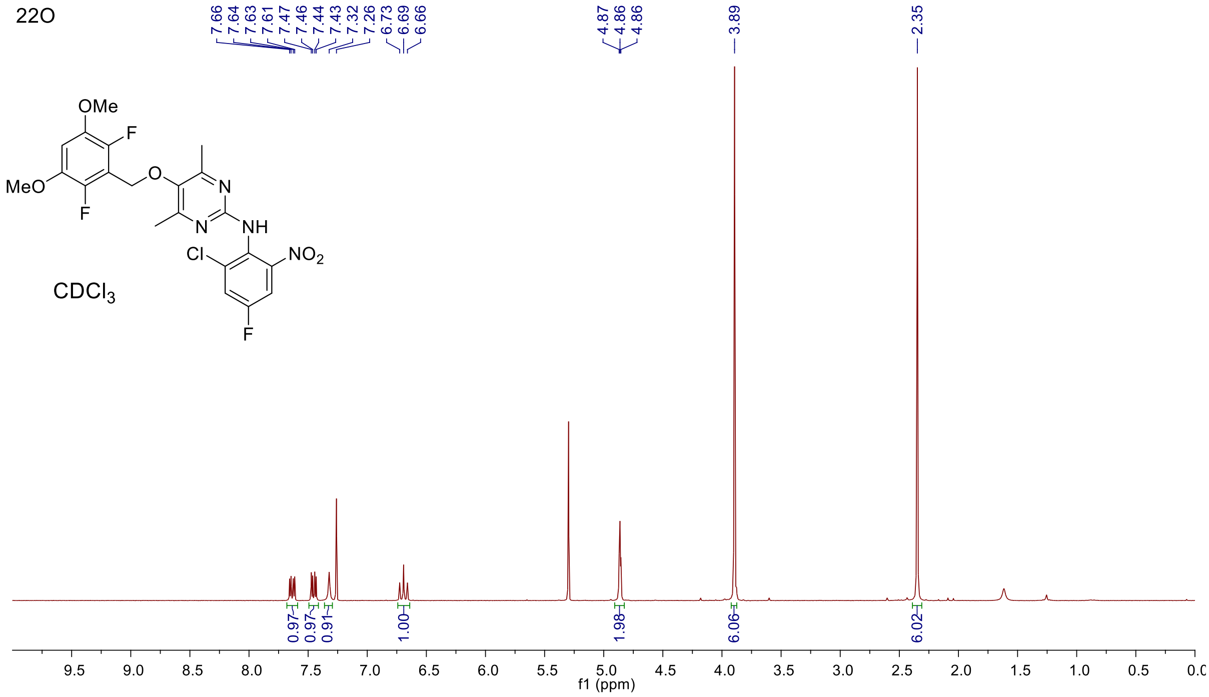
7.66
7.64
7.63
7.61
7.47
7.46
7.44
7.43
7.32
7.26
6.73
6.69
6.66
4.87
4.86
4.86
3.89
2.35
OMe
F
MeO
F
O
N
N
NH
Cl
NO2
F
CDCl3
0.97
0.97
0.91
1.00
1.98
6.06
6.02
f1 (ppm)

22O

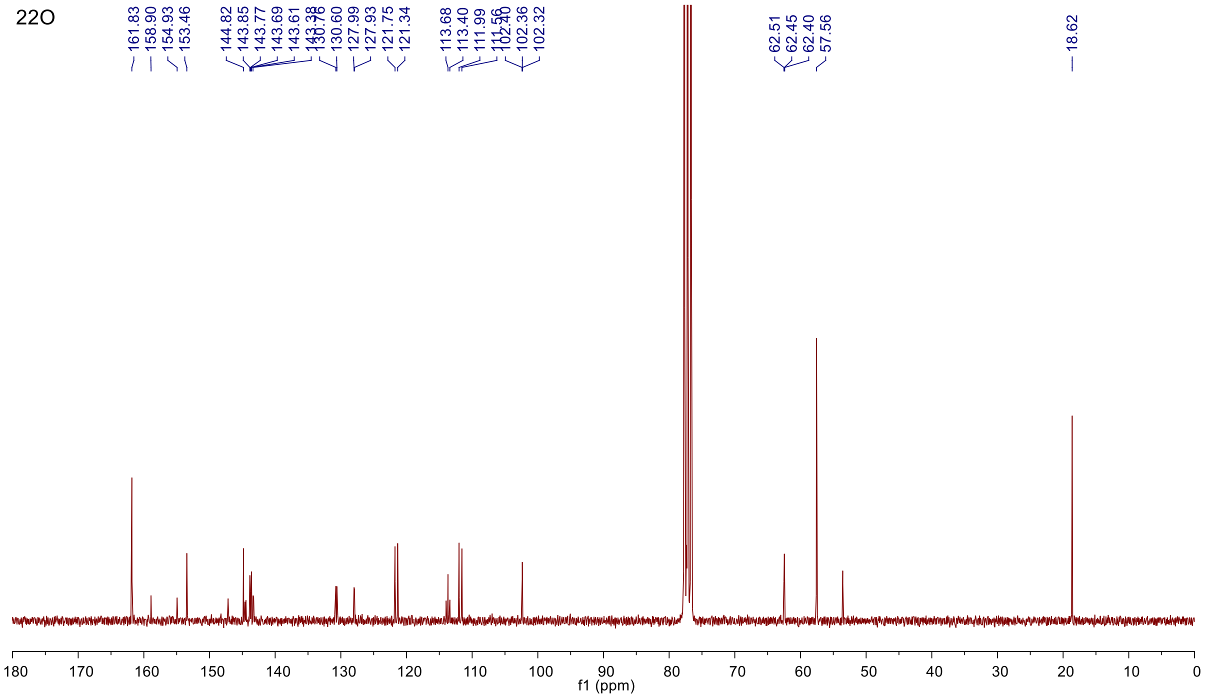
161.83
158.90
154.93
153.46
144.82
143.85
143.77
143.69
143.61
130.60
127.99
127.93
121.75
121.34
113.68
113.40
111.99
102.36
102.32
62.51
62.45
62.40
57.56
18.62
f1 (ppm)

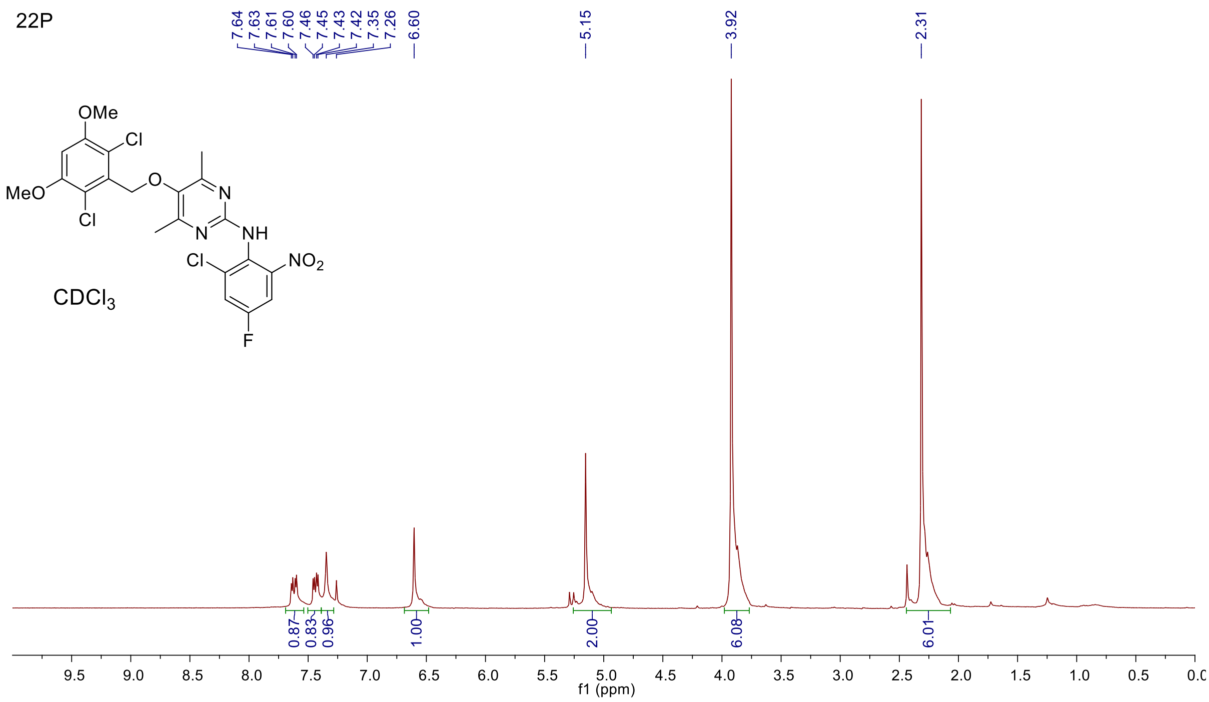
22P
7.64
7.63
7.61
7.60
7.46
7.45
7.43
7.42
7.35
7.26
6.60
5.15
3.92
2.31
OMe
Cl
MeO
O
N
NH
Cl
N
Cl
NO2
F
CDCl3
0.87
0.83
0.96
1.00
2.00
6.08
6.01
f1 (ppm)

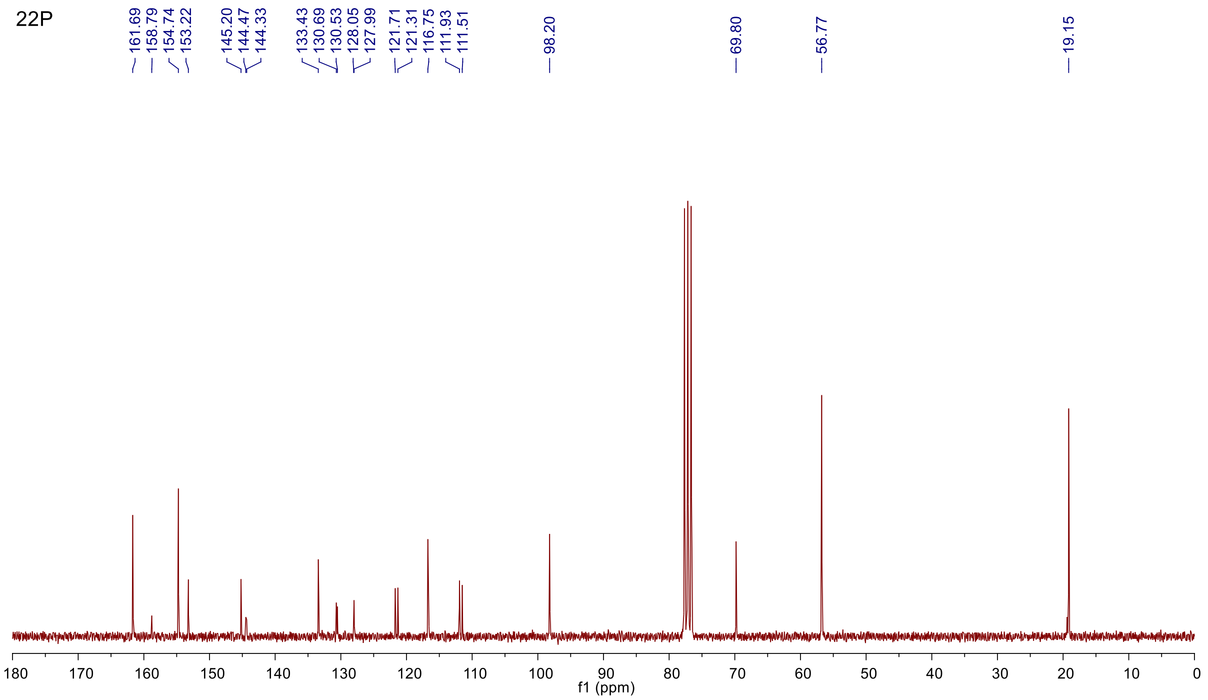
22P
161.69
158.79
154.74
153.22
145.20
144.47
144.33
133.43
130.69
130.53
128.05
127.99
121.71
121.31
116.75
111.93
111.51
98.20
69.80
56.77
19.15
f1 (ppm)

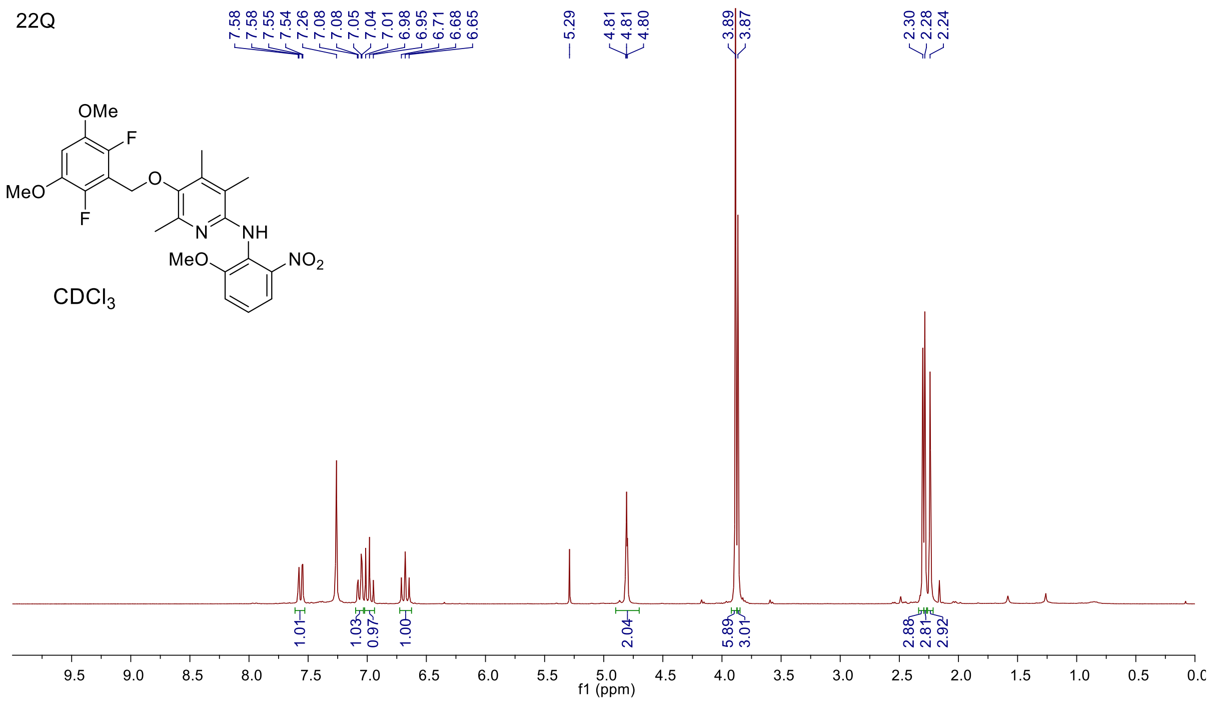
22Q
OMe
F
MeO
F
O
N
NH
MeO
NO2
CDCl3
7.58
7.58
7.55
7.54
7.26
7.08
7.08
7.05
7.04
7.01
6.98
6.95
6.71
6.68
6.65
5.29
4.81
4.81
4.80
3.89
3.87
2.30
2.28
2.24
1.01
1.03
0.97
1.00
2.04
5.89
3.01
2.88
2.81
2.92
f1 (ppm)

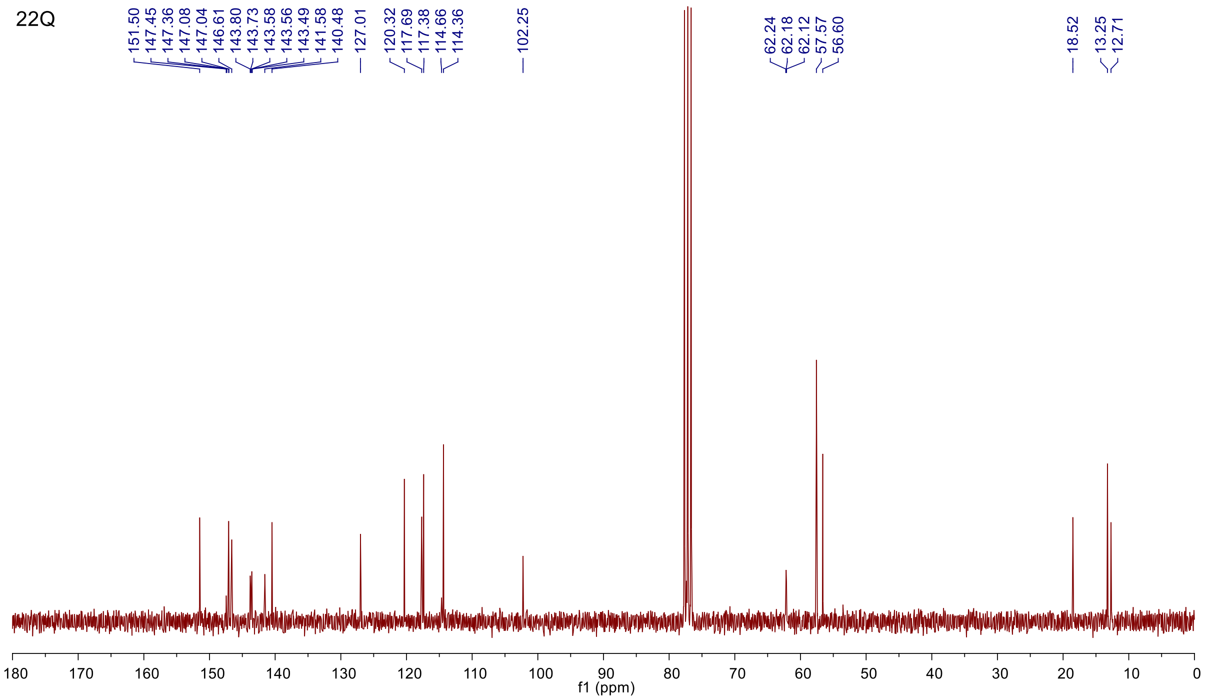
22Q
151.50
147.45
147.36
147.08
147.04
146.61
143.80
143.73
143.58
143.56
143.49
141.58
140.48
127.01
120.32
117.69
117.38
114.66
114.36
102.25
62.24
62.18
62.12
57.57
56.60
18.52
13.25
12.71
f1 (ppm)

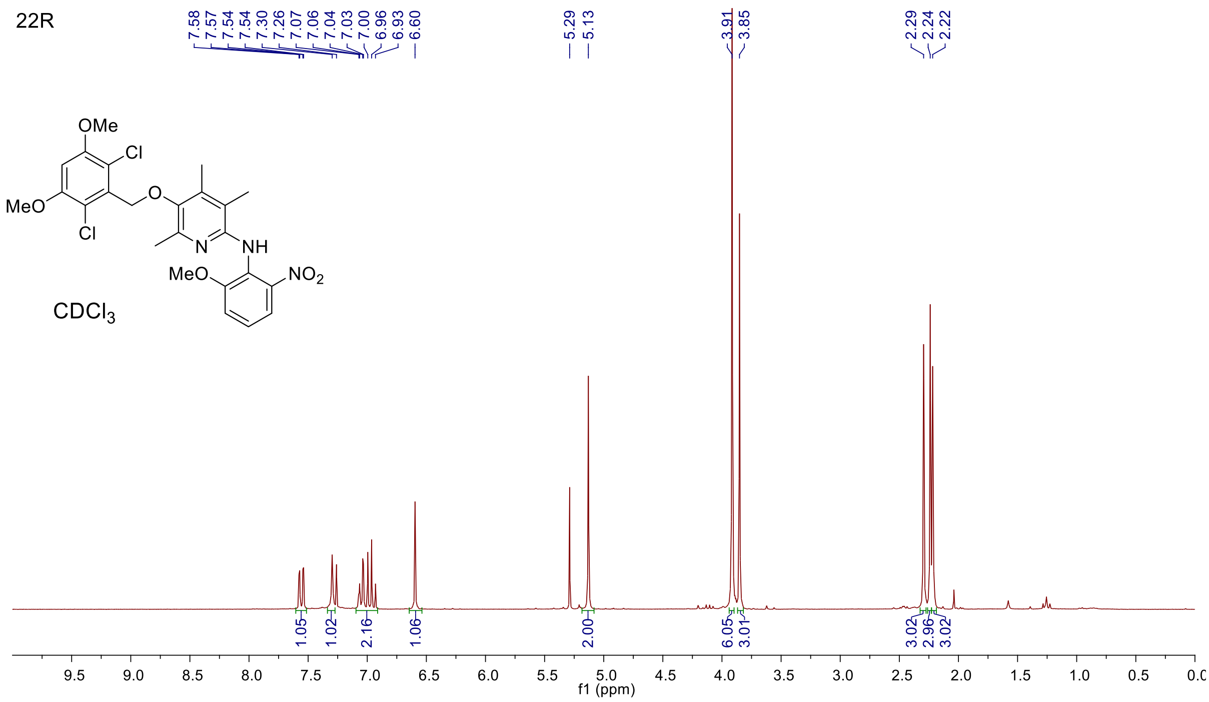
22R
7.58 7.57 7.54 7.54 7.30 7.26 7.07 7.06 7.04 7.03 7.00 6.96 6.93 6.60
5.29 5.13
3.91 3.85
2.29 2.24 2.22
OMe
Cl
MeO
Cl
O
N
NH
MeO
NO2
CDCl3
1.05 1.02 2.16 1.06 2.00 6.05 3.01 3.02 2.96 3.02
f1 (ppm)

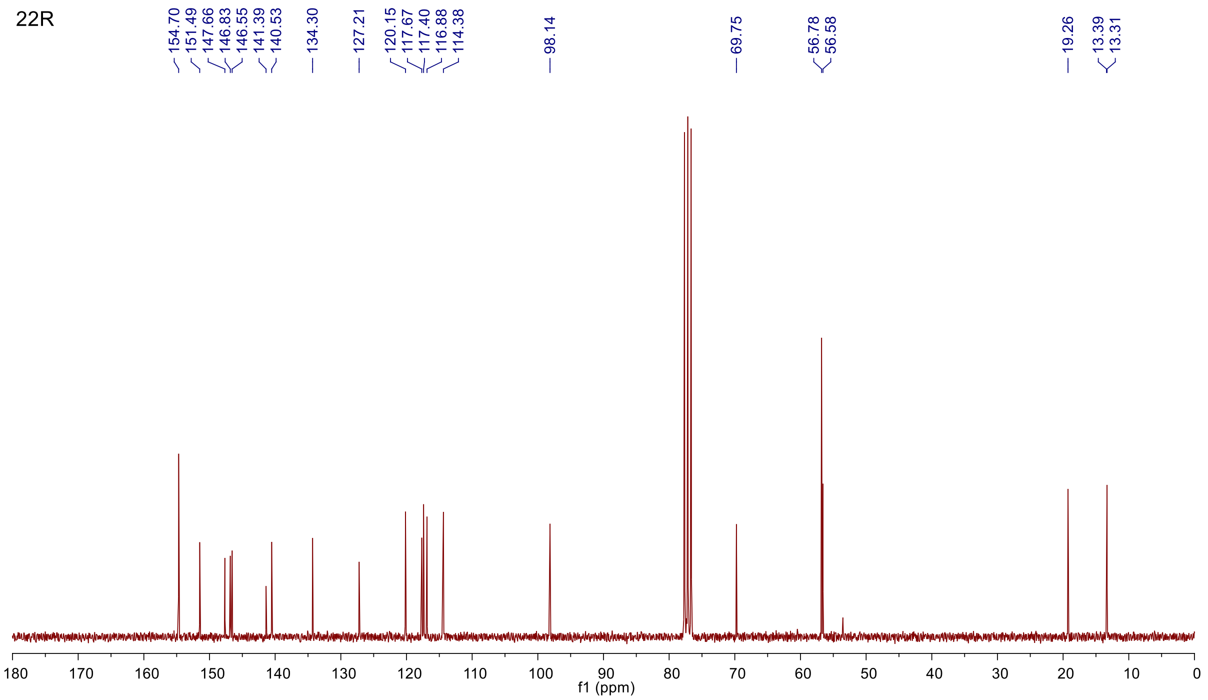
22R
154.70 151.49 147.66 146.83 146.55 141.39 140.53
134.30
127.21
120.15 117.67 117.40 116.88 114.38
98.14
69.75
56.78 56.58
19.26
13.39 13.31
f1 (ppm)

22S

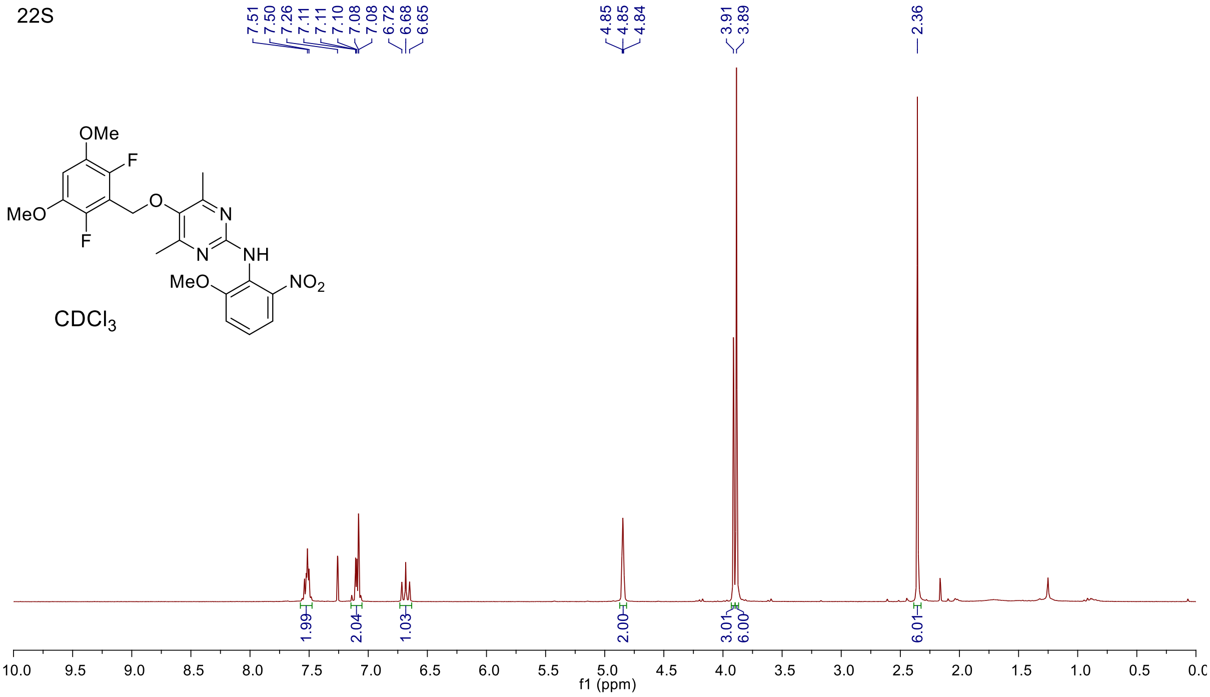

7.51
7.50
7.26
7.11
7.11
7.10
7.08
6.72
6.68
6.65
4.85
4.85
4.84
3.91
3.89
2.36
OMe
F
MeO
F
O
N
N
NH
MeO
NO_2
$CDCl_3$
1.99
2.04
1.03
2.00
3.01
6.00
6.01
f1 (ppm)

22S

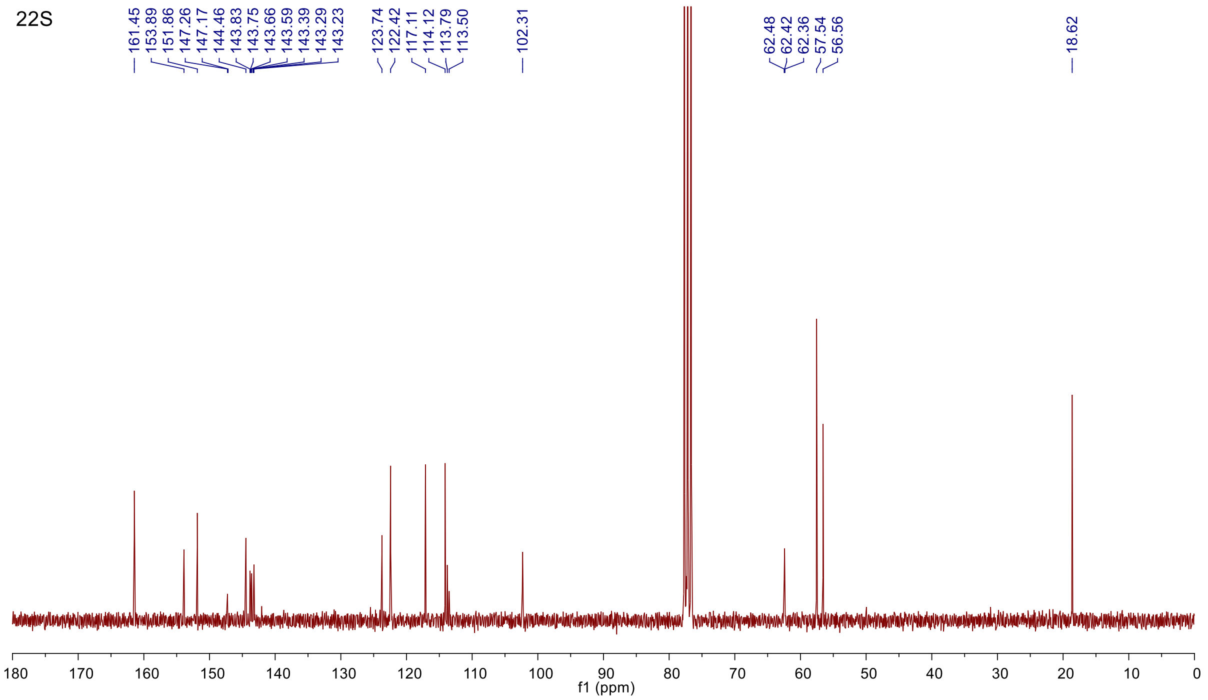

161.45
153.89
151.86
147.26
147.17
144.46
143.83
143.75
143.66
143.59
143.39
143.29
143.23
123.74
122.42
117.11
114.12
113.79
113.50
102.31
62.48
62.42
62.36
57.54
56.56
18.62
f1 (ppm)

22T

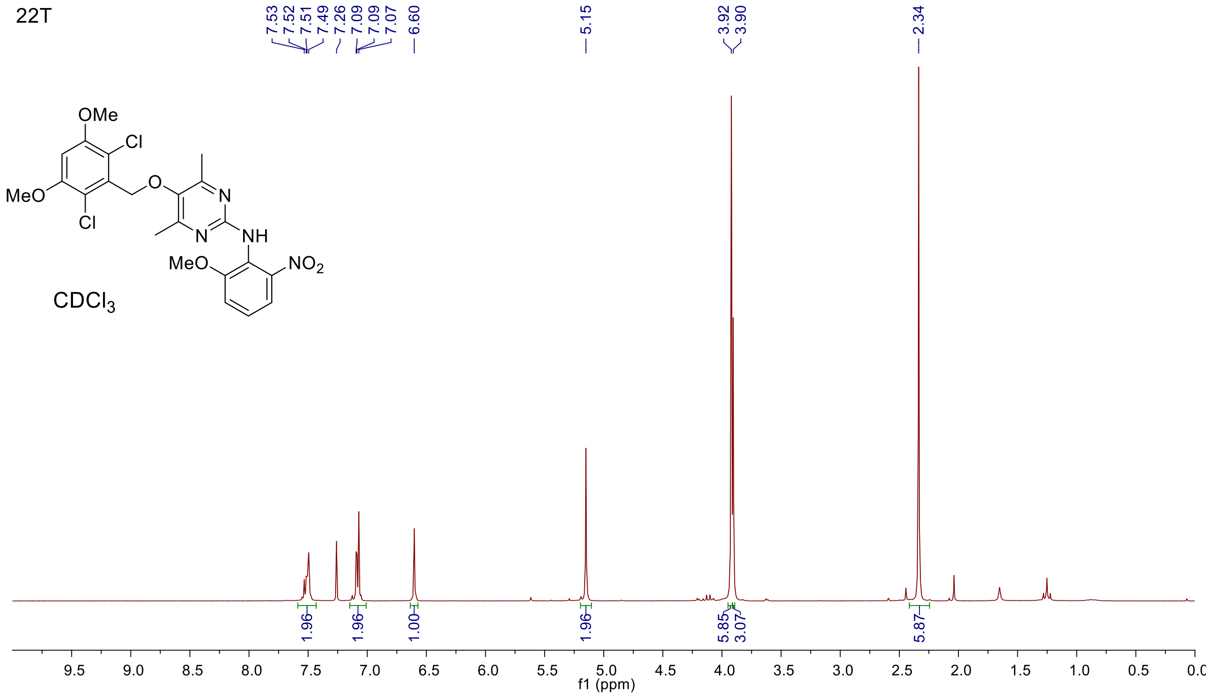
7.53
7.52
7.51
7.49
7.26
7.09
7.09
7.07
6.60
5.15
3.92
3.90
2.34
OMe
Cl
MeO
Cl
O
N
N
NH
MeO
NO_2
$CDCl_3$
1.96
1.96
1.00
1.96
5.85
3.07
5.87
9.5 9.0 8.5 8.0 7.5 7.0 6.5 6.0 5.5 5.0 4.5 4.0 3.5 3.0 2.5 2.0 1.5 1.0 0.5 0.0
f1 (ppm)

22T

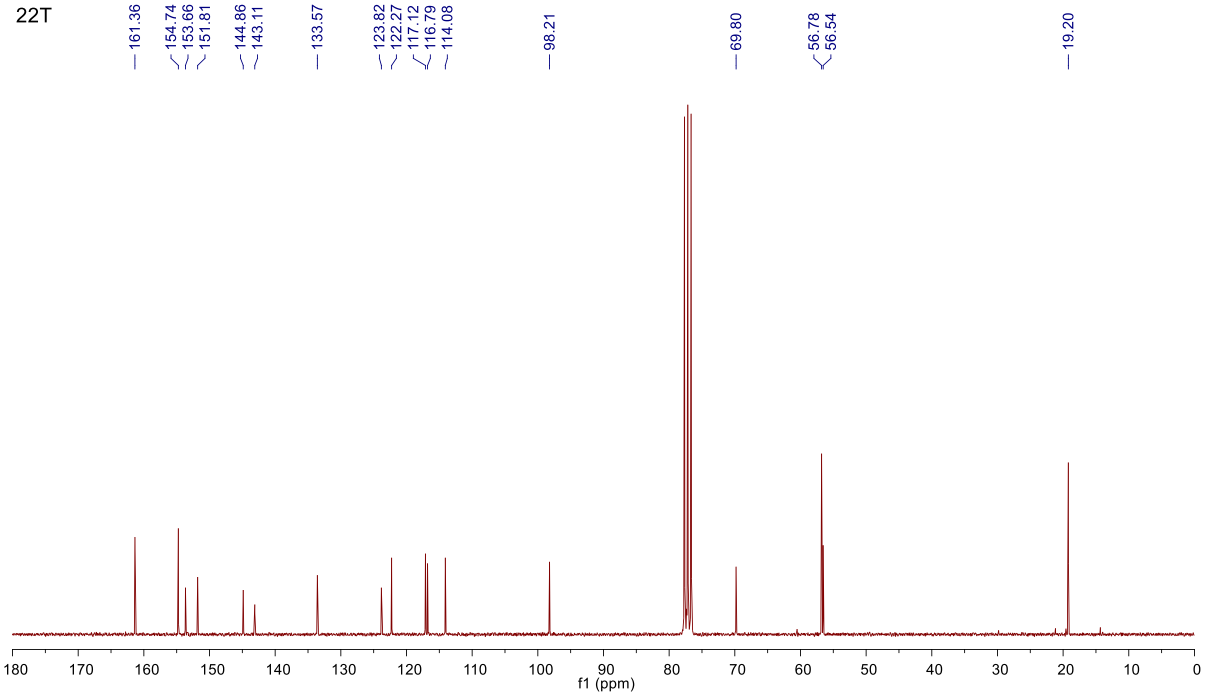
161.36
154.74
153.66
151.81
144.86
143.11
133.57
123.82
122.27
117.12
116.79
114.08
98.21
69.80
56.78
56.54
19.20
180 170 160 150 140 130 120 110 100 90 80 70 60 50 40 30 20 10 0
f1 (ppm)

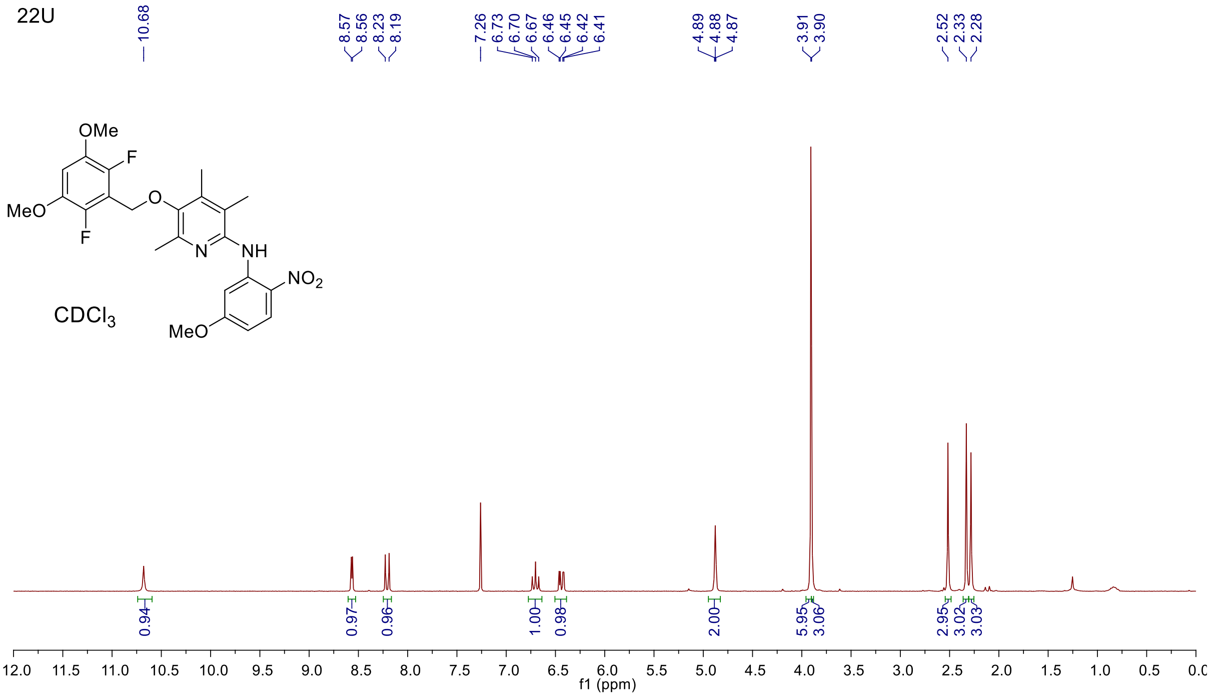
22U
10.68
8.57
8.56
8.23
8.19
7.26
6.73
6.70
6.67
6.46
6.45
6.42
6.41
4.89
4.88
4.87
3.91
3.90
2.52
2.33
2.28
OMe
F
MeO
F
O
N
NH
NO2
MeO
CDCl3
0.94
0.97
0.96
1.00
0.98
2.00
5.95
3.06
2.95
3.02
3.03
f1 (ppm)

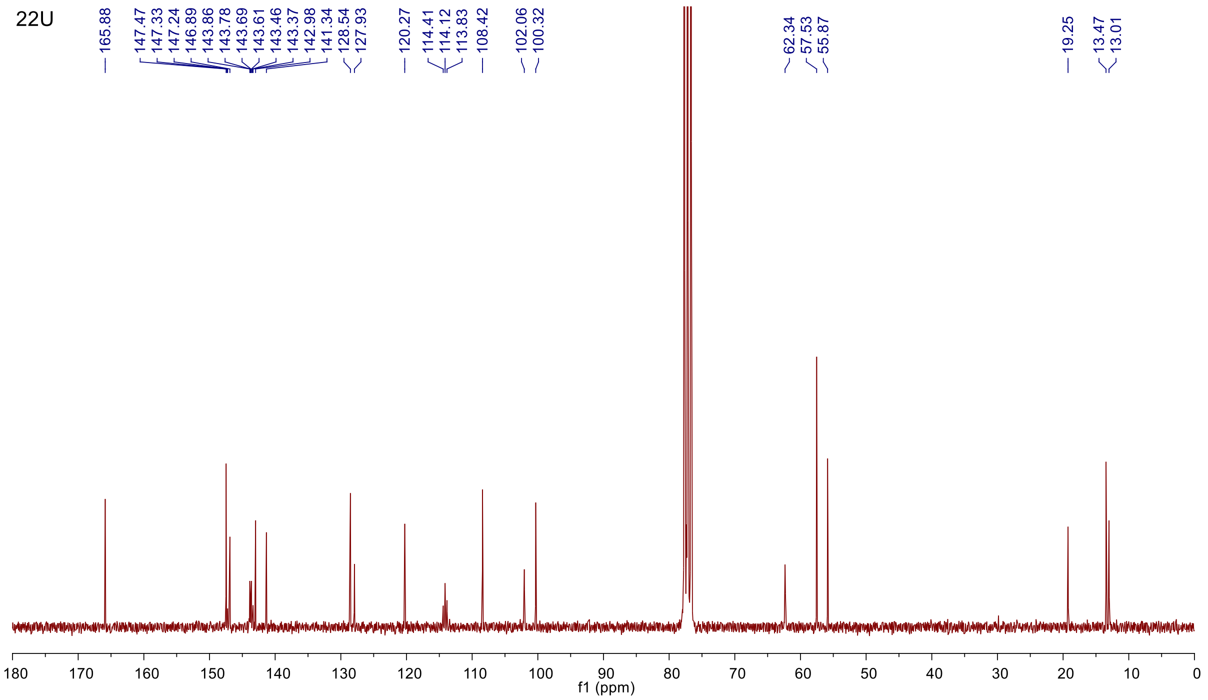
22U
165.88
147.47
147.33
147.24
146.89
143.86
143.78
143.69
143.61
143.46
143.37
142.98
141.34
128.54
127.93
120.27
114.41
114.12
113.83
108.42
102.06
100.32
62.34
57.53
55.87
19.25
13.47
13.01
f1 (ppm)

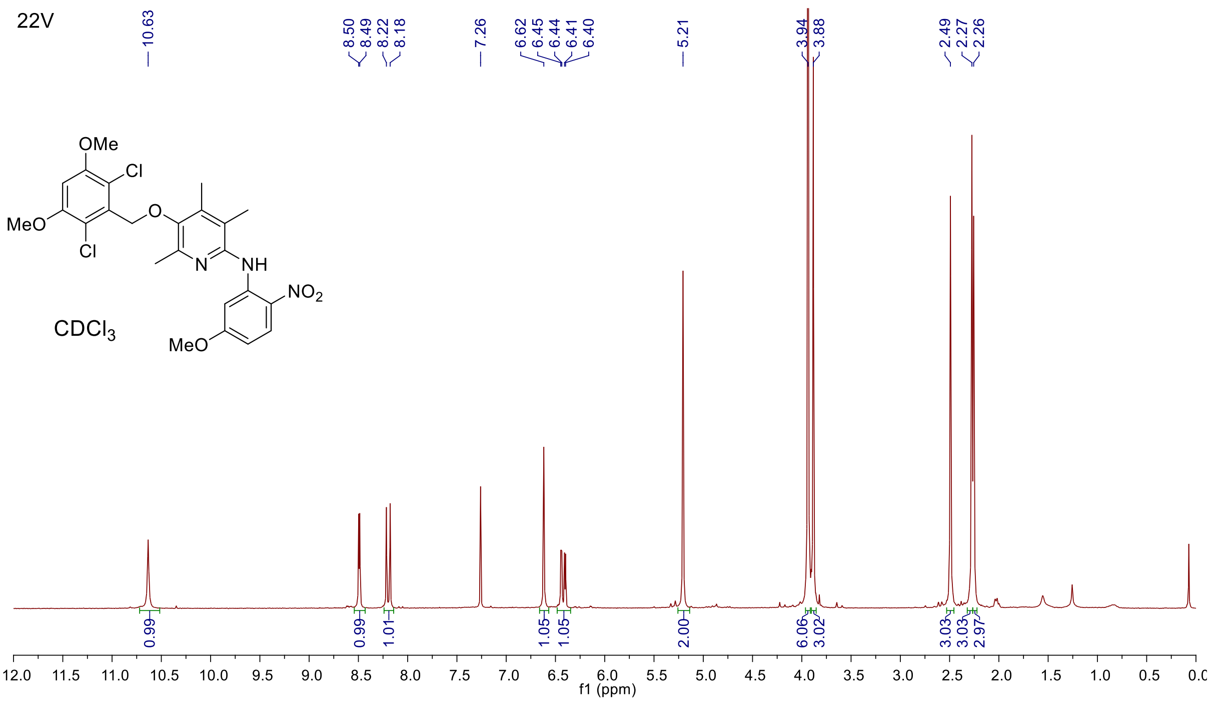

22V
10.63
8.50
8.49
8.22
8.18
7.26
6.62
6.45
6.44
6.41
6.40
5.21
3.94
3.88
2.49
2.27
2.26
OMe
Cl
MeO
O
Cl
N
NH
NO2
CDCl3
MeO
0.99
0.99
1.01
1.05
1.05
2.00
6.06
3.02
3.03
3.03
2.97
12.0
11.5
11.0
10.5
10.0
9.5
9.0
8.5
8.0
7.5
7.0
6.5
6.0
5.5
5.0
4.5
4.0
3.5
3.0
2.5
2.0
1.5
1.0
0.5
f1 (ppm)

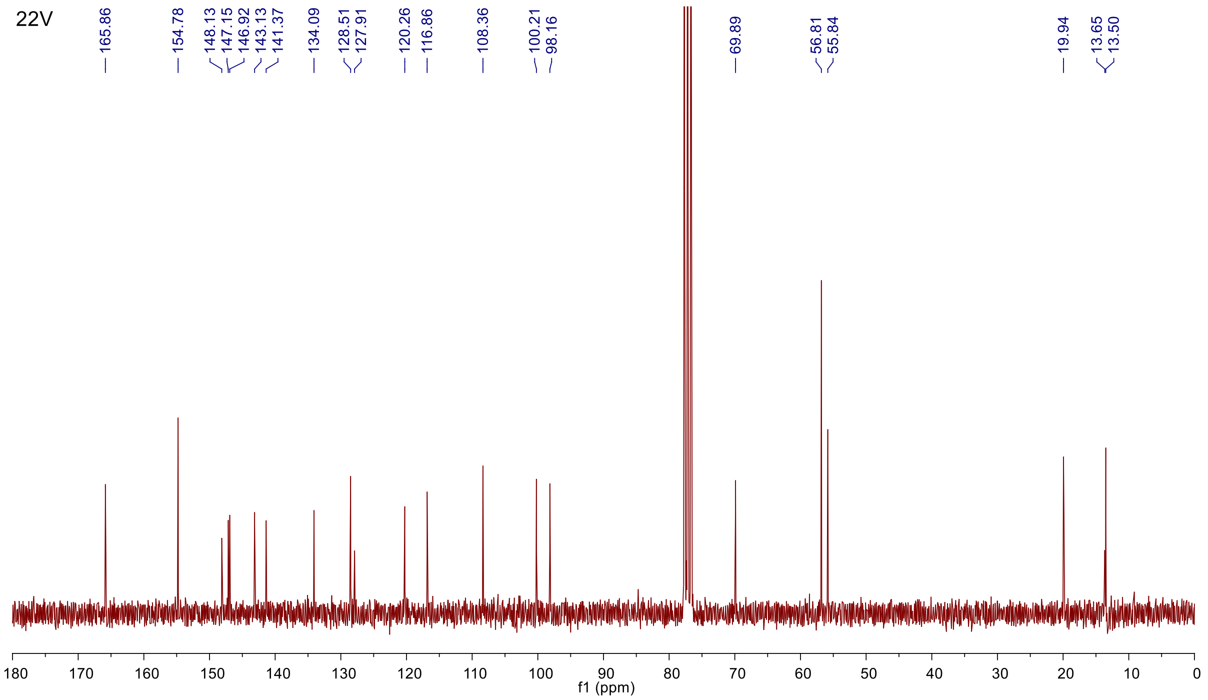

22V
165.86
154.78
148.13
147.15
146.92
143.13
141.37
134.09
128.51
127.91
120.26
116.86
108.36
100.21
98.16
69.89
56.81
55.84
19.94
13.65
13.50
180
170
160
150
140
130
120
110
100
90
80
70
60
50
40
30
20
10
0
f1 (ppm)

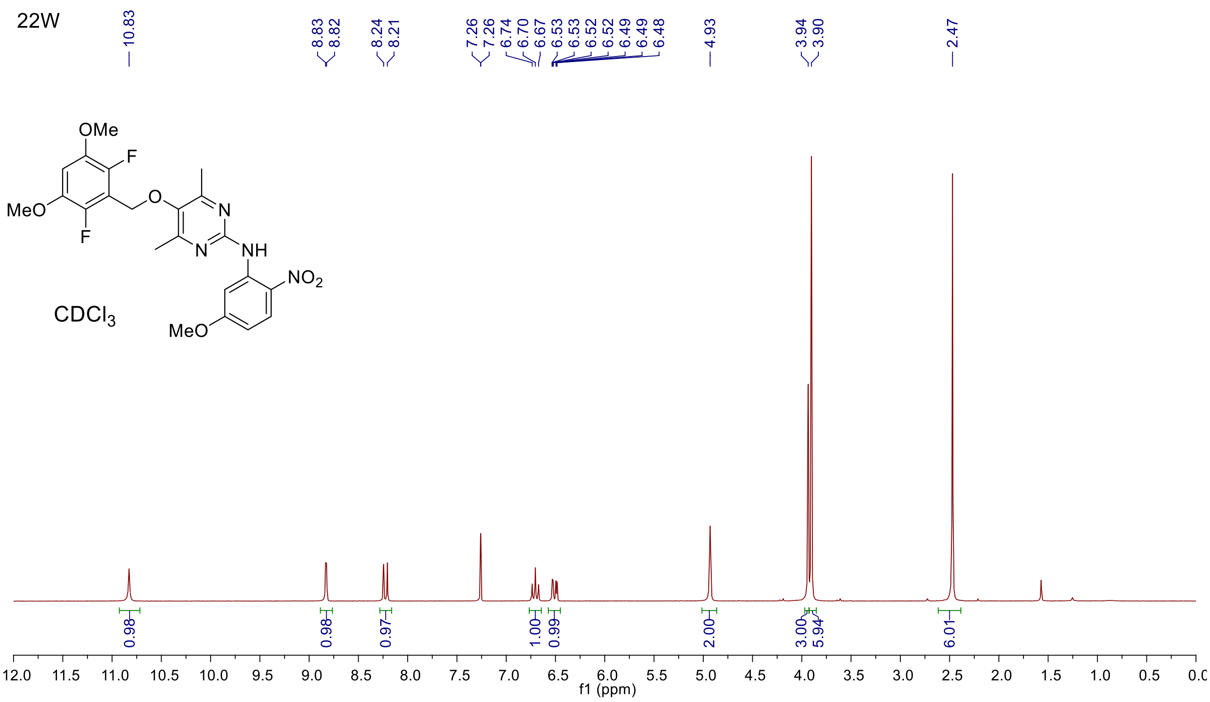
22W
10.83
8.83
8.82
8.24
8.21
7.26
7.26
6.74
6.70
6.67
6.53
6.53
6.52
6.52
6.49
6.49
6.48
4.93
3.94
3.90
2.47
OMe
F
MeO
O
N
F
N
NH
NO2
CDCl3
MeO
0.98
0.98
0.97
1.00
0.99
2.00
3.00
5.94
6.01
12.0 11.5 11.0 10.5 10.0 9.5 9.0 8.5 8.0 7.5 7.0 6.5 6.0 5.5 5.0 4.5 4.0 3.5 3.0 2.5 2.0 1.5 1.0 0.5 0.0
f1 (ppm)

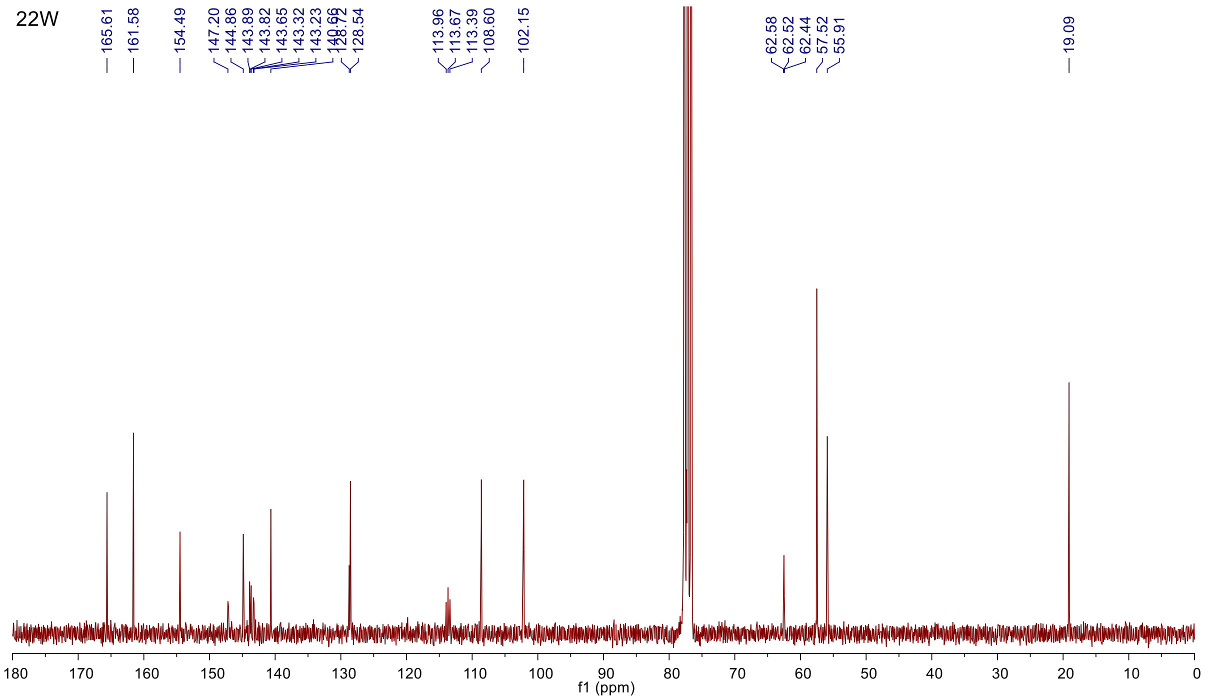
22W
165.61
161.58
154.49
147.20
144.86
143.89
143.82
143.65
143.32
143.23
128.72
128.54
113.96
113.67
113.39
108.60
102.15
62.58
62.52
62.44
57.52
55.91
19.09
180 170 160 150 140 130 120 110 100 90 80 70 60 50 40 30 20 10 0
f1 (ppm)

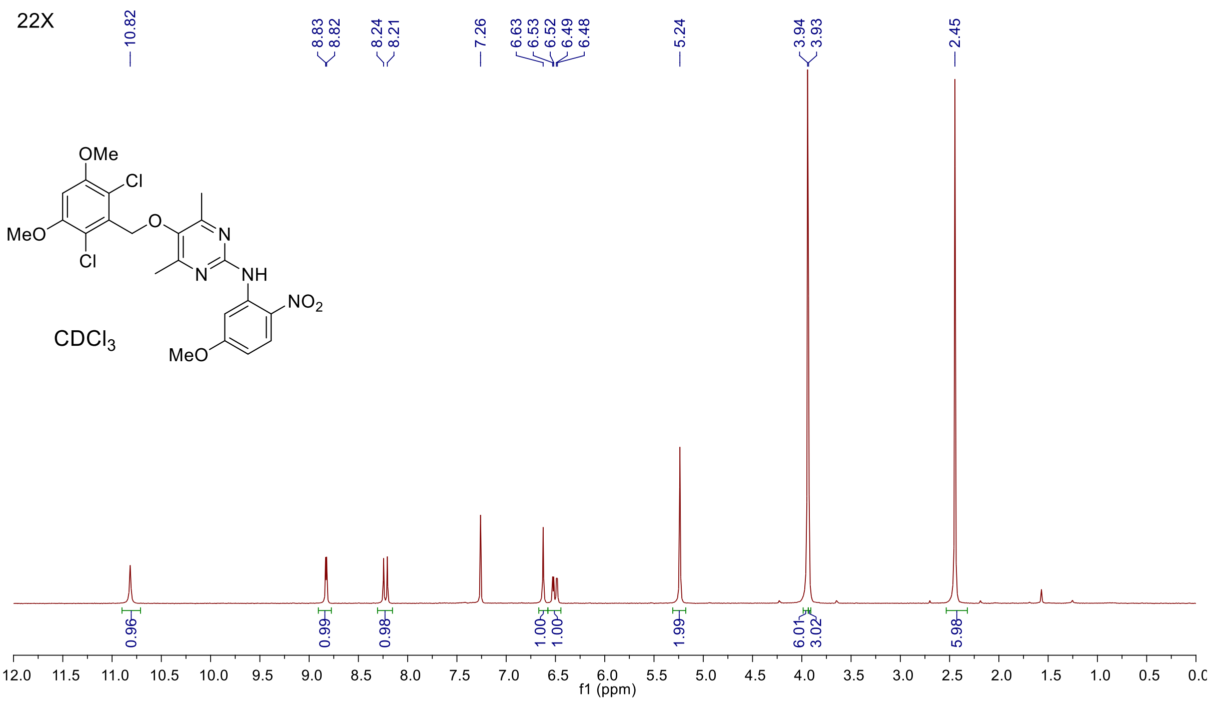
22X
10.82
8.83
8.82
8.24
8.21
7.26
6.63
6.53
6.52
6.49
6.48
5.24
3.94
3.93
2.45
OMe
Cl
MeO
O
N
N
NH
Cl
NO2
CDCl3
MeO
0.96
0.99
0.98
1.00
1.00
1.99
6.01
3.02
5.98
f1 (ppm)

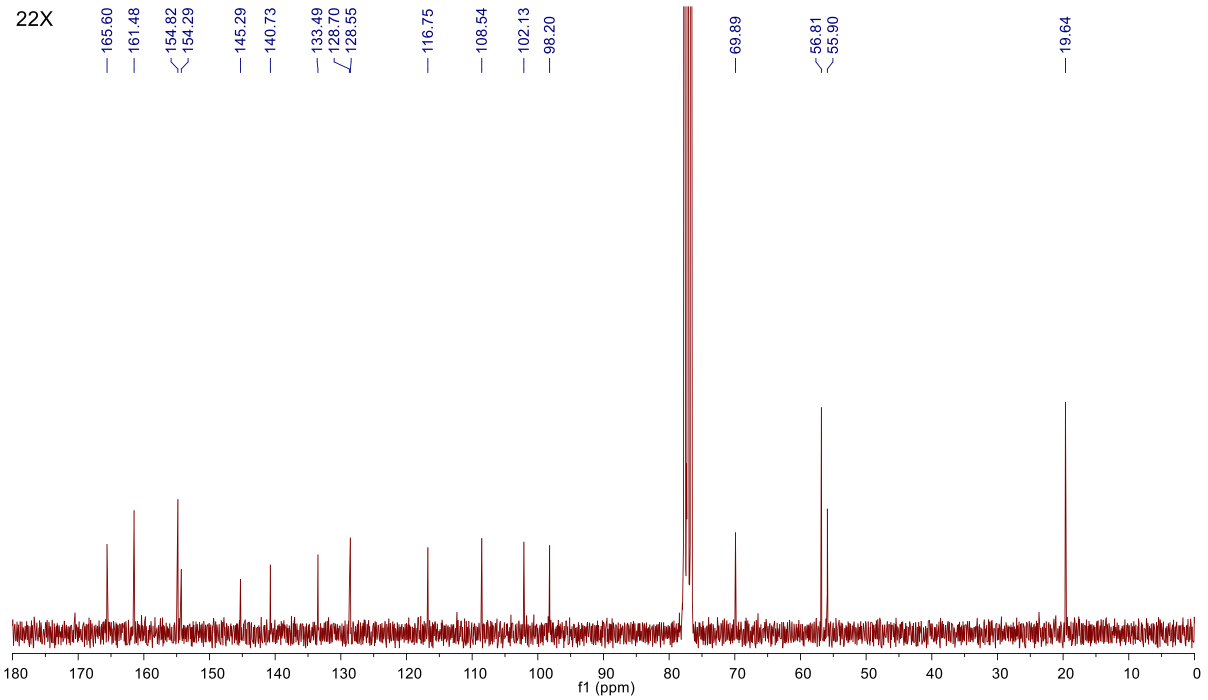
22X
165.60
161.48
154.82
154.29
145.29
140.73
133.49
128.70
128.55
116.75
108.54
102.13
98.20
69.89
56.81
55.90
19.64
f1 (ppm)

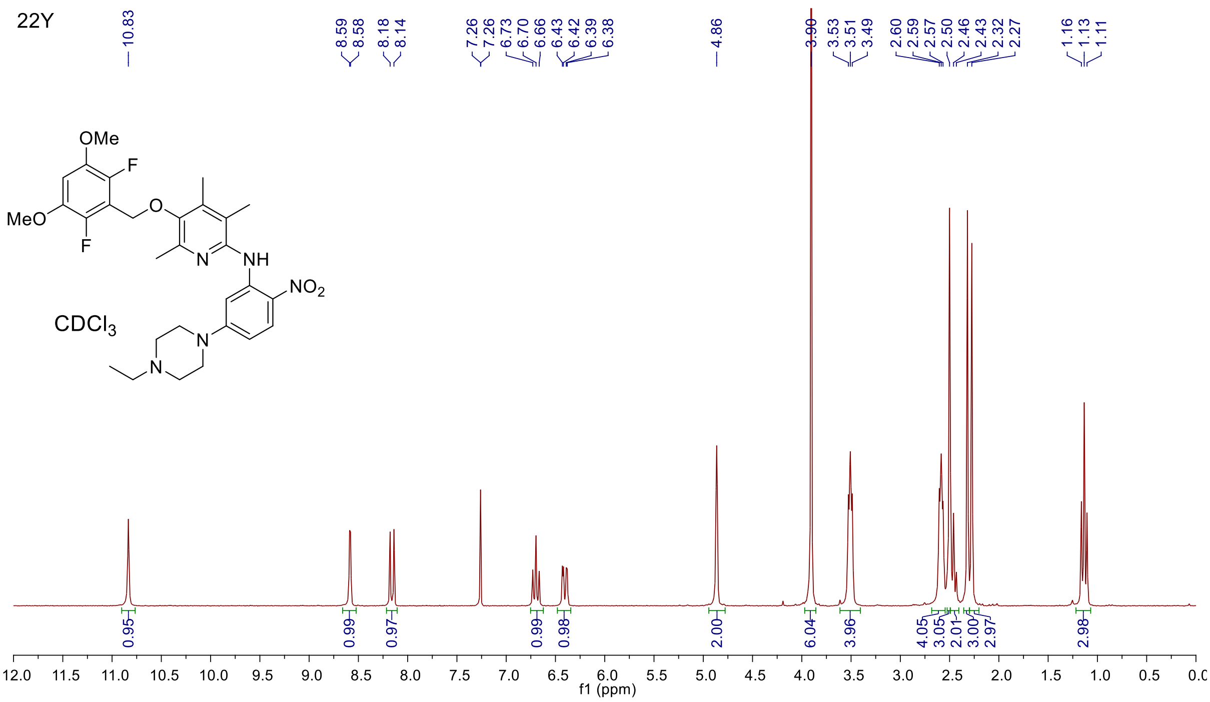
22Y
10.83
8.59
8.58
8.18
8.14
7.26
7.26
6.73
6.70
6.66
6.43
6.42
6.39
6.38
4.86
3.53
3.51
3.49
2.60
2.59
2.57
2.50
2.46
2.43
2.32
2.27
1.16
1.13
1.11
OMe
F
MeO
F
O
N
NH
NO2
N
N
CDCl3
0.95
0.99
0.97
0.99
0.98
2.00
6.04
3.96
4.05
3.05
2.01
3.00
2.97
2.98
f1 (ppm)

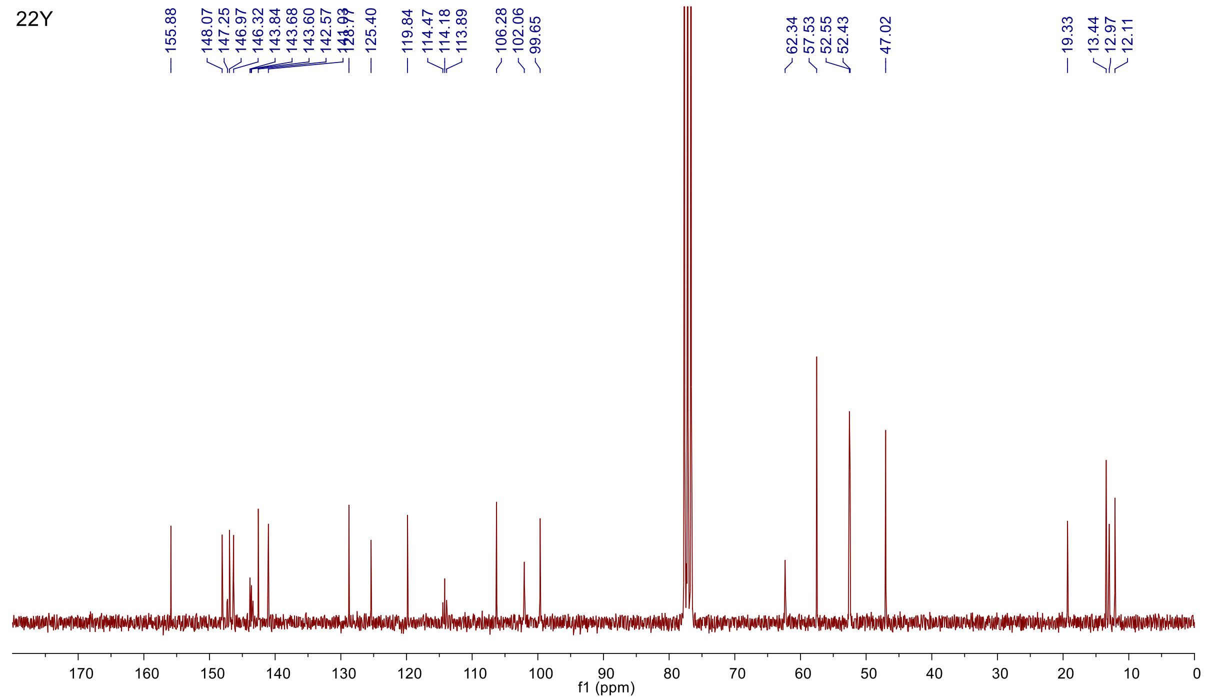
22Y
155.88
148.07
147.25
146.97
146.32
143.84
143.68
143.60
142.57
125.40
119.84
114.47
114.18
113.89
106.28
102.06
99.65
62.34
57.53
52.55
52.43
47.02
19.33
13.44
12.97
12.11
f1 (ppm)

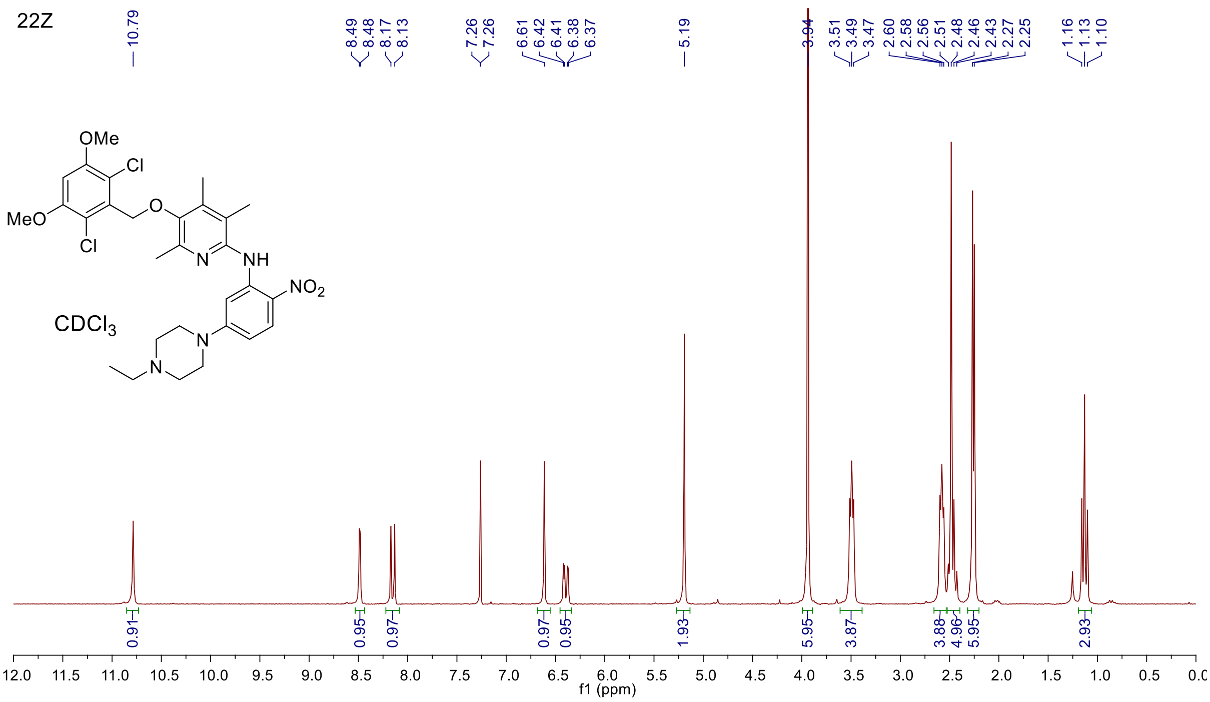
22Z
CDCl3
OMe
Cl
MeO
Cl
O
N
NH
NO2
N
N
f1 (ppm)

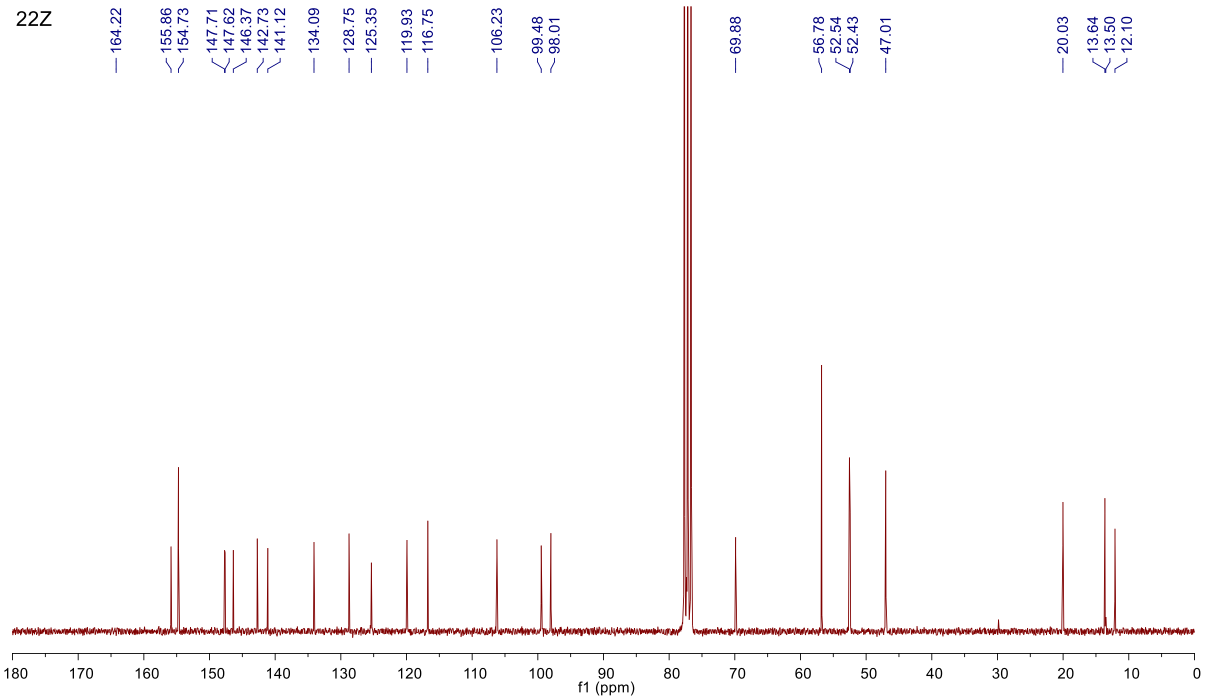
22Z
f1 (ppm)

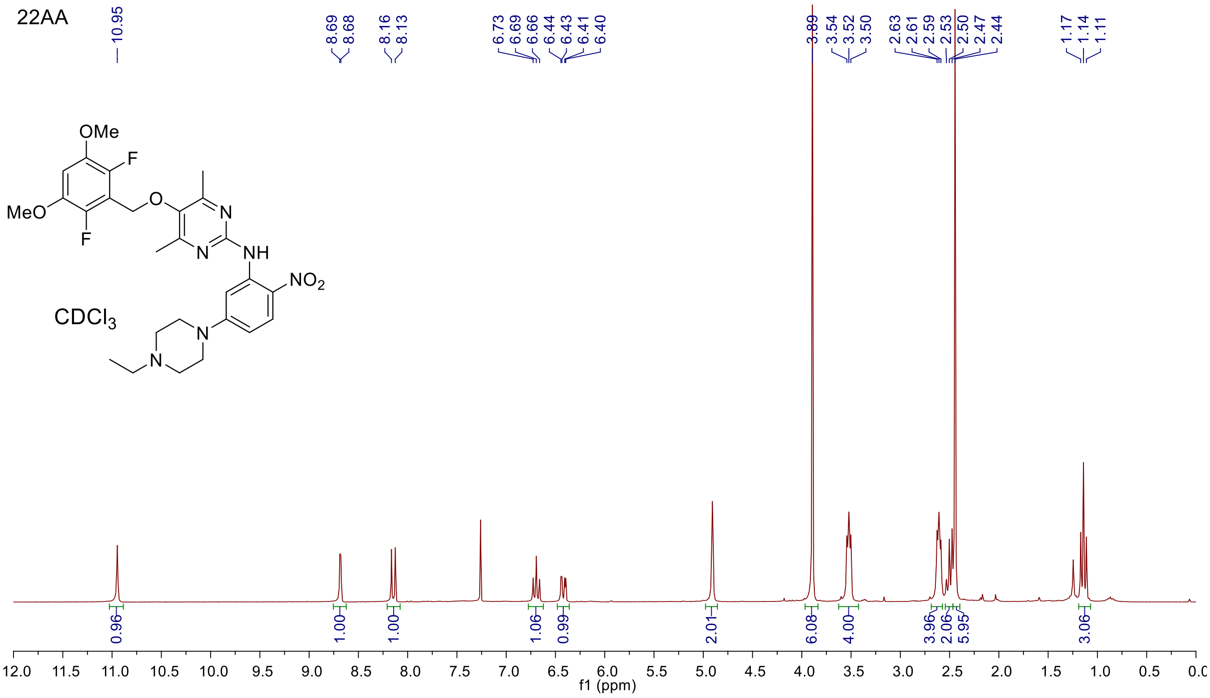
22AA
10.95
8.69
8.68
8.16
8.13
6.73
6.69
6.66
6.44
6.43
6.41
6.40
3.89
3.54
3.52
3.50
2.63
2.61
2.59
2.53
2.50
2.47
2.44
1.17
1.14
1.11
OMe
F
O
N
MeO
F
N
NH
NO2
CDCl3
N
N
0.96
1.00
1.00
1.06
0.99
2.01
6.08
4.00
3.96
2.06
5.95
3.06
f1 (ppm)

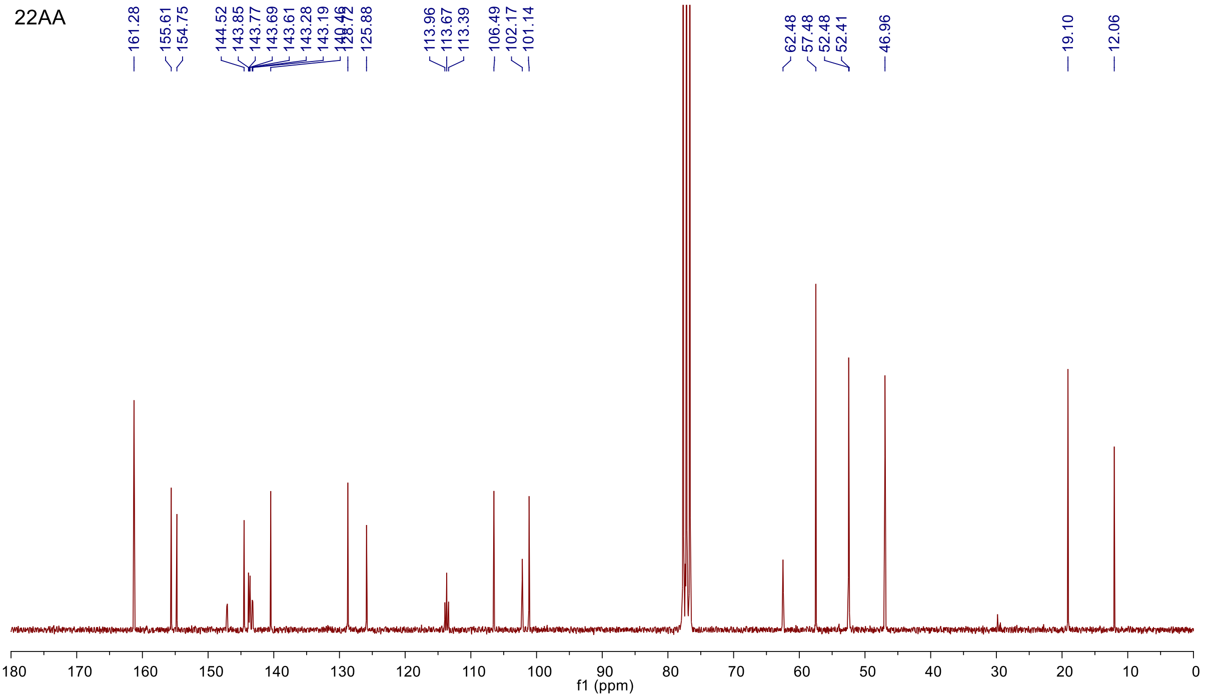
22AA
161.28
155.61
154.75
144.52
143.85
143.77
143.69
143.61
143.28
143.19
140.46
128.72
125.88
113.96
113.67
113.39
106.49
102.17
101.14
62.48
57.48
52.48
52.41
46.96
19.10
12.06
f1 (ppm)

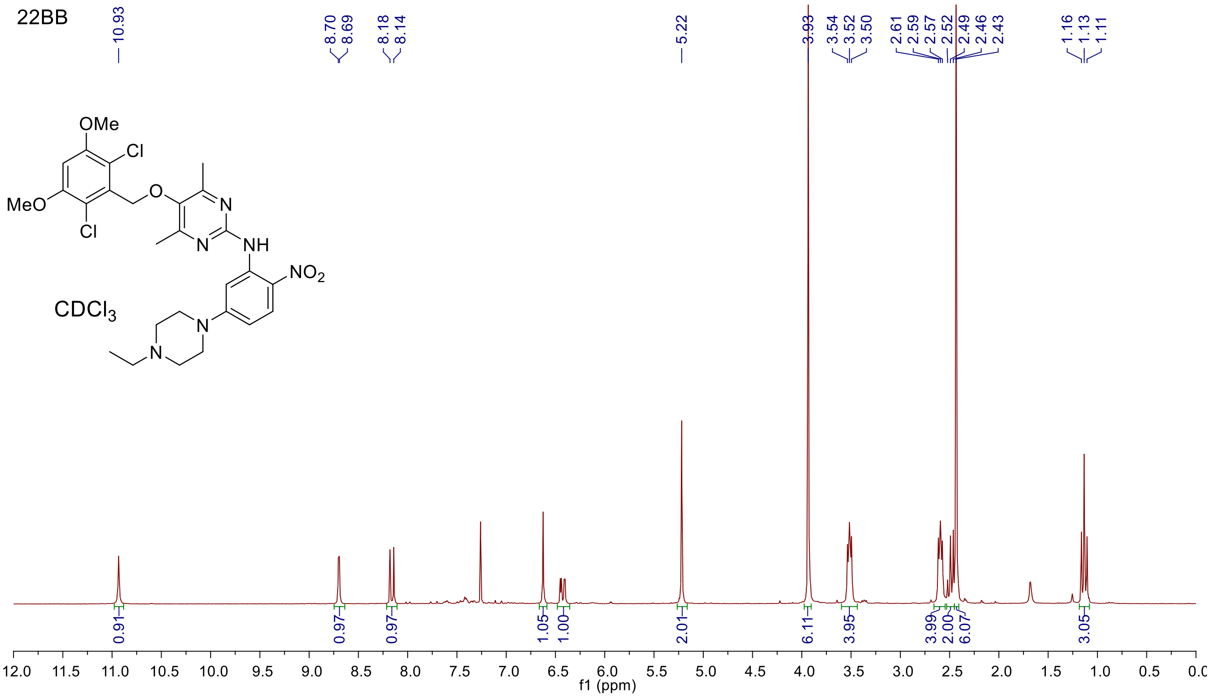
22BB
10.93
8.70
8.69
8.18
8.14
5.22
3.93
3.54
3.52
3.50
2.61
2.59
2.57
2.52
2.49
2.46
2.43
1.16
1.13
1.11
OMe
Cl
MeO
O
N
Cl
N
NH
NO2
CDCl3
N
N
0.91
0.97
0.97
1.05
1.00
2.01
6.11
3.95
3.99
2.00
6.07
3.05
f1 (ppm)

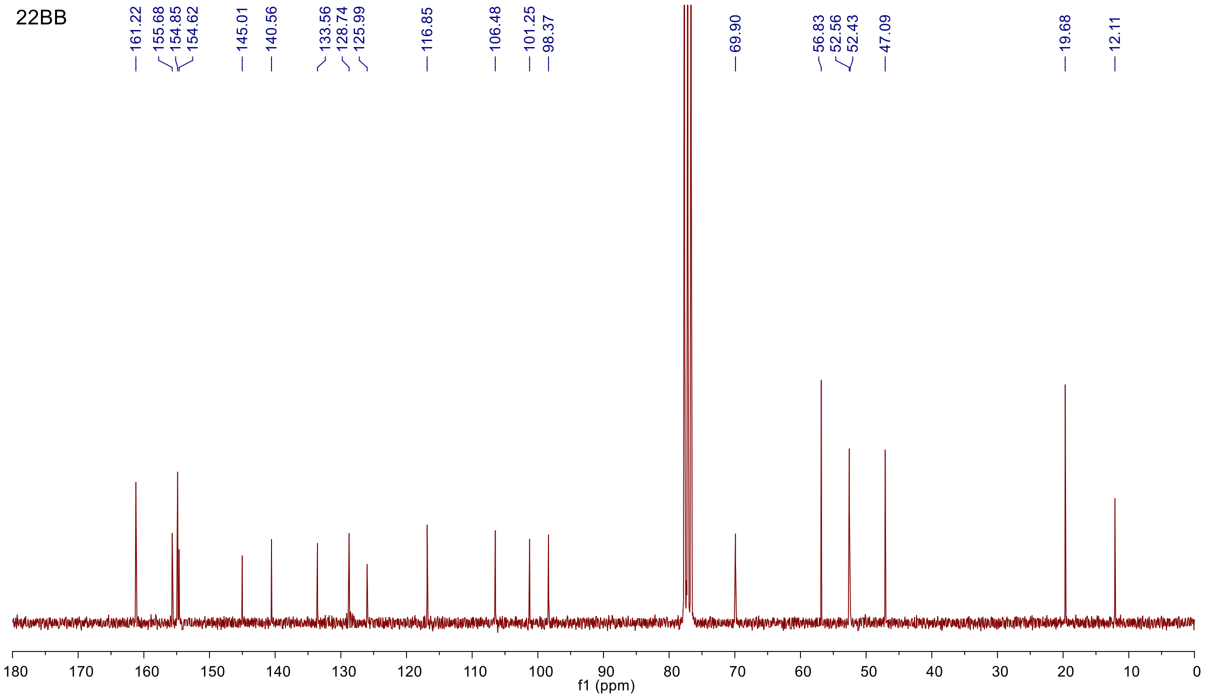
22BB
161.22
155.68
154.85
154.62
145.01
140.56
133.56
128.74
125.99
116.85
106.48
101.25
98.37
69.90
56.83
52.56
52.43
47.09
19.68
12.11
f1 (ppm)

23A

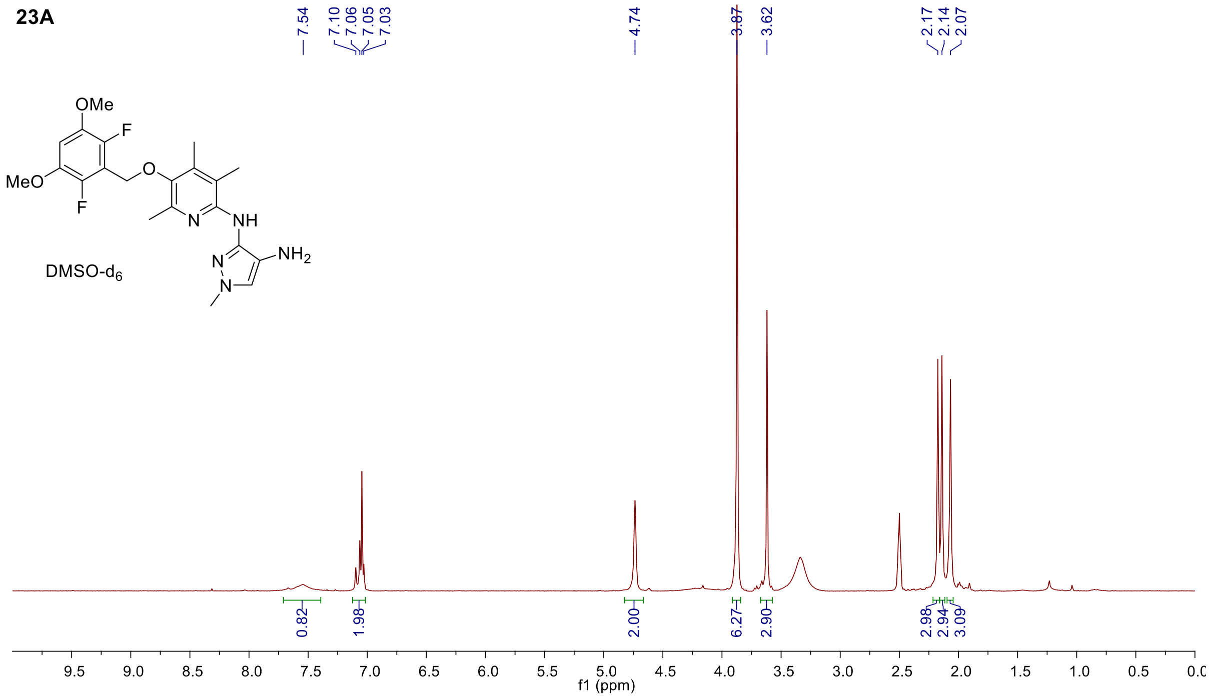
OMe
F
MeO
O
F
N
NH
NH2
N
N
DMSO-d6
7.54
7.10
7.06
7.05
7.03
4.74
3.87
3.62
2.17
2.14
2.07
0.82
1.98
2.00
6.27
2.90
2.98
2.94
3.09
f1 (ppm)

23A

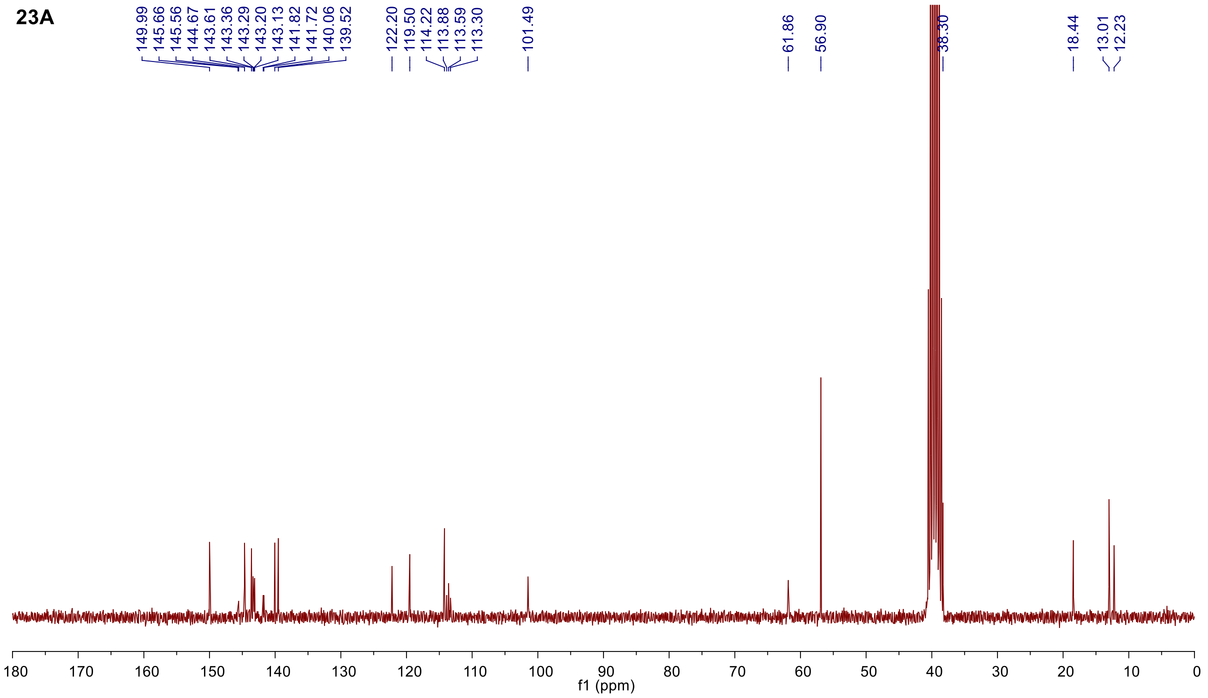
149.99
145.66
145.56
144.67
143.61
143.36
143.29
143.20
143.13
141.82
141.72
140.06
139.52
122.20
119.50
114.22
113.88
113.59
113.30
101.49
61.86
56.90
38.30
18.44
13.01
12.23
f1 (ppm)

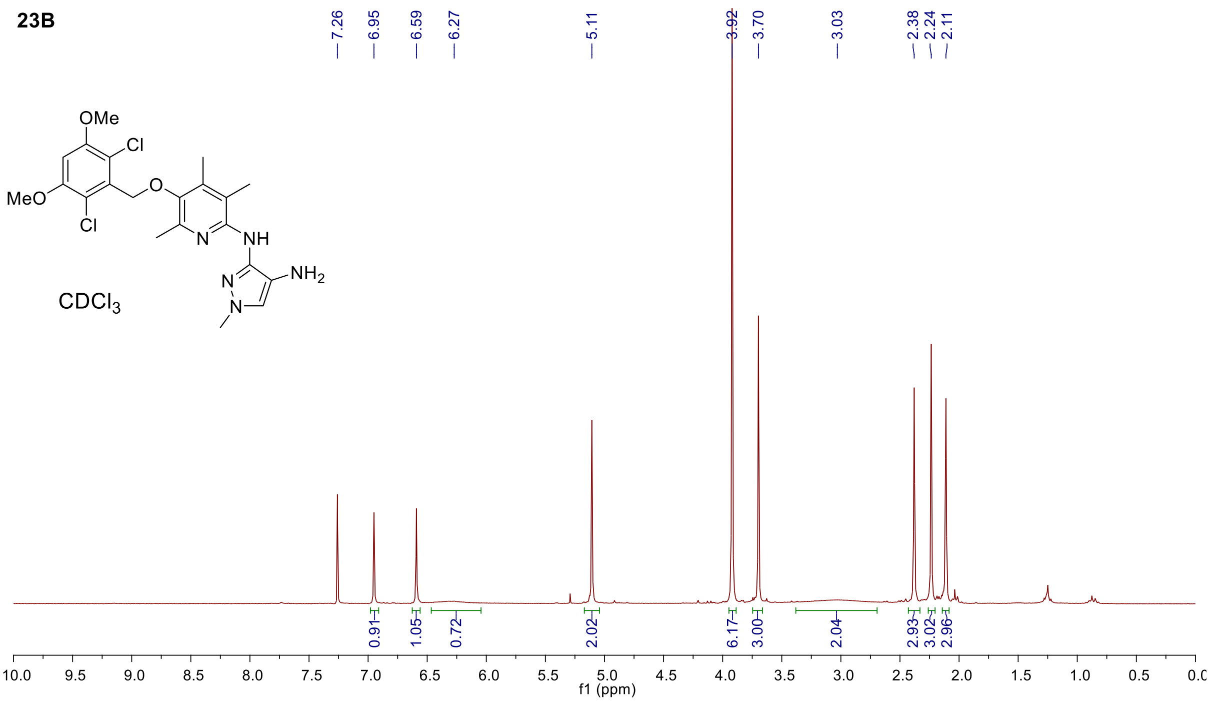
23B
OMe
Cl
MeO
Cl
O
N
NH
N
N
NH2
CDCl3
7.26
6.95
6.59
6.27
5.11
3.92
3.70
3.03
2.38
2.24
2.11
0.91
1.05
0.72
2.02
6.17
3.00
2.04
2.93
3.02
2.96
f1 (ppm)

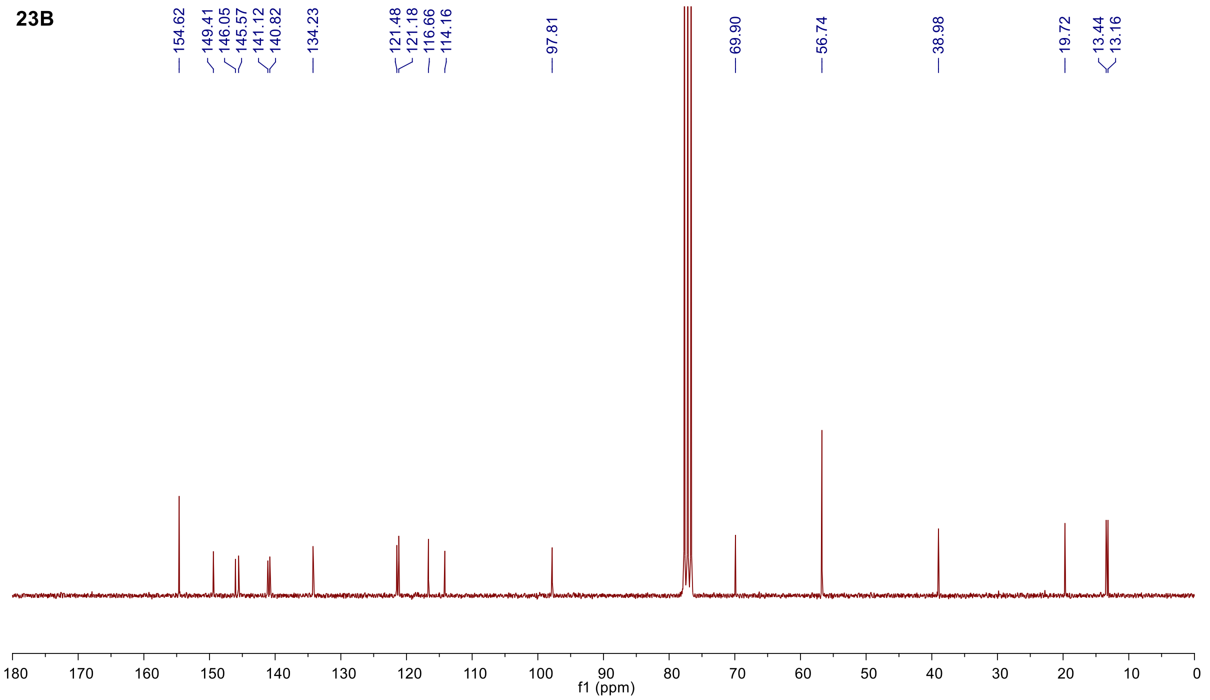
23B
154.62
149.41
146.05
145.57
141.12
140.82
134.23
121.48
121.18
116.66
114.16
97.81
69.90
56.74
38.98
19.72
13.44
13.16
f1 (ppm)

23C

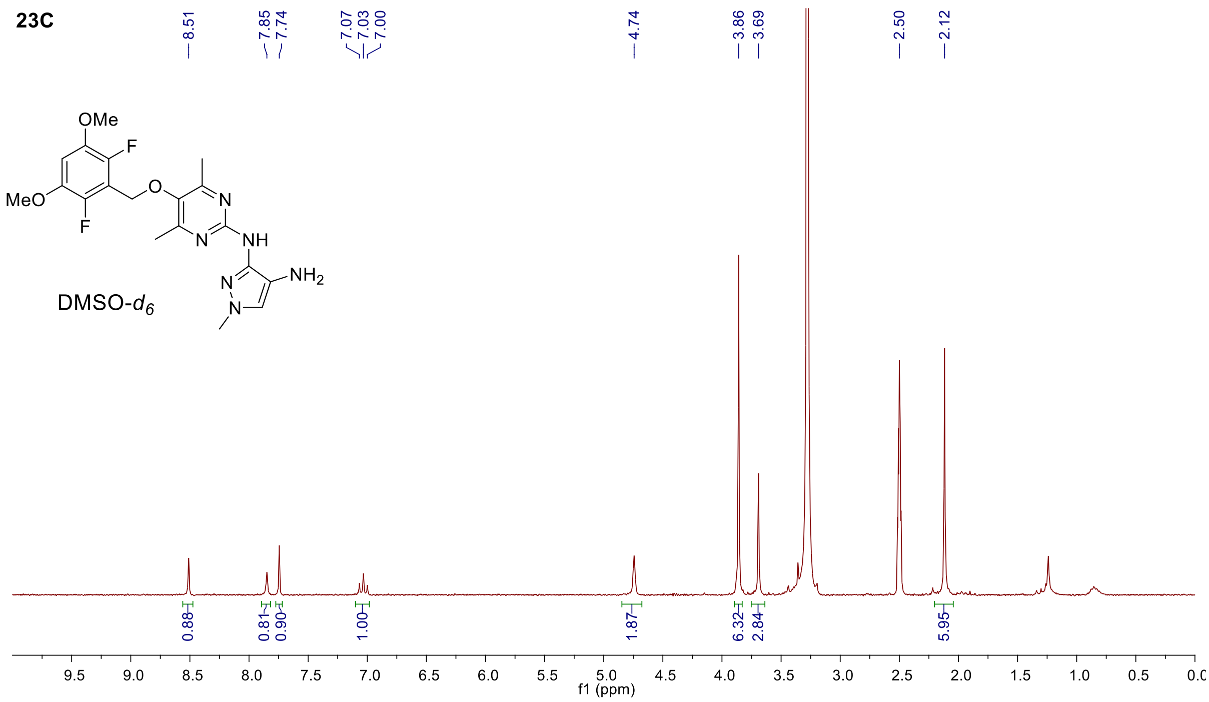

8.51
7.85
7.74
7.07
7.03
7.00
4.74
3.86
3.69
2.50
2.12
OMe
F
MeO
O
N
N
NH
F
N
N
NH2
DMSO-d6
0.88
0.81
0.90
1.00
1.87
6.32
2.84
5.95
9.5 9.0 8.5 8.0 7.5 7.0 6.5 6.0 5.5 5.0 4.5 4.0 3.5 3.0 2.5 2.0 1.5 1.0 0.5 0.0
f1 (ppm)

23C

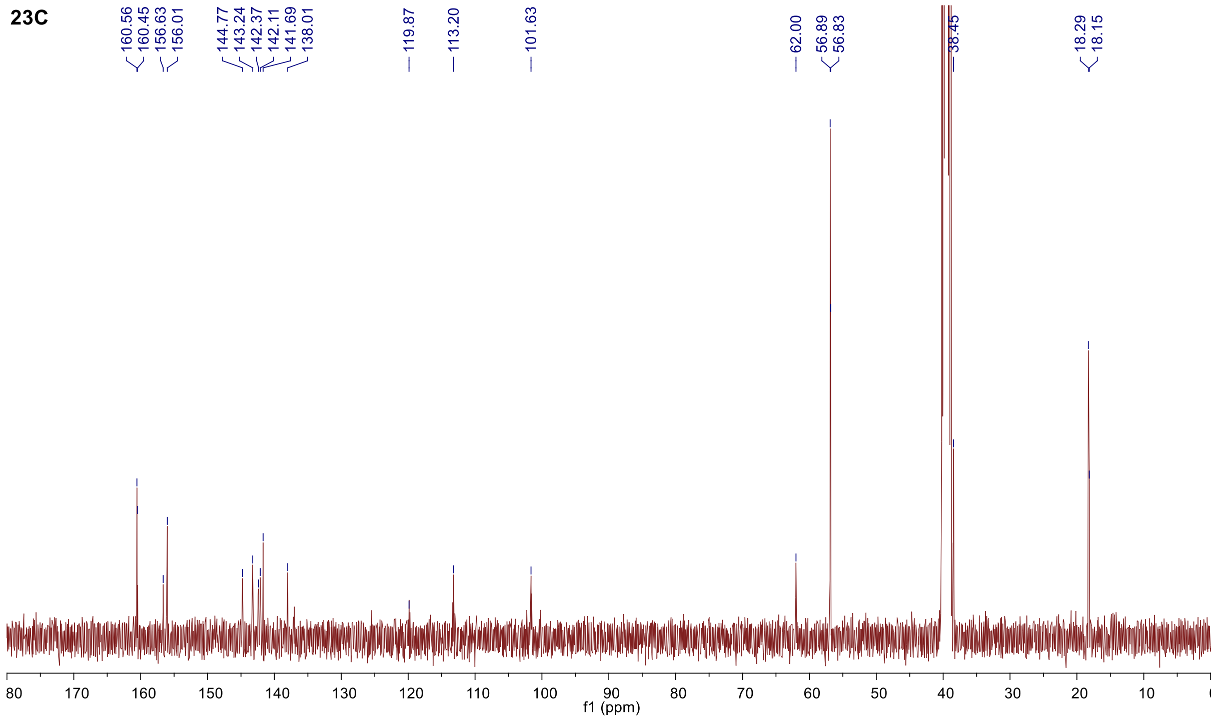

160.56
160.45
156.63
156.01
144.77
143.24
142.37
142.11
141.69
138.01
119.87
113.20
101.63
62.00
56.89
56.83
38.45
18.29
18.15
80 170 160 150 140 130 120 110 100 90 80 70 60 50 40 30 20 10
f1 (ppm)

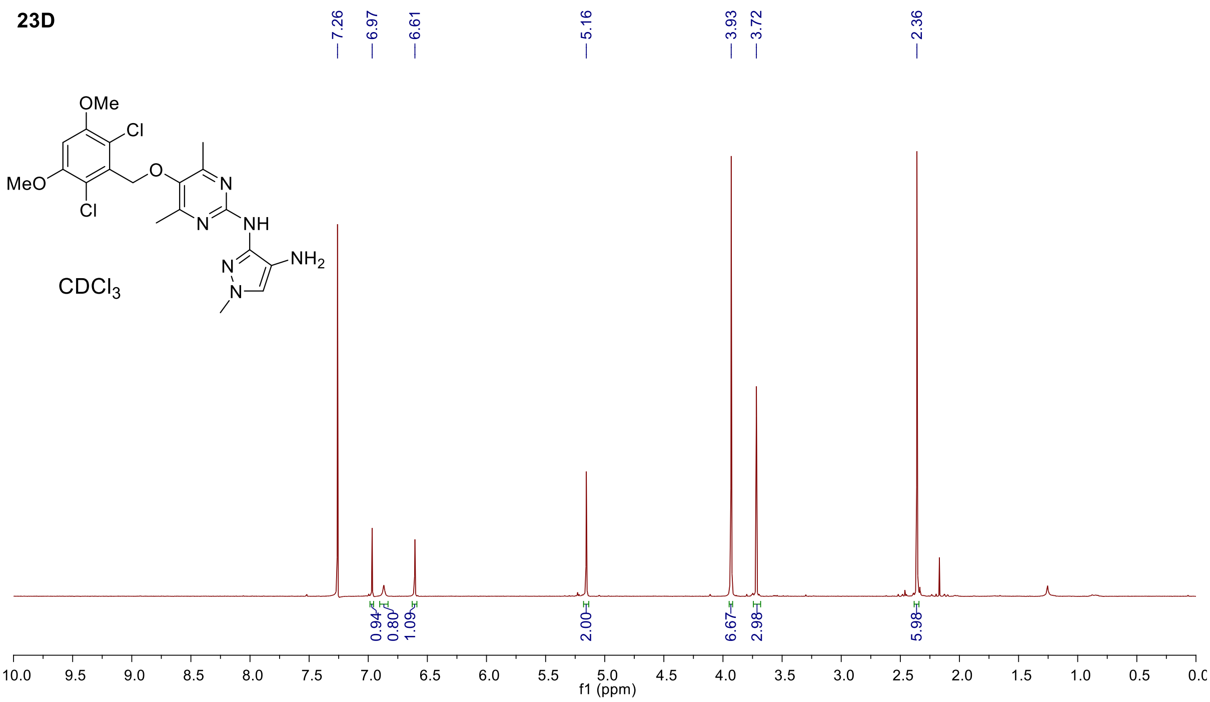
23D
7.26
6.97
6.61
5.16
3.93
3.72
2.36
OMe
Cl
MeO
Cl
O
N
N
NH
NH2
N
N
CDCl3
0.94
0.80
1.09
2.00
6.67
2.98
5.98
10.0 9.5 9.0 8.5 8.0 7.5 7.0 6.5 6.0 5.5 5.0 4.5 4.0 3.5 3.0 2.5 2.0 1.5 1.0 0.5 0.0
f1 (ppm)

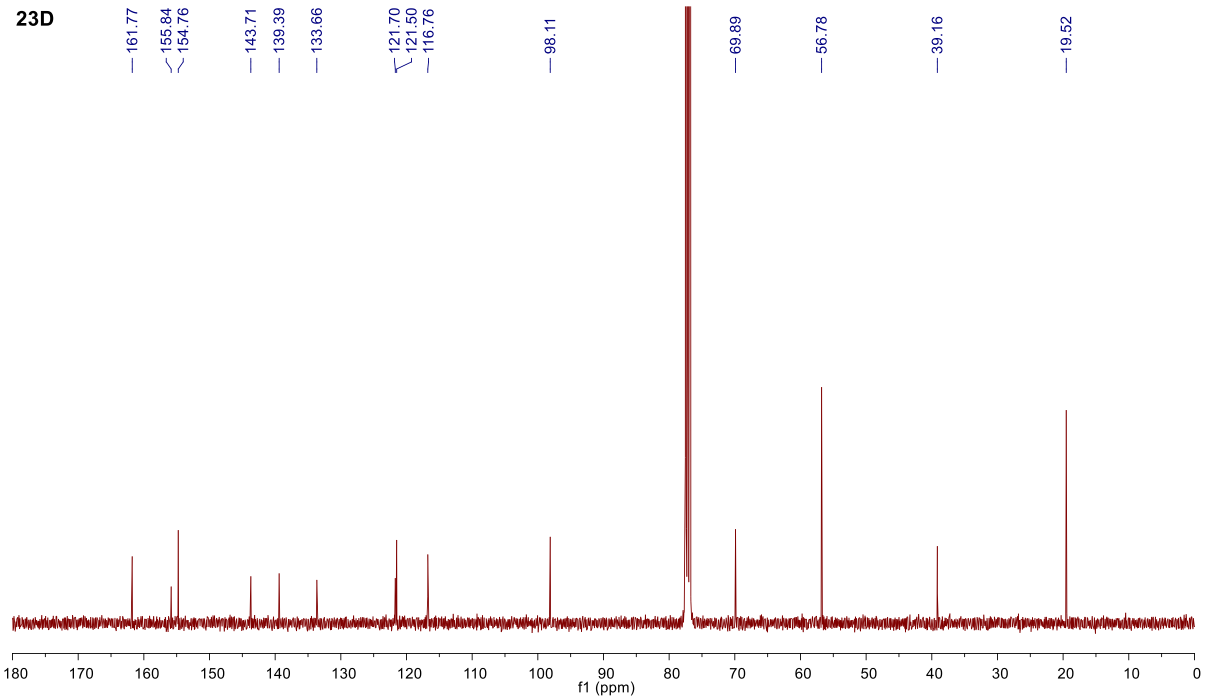
23D
161.77
155.84
154.76
143.71
139.39
133.66
121.70
121.50
116.76
98.11
69.89
56.78
39.16
19.52
180 170 160 150 140 130 120 110 100 90 80 70 60 50 40 30 20 10 0
f1 (ppm)

23E

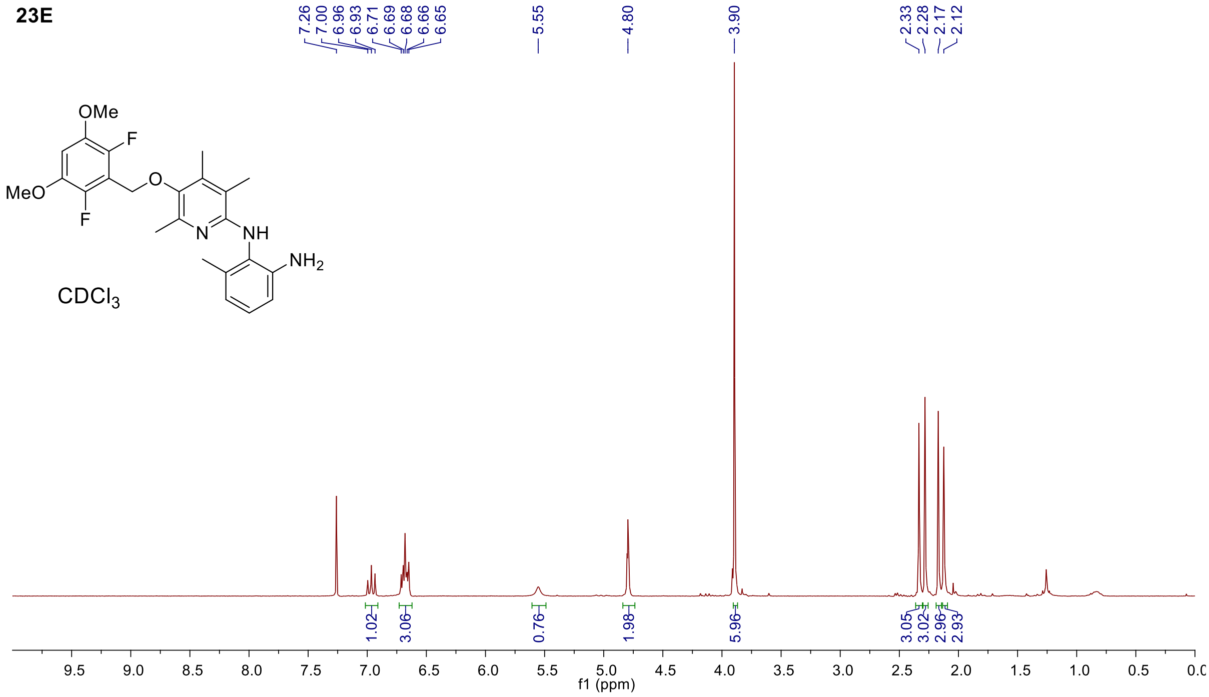

OMe
F
MeO
O
F
N
NH
NH2
CDCl3
7.26
7.00
6.96
6.93
6.71
6.69
6.68
6.66
6.65
5.55
4.80
3.90
2.33
2.28
2.17
2.12
1.02
3.06
0.76
1.98
5.96
3.05
3.02
2.96
2.93
9.5 9.0 8.5 8.0 7.5 7.0 6.5 6.0 5.5 5.0 4.5 4.0 3.5 3.0 2.5 2.0 1.5 1.0 0.5 0.0
f1 (ppm)

23E

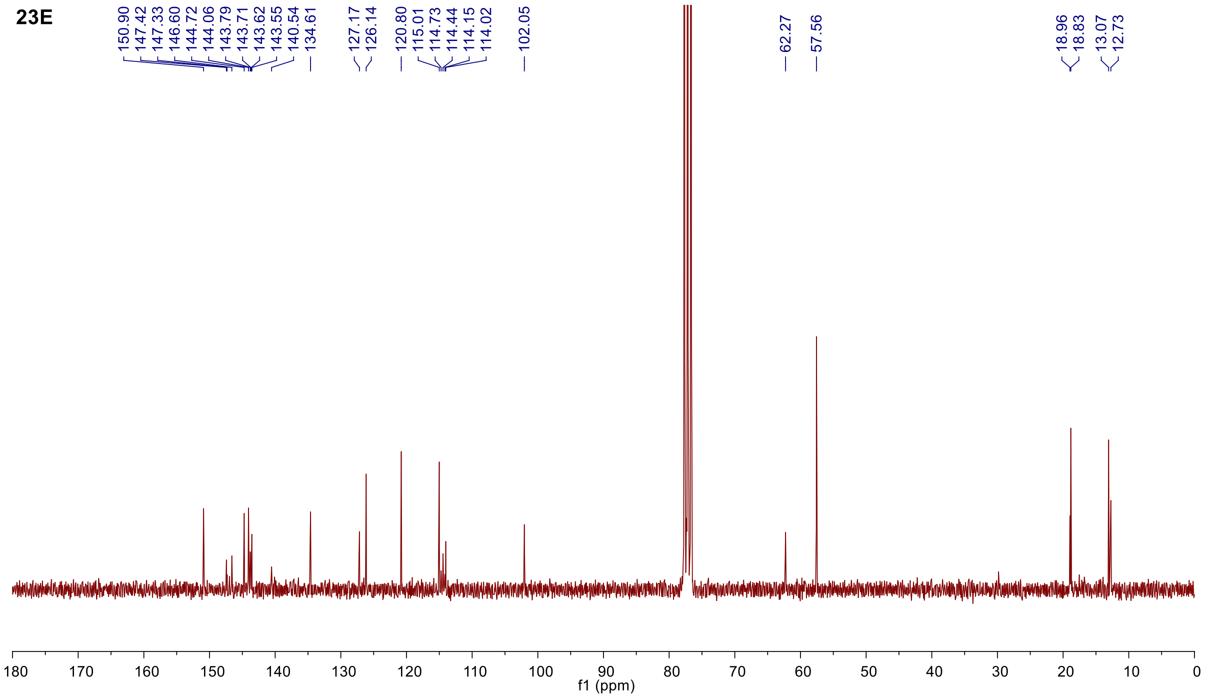

150.90
147.42
147.33
146.60
144.72
144.06
143.79
143.71
143.62
143.55
140.54
134.61
127.17
126.14
120.80
115.01
114.73
114.44
114.15
114.02
102.05
62.27
57.56
18.96
18.83
13.07
12.73
180 170 160 150 140 130 120 110 100 90 80 70 60 50 40 30 20 10 0
f1 (ppm)

23F

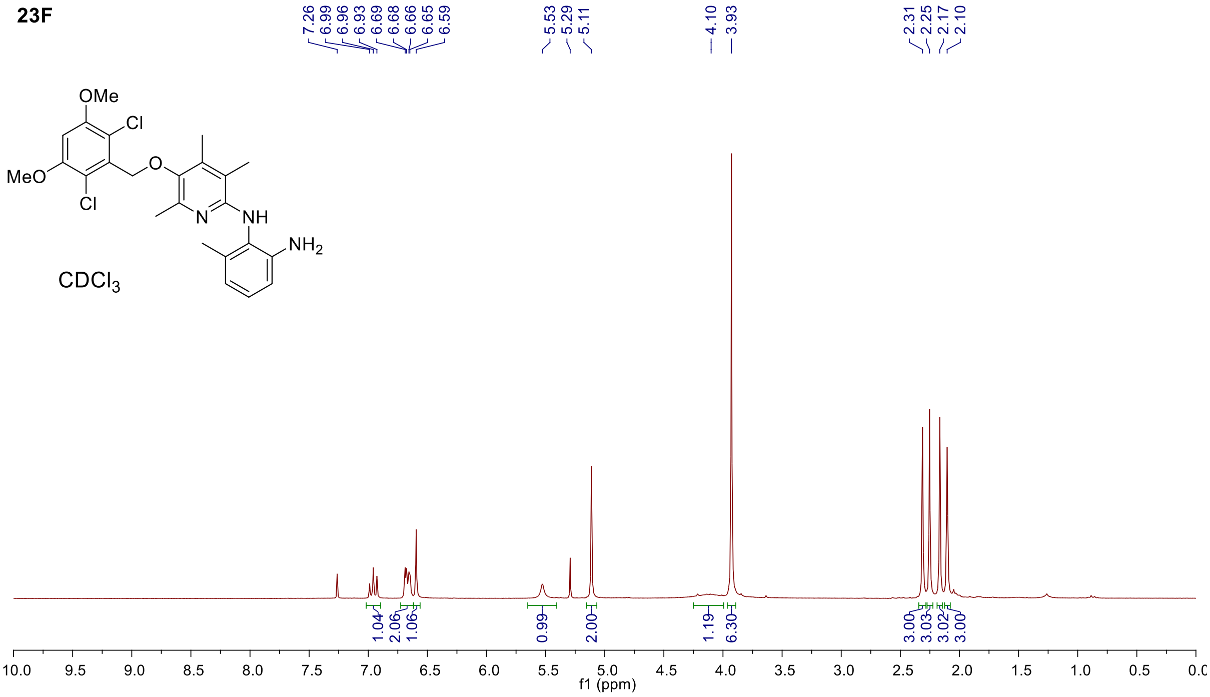

7.26
6.99
6.96
6.93
6.69
6.68
6.66
6.65
6.59
5.53
5.29
5.11
4.10
3.93
2.31
2.25
2.17
2.10
OMe
Cl
MeO
O
Cl
N
NH
NH2
CDCl3
1.04
2.06
1.06
0.99
2.00
1.19
6.30
3.00
3.03
3.02
3.00
f1 (ppm)

23F

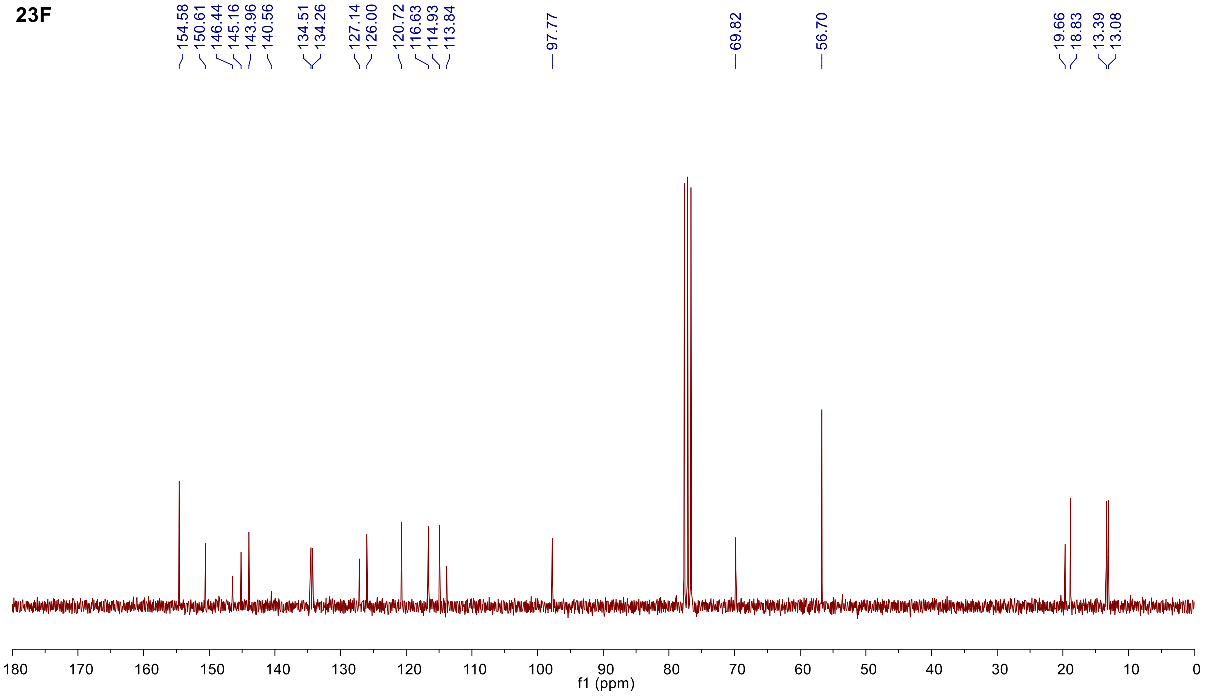

154.58
150.61
146.44
145.16
143.96
140.56
134.51
134.26
127.14
126.00
120.72
116.63
114.93
113.84
97.77
69.82
56.70
19.66
18.83
13.39
13.08
f1 (ppm)

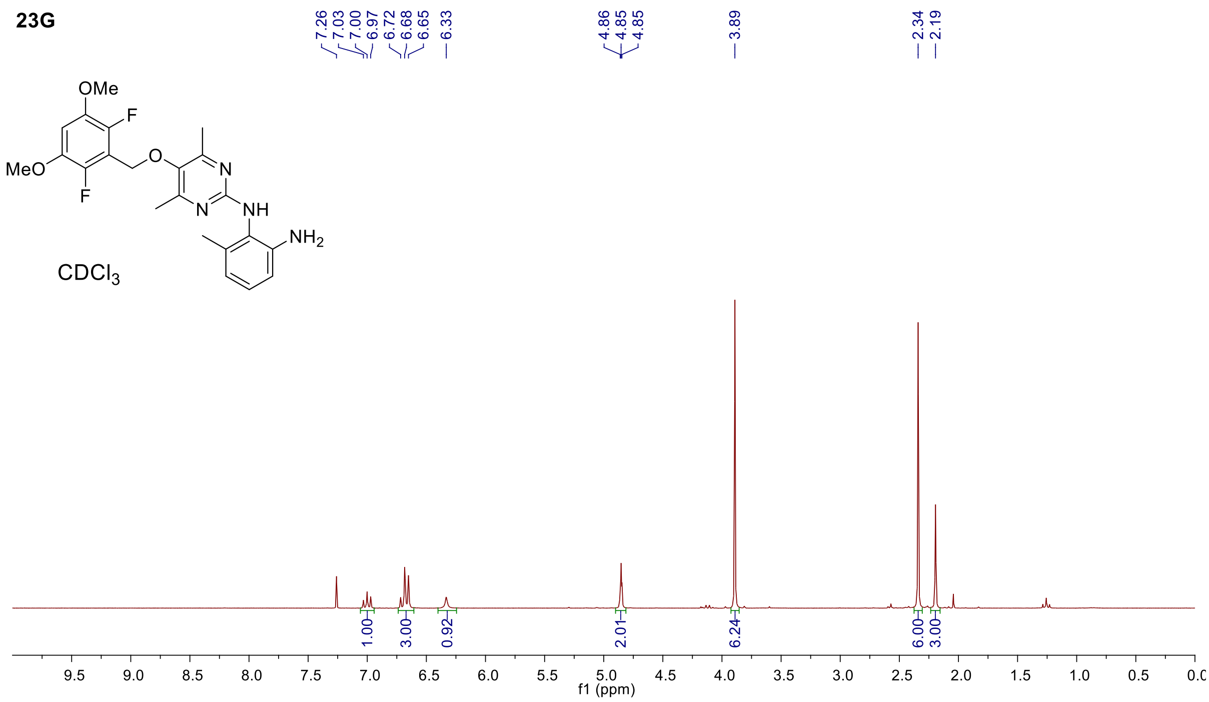
23G
OMe
F
MeO
F
O
N
N
NH
NH2
CDCl3
7.26
7.03
7.00
6.97
6.72
6.68
6.65
6.33
4.86
4.85
4.85
3.89
2.34
2.19
1.00
3.00
0.92
2.01
6.24
6.00
3.00
f1 (ppm)

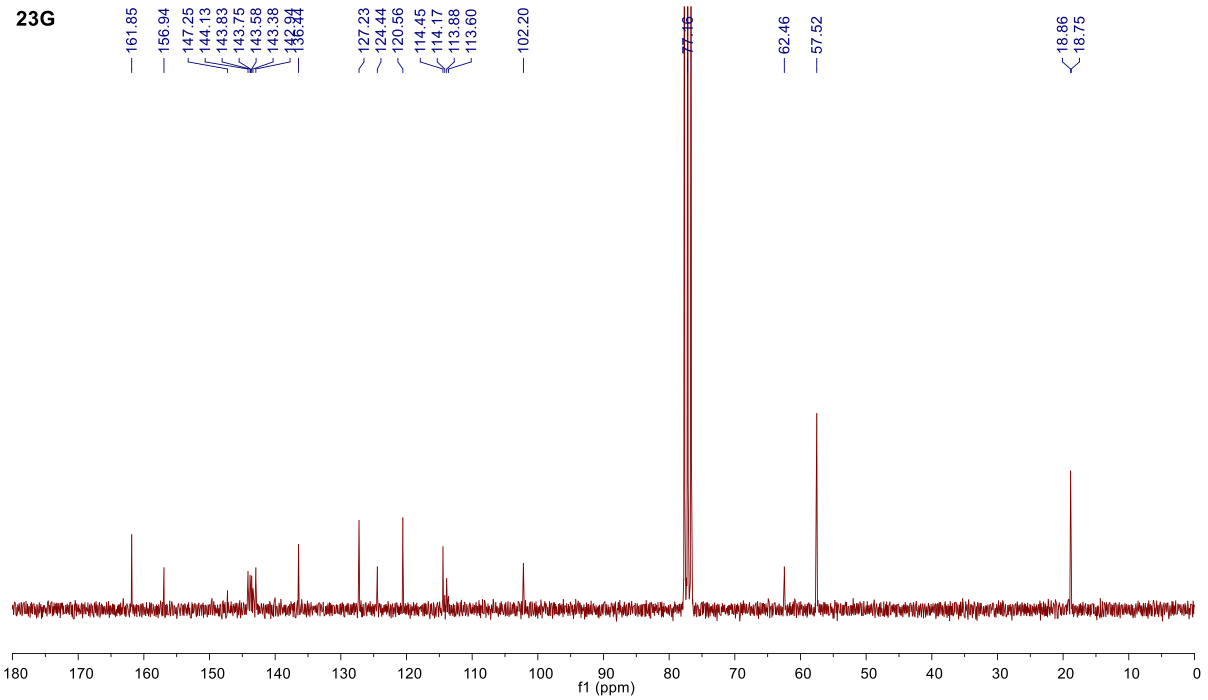
23G
161.85
156.94
147.25
144.13
143.83
143.75
143.58
143.38
127.23
124.44
120.56
114.45
114.17
113.88
113.60
102.20
62.46
57.52
18.86
18.75
f1 (ppm)

23H

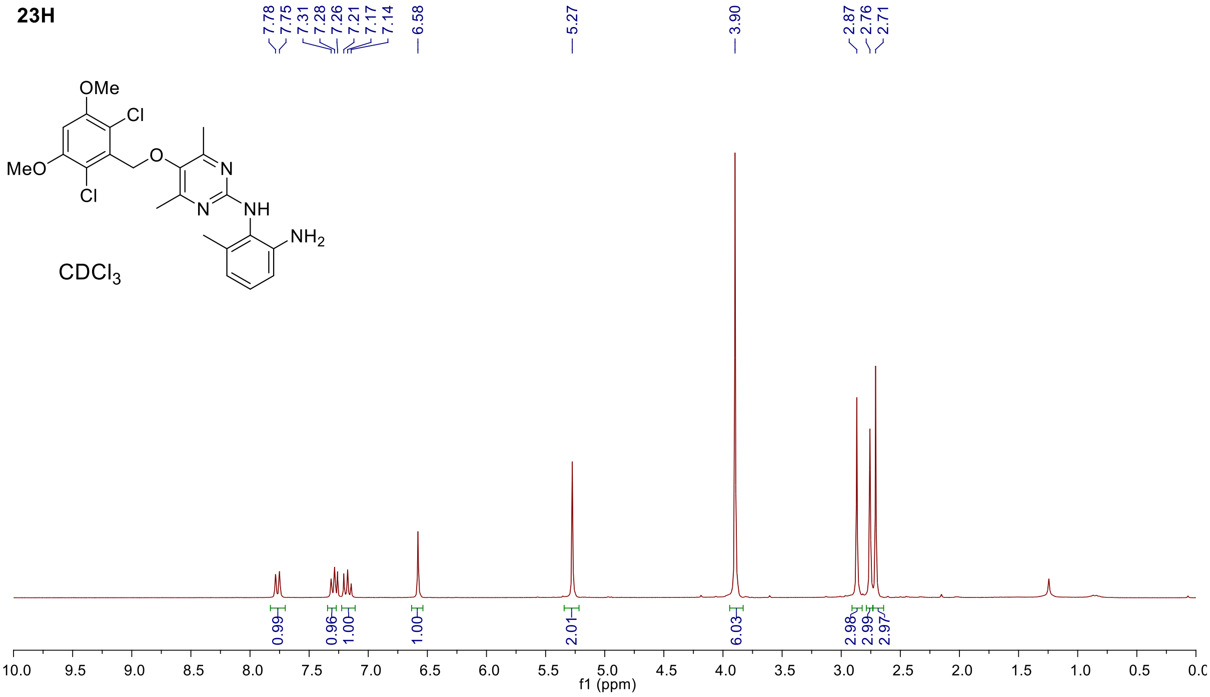
OMe
Cl
MeO
Cl
O
N
N
NH
NH2
CDCl3
7.78
7.75
7.31
7.28
7.26
7.21
7.17
7.14
6.58
5.27
3.90
2.87
2.76
2.71
0.99
0.96
1.00
1.00
2.01
6.03
2.98
2.99
2.97
f1 (ppm)

23H

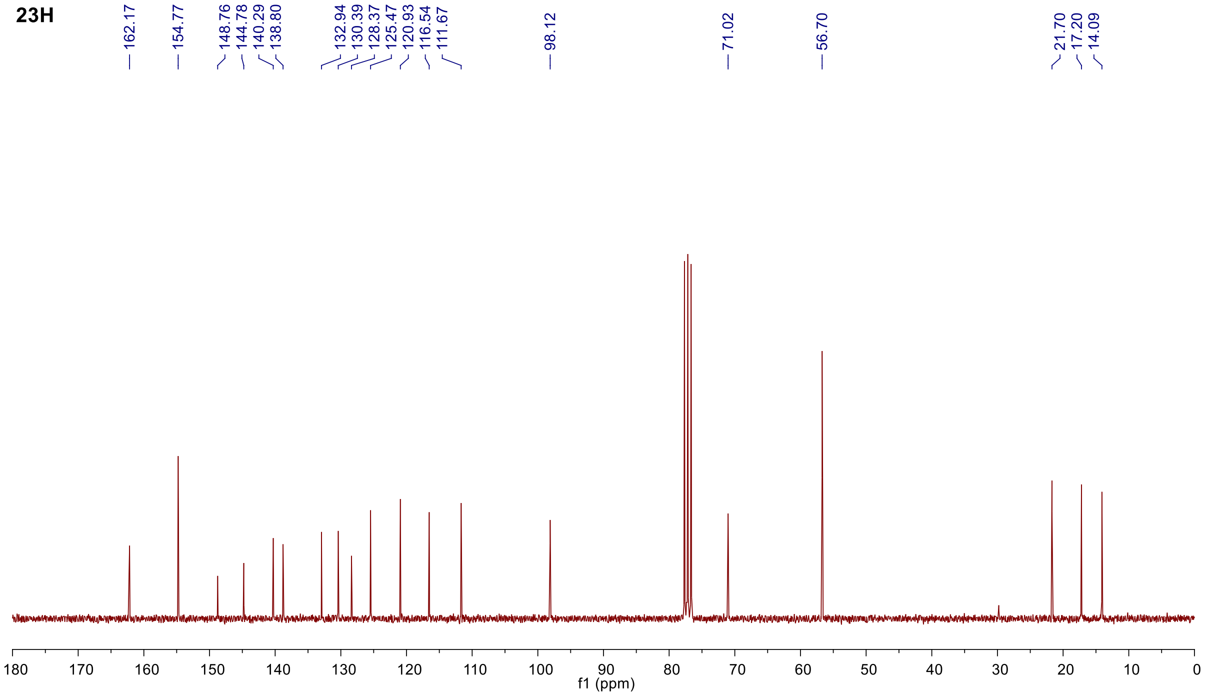
162.17
154.77
148.76
144.78
140.29
138.80
132.94
130.39
128.37
125.47
120.93
116.54
111.67
98.12
71.02
56.70
21.70
17.20
14.09
f1 (ppm)

23l

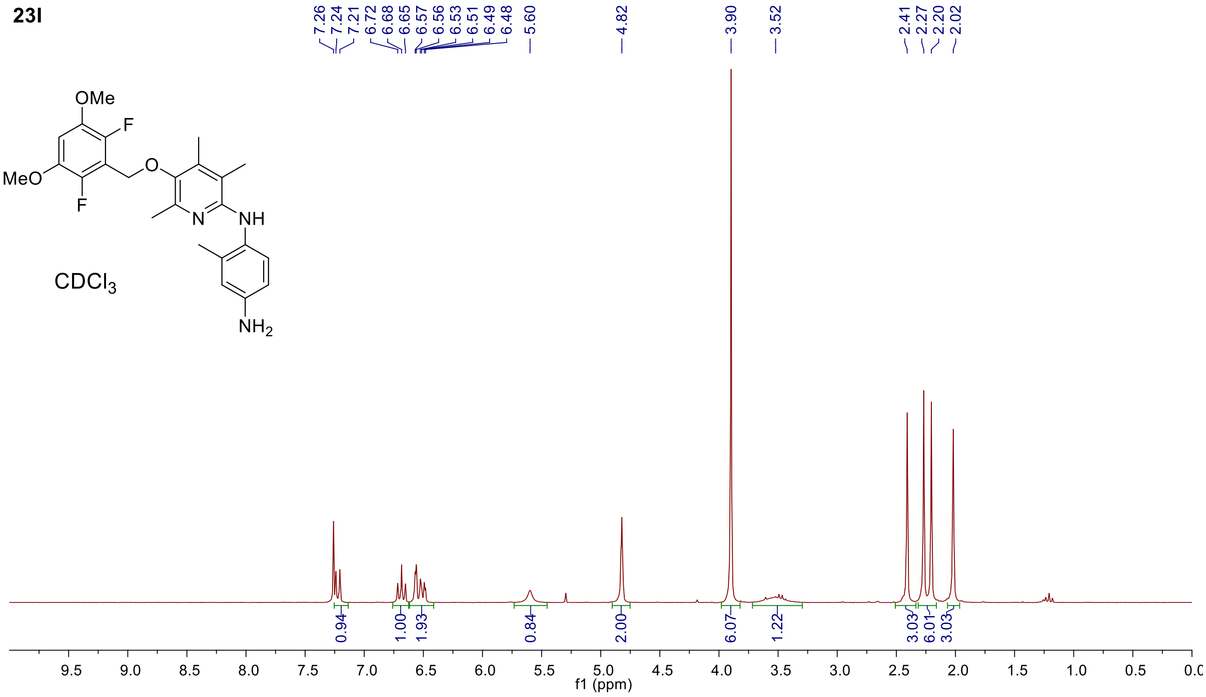
OMe
F
MeO
O
N
NH
F
NH2
CDCl3
7.26
7.24
7.21
6.72
6.68
6.65
6.57
6.56
6.53
6.51
6.49
6.48
5.60
4.82
3.90
3.52
2.41
2.27
2.20
2.02
0.94
1.00
1.93
0.84
2.00
6.07
1.22
3.03
6.01
3.03
9.5 9.0 8.5 8.0 7.5 7.0 6.5 6.0 5.5 5.0 4.5 4.0 3.5 3.0 2.5 2.0 1.5 1.0 0.5 0.0
f1 (ppm)

23l

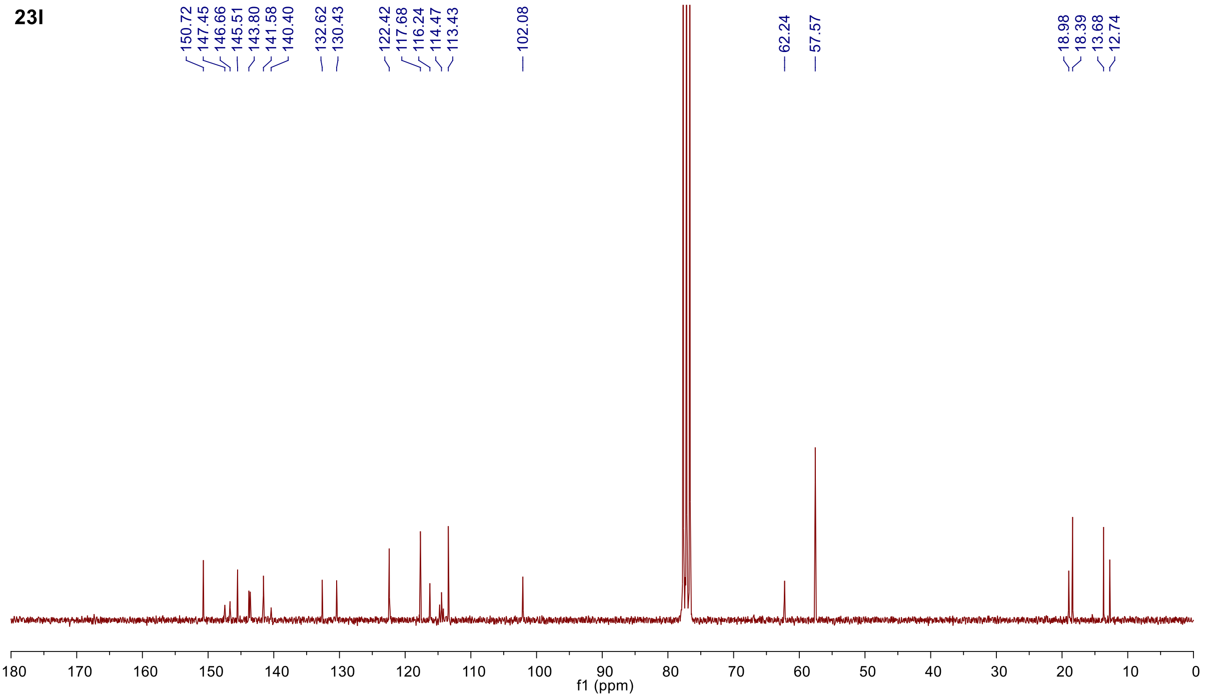
150.72
147.45
146.66
145.51
143.80
141.58
140.40
132.62
130.43
122.42
117.68
116.24
114.47
113.43
102.08
62.24
57.57
18.98
18.39
13.68
12.74
180 170 160 150 140 130 120 110 100 90 80 70 60 50 40 30 20 10 0
f1 (ppm)

23J

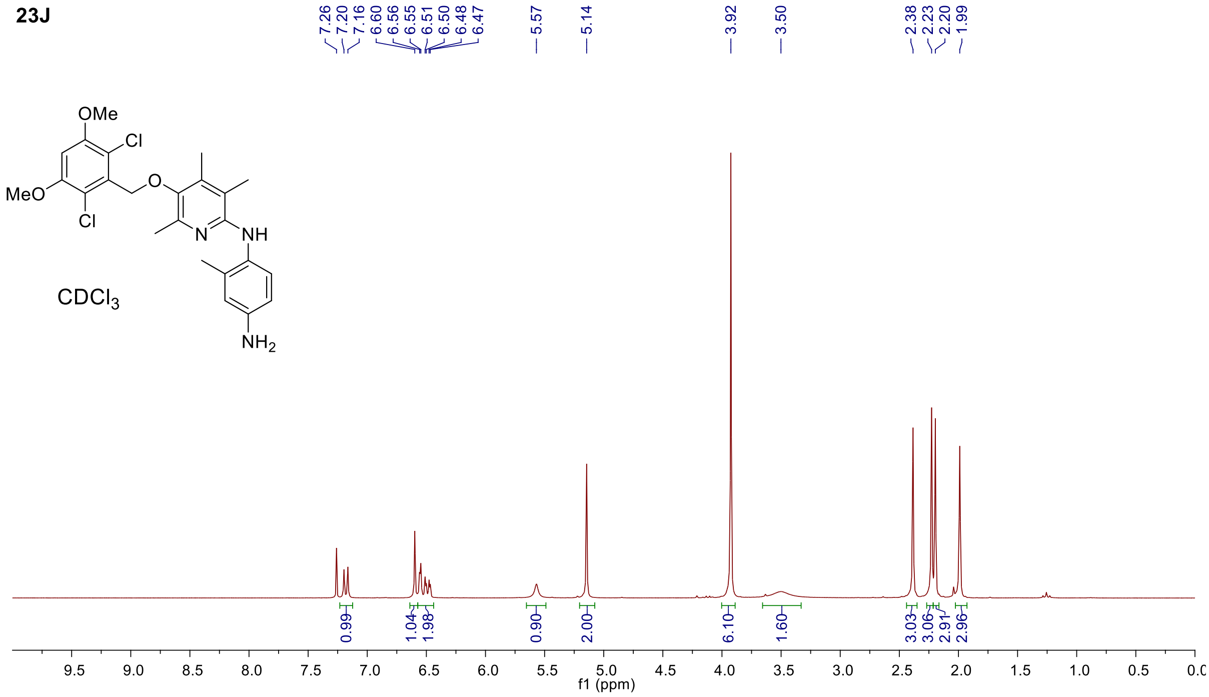
7.26
7.20
7.16
6.60
6.56
6.55
6.51
6.50
6.48
6.47
5.57
5.14
3.92
3.50
2.38
2.23
2.20
1.99
OMe
Cl
MeO
O
Cl
N
NH
NH2
CDCl3
0.99
1.04
1.98
0.90
2.00
6.10
1.60
3.03
3.06
2.91
2.96
9.5 9.0 8.5 8.0 7.5 7.0 6.5 6.0 5.5 5.0 4.5 4.0 3.5 3.0 2.5 2.0 1.5 1.0 0.5 0.0
f1 (ppm)

23J

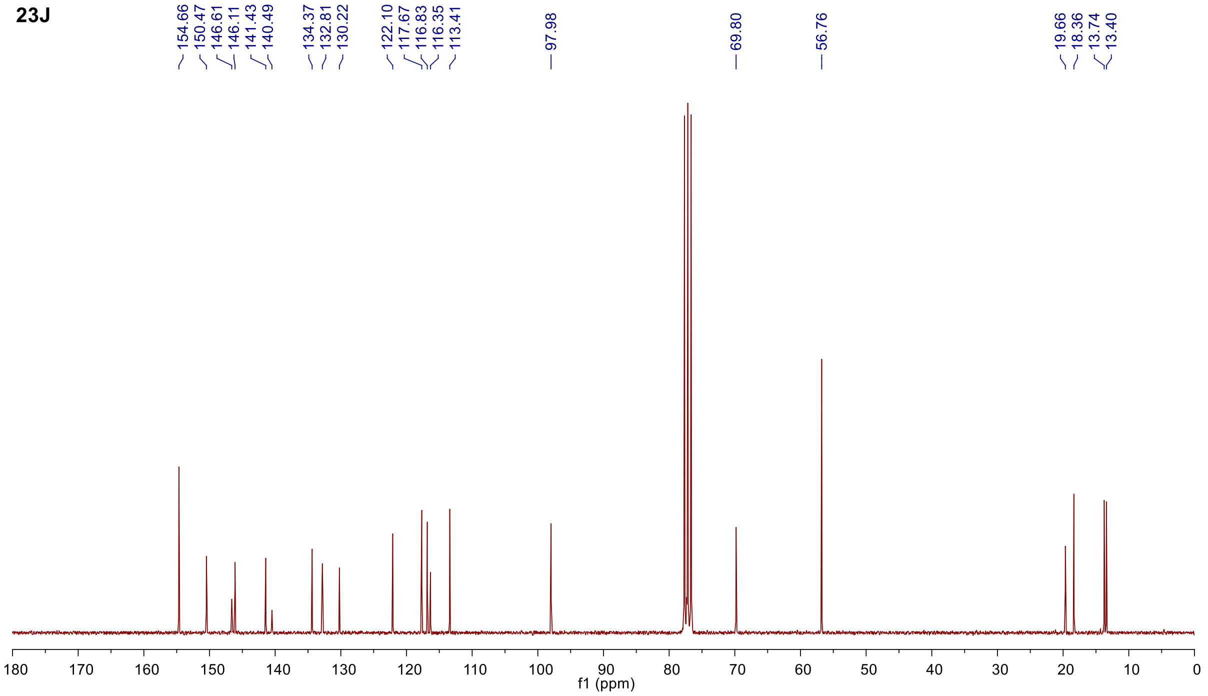
154.66
150.47
146.61
146.11
141.43
140.49
134.37
132.81
130.22
122.10
117.67
116.83
116.35
113.41
97.98
69.80
56.76
19.66
18.36
13.74
13.40
180 170 160 150 140 130 120 110 100 90 80 70 60 50 40 30 20 10 0
f1 (ppm)

23K

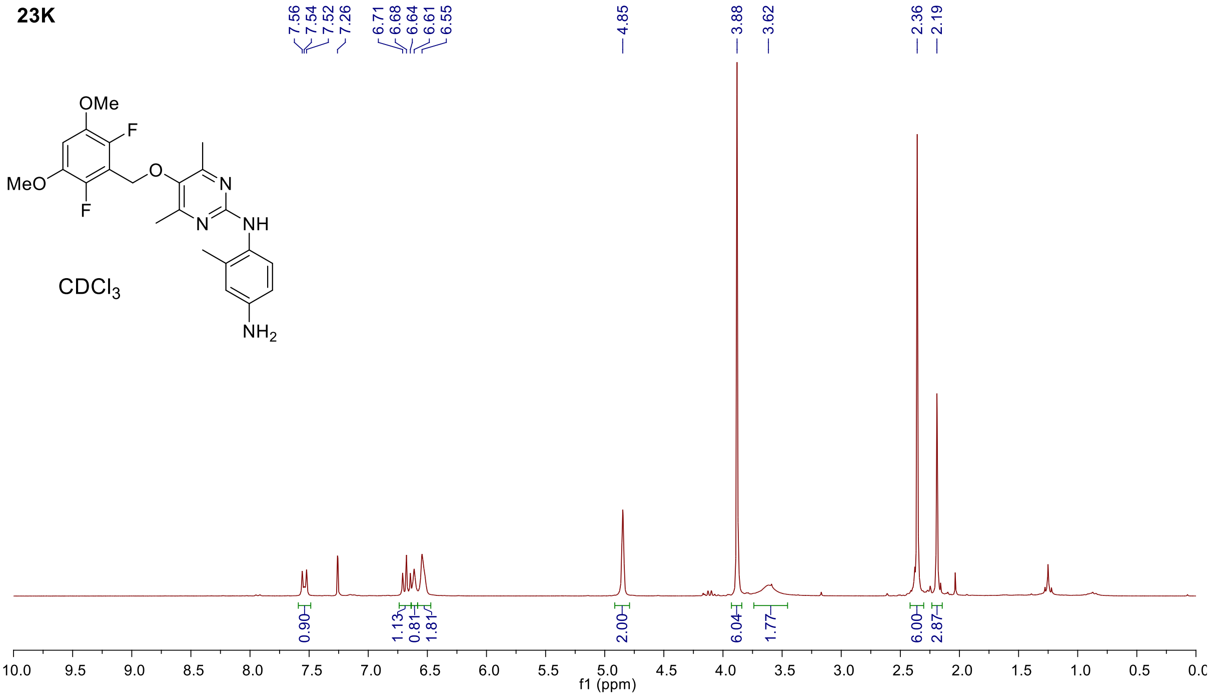
7.56
7.54
7.52
7.26
6.71
6.68
6.64
6.61
6.55
4.85
3.88
3.62
2.36
2.19
OMe
F
MeO
F
O
N
N
NH
NH2
CDCl3
0.90
1.13
0.81
1.81
2.00
6.04
1.77
6.00
2.87
f1 (ppm)

23K

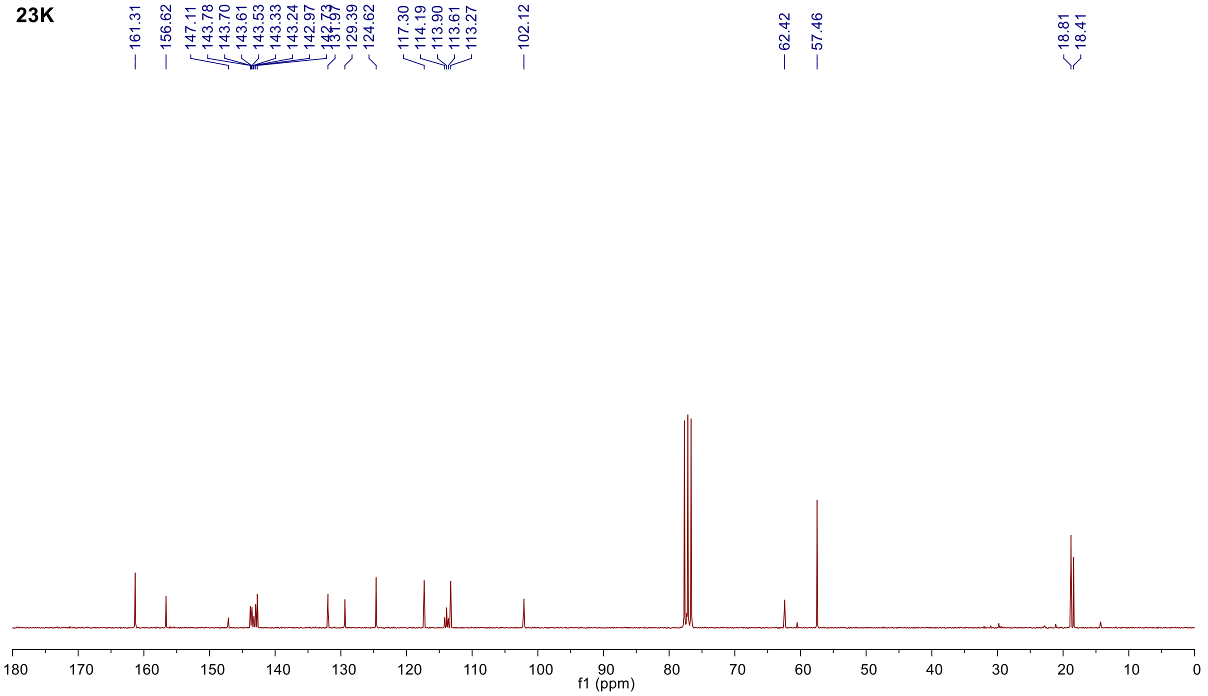
161.31
156.62
147.11
143.78
143.70
143.61
143.53
143.33
143.24
142.97
142.73
131.97
129.39
124.62
117.30
114.19
113.90
113.61
113.27
102.12
62.42
57.46
18.81
18.41
f1 (ppm)

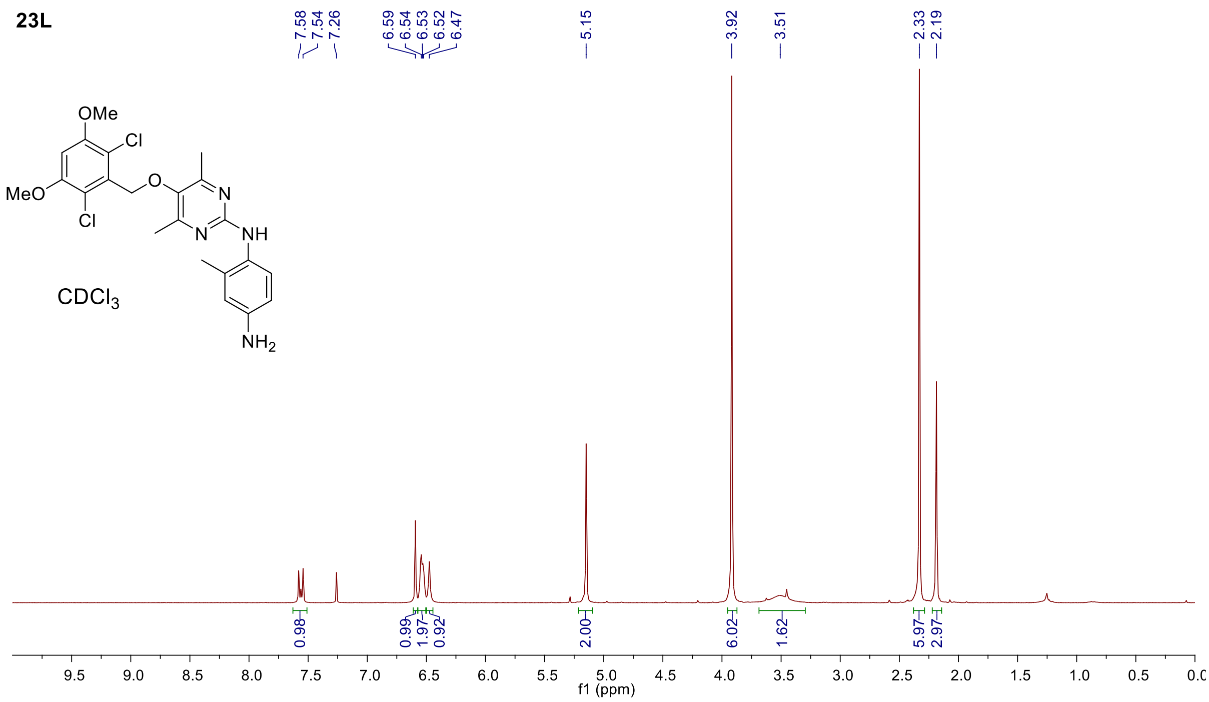
23L
7.58
7.54
7.26
6.59
6.54
6.53
6.52
6.47
5.15
3.92
3.51
2.33
2.19
OMe
Cl
MeO
O
N
Cl
N
NH
CDCl3
NH2
0.98
0.99
1.97
0.92
2.00
6.02
1.62
5.97
2.97
f1 (ppm)

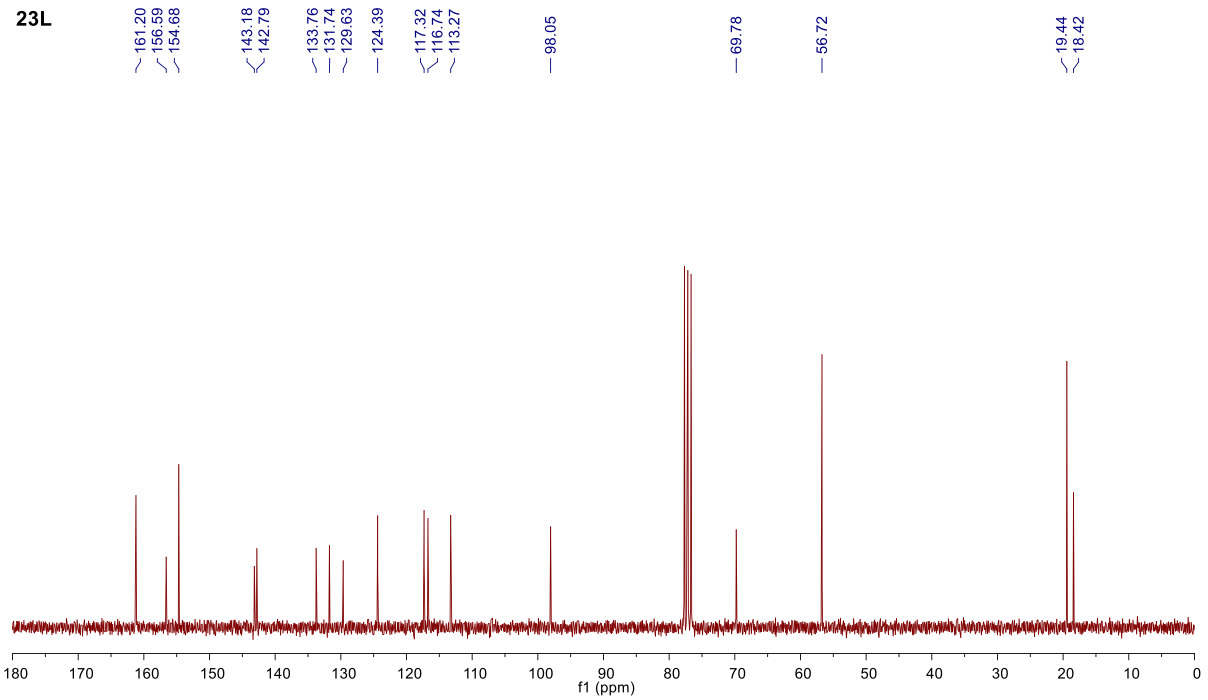
23L
161.20
156.59
154.68
143.18
142.79
133.76
131.74
129.63
124.39
117.32
116.74
113.27
98.05
69.78
56.72
19.44
18.42
f1 (ppm)

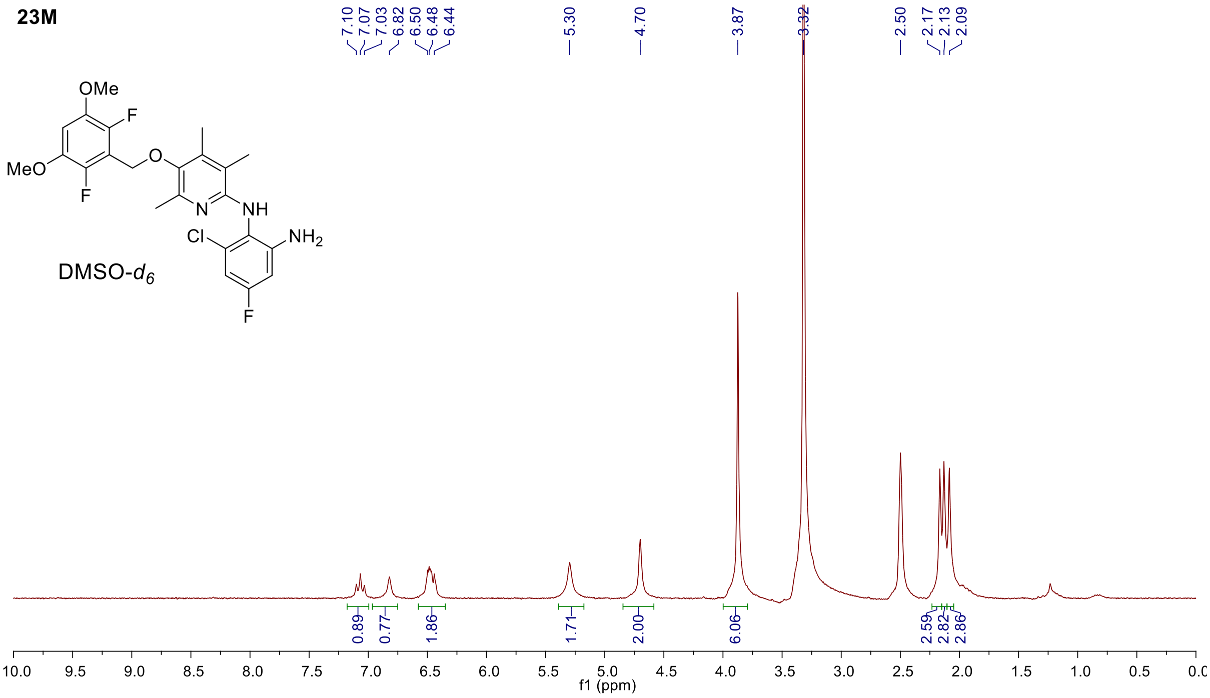
23M
7.10
7.07
7.03
6.82
6.50
6.48
6.44
5.30
4.70
3.87
3.32
2.50
2.17
2.13
2.09
OMe
F
MeO
F
O
N
NH
Cl
NH2
F
DMSO-d6
0.89
0.77
1.86
1.71
2.00
6.06
2.59
2.82
2.86
f1 (ppm)

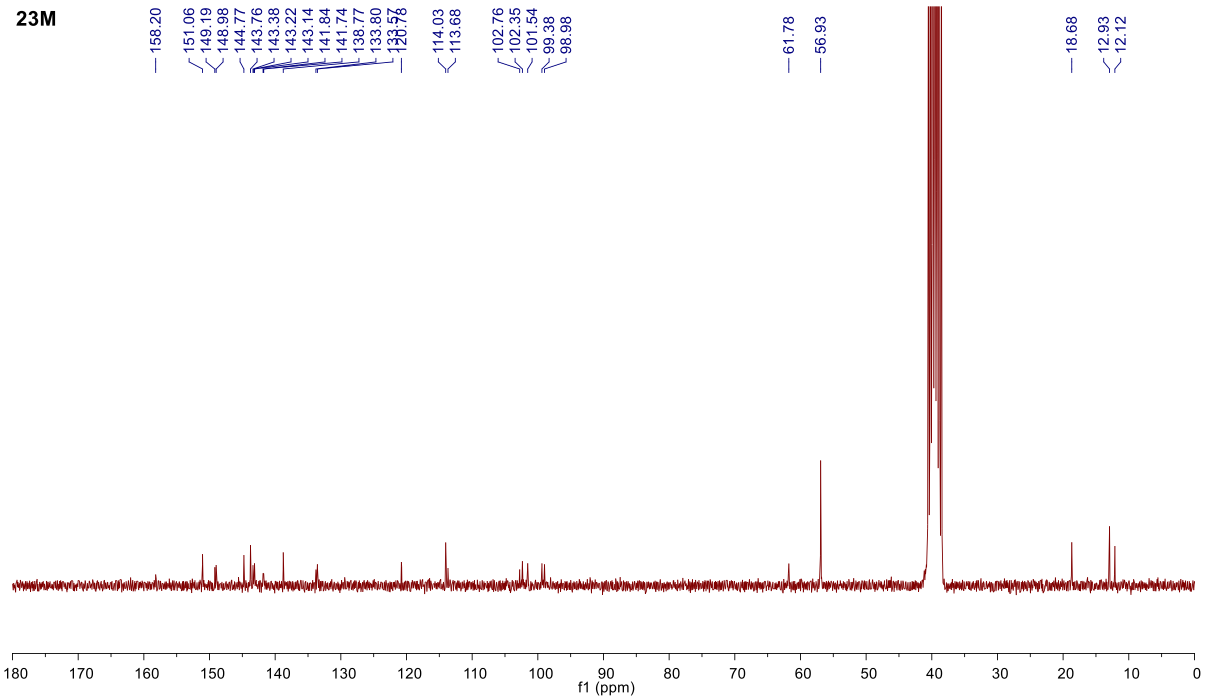
23M
158.20
151.06
149.19
148.98
144.77
143.76
143.38
143.22
143.14
141.84
141.74
138.77
133.80
120.78
114.03
113.68
102.76
102.35
101.54
99.38
98.98
61.78
56.93
18.68
12.93
12.12
f1 (ppm)

23N

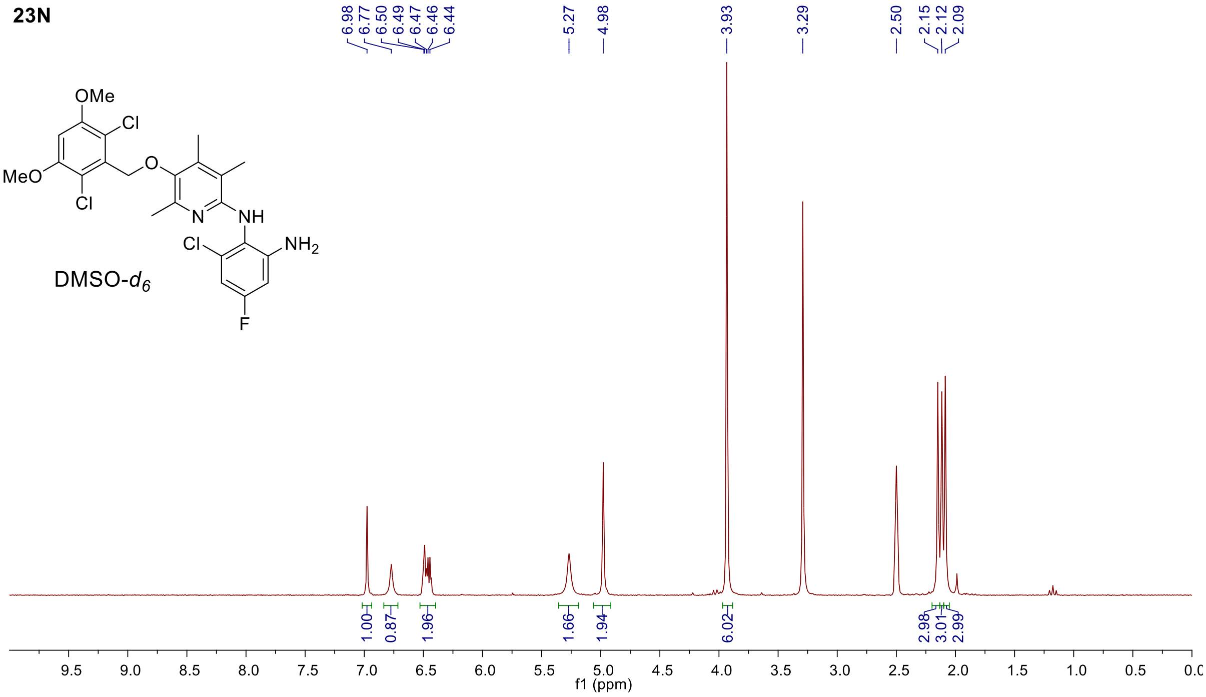
6.98
6.77
6.50
6.49
6.47
6.46
6.44
5.27
4.98
3.93
3.29
2.50
2.15
2.12
2.09
OMe
Cl
MeO
O
Cl
N
NH
Cl
NH2
F
DMSO-d6
1.00
0.87
1.96
1.66
1.94
6.02
2.98
3.01
2.99
9.5
9.0
8.5
8.0
7.5
7.0
6.5
6.0
5.5
5.0
4.5
4.0
3.5
3.0
2.5
2.0
1.5
1.0
0.5
0.0
f1 (ppm)

23N

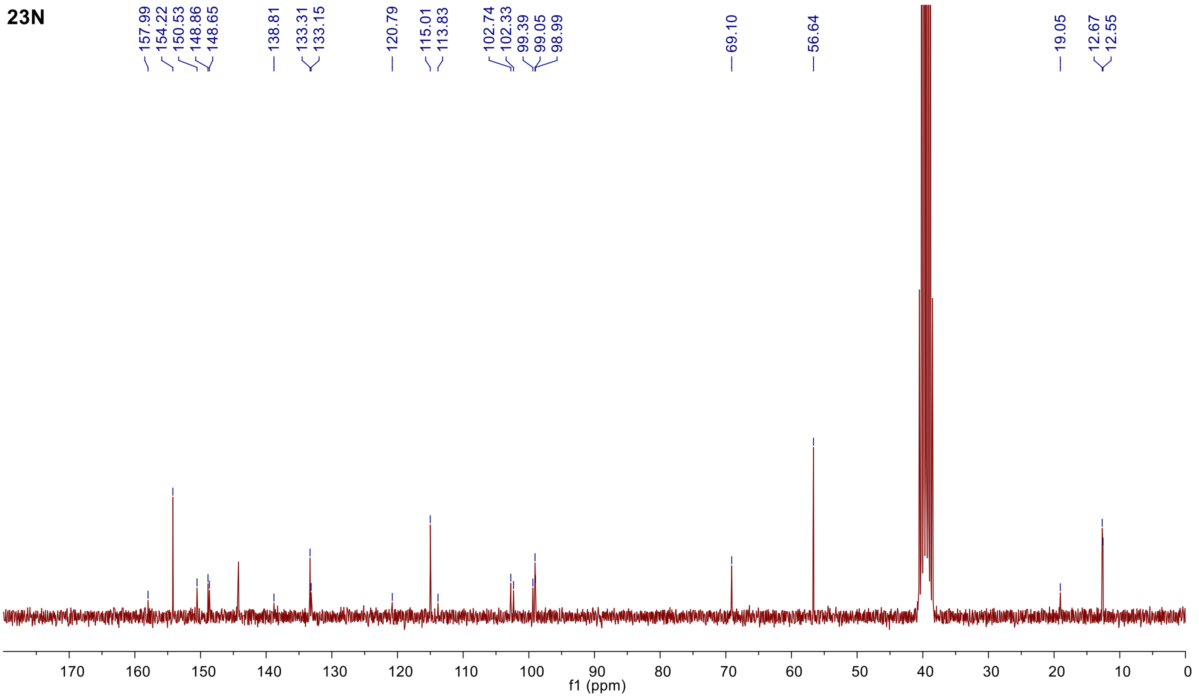
157.99
154.22
150.53
148.86
148.65
138.81
133.31
133.15
120.79
115.01
113.83
102.74
102.33
99.39
99.05
98.99
69.10
56.64
19.05
12.67
12.55
170
160
150
140
130
120
110
100
90
80
70
60
50
40
30
20
10
0
f1 (ppm)

23O

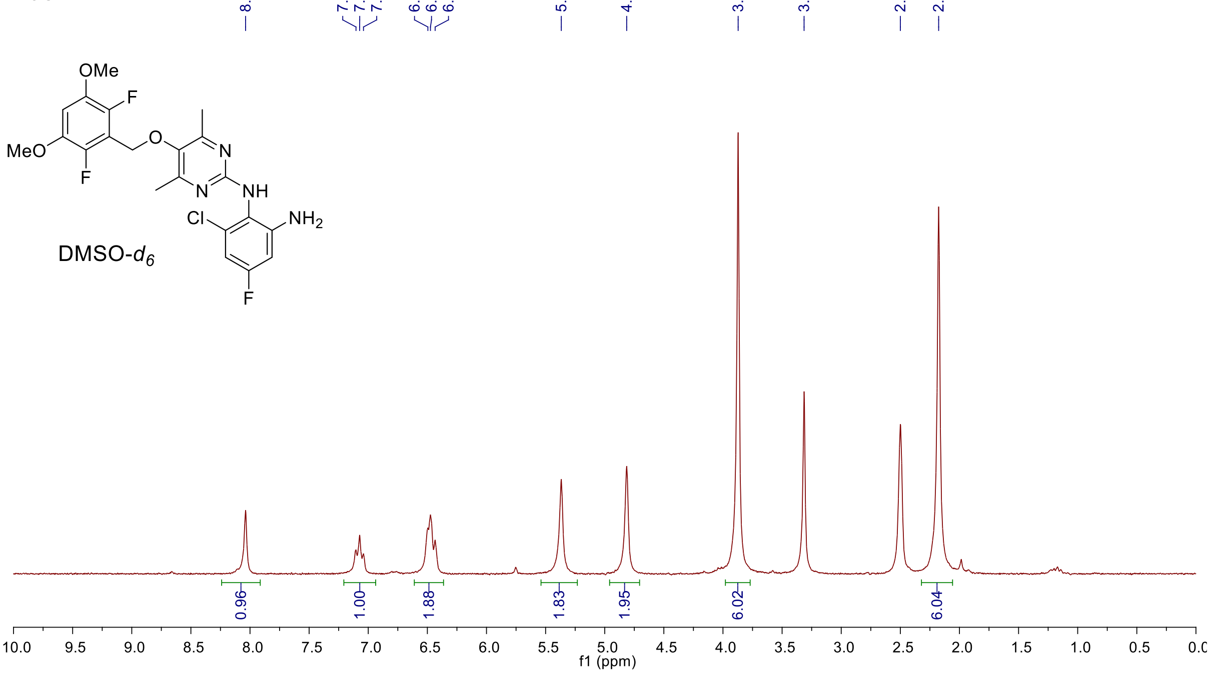
8.04
7.10
7.07
7.04
6.50
6.47
6.43
5.37
4.81
3.87
3.31
2.50
2.18
OMe
F
MeO
F
O
N
N
NH
Cl
NH2
F
DMSO-d6
0.96
1.00
1.88
1.83
1.95
6.02
6.04
10.0 9.5 9.0 8.5 8.0 7.5 7.0 6.5 6.0 5.5 5.0 4.5 4.0 3.5 3.0 2.5 2.0 1.5 1.0 0.5 0.0
f1 (ppm)

23O

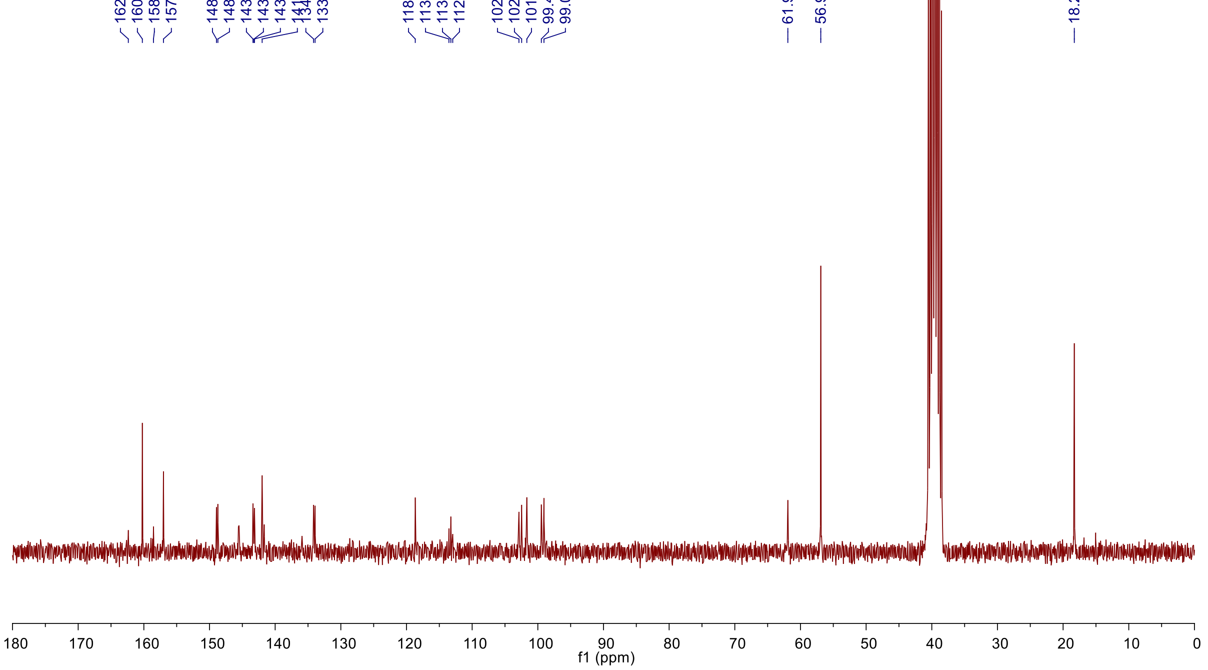
162.36
160.23
158.53
157.01
148.94
148.73
143.38
143.22
143.15
141.98
134.15
133.92
118.66
113.54
113.25
112.96
102.88
102.46
101.67
99.46
99.07
61.92
56.91
18.29
180 170 160 150 140 130 120 110 100 90 80 70 60 50 40 30 20 10 0
f1 (ppm)

23P

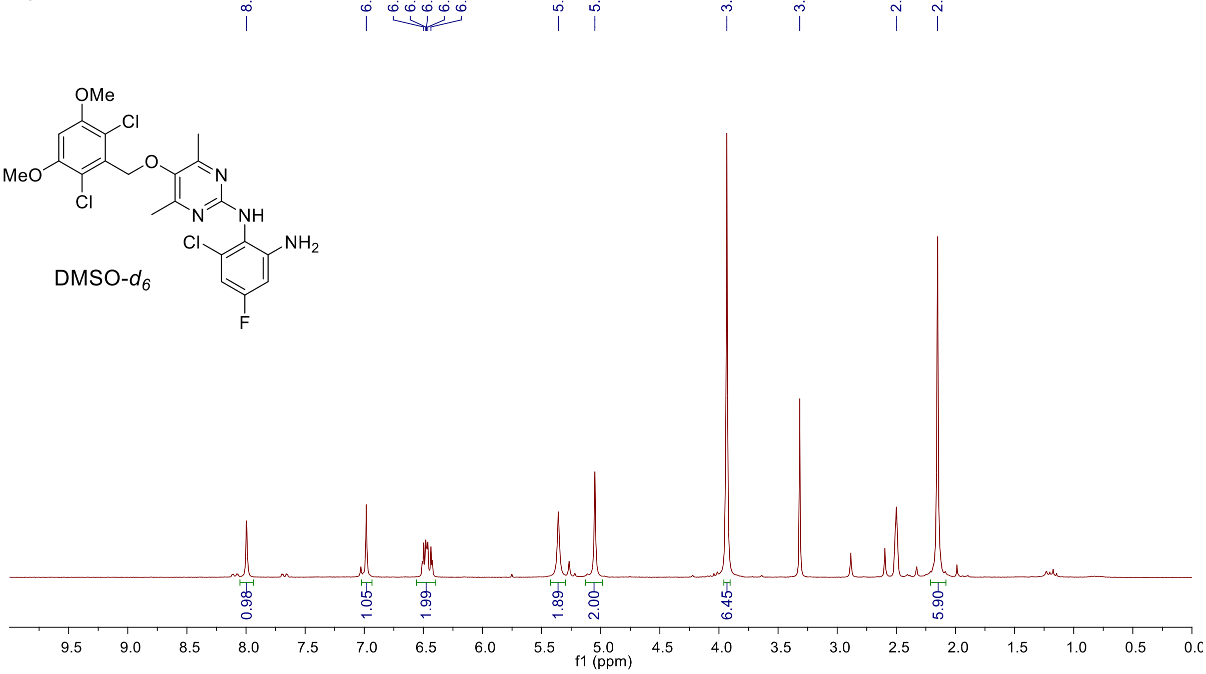
8.00
6.98
6.50
6.48
6.47
6.46
6.44
5.36
5.05
3.93
3.32
2.50
2.15
OMe
Cl
MeO
O
N
N
NH
Cl
NH2
F
Cl
DMSO-d6
0.98
1.05
1.99
1.89
2.00
6.45
5.90
9.5 9.0 8.5 8.0 7.5 7.0 6.5 6.0 5.5 5.0 4.5 4.0 3.5 3.0 2.5 2.0 1.5 1.0 0.5 0.0
f1 (ppm)

23P

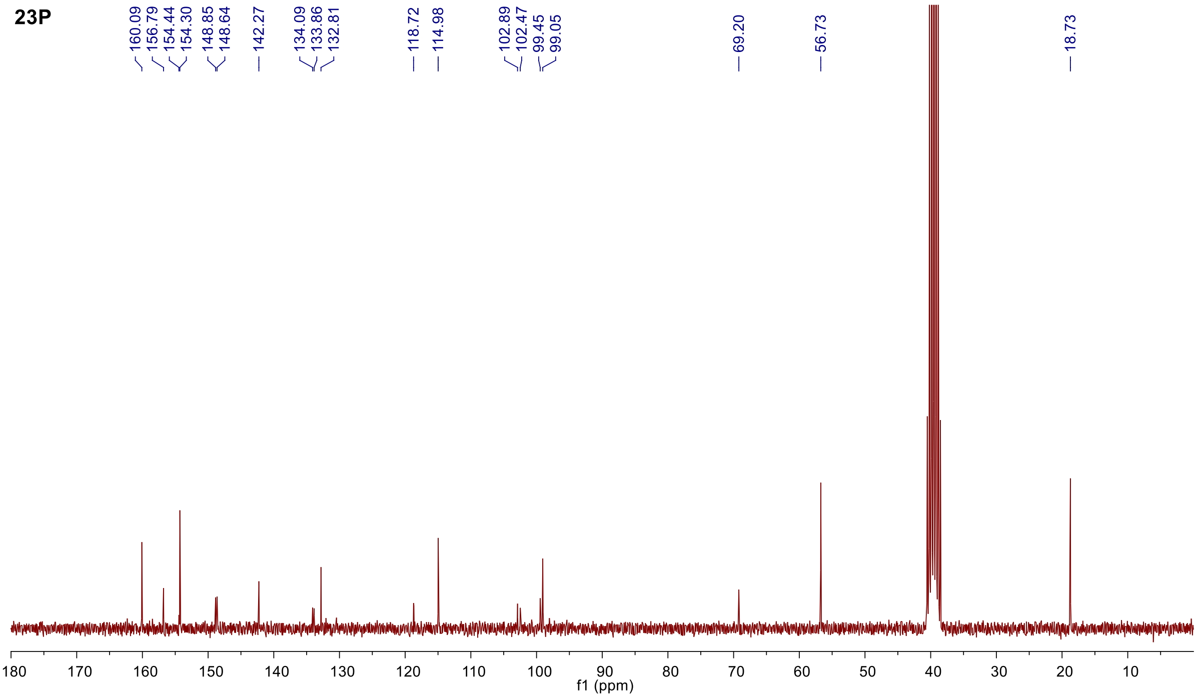
160.09
156.79
154.44
154.30
148.85
148.64
142.27
134.09
133.86
132.81
118.72
114.98
102.89
102.47
99.45
99.05
69.20
56.73
18.73
180 170 160 150 140 130 120 110 100 90 80 70 60 50 40 30 20 10
f1 (ppm)

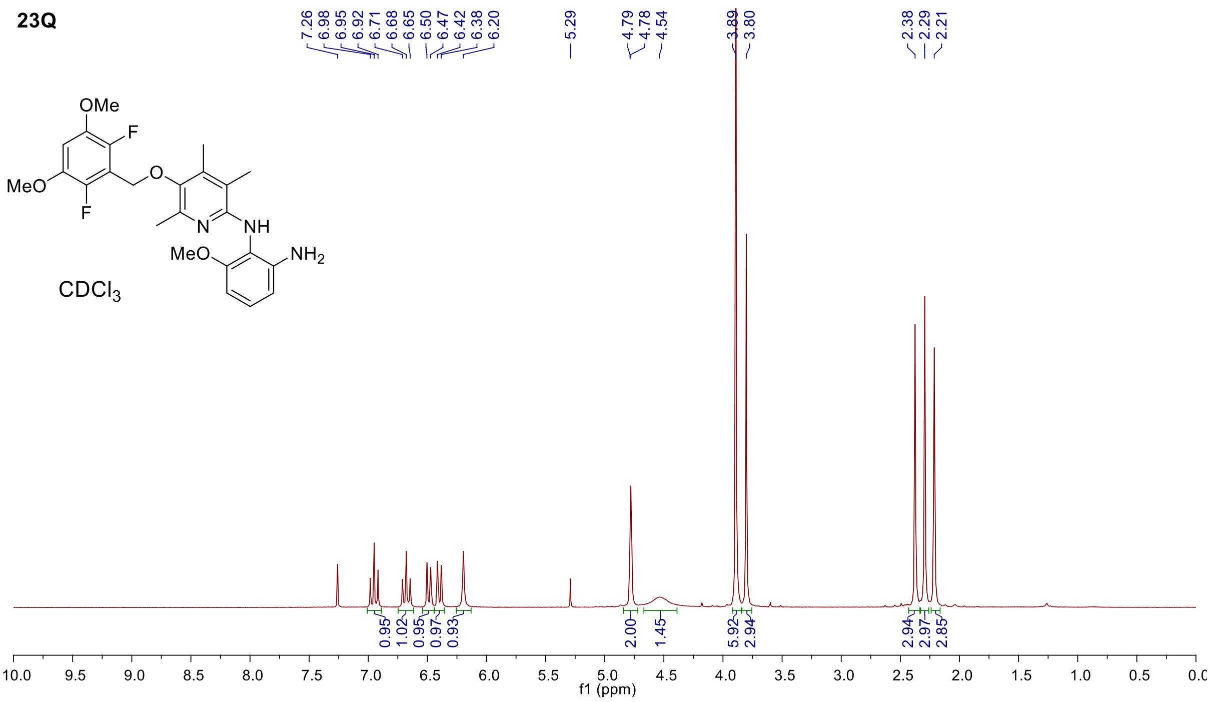
23Q
7.26 6.98 6.95 6.92 6.71 6.68 6.65 6.50 6.47 6.42 6.38 6.20
5.29
4.79 4.78 4.54
3.89 3.80
2.38 2.29 2.21
OMe
F
MeO
O
F
N
NH
MeO
NH2
CDCl3
0.95 1.02 0.95 0.97 0.93
2.00 1.45
5.92 2.94
2.94 2.97 2.85
f1 (ppm)

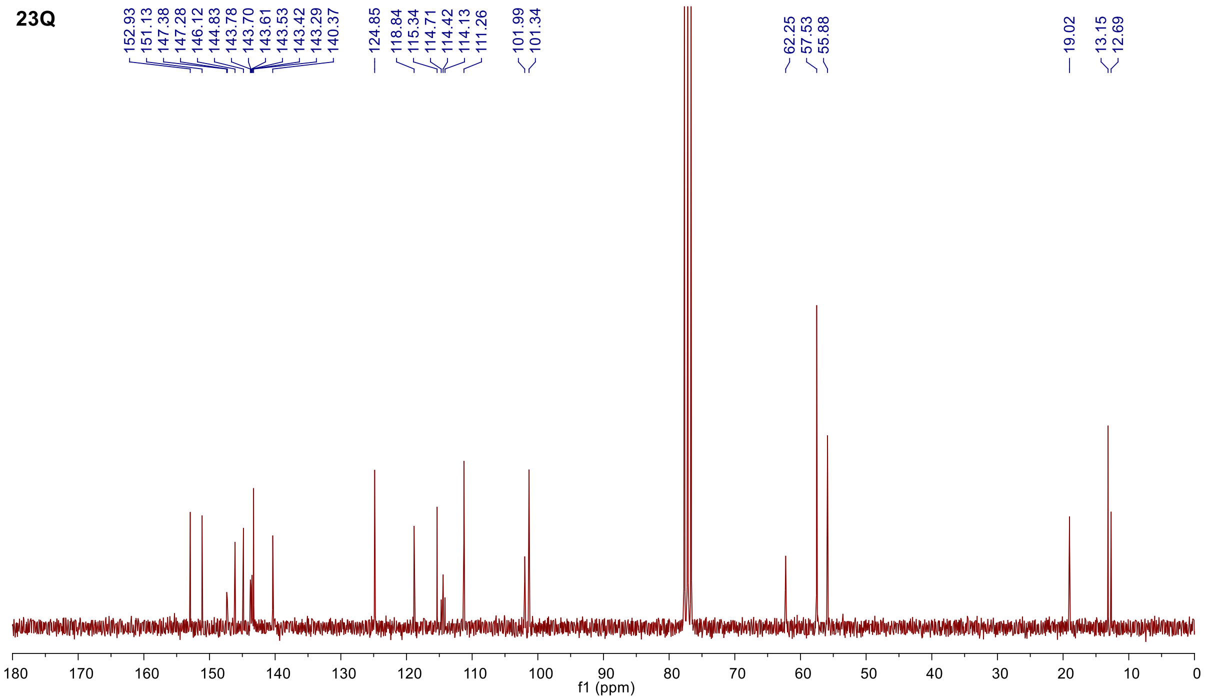
23Q
152.93 151.13 147.38 147.28 146.12 144.83 143.78 143.70 143.61 143.53 143.42 143.29 140.37
124.85 118.84 115.34 114.71 114.42 114.13 111.26
101.99 101.34
62.25 57.53 55.88
19.02 13.15 12.69
f1 (ppm)

23R

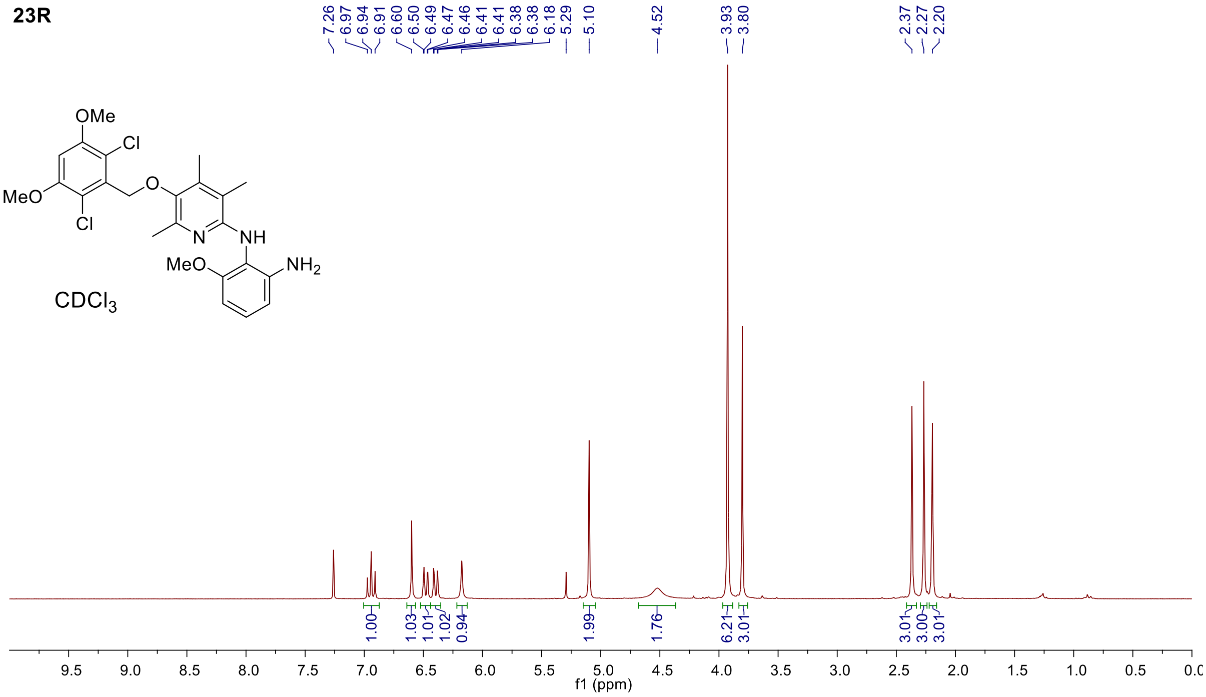
7.26 6.97 6.94 6.91 6.60 6.50 6.49 6.47 6.46 6.41 6.41 6.38 6.38 6.18 5.29 5.10
4.52
3.93 3.80
2.37 2.27 2.20
OMe
Cl
MeO
O
Cl
N
NH
MeO
NH2
CDCl3
1.00 1.03 1.01 1.02 0.94 1.99 1.76 6.21 3.01 3.01 3.00 3.01
9.5 9.0 8.5 8.0 7.5 7.0 6.5 6.0 5.5 5.0 4.5 4.0 3.5 3.0 2.5 2.0 1.5 1.0 0.5 0.0
f1 (ppm)

23R

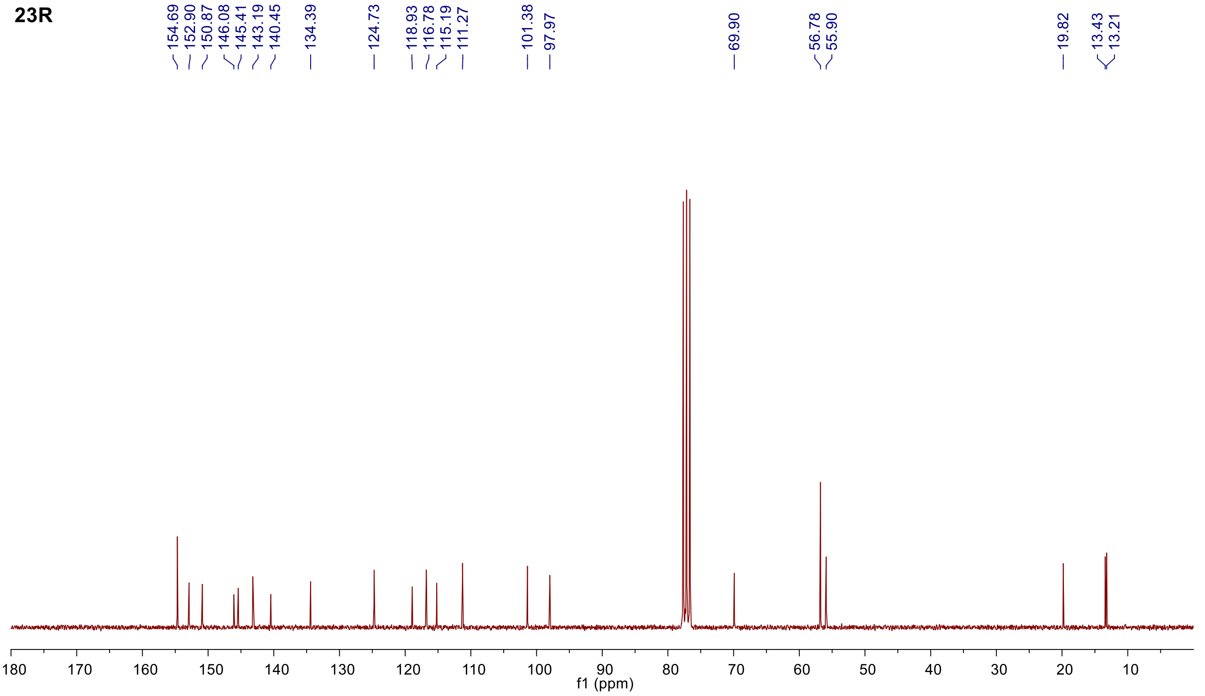
154.69 152.90 150.87 146.08 145.41 143.19 140.45 134.39 124.73 118.93 116.78 115.19 111.27 101.38 97.97
69.90
56.78 55.90
19.82 13.43 13.21
180 170 160 150 140 130 120 110 100 90 80 70 60 50 40 30 20 10
f1 (ppm)

23S

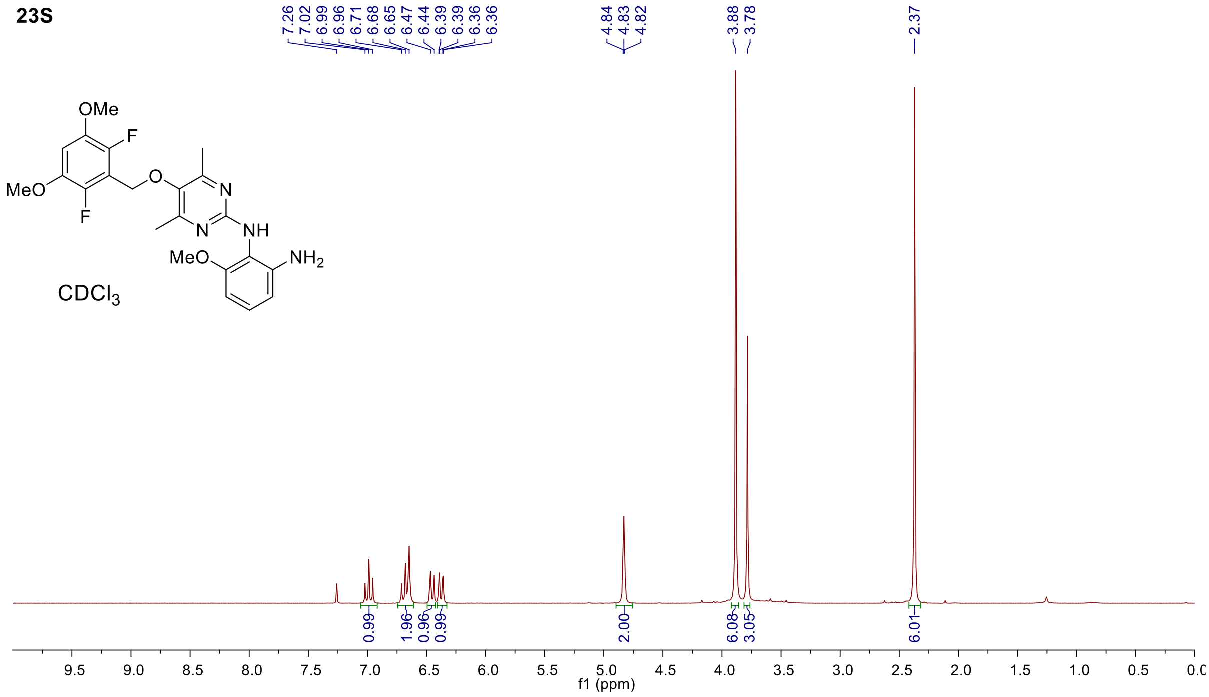
7.26 7.02 6.99 6.96 6.71 6.68 6.65 6.47 6.44 6.39 6.39 6.36 6.36
4.84 4.83 4.82
3.88 3.78
2.37
OMe
F
MeO
O
N
N
NH
F
MeO
NH2
CDCl3
0.99 1.96 0.96 0.99 2.00 6.08 3.05 6.01
f1 (ppm)

23S

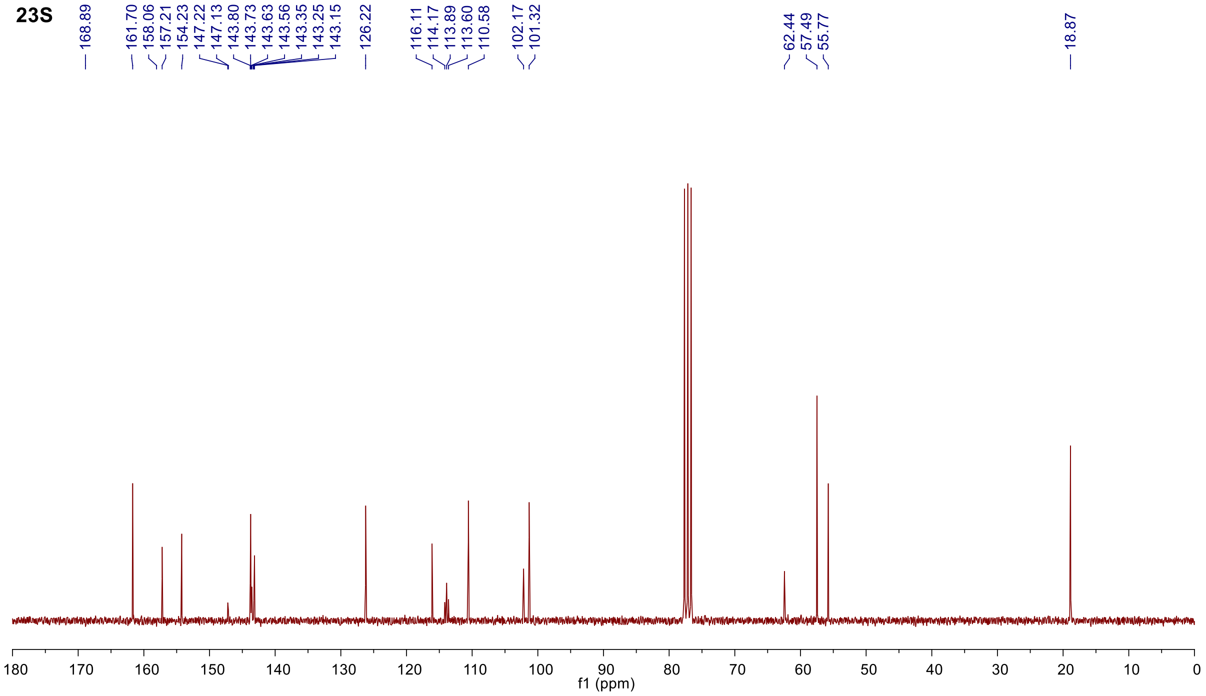
168.89 161.70 158.06 157.21 154.23 147.22 147.13 143.80 143.73 143.63 143.56 143.35 143.25 143.15 126.22 116.11 114.17 113.89 113.60 110.58 102.17 101.32
62.44 57.49 55.77
18.87
f1 (ppm)

23T

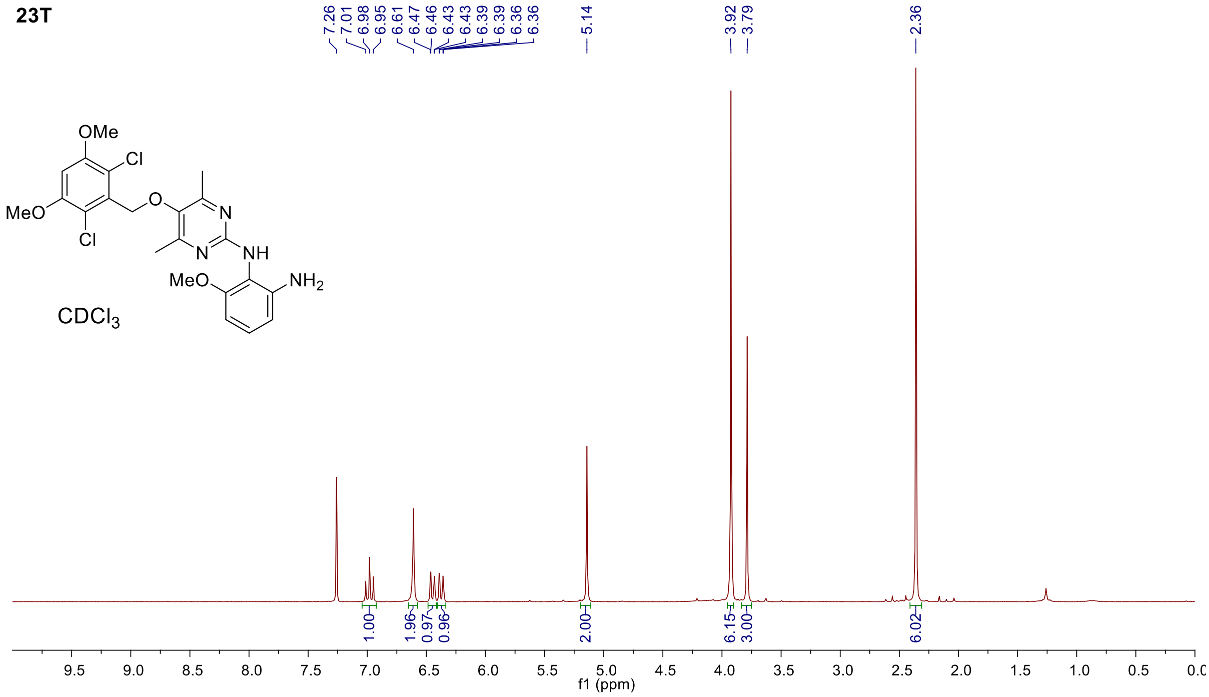

7.26
7.01
6.98
6.95
6.61
6.47
6.46
6.43
6.43
6.39
6.39
6.36
6.36
5.14
3.92
3.79
2.36
OMe
Cl
MeO
Cl
O
N
N
NH
MeO
NH2
CDCl3
1.00
1.96
0.97
0.96
2.00
6.15
3.00
6.02
f1 (ppm)

23T

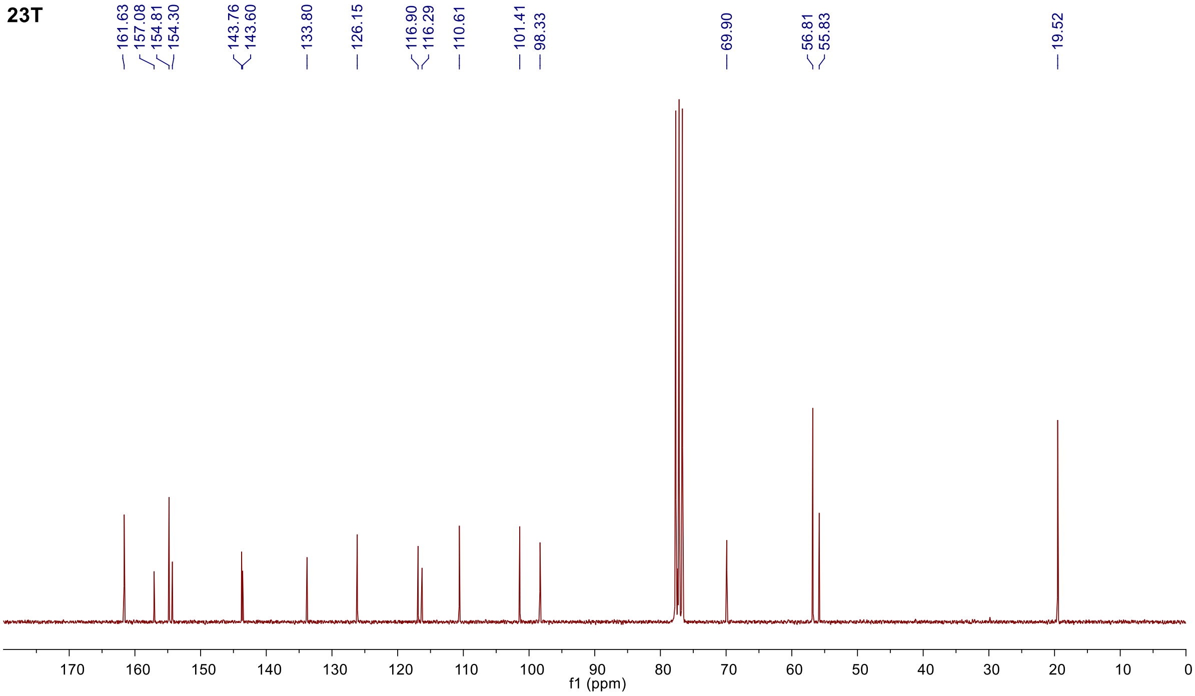

161.63
157.08
154.81
154.30
143.76
143.60
133.80
126.15
116.90
116.29
110.61
101.41
98.33
69.90
56.81
55.83
19.52
f1 (ppm)

23U

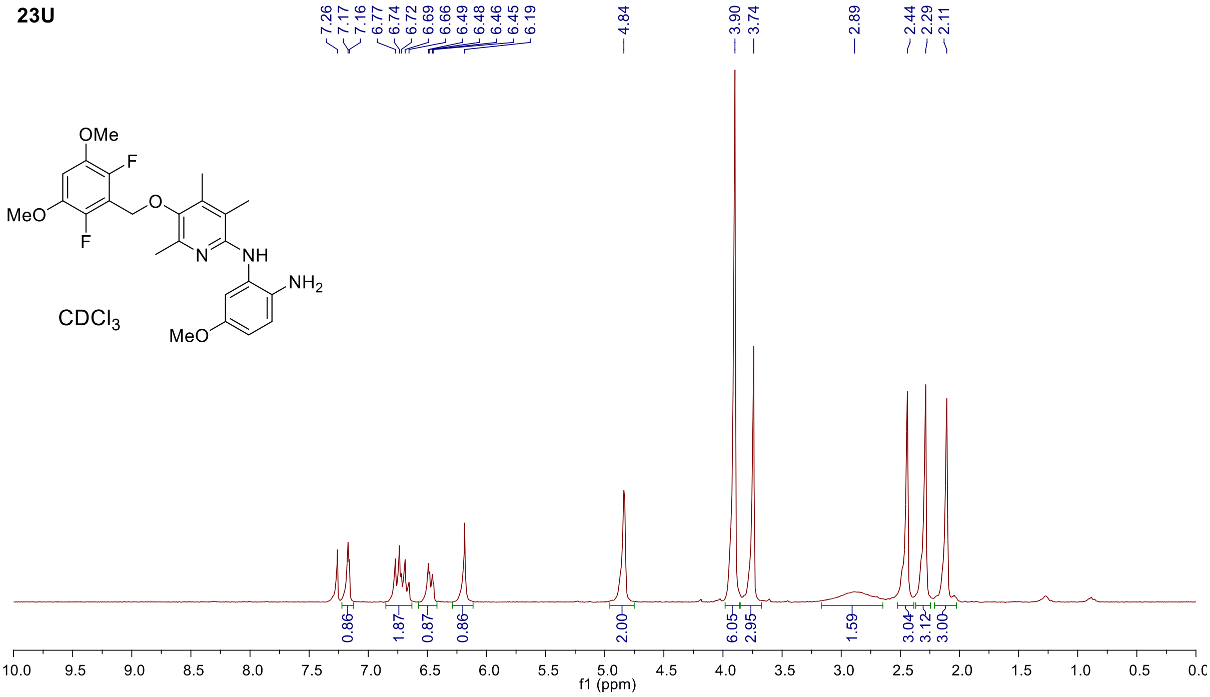
OMe
F
MeO
F
O
N
NH
NH2
MeO
CDCl3
7.26
7.17
7.16
6.77
6.74
6.72
6.69
6.66
6.49
6.48
6.46
6.45
6.19
4.84
3.90
3.74
2.89
2.44
2.29
2.11
0.86
1.87
0.87
0.86
2.00
6.05
2.95
1.59
3.04
3.12
3.00
f1 (ppm)

23U

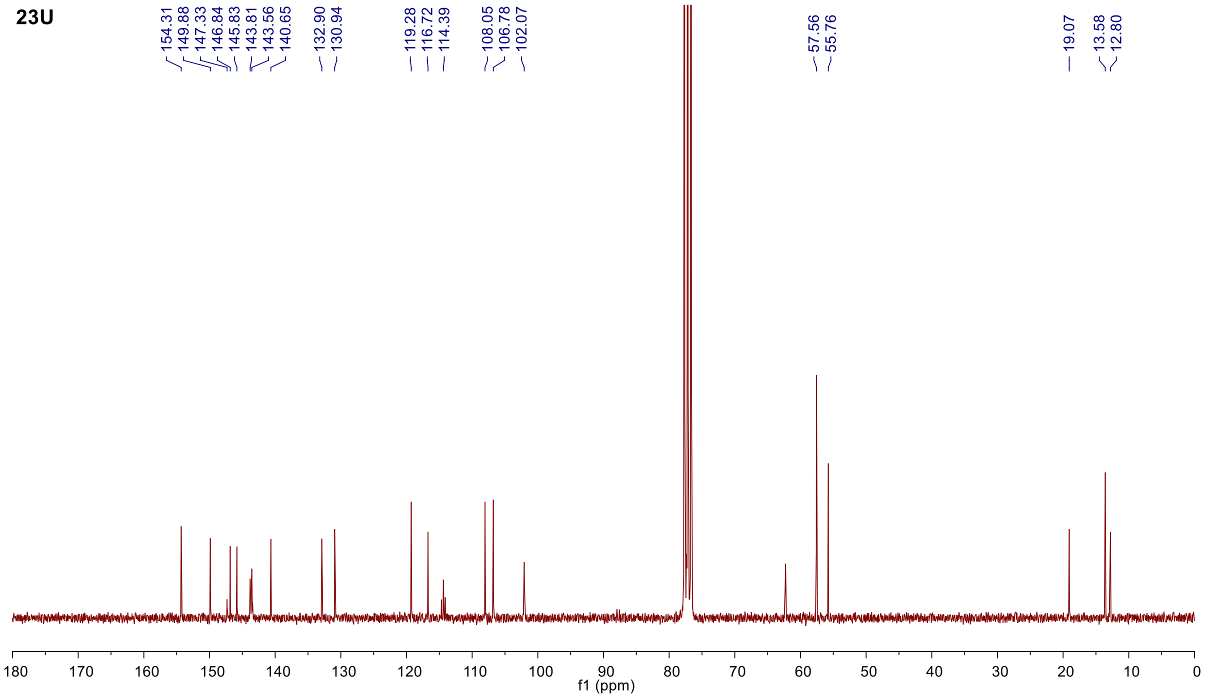
154.31
149.88
147.33
146.84
145.83
143.81
143.56
140.65
132.90
130.94
119.28
116.72
114.39
108.05
106.78
102.07
57.56
55.76
19.07
13.58
12.80
f1 (ppm)

23V

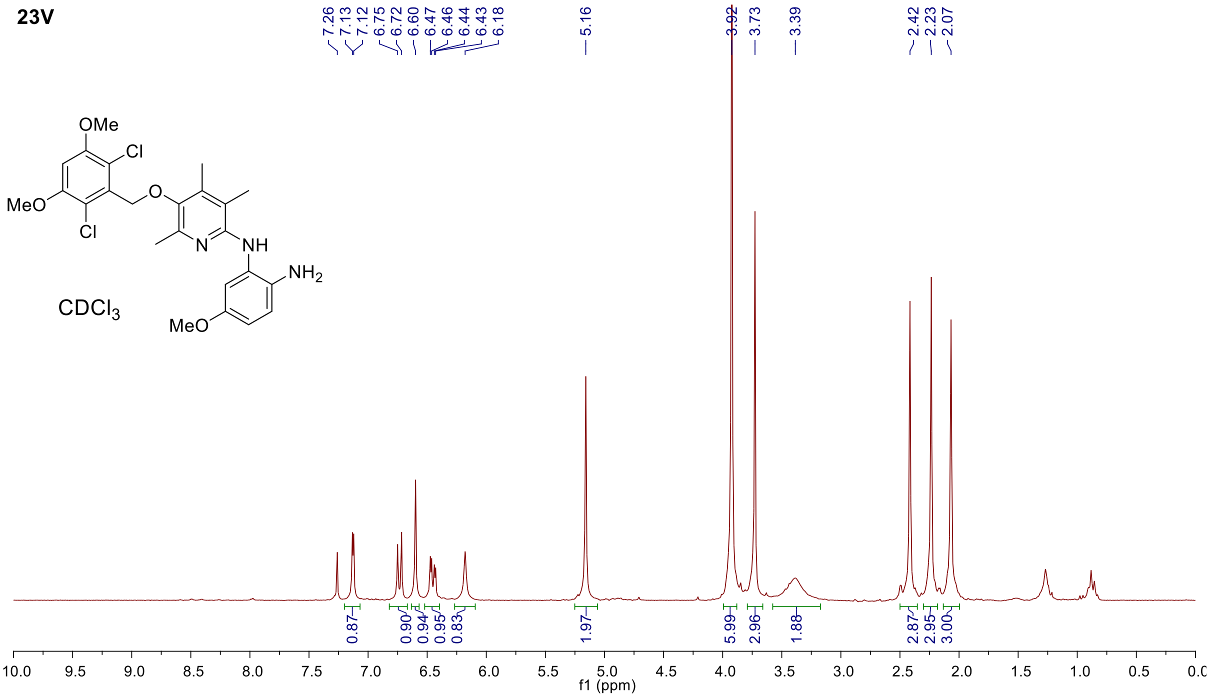
7.26
7.13
7.12
6.75
6.72
6.60
6.47
6.46
6.44
6.43
6.18
5.16
3.92
3.73
3.39
2.42
2.23
2.07
OMe
Cl
MeO
O
Cl
N
NH
NH_2
MeO
$CDCl_3$
0.87
0.90
0.94
0.95
0.83
1.97
5.99
2.96
1.88
2.87
2.95
3.00
10.0 9.5 9.0 8.5 8.0 7.5 7.0 6.5 6.0 5.5 5.0 4.5 4.0 3.5 3.0 2.5 2.0 1.5 1.0 0.5 0.0
f1 (ppm)

23V

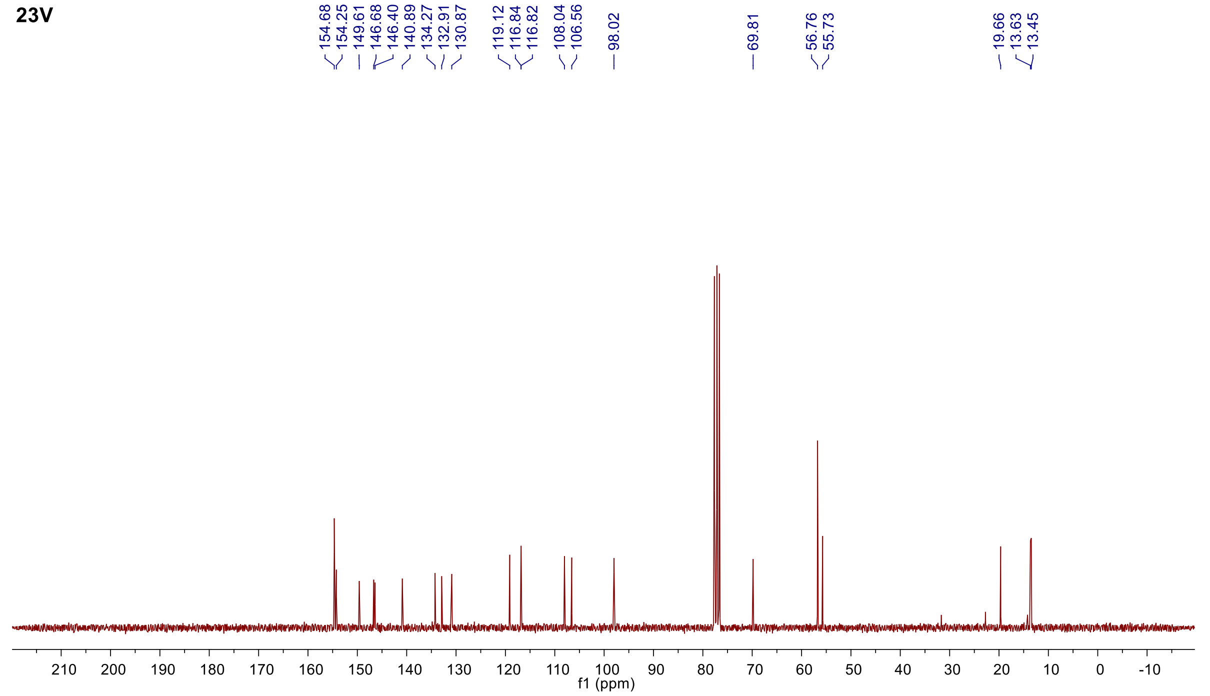
154.68
154.25
149.61
146.68
146.40
140.89
134.27
132.91
130.87
119.12
116.84
116.82
108.04
106.56
98.02
69.81
56.76
55.73
19.66
13.63
13.45
210 200 190 180 170 160 150 140 130 120 110 100 90 80 70 60 50 40 30 20 10 0 -10
f1 (ppm)

23W

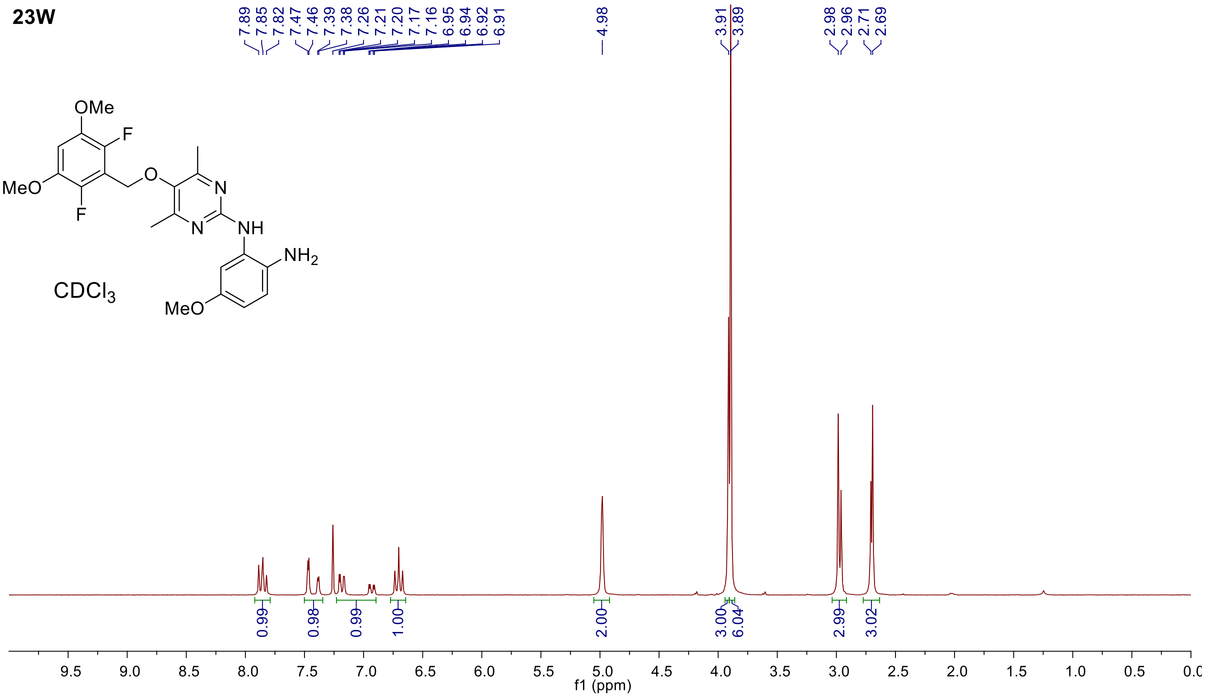
7.89
7.85
7.82
7.47
7.46
7.39
7.38
7.26
7.21
7.20
7.17
7.16
6.95
6.94
6.92
6.91
4.98
3.91
3.89
2.98
2.96
2.71
2.69
OMe
F
MeO
F
O
N
N
NH
NH$_2$
MeO
CDCl$_3$
0.99
0.98
0.99
1.00
2.00
3.00
6.04
2.99
3.02
9.5
9.0
8.5
8.0
7.5
7.0
6.5
6.0
5.5
5.0
4.5
4.0
3.5
3.0
2.5
2.0
1.5
1.0
0.5
f1 (ppm)

23W

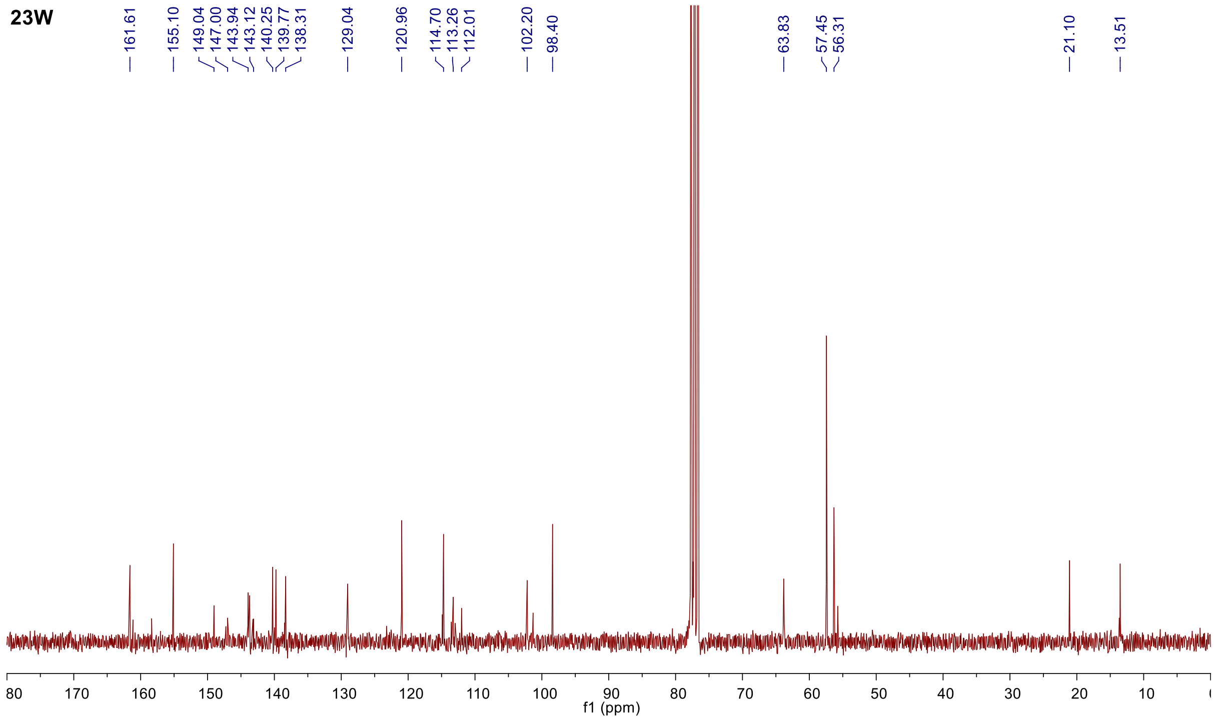
161.61
155.10
149.04
147.00
143.94
143.12
140.25
139.77
138.31
129.04
120.96
114.70
113.26
112.01
102.20
98.40
63.83
57.45
56.31
21.10
13.51
170
160
150
140
130
120
110
100
90
80
70
60
50
40
30
20
10
f1 (ppm)

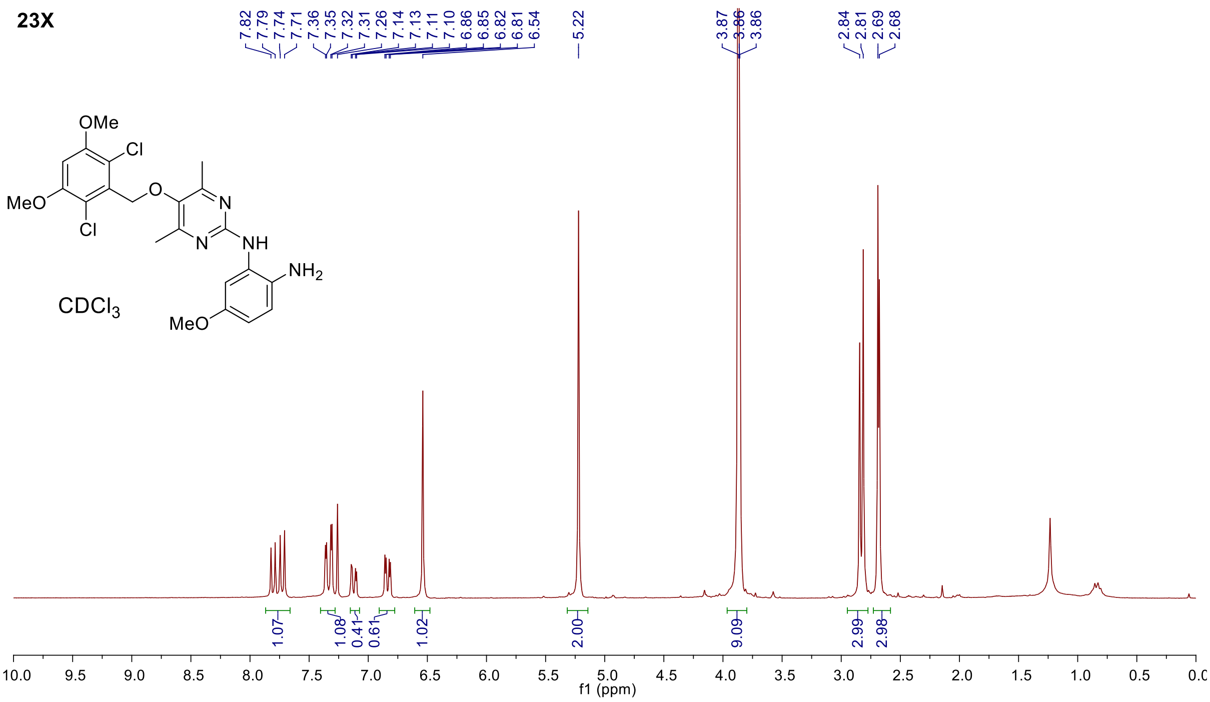
23X
CDCl3
OMe
Cl
MeO
O
N
NH
N
NH2
MeO
7.82
7.79
7.74
7.71
7.36
7.35
7.32
7.31
7.26
7.14
7.13
7.11
7.10
6.86
6.85
6.82
6.81
6.54
5.22
3.87
3.86
3.86
2.84
2.81
2.69
2.68
1.07
1.08
0.41
0.61
1.02
2.00
9.09
2.99
2.98
f1 (ppm)

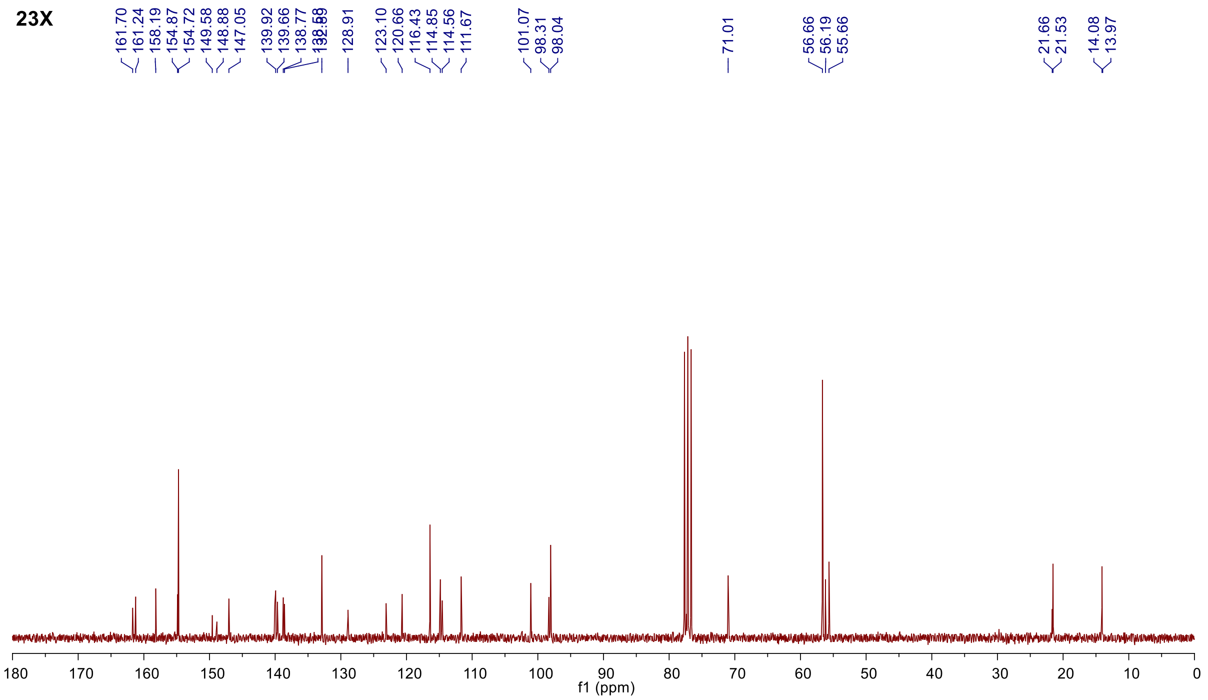
23X
161.70
161.24
158.19
154.87
154.72
149.58
148.88
147.05
139.92
139.66
138.77
128.91
123.10
120.66
116.43
114.85
114.56
111.67
101.07
98.31
98.04
71.01
56.66
56.19
55.66
21.66
21.53
14.08
13.97
f1 (ppm)

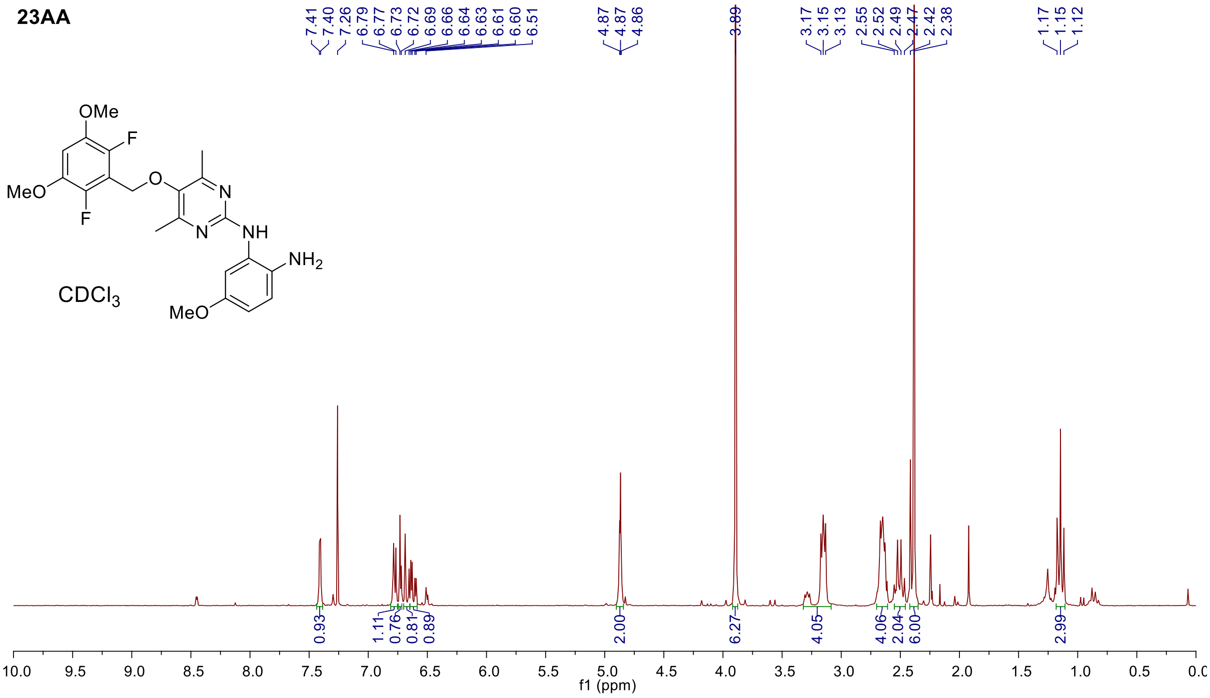
23AA
OMe
F
MeO
O
N
NH
F
N
NH2
CDCl3
MeO
7.41
7.40
7.26
6.79
6.77
6.73
6.72
6.69
6.66
6.64
6.63
6.61
6.60
6.51
4.87
4.87
4.86
3.89
3.17
3.15
3.13
2.55
2.52
2.49
2.47
2.42
2.38
1.17
1.15
1.12
0.93
1.11
0.76
0.81
0.89
2.00
6.27
4.05
4.06
2.04
6.00
2.99
f1 (ppm)

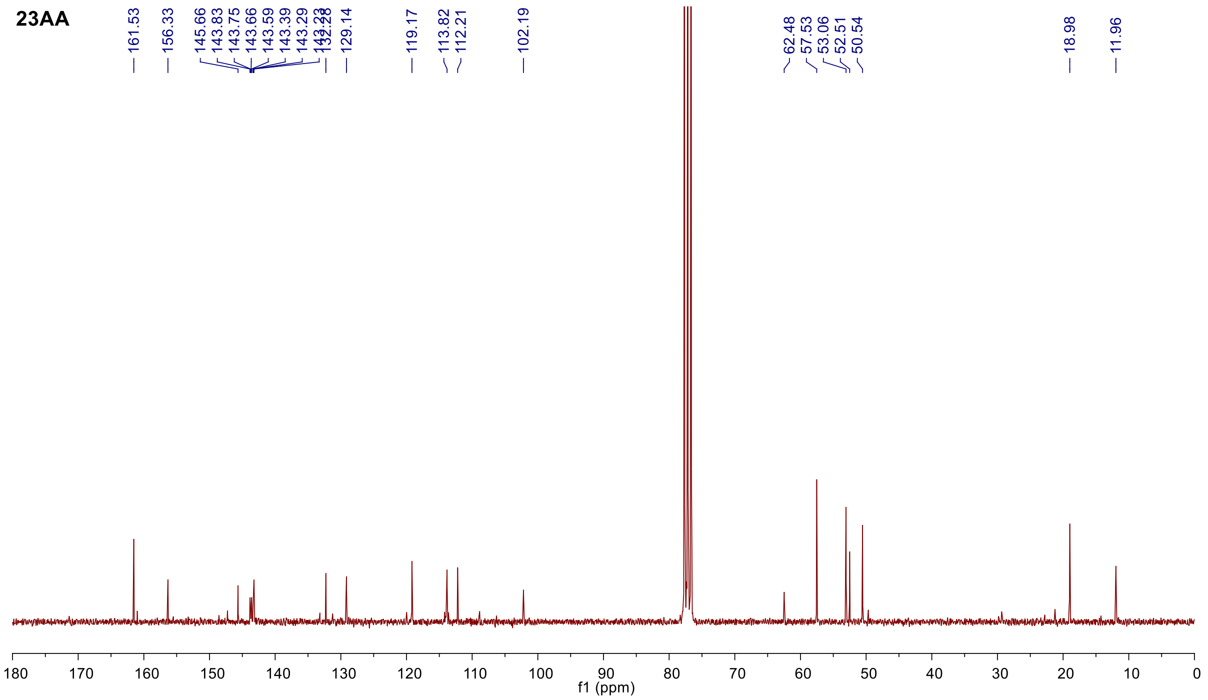
23AA
161.53
156.33
145.66
143.83
143.75
143.66
143.59
143.39
143.29
129.14
119.17
113.82
112.21
102.19
62.48
57.53
53.06
52.51
50.54
18.98
11.96
f1 (ppm)

23BB

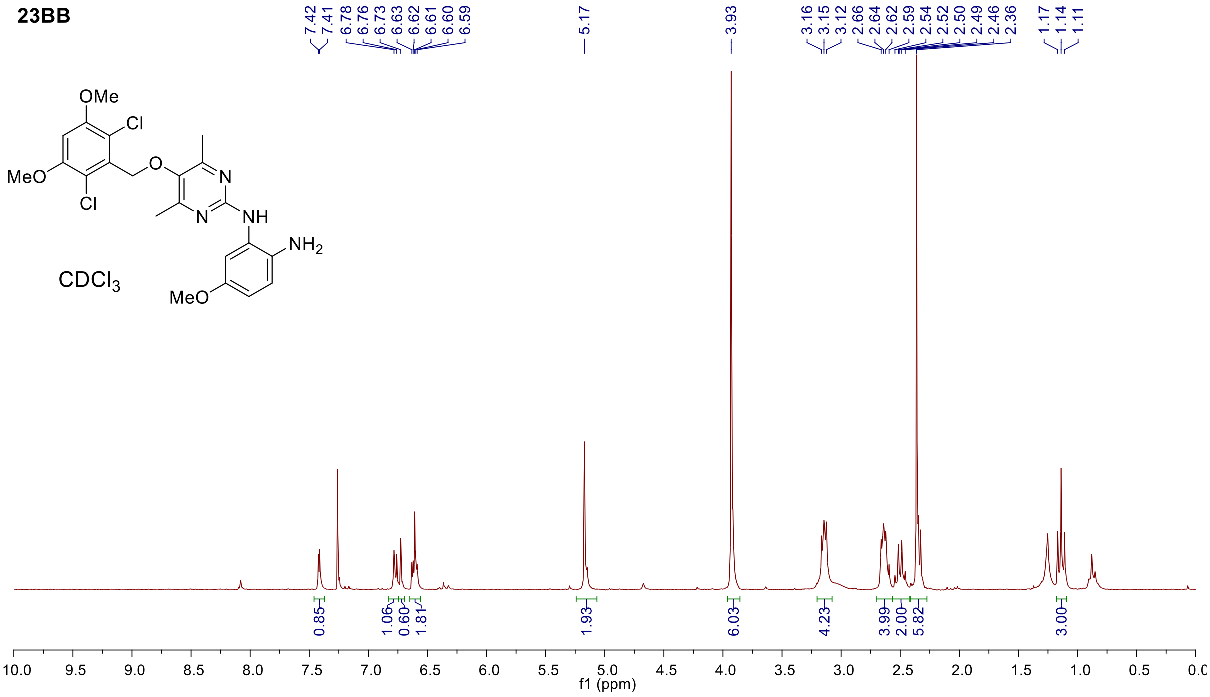
7.42
7.41
6.78
6.76
6.73
6.63
6.62
6.61
6.60
6.59
5.17
3.93
3.16
3.15
3.12
2.66
2.64
2.62
2.59
2.54
2.52
2.50
2.49
2.46
2.36
1.17
1.14
1.11
OMe
Cl
MeO
O
N
Cl
N
NH
NH2
CDCl3
MeO
0.85
1.06
0.60
1.81
1.93
6.03
4.23
3.99
2.00
5.82
3.00
10.0 9.5 9.0 8.5 8.0 7.5 7.0 6.5 6.0 5.5 5.0 4.5 4.0 3.5 3.0 2.5 2.0 1.5 1.0 0.5 0.0
f1 (ppm)

23BB

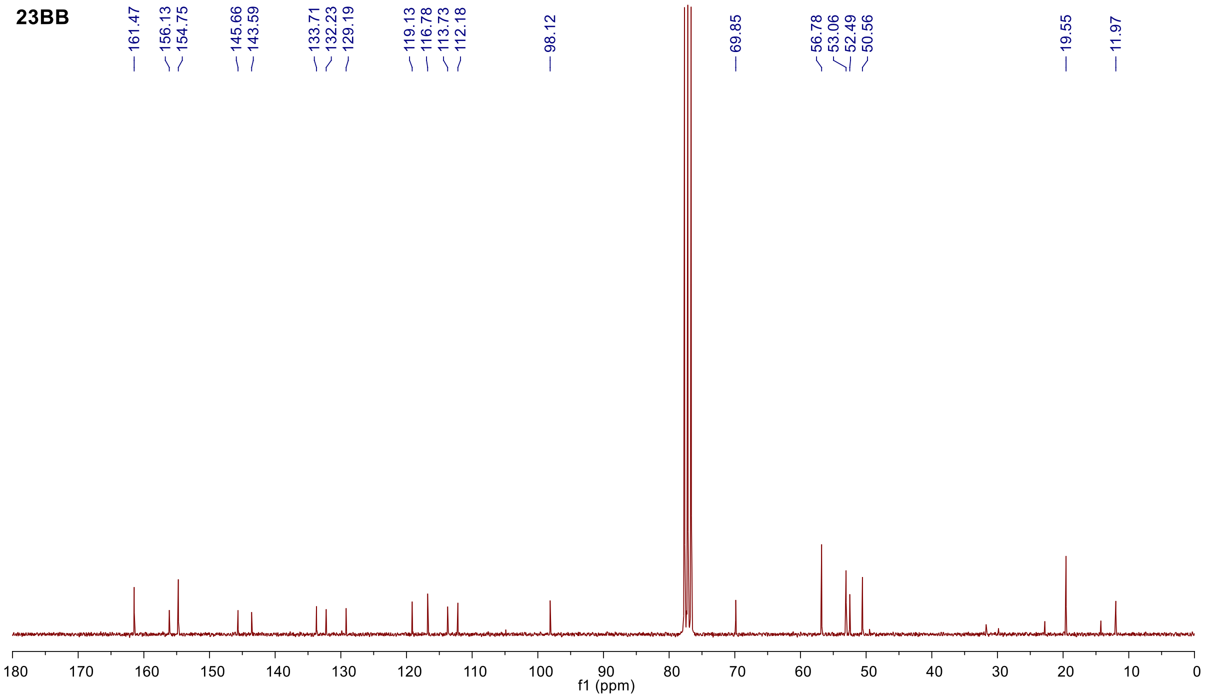
161.47
156.13
154.75
145.66
143.59
133.71
132.23
129.19
119.13
116.78
113.73
112.18
98.12
69.85
56.78
53.06
52.49
50.56
19.55
11.97
180 170 160 150 140 130 120 110 100 90 80 70 60 50 40 30 20 10 0
f1 (ppm)

41

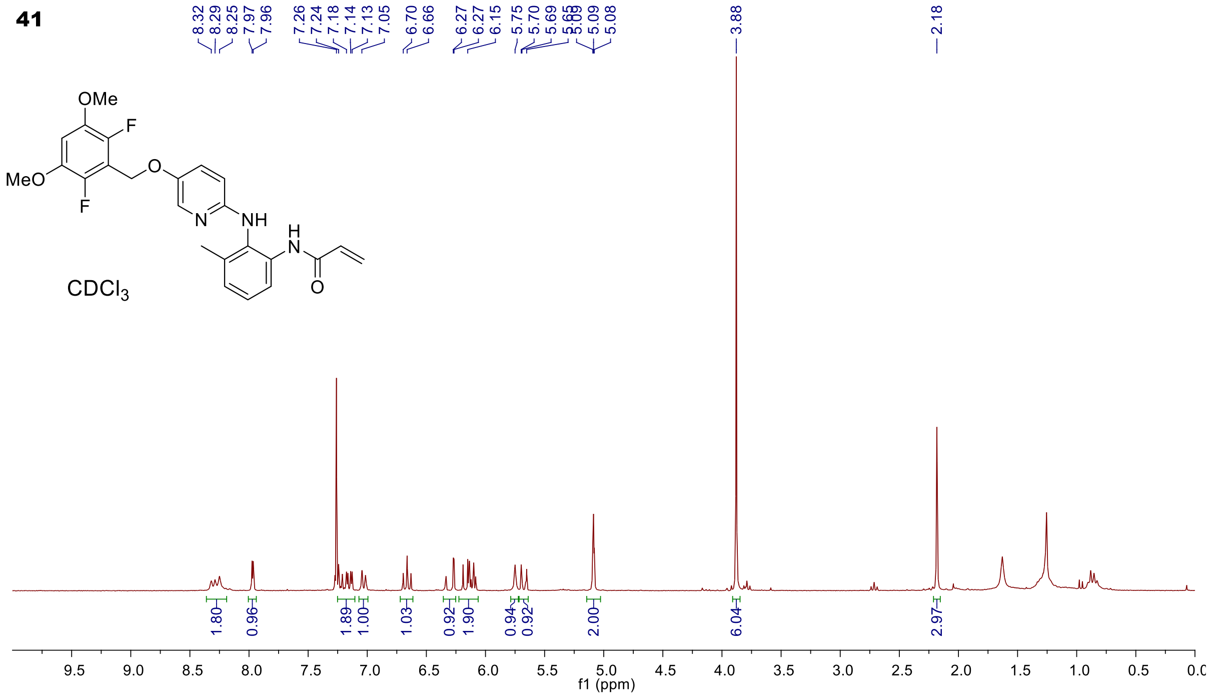

41

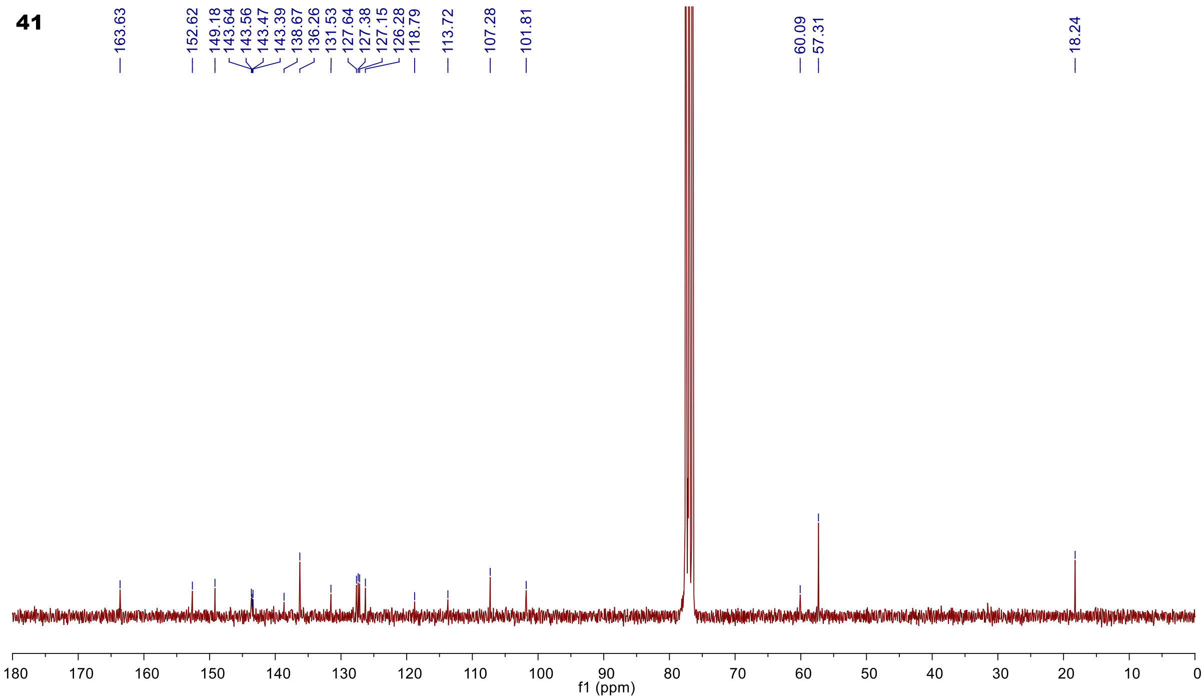

42

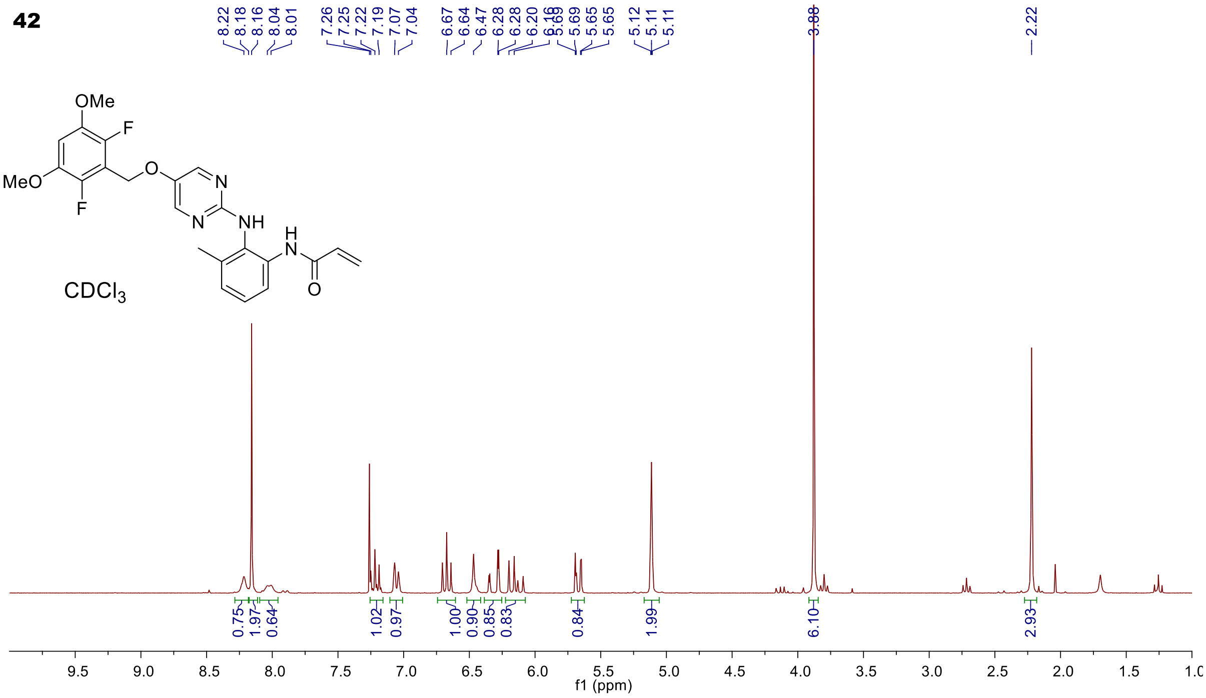
8.22
8.18
8.16
8.04
8.01
7.26
7.25
7.22
7.19
7.07
7.04
6.67
6.64
6.47
6.28
6.28
6.20
5.69
5.65
5.65
5.12
5.11
5.11
3.88
2.22
OMe
F
MeO
O
N
F
N
NH
H
N
O
CDCl3
0.75
1.97
0.64
1.02
0.97
1.00
0.90
0.85
0.83
0.84
1.99
6.10
2.93
f1 (ppm)

42

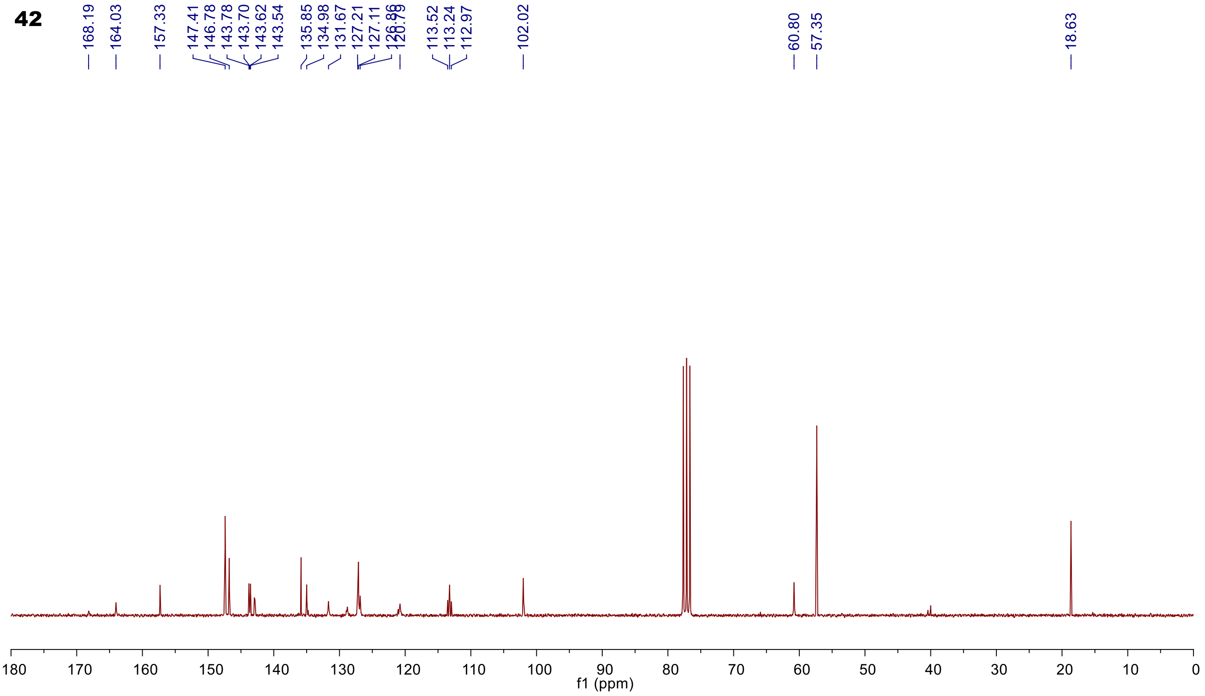
168.19
164.03
157.33
147.41
146.78
143.78
143.70
143.62
143.54
135.85
134.98
131.67
127.21
127.11
113.52
113.24
112.97
102.02
60.80
57.35
18.63
f1 (ppm)

47

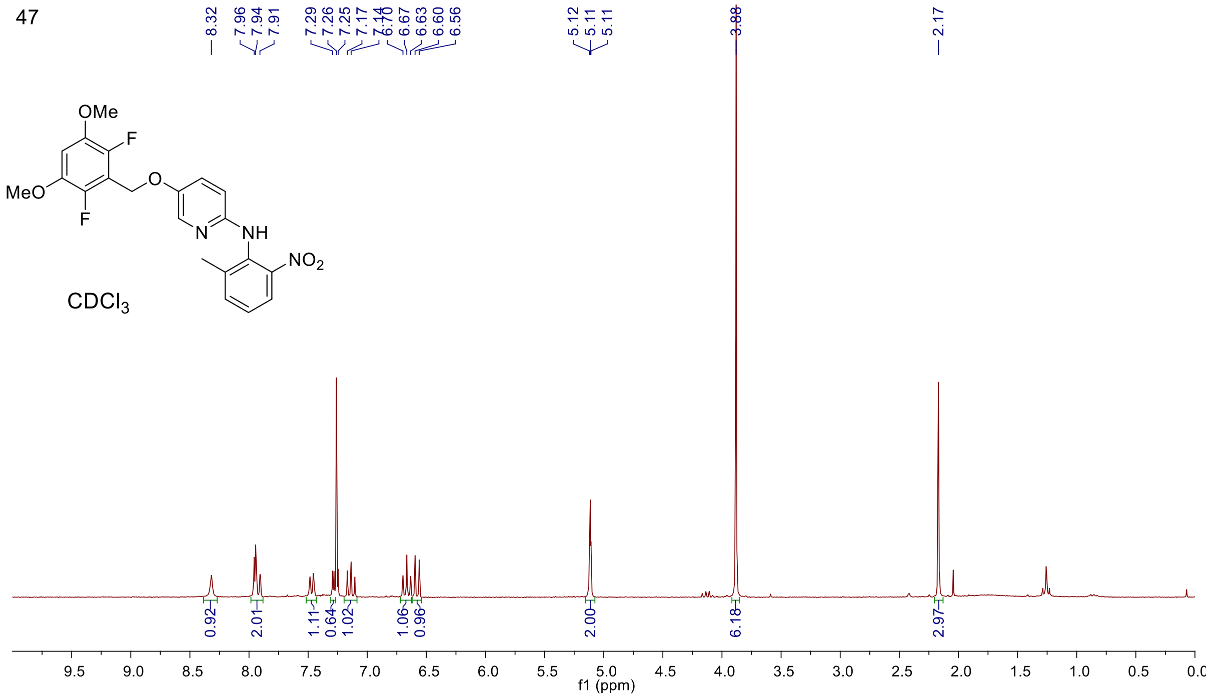
8.32
7.96
7.94
7.91
7.29
7.26
7.25
7.17
7.14
[illegible]
6.70
6.67
6.63
6.60
6.56
5.12
5.11
5.11
3.88
2.17
OMe
F
MeO
F
O
N
NH
NO2
CDCl3
0.92
2.01
1.11
0.64
1.02
1.06
0.96
2.00
6.18
2.97
f1 (ppm)

47

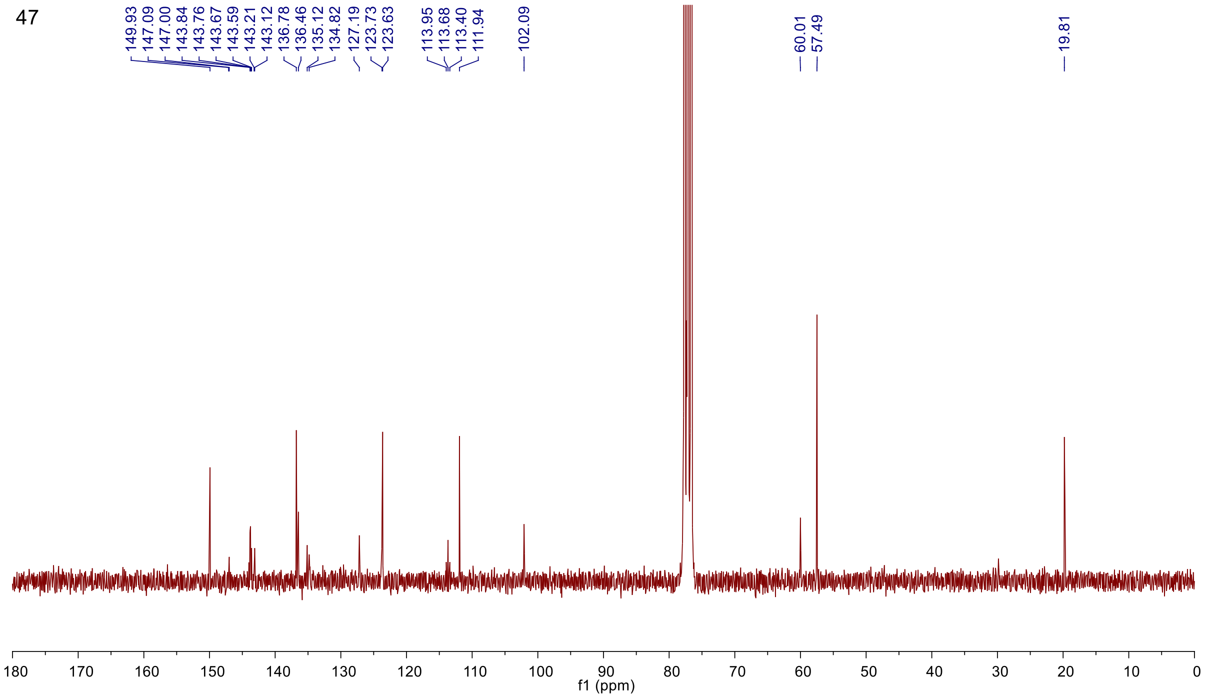
149.93
147.09
147.00
143.84
143.76
143.67
143.59
143.21
143.12
136.78
136.46
135.12
134.82
127.19
123.73
123.63
113.95
113.68
113.40
111.94
102.09
60.01
57.49
19.81
f1 (ppm)

48

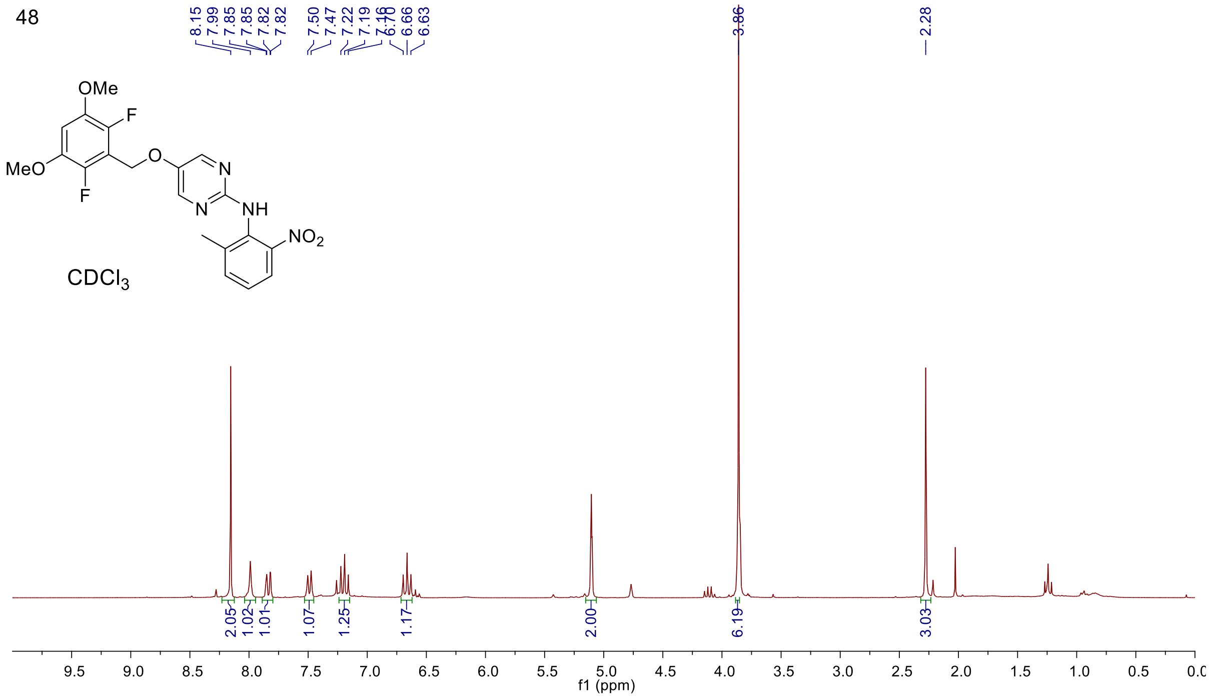
8.15
7.99
7.85
7.85
7.82
7.82
7.50
7.47
7.22
7.19
7.16
6.70
6.66
6.63
3.86
2.28
OMe
F
MeO
O
N
N
NH
F
NO2
CDCl3
2.05
1.02
1.01
1.07
1.25
1.17
2.00
6.19
3.03
9.5 9.0 8.5 8.0 7.5 7.0 6.5 6.0 5.5 5.0 4.5 4.0 3.5 3.0 2.5 2.0 1.5 1.0 0.5 0.0
f1 (ppm)

48

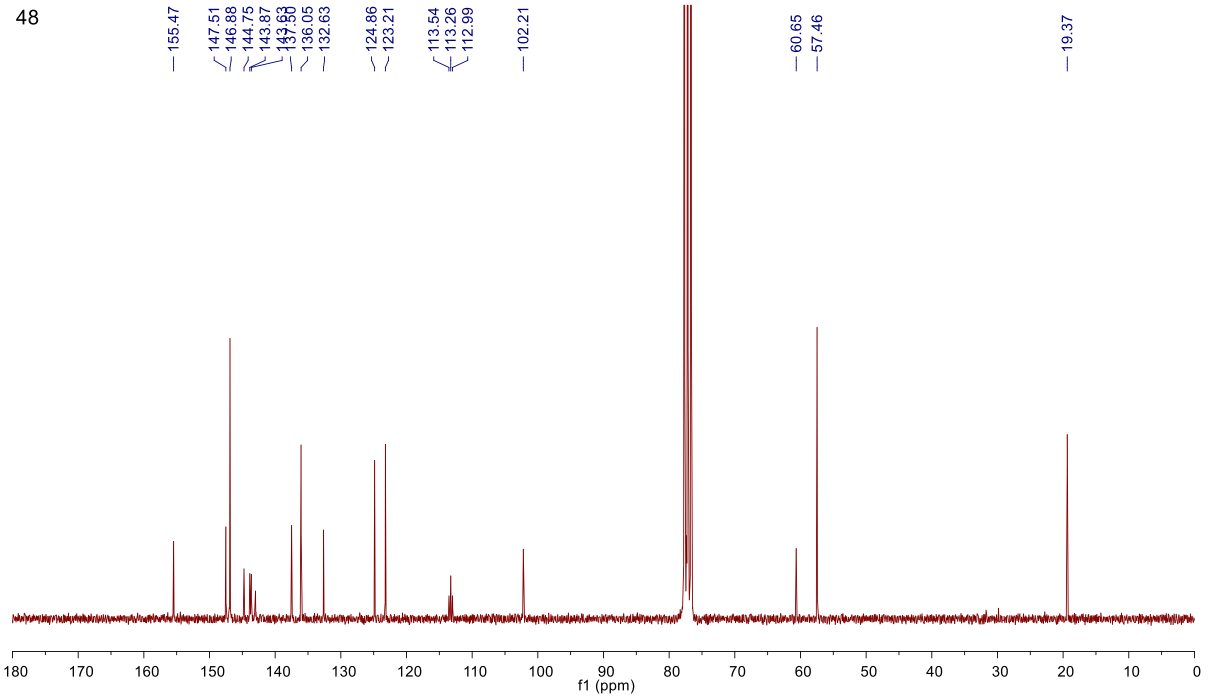
155.47
147.51
146.88
144.75
143.87
143.63
137.50
136.05
132.63
124.86
123.21
113.54
113.26
112.99
102.21
60.65
57.46
19.37
180 170 160 150 140 130 120 110 100 90 80 70 60 50 40 30 20 10 0
f1 (ppm)

49

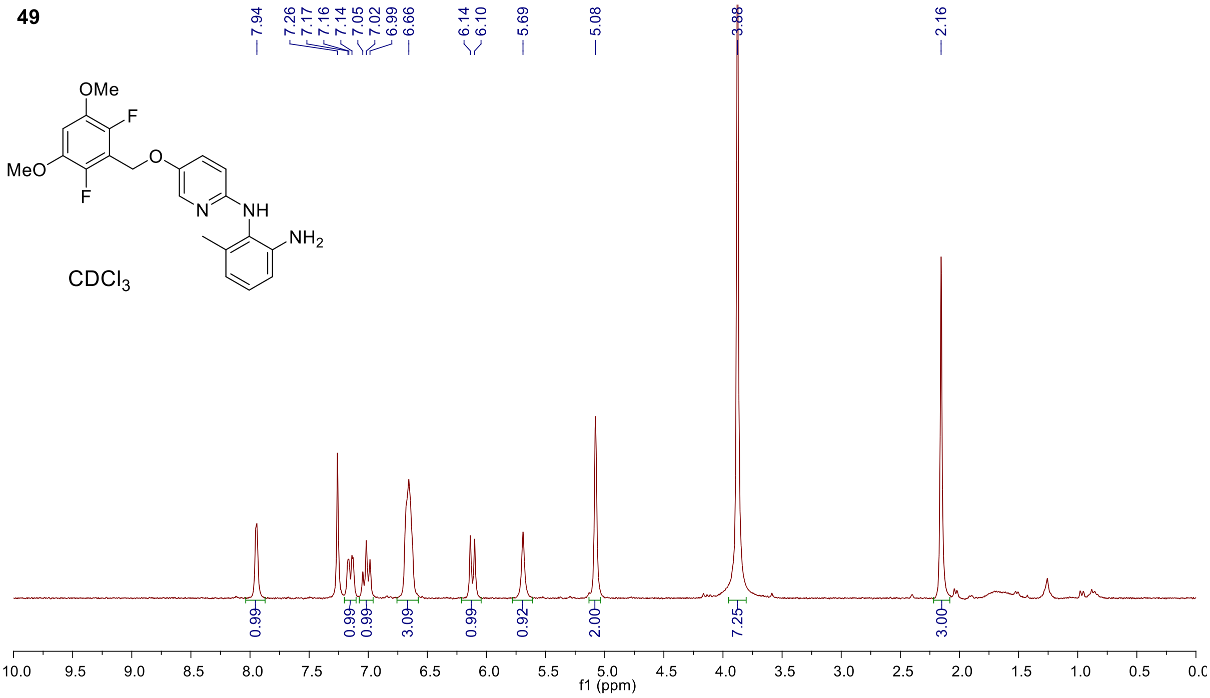

7.94
7.26
7.17
7.16
7.14
7.05
7.02
6.99
6.66
6.14
6.10
5.69
5.08
3.88
2.16
OMe
F
MeO
F
O
N
NH
NH2
CDCl3
0.99
0.99
0.99
3.09
0.99
0.92
2.00
7.25
3.00
f1 (ppm)

49

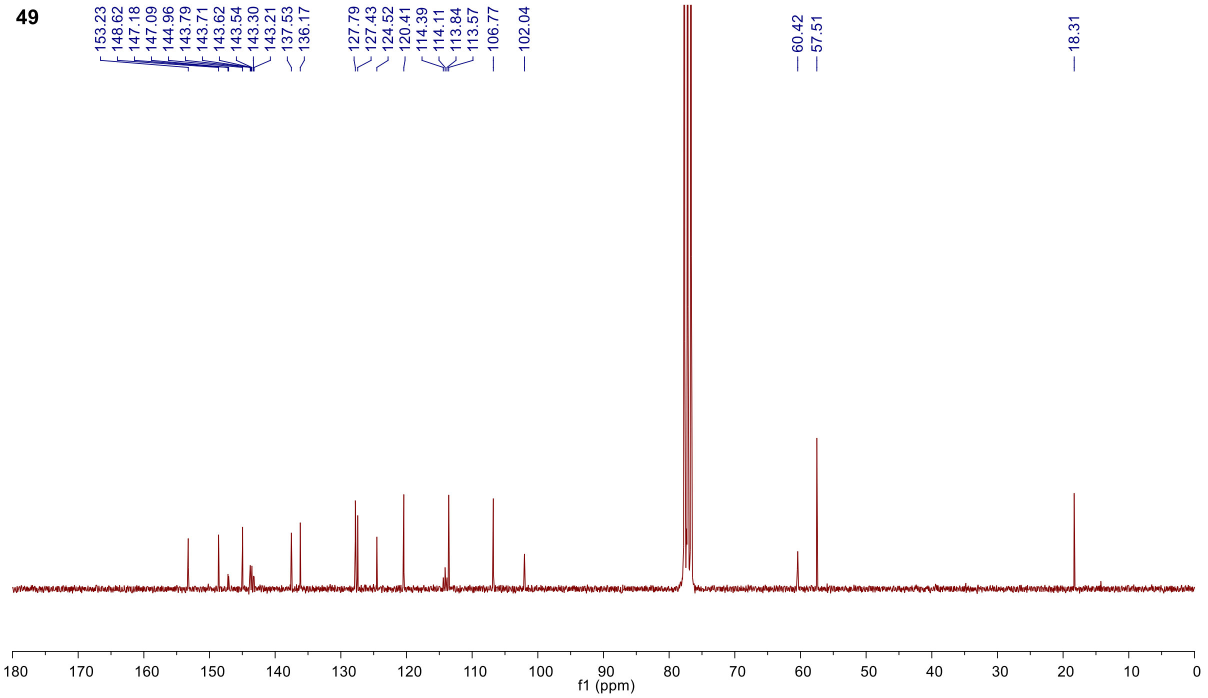

153.23
148.62
147.18
147.09
144.96
143.79
143.71
143.62
143.54
143.30
143.21
137.53
136.17
127.79
127.43
124.52
120.41
114.39
114.11
113.84
113.57
106.77
102.04
60.42
57.51
18.31
f1 (ppm)

50

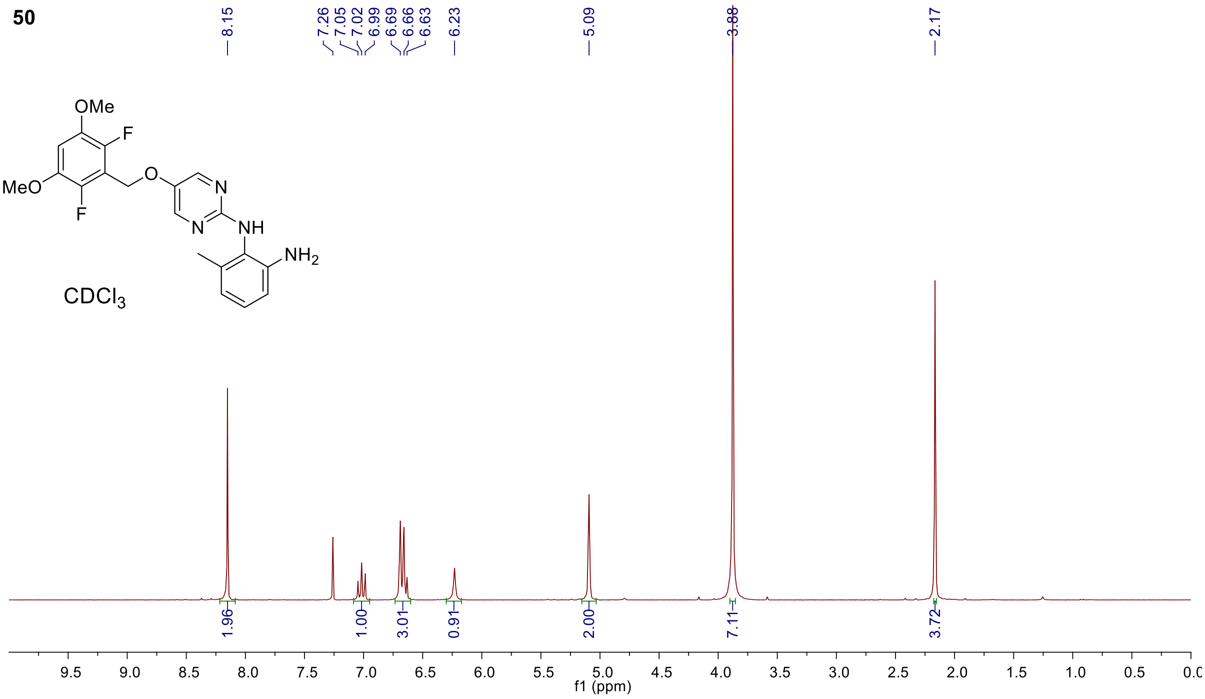
8.15
7.26
7.05
7.02
6.99
6.69
6.66
6.63
6.23
5.09
3.88
2.17
OMe
F
MeO
F
O
N
N
NH
NH2
CDCl3
1.96
1.00
3.01
0.91
2.00
7.11
3.72
9.5 9.0 8.5 8.0 7.5 7.0 6.5 6.0 5.5 5.0 4.5 4.0 3.5 3.0 2.5 2.0 1.5 1.0 0.5 0.0
f1 (ppm)

50

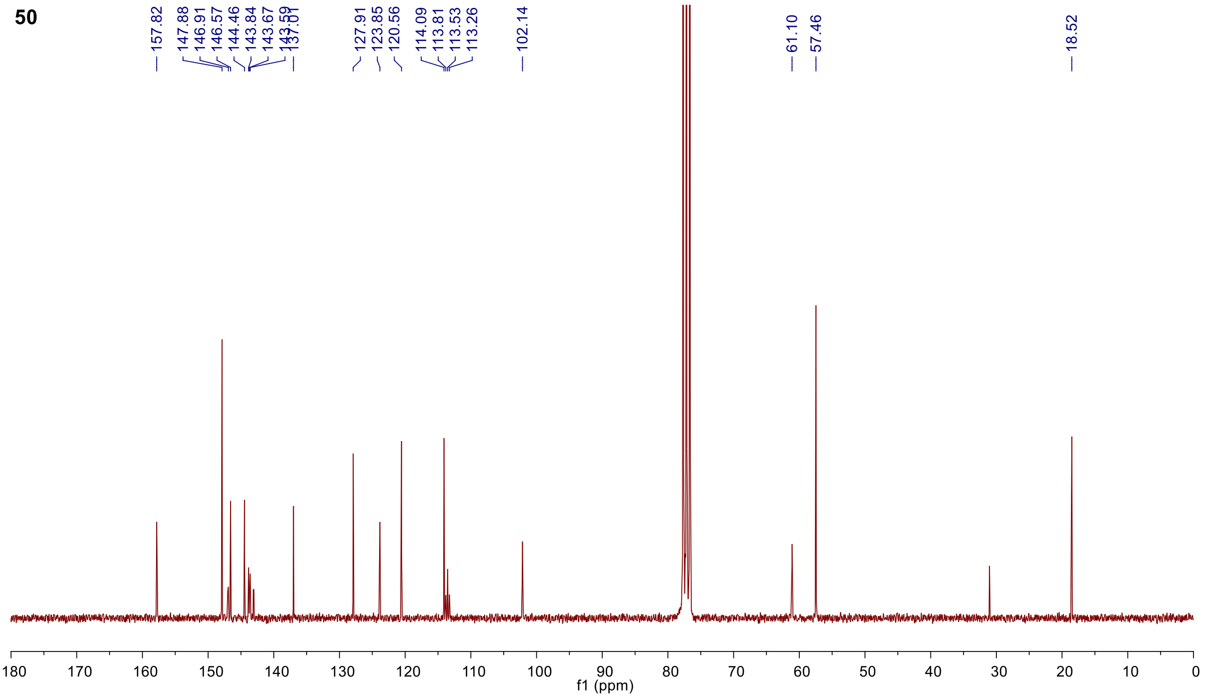
157.82
147.88
146.91
146.57
144.46
143.84
143.67
143.59
137.01
127.91
123.85
120.56
114.09
113.81
113.53
113.26
102.14
61.10
57.46
18.52
180 170 160 150 140 130 120 110 100 90 80 70 60 50 40 30 20 10 0
f1 (ppm)
